# Disease-stage-specific immunometabolic remodeling in pediatric obstructive sleep apnea: a single-cell transcriptomic atlas of adenoid tissue

**DOI:** 10.1038/s44321-026-00419-3

**Published:** 2026-04-27

**Authors:** Qin Yang, Yunfei Cui, Xiao Huang, Junlin Liu, Xiaopeng Ma, George Fu Gao, Hongguang Pan, Shijie Qin

**Affiliations:** 1 Department of Sleep Medicine Center, Shenzhen Children’s Hospital, Shenzhen, 518038 China; 2 Department of Respiratory Medicine, Shenzhen Children’s Hospital, Shenzhen, 518038 China; 3 Faculty of Health Sciences, University of Macau, Macau SAR, China; 4 CAS Key Laboratory of Pathogen Microbiology and Immunology, Institute of Microbiology, Chinese Academy of Sciences (CAS), Beijing, 100101 China; 5 Department of Otolaryngology, Shenzhen Children’s Hospital, Shenzhen, 518038 China; 6 Innovative Vaccine and Immunotherapy Research Center, The Second Affiliated Hospital Zhejiang University School of Medicine, Hangzhou, 310009 China

**Keywords:** Immunology, Metabolism, Methods & Resources

## Abstract

Hypertrophied adenoids in children can impair breathing and lead to obstructive sleep apnea (OSA), often accompanied by abnormal growth and weakened stamina and immunity. However, the cause of the pathological transformation in these originally immune-enhancing lymphoid tissues remains unclear. Our study provides the first single cell transcriptomic and immune repertoire atlas of adenoids from normal snoring to mild, moderate, and severe OSA, and identified markedly asynchronous functional modules, transcriptional regulatory networks and intercellular communications during the progression of OSA. Children with severe OSA exhibited exhibit active Hippo, Notch, and Wnt signaling, alongside significant downregulation of energy synthesis. Analysis revealed compromised T-cell and B-cell immunity, as well as reduced antigen processing by innate immune cells, coupled with diminished cell-cell communication in severe OSA group. T-cell receptor and B-cell receptor sequencing results also support more infection imprints and abnormal germinal centers and antibody class switching. Mechanistically, HIF1A-mediated hypoxic signaling likely drives the downregulation of key immune components (including HLA and interferon molecules), positioning it as a promising therapeutic target for OSA.

## Synopsis

Through single cell sequencing technology, this study reveals the stage-specific characteristics of adenoid tissues in children with obstructive sleep apnea (OSA), ranging from immune activation to functional exhaustion, and identifies the HIF1A signaling as a key mechanism and therapeutic target.

- Children with severe OSA exhibit core pathological features in adenoid tissues, including tissue disorganization, abnormal proliferation, and dysregulation of growth and metabolism.
- The immune and inflammatory landscape evolves with OSA progression, characterized by early-stage activation followed by T/B cell functional exhaustion and impaired antigen presentation in innate immune cells during severe phase.
- TCR and BCR analyses confirm that children with severe OSA display more infection imprints and impaired antibody maturation.
- HIF1A-mediated hypoxia signaling suppresses the transcription of immune-related molecules, representing a potential mechanism for the immune decline observed in children with severe OSA.

## Abstract (web summary)

Through single cell sequencing technology, this study reveals the stage-specific characteristics of adenoid tissues in children with obstructive sleep apnea (OSA), ranging from immune activation to functional exhaustion, and identifies the HIF1A signaling as a key mechanism and therapeutic target.

## Graphical abstract

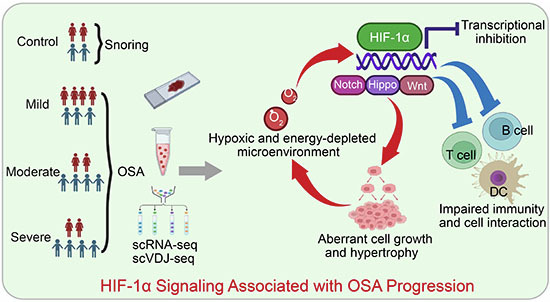

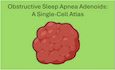

The paper explained **Problem**

Pediatric adenoids can undergo pathological hypertrophy influenced by complex factors, and severe cases may progress to OSA, exhibiting significant disease heterogeneity. Although lymphocytes within adenoids are inherently effector cells involved in immune enhancement, the specific cellular subsets and molecular mechanisms underlying their functional transformation or phenotypic changes under pathological conditions remain unclear. This knowledge gap limits the ability to scientifically weigh the potential immunological benefits of preserving adenoids against the necessity of their removal in clinical decision-making and hinders the development of targeted therapeutic strategies.

**Results**

We present a single-cell transcriptome and immune repertoire atlas of adenoid tissues from children with varying disease progression. Children with severe OSA exhibited abnormal growth and development signaling, cellular disorganization, along with downregulation of energy metabolism and ATP-related gene transcription. The transition from normal snoring to early OSA was accompanied by significant immune activation, inflammation, increased proliferation, and epithelial changes, whereas in the severe OSA stage, T-cell and B-cell immunity were impaired, antigen-processing capacity of innate immune cells was diminished, and cell-cell communication was reduced. TCR and BCR sequencing results supported the presence of stronger infection imprints, an active germinal center, and insufficient antibody maturation in the severe OSA group. Mechanistically, HIF1A-mediated hypoxia signaling may drive the downregulation of key immune molecules, making it a promising therapeutic target for severe OSA.

**Impact**

This study reveals that the transcriptional profile of adenoid tissues from children with varying degrees of OSA progression exhibits distinct stage characteristics, and proposes the HIF1A-mediated hypoxia signaling axis as a potential intervention target. These findings would provide a scientific basis for clinical decision-making on whether to remove or preserve dysfunctional adenoids in order to maximize therapeutic benefits.

## Introduction

The adenoids, located in the nasopharynx of children, serve as an important immune organ similar to the tonsils (Xu et al, 2024). Under normal circumstances, the adenoids gradually shrink as children grow up. However, when the adenoids undergo abnormal hyperplasia due to inflammation or other factors, they become enlarged, obstructing the respiratory tract and affecting children’s sleep breathing (Al-Iede et al, 2025; Xu et al, 2024). Adenoid hypertrophy is common in preschool and school-aged children and is one of the main causes of pediatric obstructive sleep apnea (OSA) (Al-Iede et al, 2025; Fitzgerald et al, 2025). Pediatric OSA is a severe sleep-related breathing disorder, with symptoms including nighttime snoring, mouth breathing, and apnea (Al-Iede et al, 2025; Fitzgerald et al, 2025). Long-term OSA not only affects children’s sleep quality, leading to daytime sleepiness and poor concentration, but may also trigger a series of serious complications, such as decreased immunity, lack of energy, delayed growth and development, learning disabilities, cognitive decline, and even affect craniofacial development, resulting in the formation of an “adenoidal face” (Al-Iede et al, 2025; Fitzgerald et al, 2025).

Children with adenoid hypertrophy also exhibit heterogeneity, with some only exhibiting normal snoring, while others may progress to OSA (Yang et al, 2024). Currently, the process of transition from normal snoring to early OSA in the adenoids remains unknown, and the differences in cellular composition and molecular mechanisms of adenoids with varying degrees of OSA severity have not been elucidated. Single-cell transcriptomic sequencing (scRNA-seq) technology enables comprehensive analysis of gene expression at the single-cell level (Li et al, 2024; Sun et al, 2025). It breaks through the limitations of traditional bulk sequencing, which treats tissue or cell populations as a homogeneous whole, and can effectively reveal inter-cell heterogeneity (Sun et al, 2025). Previous studies have provided single cell atlas of adenoids, almost composed of B cells and T cells (Cortese et al, 2023), but there is a lack of comparison between adenoids with different severities of OSA. The single-cell profile of PBMCs from children with OSA provides some disease-dependent cell types and molecular markers, but is insufficient to characterize changes in the microenvironment of adenoids (Yu et al, 2025). In addition, due to the unknown T-cell receptor (TCR) and B-cell receptor (BCR) information in adenoid tissue, the precise cell clones driving adenoid hypertrophy and the emergence of OSA remain unknown.

Here, we have integrated scRNA-seq and single-cell TCR and BCR sequencing (scTCR-seq/scBCR-seq) to characterize adenoid tissues across different stages of disease progression in children. This provides a detailed single-cell atlas of abnormal adenoid hypertrophy and explores profound molecular atlas changes during the early onset and later progression of OSA.

## Results

### Clinical monitoring and single cell atlas of adenoid hypertrophy with different conditions

**Figure 1:**
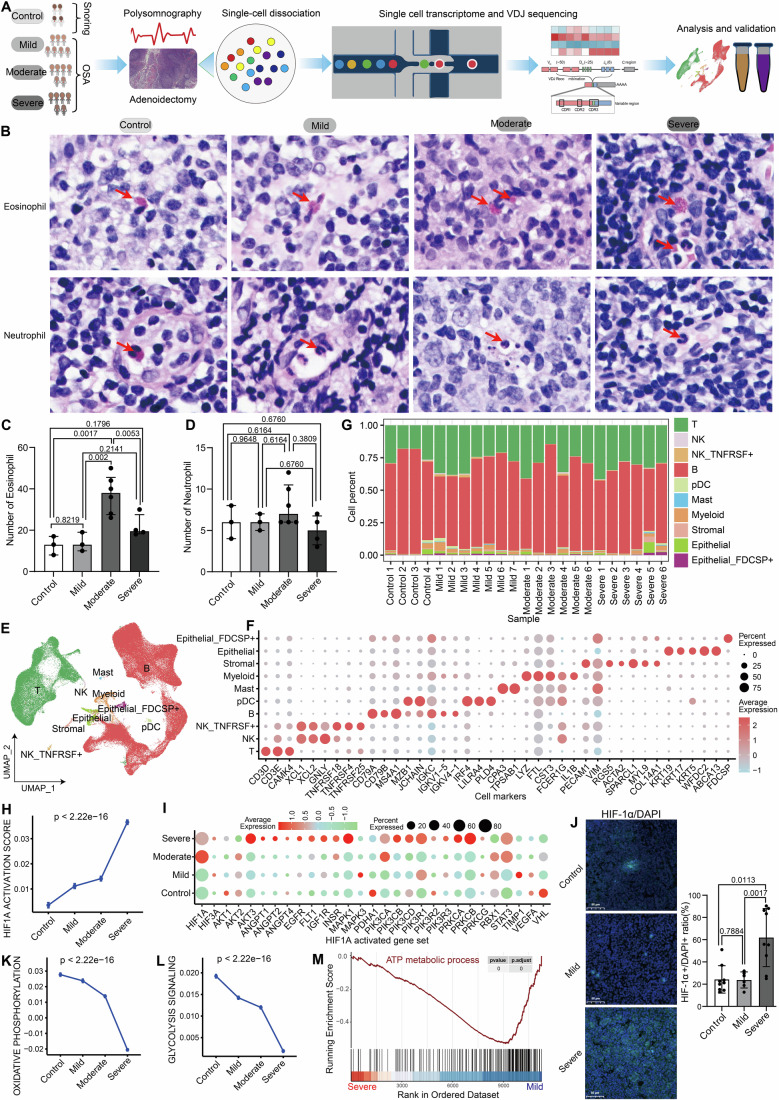
Pipeline and characteristics of main cell populations in the single-cell atlas of hypertrophic adenoids. (**A**) Single-cell sequencing design and bioinformatics analysis pipeline for adenoids at different disease stages (normal snoring, mild, moderate, and severe OSA). (**B**) HE staining results showing the numbers of eosinophils and neutrophils in adenoids at different disease stages. The red arrows in the first row indicate areas of eosinophil infiltration, while those in the second row indicate areas of neutrophil infiltration. (**C**,** D**) Statistical differences in eosinophils and neutrophils in adenoids at different disease stages. HE data was showed as mean ± SEM, control = 3 samples, mild = 3 samples, moderate = 6 samples, severe = 3 samples. Statistical testing using one-way analysis of variance. (**E**) Identification of major single-cell populations in adenoids at different disease stages. (**F**) Expression levels of marker genes corresponding to major cell types in adenoids, used to demonstrate the accuracy and specificity of cell type identification. (**G**) Proportions of adenoid cell populations at different disease stages (normal snoring, mild, moderate, and severe OSA). (**H**) Activity scores of hypoxia-activated gene sets in adenoids at different disease stages. Gene set scoring only represents the overall activity of the transcriptional expression levels of these functional genes. Multiple Kruskal–Wallis tests were used to assess the overall significance of gene set scoring across four groups. The error bars represent the median absolute deviation (MAD) of gene set activity. (**I**) Expression levels of hypoxia activation-related genes in adenoids at different disease stages. (**J**) Immunofluorescence showed the protein expression levels of HIF-1α in adenoid tissues of the control group and mild and severe OSA groups. Representative immunofluorescence images were shown, DAPI (blue), HIF-1α (cyan), 20×, scale bar: 50 μm. Samples were collected from the control group (*n* = 3), the mild group (*n* = 2), and the severe group (*n* = 3). For each sample within each group, three visual fields of equal area were randomly selected for quantification, and showed as mean ± SEM. Statistical testing using one-way analysis of variance. (**K**,** L**) Activity scores of energy metabolism and glucose metabolism-related gene sets in adenoids at different disease stages, including oxidative phosphorylation and glucose metabolism gene sets. Multiple Kruskal–Wallis tests were used to assess the overall significance of gene set scoring across four groups. The error bars represent the median absolute deviation (MAD) of gene set activity. (**M**) GSEA analysis showing the enrichment differences of ATP energy metabolism in adenoid cells between mild and severe OSA. The GSEA algorithm uses a permutation test to calculate statistical significance, while using the Benjamini–Hochberg method for multiple hypothesis testing adjustment. Source data are available online for this figure.

To investigate the underlying cellular composition and molecular differences behind adenoidal hypertrophy and OSA of varying severities, we designed the following pipeline study (Fig. 1A). Children were categorized into normal snoring symptoms and mild, moderate, and severe OSA patients based on polysomnography results, followed by clinical indicator assessments and single-cell sequencing. There were 11 (57.9%) males and 8 (42.1%) females in the OSA group, with ages ranging from 3.8 to 11.3 years. In the OSA group, 12 cases (63.2%) exhibited adenoid obstruction exceeding 90%, while 7 cases (36.8%) demonstrated obstruction exceeding 75% (Table EV1). In the control group, adenoid obstruction was recorded at 65% in two cases and at 90% in another two cases. No statistically significant differences were observed in age, gender, or BMI between the OSA groups stratified by severity and the control group (*p* > 0.05, Table EV1). There was also no significant difference in sleep structure characteristics (such as total sleep time, sleep efficiency and rapid eye movement) between the OSA group and control group (Table EV2). A further comparison of polysomnography indicators among the different severity OSA groups and the control group demonstrated that as OSA severity increased, the oxygen desaturation index (ODI), obstructive apnea-hypopnea index (OAHI), and apnea-hypopnea index (AHI) during sleep also significantly increased, which was consistent with the progression of disease severity (*p* < 0.05, Table 1). The hematoxylin-eosin (HE) staining demonstrated that eosinophil counts showed an increasing trend from mild to moderate OSA (*p* < 0.05) (Fig. 1B,C); however, notably, a decrease was observed in the severe OSA group compared to the moderate group (*p* < 0.05) (Fig. 1C). In contrast, no significant differences in neutrophil counts were found among the groups (Fig. 1D).

**Table 1:** Comparison of sleep-related respiratory events and blood oxygen saturation characteristics across sleep stages in OSA groups of varying severity and the control group.

	Control (*n* = 4)	Mild OSA (*n* = 7)	Moderate OSA (*n* = 6)	Severe OSA (*n* = 6)	*P* Value
**Respiratory events (events/h)**					
HI (x ± s)	0.33 ± 0.25	2.54 ± 0.95	5.02 ± 1.88	10.53 ± 6.70^a,b^	0.0012
OAI [M(P25, P75)]	0.000(0.000, 0.075)	0.000(0.000, 0.000)	0.000(0.000, 1.075)	4.150(1.200, 7.750)	0.1821
CAI (x ± s)	0.68 ± 0.57	0.70 ± 0.15	0.53 ± 0.42	0.50 ± 0.41	0.8167
OAHI (x ± s)	0.40 ± 0.17	2.51 ± 1.16	6.06 ± 2.36^a^	15.39 ± 5.06^a,b,c^	<0.0001
AHI (x ± s)	1.08 ± 0.43	3.21 ± 1.50	6.58 ± 2.19	15.85 ± 5.25^a,b,c^	<0.0001
ODI (x ± s)	2.70 ± 1.37	3.83 ± 2.19	9.25 ± 4.73	22.98 ± 5.57^a,b,c^	<0.0001
NREM ODI (x ± s)	7.65 ± 2.43	9.71 ± 6.56	25.58 ± 24.98	52.25 ± 8.06^a,b,c^	0.0001
REM ODI (x ± s)	1.18 ± 0.91	1.97 ± 1.02	7.25 ± 3.29	16.45 ± 8.51^a,b,c^	<0.0001
**SpO2 across sleep stages (%)**					
Wake SpO2 [M (P25, P75)]	97.00(97.00, 97.75)	97.00(97.00, 98.00)	97.00(97.00, 77.25)	96.50(96.00, 98.00)	0.8438
NREM Sleep SpO2 (x ± s)	96.25 ± 0.96	96.43 ± 0.98	96.33 ± 1.03	94.50 ± 1.76	0.0423
REM Sleep SpO2 [M (P25, P75)]	96.50(96.00, 97.00)	97.00(96.00, 97.00)	96.00(96.00, 96.50)	96.50(95.00, 97.00)	0.6386
Minimum SpO2 (x ± s)	87.75 ± 4.27	87.57 ± 6.66	87.67 ± 3.99	80.67 ± 6.68	0.1217

Numerical variables are shown as mean ± standard deviation or median (1st quarter-3rd quarter). Groups were compared using one-way ANOVA, followed by Tukey’s post hoc test (HSD) for multiple comparisons. *P* value <0.05 was considered statistically significant.

*HI* hypopnea index, *OAI* obstructive apnea index, *CAI* central apnea index, *OAHI* obstructive apnea-hypopnea index, *AHI* apnea-hypopnea index, *ODI* oxygen desaturation index, *NREM* non-rapid eye movement, *SpO*_2_ saturation of peripheral oxygen.

^a^*p* < 0.05 versus the Control group.

^b^*p* < 0.05 versus the mild OSA group.

^c^*p* < 0.05 versus the moderate OSA group.

**Figure EV1:**
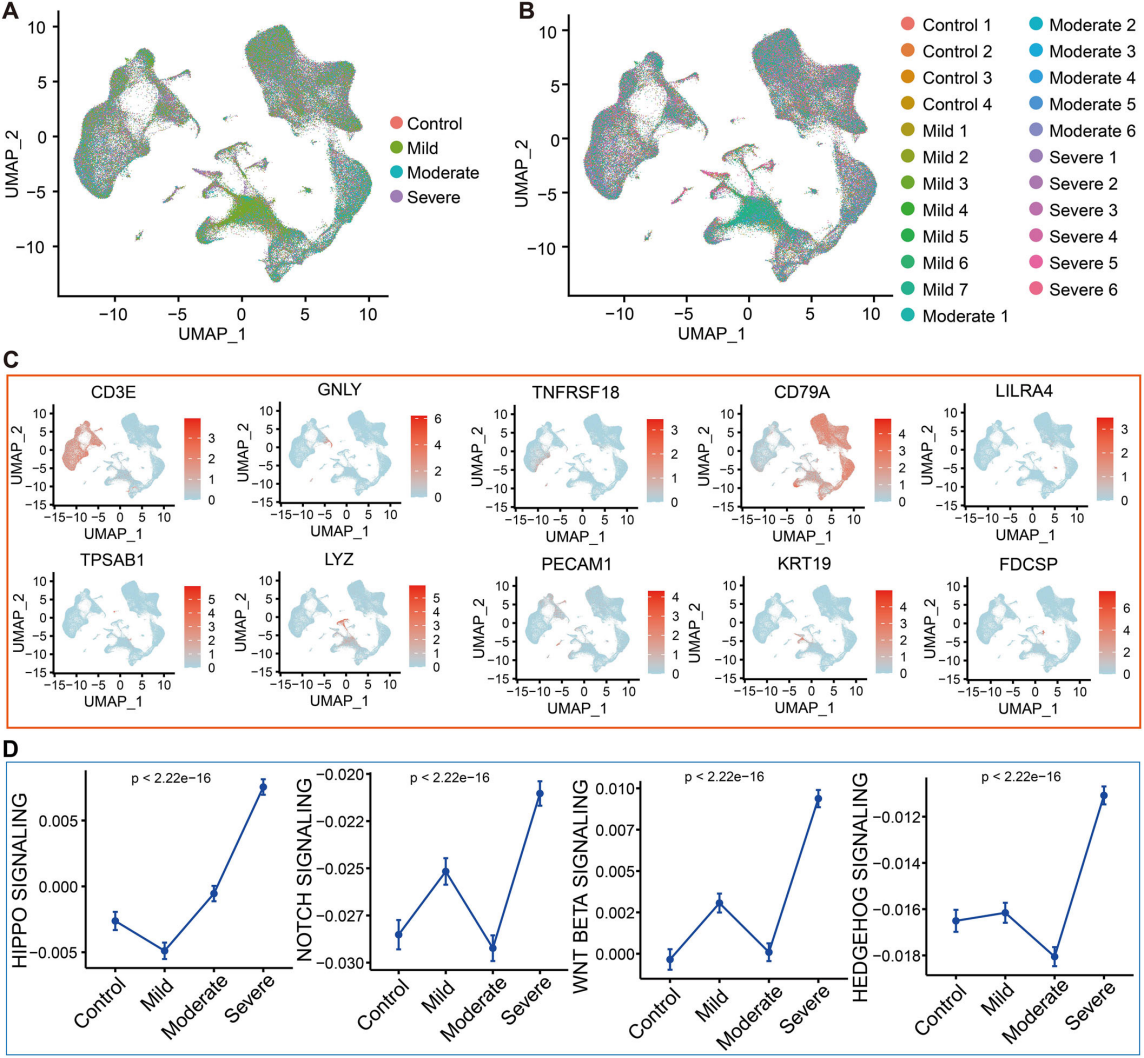
Identification of the main cell population of adenoid tissue and changes in the gene set related to growth and development. (**A**,** B**) UMAP plots show whether there are batch effects among major cell populations of hypertrophic adenoids between different groups and different samples. A uniform distribution indicates that batch effects between groups and samples have been removed. (**C**) The UMAP plot shows the expression levels of marker genes corresponding to major cell populations of hypertrophic adenoids. (**D**) Activity scores of growth and development-related gene sets in adenoids at different disease stages, including Hippo, Notch, Wnt, and Hedgehog gene sets. Gene set scoring only represents the overall activity of the transcriptional expression levels of these functional genes. Multiple Kruskal–Wallis tests were used to assess the overall significance of gene set scoring across four groups. The above error bars represent the median absolute deviation (MAD) of gene set activity.

These pediatric patients underwent surgical removal of hypertrophic adenoids, and 23 post-resection tissue samples were used for single-cell transcriptome and immune repertoire sequencing. After integration and removal of batch effects, we obtained 183,219 single cells, mainly including B cells (*CD79A*^+^), T cells (*CD3E*^+^), NK cells (*GNLY*^+^), myeloid cells (*LYZ*^+^), and stromal cells (*PECAM1*^+^) (Figs. 1E,F and EV1A–C). Among them, B cells accounted for ~75–80%, and T cells accounted for ~10–20%, constituting almost the entirety of the secondary lymphoid tissue adenoids (Fig. 1G). In addition, we also identified a subset of conventional epithelial cells (*KRT19*^+^/*KRT17*^+^) and epithelial cells with high expression of *FDCSP*, the latter of which has been reported in the tonsillar single-cell atlas. (Fig. 1E,F) (Massoni-Badosa et al, 2024). Interestingly, these epithelial cells showed enrichment in the severe group (Fig. 1G), and studies have found that proliferation of adenoid epithelial cells can lead to hypertrophy and OSA, although the proportion is lower (Yang et al, 2024; Ramirez et al, 2024). Additionally, mast cells, NK cells, and others were also found to be increased to some extent in the mild OSA group (Fig. 1G). However, these results did not show enrichment in the moderate and severe groups, indicating significant heterogeneity between normal snoring and OSA of varying severities.

A typical feature of OSA is adenoid hypertrophy; our analysis revealed that moderate and severe OSA groups exhibited higher Hippo, Notch, Wnt and Hedgehog signaling scores at the mRNA level (Fig. EV1D), indicating their abnormally active growth and development status. Interestingly, the moderate group is not a stable linear abnormal proliferation change in the progression of OSA disease (Fig. EV1D). In addition, adenoid hypertrophy blocking the airway often leads to poor breathing and hypoxia (Liu et al, 2025). Our results indicate that the hypoxia-related gene set, is significantly upregulated in the severe group (Fig. 1H), such as *HIF1A*, *AKT3*, *MAPK1*, *STAT3*, *PIK3*, and *PRKC* families (Fig. 1I). Immunofluorescence experiments also demonstrated that the key regulator HIF-1α protein level in severe OSA adenoid tissue was highest compared to the control and mild OSA groups (Fig. 1J). Furthermore, we also found that energy metabolism at the transcriptional level, including oxidative phosphorylation, glucose metabolism and ATP metabolism, are significantly downregulated in the severe group (Fig. 1K–M) which is consistent with the clinical manifestation of energy deficiency in children with severe OSA.

### Functional modules and cytokines associated with the progression of OSA at the mRNA level

**Figure 2:**
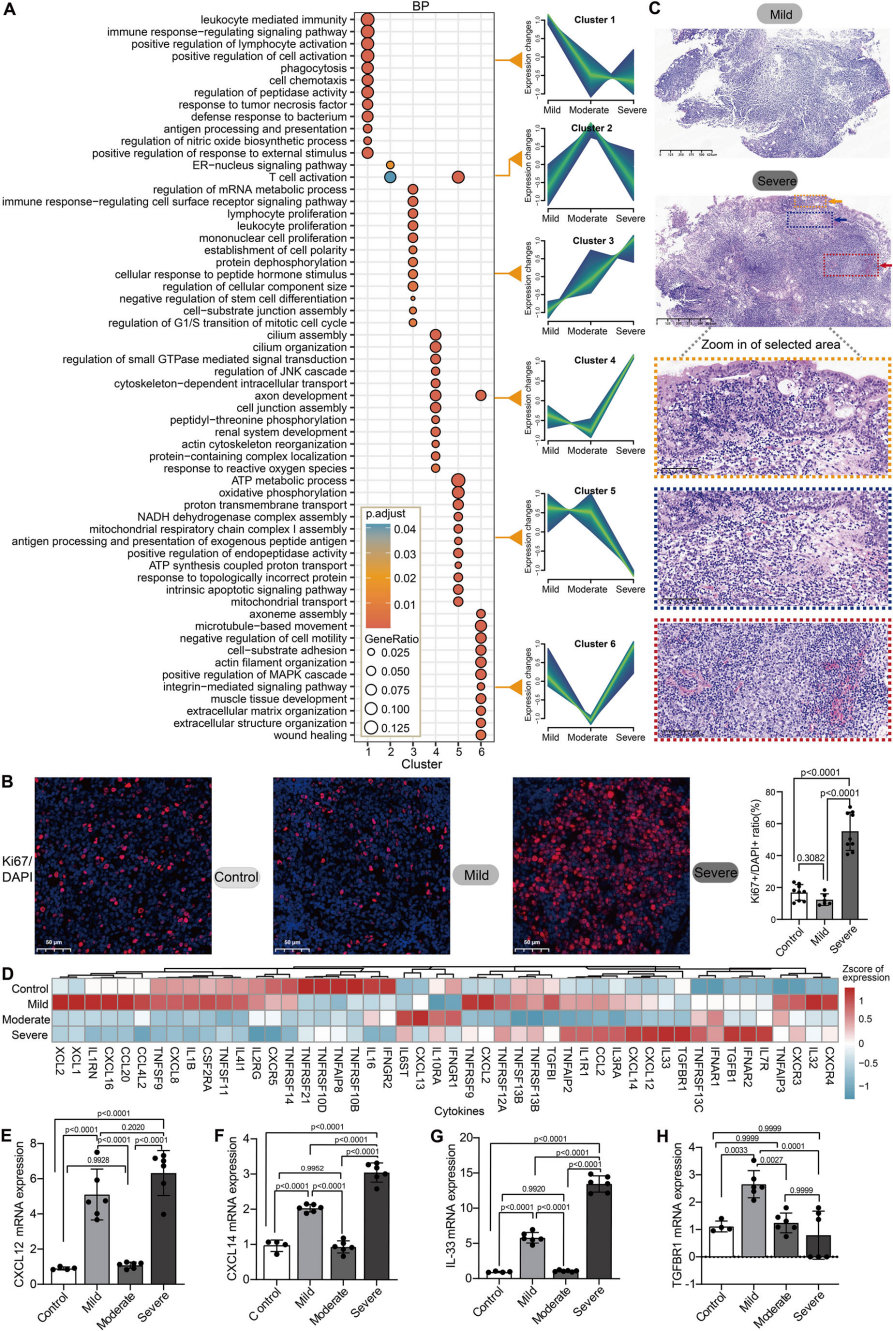
Functional modules and cytokine profiles along OSA disease progressions. (**A**) Clustering and functional enrichment analysis of expression profiles of adenoids with different severities of OSA based on a fuzzy clustering algorithm. Six clusters of dynamically differentially expressed genes and functional modules were identified along the progression of OSA. (**B**) Immunofluorescence showed the protein expression levels of Ki67 in adenoid tissues of the control, mild and severe OSA groups. Representative immunofluorescence images were shown, DAPI (blue), HIF-1α (red), 20×, scale bar: 50 μm. Samples were collected from the control group (*n* = 3), the mild group (*n* = 2), and the severe group (*n* = 3). For each sample within each group, three visual fields of equal area were randomly selected for quantification, and showed as mean ± SEM. Statistical testing using one-way analysis of variance. (**C**) Histopathological features of adenoid tissues with varying degrees of OSA. Low-magnification (4×) views demonstrating mild and moderate/severe OSA in the upper part, and high-magnification (20×) views of areas outlined. Different colored arrows and dashed boxes together indicate the key pathological areas in the moderate/severe group. yellow zoom box: epithelial hyperplasia with neutrophilic infiltration and focal cystic change; blue zoom box: subepithelial edema infiltrated by lymphocytes and plasma cells; red zoom box: hyperemia and hemorrhage within the adenoid parenchyma. (**D**) Differentially expressed cytokines in adenoid cells at different disease stages (normal snoring, mild, moderate, and severe OSA). Cytokines that were differentially expressed in at least one major cell type were included in the visualization. (**E**–**H**) Q-PCR verification of the upregulation of cytokines (*CXCL12*, *CXCL14*, *IL33*, and *TGFBR1*) in severe OSA. Q-PCR data were shown as mean ± SEM, control = 4 samples, mild = 6 samples, moderate = 6 samples, severe = 6 samples. Statistical testing using one-way analysis of variance. Source data are available online for this figure.

To identify key gene modules with continuous changes in OSA severity, we employed the mfuzz algorithm to perform fuzzy clustering on the pseudo-bulk expression data of mild, moderate, and severe OSA groups. The results revealed six gene modules with distinct expression trends along the progression of OSA disease (Fig. 2A; Dataset EV1). Cluster 1 initially declined sharply as OSA worsened, then stabilized in the severe group, representing the early rapid progression stage of the disease. It is primarily involved in immune processes such as lymphocyte activation, phagocytosis, and cell chemotaxis, indicating a decrease in immune activation during disease progression (Fig. 2A; Appendix Fig. S1; Dataset EV2). Cluster 2 initially increased and then decreased, enriched only in the rare and significant ER-nucleus signaling pathway, suggesting significant endoplasmic reticulum functional fluctuations in moderate OSA (Fig. 2A; Appendix Fig. S1; Dataset EV2). Cluster 3 continuously increased along the progression of OSA disease, representing a set of characteristic genes associated with disease exacerbation. It is primarily enriched in lymphocyte proliferation, leukocyte proliferation, establishment of cell polarity, and mRNA metabolic processes, indicating that cell proliferation status intensifies with disease progression (Fig. 2A; Appendix Fig. S1A; Dataset EV2). Cluster 4 remained largely unchanged during the early stages of disease progression but suddenly increased during the progression to severe stages. Its representative functions include ciliary movement and organization, JNK cascade, actin cytoskeleton, and cell junction assembly (Fig. 2A; Appendix Fig. S1A; Dataset EV2). Cluster 5 showed minimal changes in the early stages of disease, but decreased in the severe group. Its representative enriched functions mainly include ATP synthesis, mitochondrial metabolism, and NADH energy supply (Fig. 2A; Appendix Fig. S1A; Dataset EV2). Cluster 6 initially decreased and then increased along the progression of disease, primarily enriched in microtubule bundle formation and ciliary-related functions. Overall, analysis based on gene modules indicates that the characteristics of severe OSA mainly include cell proliferation, cellular morphological disorder, and increased epithelial cell morphology, as well as decreased energy metabolism and immune activation (Fig. 2A). Immunofluorescence experiments showed that there was indeed a significant upregulation of Ki67 protein levels in the severe OSA group indicating an active proliferative state (Fig. 2B). Furthermore, HE-stained pathological sections revealed more pronounced structural disorganization in the severe OSA group, characterized by irregular lymphoid follicle morphology with blurred boundaries, loosely arranged surface mucosal epithelium, increased number of small blood vessels with dilated lumina, as well as stromal loosening and widened intercellular spaces (Fig. 2C).

Next, we analyzed the number of differentially expressed genes (DEGs) in the main cell populations (Dataset EV3). The results indicated that B cells, T cells, and epithelial cells exhibited significant expression changes, accompanied by the highest number of DEGs, suggesting that they may be major contributors to disease progression (Appendix Fig. S1B,C). We paid special attention to and discovered distinct cytokine expression patterns in adenoids across different disease groups. Specifically, the severe OSA group exhibited high expression of cytokines such as *CXCL14*, *CXCL12*, *TGFBR1*, *IL33*, *FNAR2*, and *TGFB1* (Fig. 2D). The results from qPCR validation also confirmed that *CXCL14*, C*XCL12*, and *IL33* were indeed upregulated to the highest extent in the severe OSA group (Fig. 2E–H), indicating their potential as clinical markers for severe OSA. Interestingly, the normal snoring group shared multiple cytokines with the mild OSA group, but notably exhibited specific high expression of *TNFRSF21*, *TNFRSF10D*, *TNFAIP8*, *TNFRSF10B*, *IL16*, and *IFNGR2* (Fig. 2D). These specific cytokines may be related to the transition from normal snoring to early OSA, and their causal relationship deserves further investigation. Additionally, the cytokine expression profile indicated a skipped expression pattern in the moderate OSA group compared to the mild and severe groups, suggesting a nonlinear change (Fig. 2B).

### Children with severe OSA exhibit diminished B-cell immunity and immunoglobulin at the mRNA level

**Figure 3:**
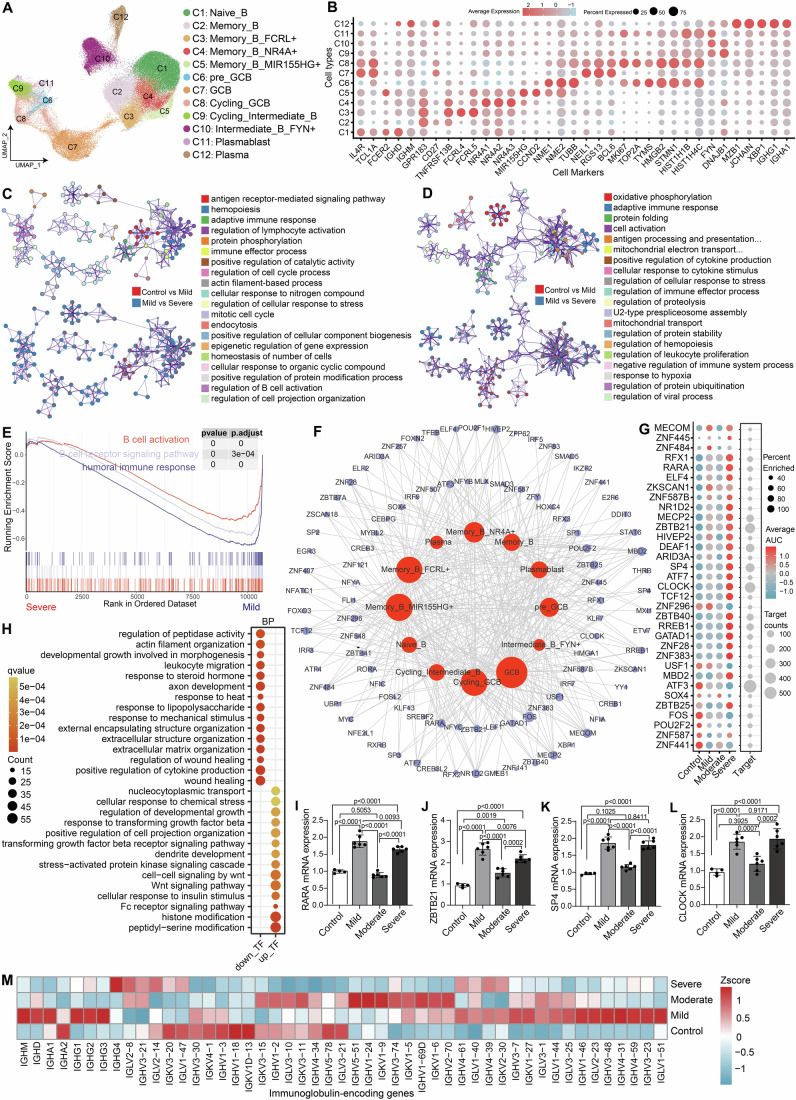
Identification of B cell subsets and related cellular composition and molecular changes. (**A**) Identification of B cell subsets in adenoids at different disease stages. (**B**) Expression levels of marker genes corresponding to B cell subsets in adenoids at different disease stages, used to demonstrate the accuracy and specificity of cell type identification. (**C**,** D**) The network diagram displays the functional modules enriched by the top shared differentially expressed genes that are upregulated (**C**) or downregulated (**D**) between B-cell subsets in the control versus the mild OSA or the mild OSA versus the severe OSA. The upper part of the network represents the enriched biological processes or pathways and their interrelationships. The lower part of the network illustrates the overlap of functional networks enriched from the two sets of differentially expressed genes (red: control vs. mild OSA; blue: mild OSA vs. severe OSA). (**E**) GSEA enrichment results of B cell-related functions between the mild and severe OSA groups, including B cell activation, BCR signaling pathway, and humoral immunity. (**F**) Transcription factor regulatory networks with differential enrichment (AUC) appearing at least once in B cell subsets of adenoids at different disease stages. Red circles represent B cell subsets, with larger sizes indicating more transcription factors with differences in the cells. Blue circles represent transcription factors, with larger sizes indicating that the transcription factors are dysregulated in more cell types. (**G**) Dotplot showing key transcription factors with gradient changes along the OSA disease course. (**H**) Functional enrichment analysis of transcription factors with upregulated or downregulated enrichment in the severe OSA group and their target genes. (**I**–**L**) Q-PCR verification of transcription factors with upregulated enrichment in the severe OSA group, including *RARA*, *SP4*, *ZBTB21*, and *CLOCK*. Q-PCR data were shown as mean ± SEM, control = 4 samples, mild = 6 samples, moderate = 6 samples, severe = 6 samples. Statistical testing using one-way analysis of variance. (**M**) Heatmap showing differential immunoglobulin-encoding genes and immunoglobulin variable region-encoding genes in adenoid B cells at different disease stages (normal snoring, mild, moderate, and severe OSA). Source data are available online for this figure.

**Figure EV2:**
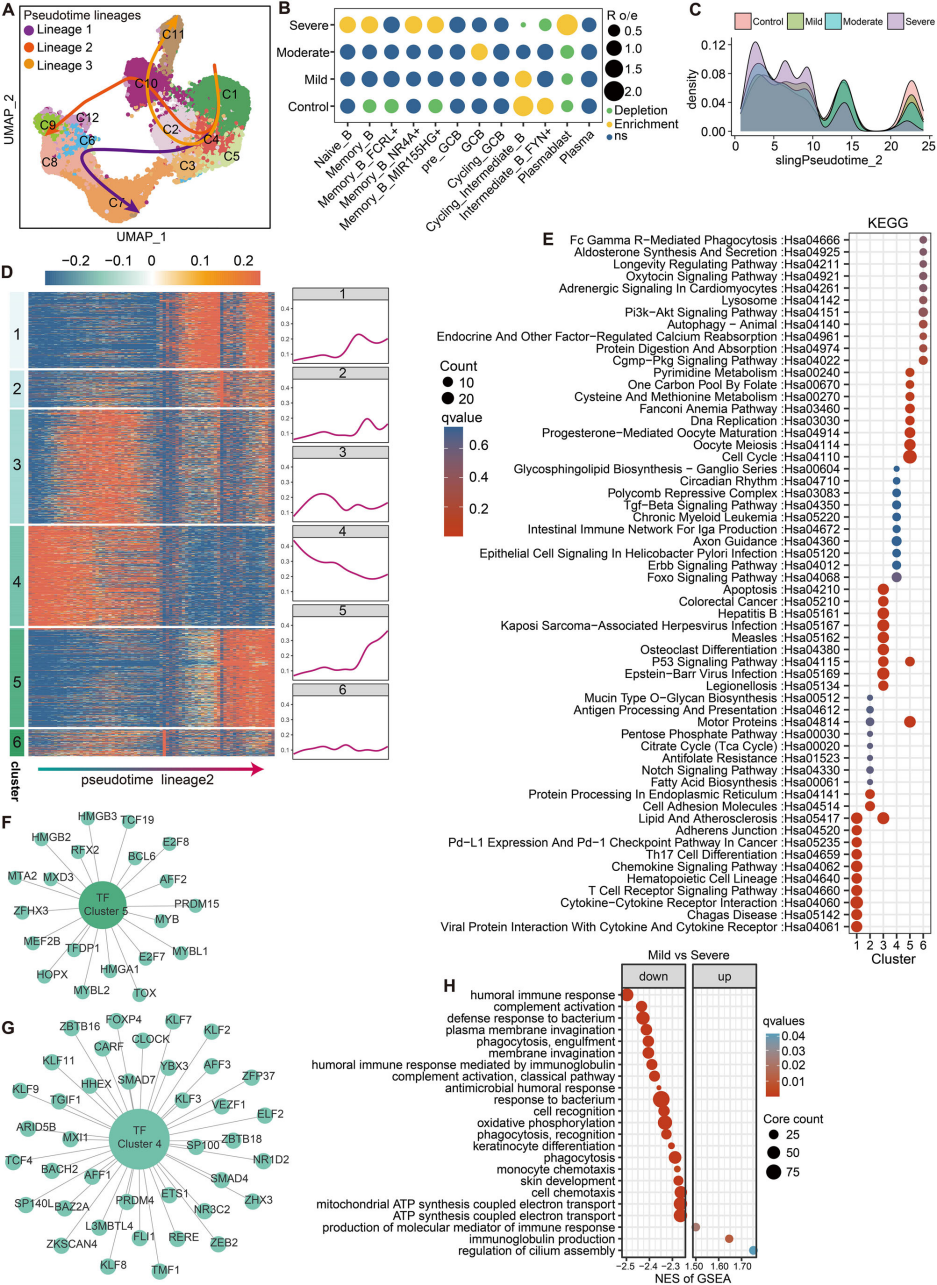
Identification of B cell subsets and results of branch trajectory analysis. (**A**) UMAP plot shows three B-cell differentiation trajectories identified based on pseudotime analysis. (**B**) Enrichment of the abundance of different B cell subsets in adenoid groups with different disease courses. (**C**) The density plot shows the proportion of cells along the second differentiation trajectory (intermediate B cells) in different disease course groups, and it is found that the severe OSA group is significantly reduced at the end of differentiation. (**D**) The heatmap shows the clustering and expression changes of dynamically differentially expressed genes (DDEGs) during the second differentiation trajectory (intermediate B cells). (**E**) KEGG enrichment analysis of DDEGs during the second differentiation trajectory (intermediate B cells). (**F**) Transcription factors included in the gradient upregulated genes (Cluster 5) during the second differentiation trajectory (intermediate B cells). (**G**) Transcription factors included in the gradient downregulated genes (Cluster 4) during the second differentiation trajectory (intermediate B cells). (**H**) GSEA enrichment analysis of the total B cell expression profiles between the mild and severe OSA groups, used to show the biological processes that are significantly upregulated or downregulated between the groups.

As the main component of adenoids, we identified 12 B-cell subpopulations, including germinal center B (GCB) (*NEIL1*^+^/*RGS13*^+^), memory B (*CD27*^+^/*TNFRSF13B*^+^), plasma cells (*MZB1*^+^/*JCHAIN*^+^), intermediate B cells (*IGHD*^+^/*CD27*^+^), and naive B cells (*IL4R*^+^/*TCL1A*^+^) in different states (Fig. 3A,B; Appendix Fig. S2A). These B-cell subpopulations exhibit three differentiation trajectories: from naive B to GCB, from naive B to intermediate B, and from naive B to plasma cells (Fig. EV2A). Cell abundance analysis indicates that the intermediate B cells are decreased in the severe OSA group, while GCB shows an increasing trend in the moderate OSA group (Fig. EV2B). Pseudo-time analysis also reveals that the decline along the intermediate B cell differentiation trajectory is most pronounced in the severe OSA group (Fig. EV2C). Notably, intermediate B cells are considered to have versatile functions due to their potential to differentiate into memory B or plasma cells, and can also directly become terminally differentiated but low neutralizing cells in certain disease environments. Thus, the reduction of intermediate B cells may be associated with the progression of OSA. Furthermore, we analyzed dynamically differentially expressed genes (DDEGs) along the second trajectory (intermediate B cells) and identified six expression patterns (Fig. EV2D). At the end of cell differentiation, cluster 5 is significantly upregulated, primarily involved in DNA replication and cell cycle regulation (Fig. EV2E), which is consistent with its high expression of the proto-oncogene *FYN*, which encodes a membrane-associated tyrosine kinase associated with cell growth control. Further results also revealed that cluster 5 highly expressed multiple proto-oncogenic transcription factors controlling the cell cycle, such as *MYB*, *MYBL1*, *MYBL2*, *E2F8*, *E2F7*, and *BCL6* (Fig. EV2F) (Chen et al, 2024; Coppola et al, 2025; Fan et al, 2022; Sun et al, 2024), while downregulating multiple KLF family transcription factors (*KLF2*, *KLF7*, *KLF9*, etc.) (Fig. EV2G).

Next, we calculated the DEGs across different B-cell subsets, and found that GCB may have the greatest dysregulation, accompanied by more DEGs (Appendix Fig. S2B; Dataset EV4). Then, the functional analysis was conducted on genes that were shared and altered in at least two B-cell subsets (Fig. 3C,D). The results revealed that mild OSA mainly upregulated biological processes related to antigen presentation, lymphocyte activation, B-cell activation, and hemopoiesis compared to normal snoring (Fig. 3C; Dataset EV5). Subsequently, during the progression stage of the disease, severe OSA groups, compared to mild OSA groups, not only shared the aforementioned function modules but also upregulated processes such as cell projection organization, actin filament-based processes, and cell cycle (Fig. 3C; Dataset EV5). Conversely, downregulated DEG functions indicated that the transition from normal snoring to OSA and the progression of OSA shared essential energy-providing processes such as oxidative phosphorylation, protein folding, and mitochondrial transport (Fig. 3D; Dataset EV5). The severe OSA group specifically downregulated the U2-type prespliceosome assembly functional module (Fig. 3D; Dataset EV5). Furthermore, based on GSEA analysis, we found that the severe OSA group significantly downregulated complement activation, B-cell activation, humoral immunity, as well as ATP and mitochondrial metabolism compared to the mild OSA group at the mRNA level (Figs. 3E and EV2H; Dataset EV6). However, the OSA group maintained higher levels of B-cell immune activity compared to the control group (Appendix Fig. S2C). These results strongly suggest that the B cell immune function of children with severe OSA is impaired at the mRNA level, and their intracellular ATP production and energy balance may be disrupted.

We further analyzed the transcription factors differentially enriched in different B-cell subpopulations, which may drive DEGs (Fig. 3F). The results indicated that GCB and memory B cells exhibited multiple dysregulated transcription factors, suggesting significant dysregulated expression during the occurrence and progression of OSA (Fig. 3F). We further identified transcription factors that were gradually downregulated in B cells along with the occurrence and progression of OSA, such as *ATF3*, *FOS*, *ZNF587*, *ZNF441*, and *USF1* (Fig. 3G). The target genes regulated by these transcription factors were primarily enriched in actin filament organization, wound healing, and extracellular matrix (Fig. 3H). Conversely, the severe OSA group upregulated more transcription factors, including *RARA*, *SP4*, *CLOCK*, *ZBTB21*, *TCF12*, and *RFX1* (Fig. 3G), and their target genes were mainly enriched in histone modification, peptidylserine modification, Fc receptor signaling pathway, Wnt signaling, growth and development, and dendrite development (Fig. 3H). We also conducted qPCR validation on some of the upregulated transcription factors in the OSA group. The results showed that *RARA*, *SP4*, *CLOCK*, *ZBTB21*, etc., were significantly upregulated in the OSA group compared to the control group (*p* < 0.05), with high expression in the severe OSA group, while the moderate group still exhibited an unstable intermediate state (Fig. 3I–L).

Finally, we compared the changes in immunoglobulin-encoding genes across different groups and found that *IGHD*, *IGHM*, *IGHA1*, *IGHA2*, *IGHG1*, *IGHG2*, and *IGHG3* were downregulated in the severe group (Fig. 3M). Additionally, the heavy chain V genes encoding antibodies exhibited distinct expression patterns. Compared to the mild OSA group, the severe OSA group showed significantly downregulated expression of *IGHV3*−*48*, *IGHV4*−*31*, *IGHV4*−*59*, and *IGHV3*−*23*, indicating that the V genes in the severe OSA group underwent specific transition or recombination (Fig. 3M). Interestingly, immunoglobulin-encoding genes in the normal snoring group were also downregulated like those in the severe group, except for *IGHA2*, suggesting that abnormally increased immunoglobulin expression may be associated with early OSA transition, but its protein levels and causal mechanisms remain to be explored (Fig. 3M). Furthermore, the normal snoring group exhibited distinctly different expression patterns of antibody variable (V) region-encoding genes compared to the early OSA group (Fig. 3M).

### Children with severe OSA exhibit weakened T-cell activation and heat shock protein disorder at the mRNA level

**Figure 4:**
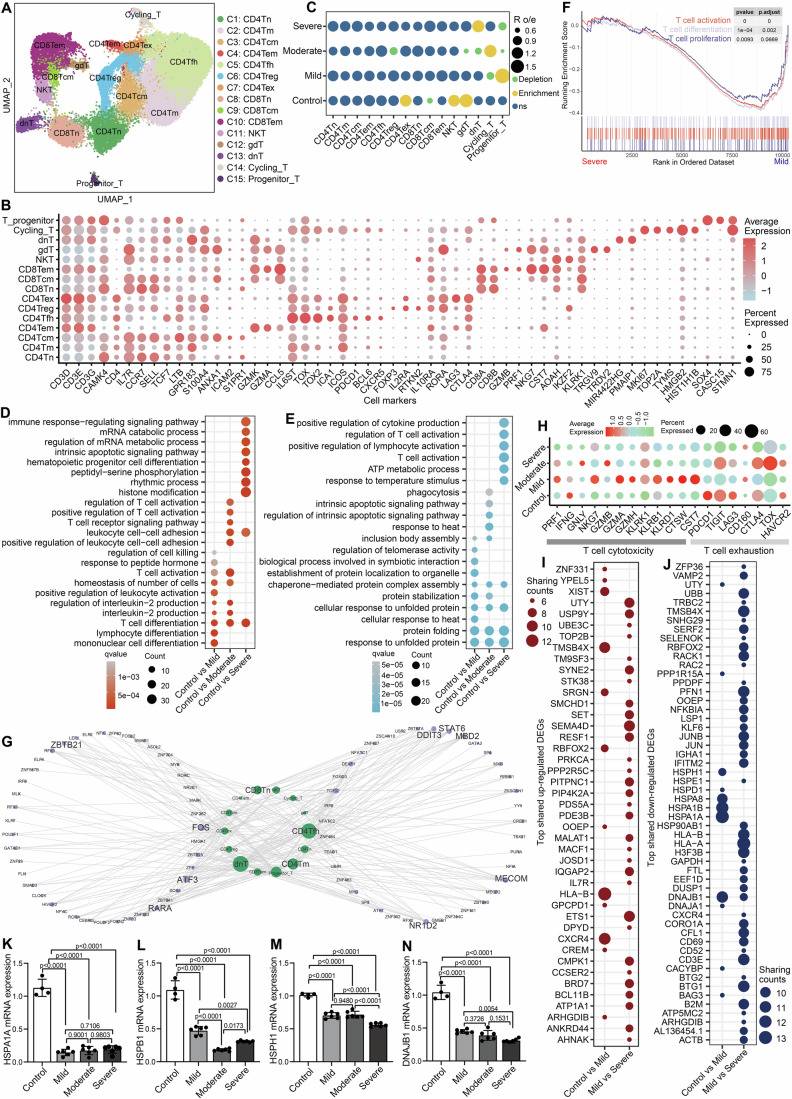
Identification of T cell subsets and related cellular composition and molecular changes. (**A**) Identification of T cell subsets in adenoids at different disease stages. (**B**) Expression levels of marker genes corresponding to T cell subsets in adenoids, used to demonstrate the accuracy and specificity of cell type identification. (**C**) Differences in cell abundance of T cell subsets in adenoids at different disease stages. (**D**,** E**) Functional enrichment analysis of genes that are commonly upregulated or downregulated in T cell subsets. Genes that are commonly upregulated or downregulated in at least 2T cell types between the OSA and control groups were used for functional enrichment analysis. (**F**) GSEA enrichment results of T cell-related functions between the mild and severe OSA groups, including T cell activation, T cell proliferation, and T cell differentiation. The GSEA algorithm uses the permutation test to calculate statistical significance, while using the Benjamini–Hochberg method for multiple hypothesis testing adjustment. (**G**) Transcription factor regulatory networks with differential enrichment (AUC) appearing at least once in T cell subsets of adenoids at different disease stages. The circles in the middle represent T cell subsets, with larger sizes indicating more transcription factors with differences in the cells. The blue circles on both sides represent transcription factors, with larger sizes indicating that the transcription factors are dysregulated in more cell types. (**H**) Expression changes of genes related to T cell cytotoxicity and T cell exhaustion in T cells of adenoids at different disease stages. (**I**,** J**) Dotplots showing genes that are commonly upregulated or downregulated in T cell subsets between the control group and the mild OSA group, as well as between the mild OSA group and the severe OSA group, with only the top frequently shared differential genes displayed. (**K**–**N**) Q-PCR verification of expression changes of heat shock protein families in adenoid cells at different disease stages. Q-PCR data were shown as mean ± SEM, control = 4 samples, mild = 6 samples, moderate = 6 samples, severe = 6 samples. Statistical testing using one-way analysis of variance. Source data are available online for this figure.

In the analysis of T cell subsets in children’s adenoids, 15 subsets were identified, including CD4T (*CD3D*^+^/*CD4*^+^) and CD8T (*CD3D*^+^/*CD8*^+^) cells in different states, double-negative T cells (dnT) (*CD3D*^+^/*CD4*^-^/*CD8*^-^), cycling T cells (*CD3D*^+^/*MKI67*^+^), NKT cells (*AOAH*^+^/*IKZF2*^+^), and γδT cells (*TRGV9*^+^/*TRDV2*^+^) and other non-classical T cells (Fig. 4A,B; Appendix Fig. S3A). Statistical analysis of cell proportions revealed that compared with the mild group, the severe OSA group had a significant enrichment of dnT cells and a reduction in cycling T cells. Compared with OSA, the normal snoring group had a higher enrichment of gdT cells, which may be related to the transformation from common adenoid hypertrophy to OSA (Fig. 4C).

**Figure EV3:**
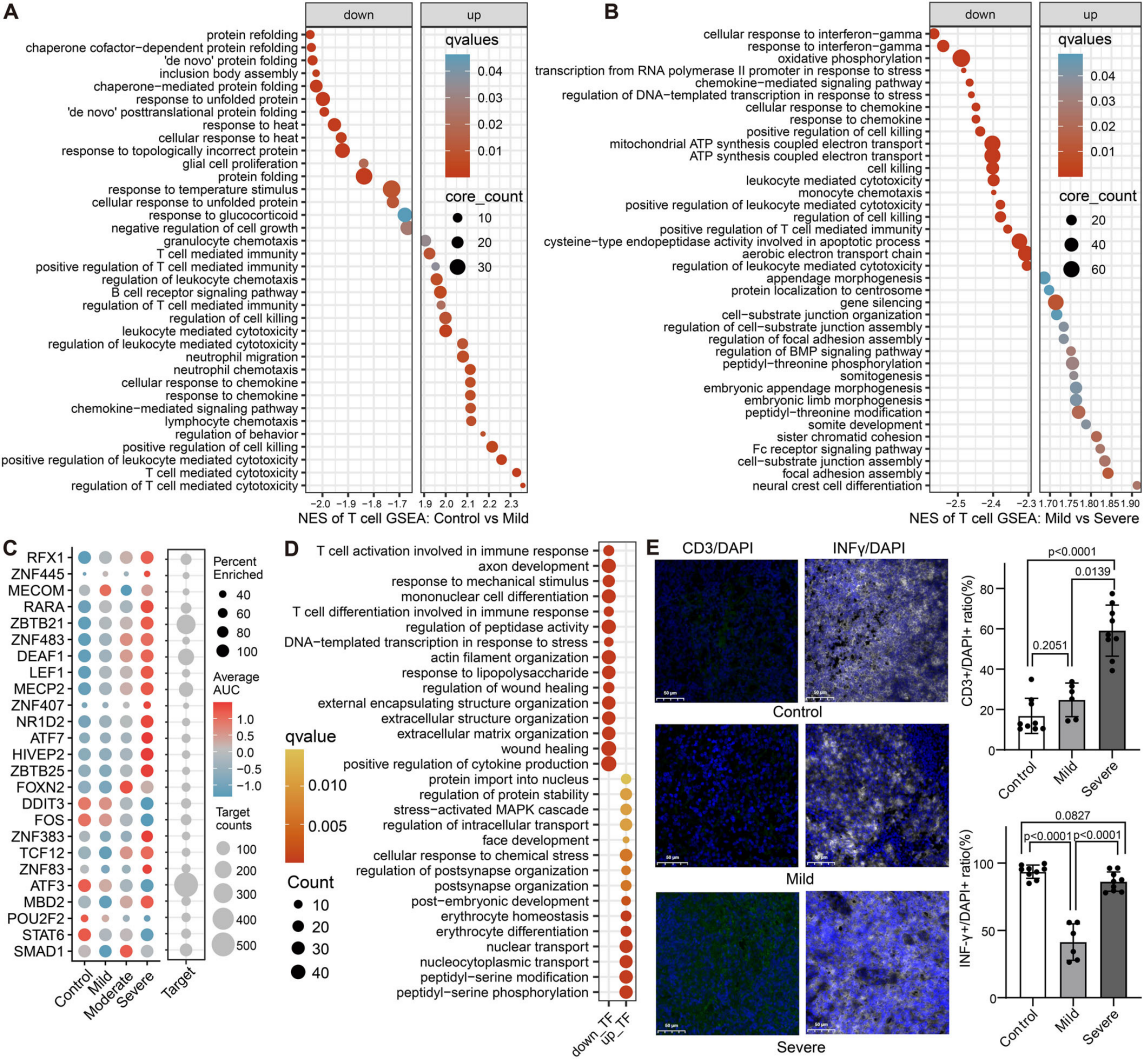
GSEA enrichment results and transcription factors associated with T-cell activity among different groups. (**A**) GSEA enrichment analysis results of the overall T cell expression profiles between the control and mild OSA groups. (**B**) GSEA enrichment analysis results of the overall T cell expression profiles between the mild and severe OSA groups. (**C**) Dotplot shows key transcription factors with gradient changes along the progression of different adenoid diseases. The color represents the AUC enrichment degree of transcription factors in different modules, the size of the circle represents the percentage of enriched cells, and the size of the gray circle corresponds to the number of target genes regulated by transcription factors. (**D**) Functional enrichment analysis of transcription factors and their target genes that are upregulated or downregulated in T cells of the severe OSA group. (**E**) Immunofluorescence showed the protein expression levels of CD3 and IFNγ in adenoid tissues of the control, mild and severe OSA groups. Representative immunofluorescence images were shown, DAPI (blue), CD3 (green), IFNγ (white), 20×, scale bar: 50 μm. Samples were collected from the control group (*n* = 3), the mild group (*n* = 2), and the severe group (*n* = 3). For each sample within each group, three visual fields of equal area were randomly selected for quantification, and showed as mean ± SEM. Statistical testing using one-way analysis of variance. Source data are available online for this figure.

Difference analysis indicated that CD4 Tfh, CD4Tm and CD8 Tem exhibited the most DEGs, especially the “control vs severe” and the “mild vs severe” (Appendix Fig. S3B; Dataset EV7). Functional analysis of the frequently shared upregulated DEGs revealed that compared with the control group, the mild OSA group significantly induced the T cell activation-related module, but it was downregulated in the severe group (Fig. 4D,E; Dataset EV8). Additionally, compared with the normal snoring group, the severe group upregulated histone modification and mRNA metabolism, but downregulated ATP metabolism and response to temperature (Fig. 4D,E). Moreover, the functional enrichment items of mild OSA and moderate OSA tended to be consistent, suggesting that their T cell functions were more similar and separated from the severe OSA group (Fig. 4D,E). Further, we used GSEA analysis to verify this, and the results showed that compared with the mild OSA and control group, T cell activation, proliferation, and differentiation in the severe OSA group were significantly downregulated (Figs. 4F and EV3A,B). This indicates that T cell immunity is weakened in the severe OSA group at the mRNA level, and occurs functional state transition at different stages of OSA.

Subsequently, we constructed a transcription factor network for T cell subsets, and the results demonstrated that T cells, including dnT, CD4 Tfh, CD4Tm, and CD8Tn, exhibited more prominent transcription factor dysregulation (Fig. 4G). Transcription factors such as *RARA*, *ATF3*, *NRID2*, and *STAT6* showed the highest frequency of dysregulation across multiple T cell subsets (Fig. 4G). Furthermore, *STAT6*, *ATF3*, and *POU2F2* were significantly downregulated in a gradient during the occurrence and aggravation of OSA (Fig. EV3C). In contrast, *TCF12*, *ZBTB25*, *ATF7*, *MECP2*, and *LEF1* were upregulated, with the severe group showing enrichment of more dysregulated transcription factors (Fig. EV3C). Functional analysis further indicated that the upregulated transcription factors and targets in the severe group are mainly involved in processes such as MAPK signaling, nuclear transport, peptidylserine phosphorylation, and stress response, while the downregulated ones are primarily enriched in T cell immunity, cytokine production, wound healing, and extracellular matrix organization (Fig. EV3D). This is basically consistent with the results of DEGs, that T cells in the severe OSA group downregulated T cell-related transcriptional regulatory modules. In order to validate T cell activity at the protein level, immunofluorescence experiments were performed and found that the severe OSA group indeed had higher levels of CD3 protein, combined with Ki67, indicating significant T lymphocyte proliferation (Fig. EV3E). However, IFNγ in the severe group was not significantly downregulated like the mRNA level (Fig. EV3E), indicating that there are different regulatory factors between mRNA levels and proteins.

We further evaluated the expression of T cell toxicity and exhaustion-related markers, and found that compared with the mild group, the severe OSA group downregulated toxicity markers, such as *PRF1*, *NKG7*, *GZMA*, *GZMH*, *CST7*, and *CSTW*, but the T cell exhaustion markers were almost the same as those of the mild group (Fig. 4H). Interestingly, the mRNA levels of T cell cytotoxicity-associated markers in the moderate OSA group were overall comparable to those in the control group. However, the moderate OSA group exhibited upregulation of multiple T cell exhaustion-related genes such as *TOX*, *TIGIT*, and *CTLA4* compared to the control group (Fig. 4H). In addition, compared with the normal snoring group, the mild OSA group also upregulated these T cell toxicity markers, indicating a potential shift from simple adenoid hypertrophy to early OSA (Fig. 4H). Interestingly, compared with the normal snoring group, the mild OSA group downregulated *PDCD1*, *TIGIT*, *LAG3* and *CTLA4*, etc. (Fig. 4H). These results indicate that adenoid T cells in children undergo significant functional changes from normal snoring to the exacerbation of OSA.

Finally, we visualized the top sharing DEGs. Mild OSA upregulated *CXCR4*, *HLA-B*, *TMSB4X*, *XIST*, etc. compared with the normal snoring group (Fig. 4I). Compared with mild OSA group, severe OSA group upregulated *BRD7*, *ETS1*, *PITPNC1*, *SEMA4D*, and *UTY*, while downregulated genes included *B2M*, *BTG*, *HLA-A*, *HLA-B*, *JUN*, *TMSB4X*, etc. (Fig. 4I,J). Interestingly, we found that the heat shock protein family (*HSPA1A*, *HSPA1B*, *DNAJB1*, also called *HSP40*, etc.) was highly consistently downregulated in the OSA group, and they were similar in the whole adenoid cells, not only T cells (Fig. 4J; Appendix Fig. S3C). We performed some qPCR to confirm that *HSPA1A*, *HSPB1*, *HSPH1*, and *DNAJB1* were indeed downregulated in the OSA group compared with normal snoring children (Fig. 4K–N). These results suggest that the early occurrence of OSA may experience the dysregulation of HSP, which may have an impact on protein folding and stress protection in children (He and Wang, 2022).

### Innate immune cells in children with severe OSA exhibit attenuated antigen processing and cell communication at the mRNA level

**Figure 5:**
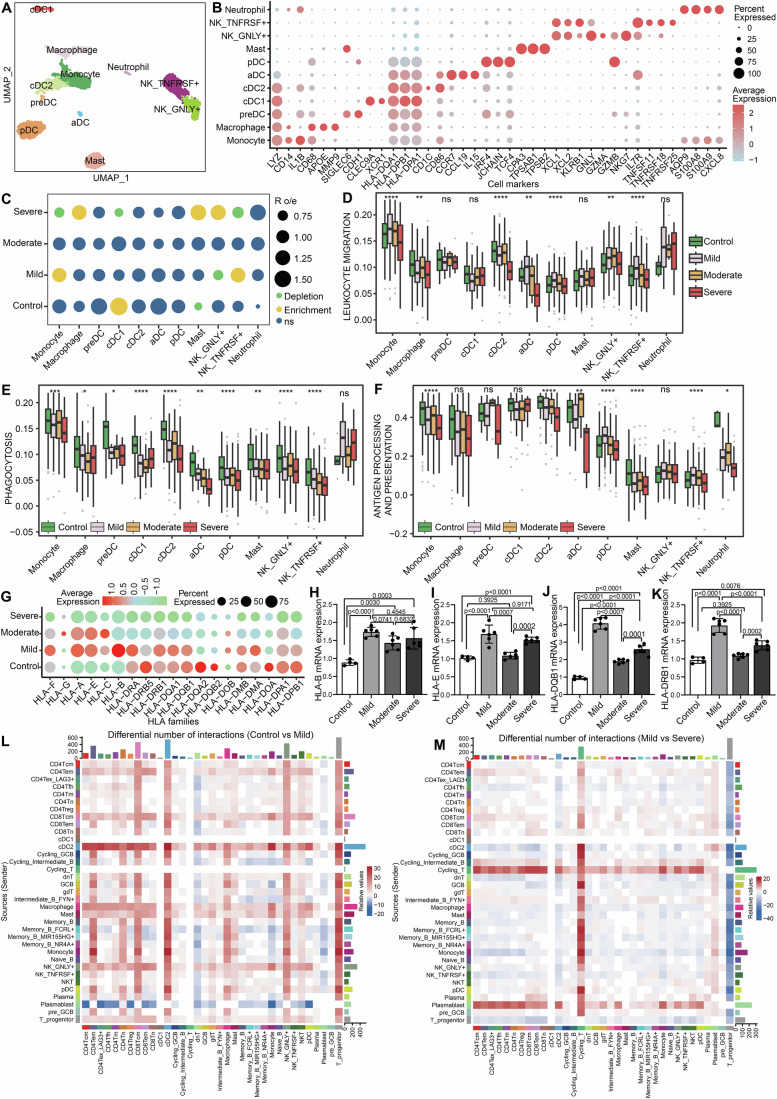
Molecular and cell-cell communication changes in innate immune cell subsets in hypertrophic adenoids. (**A**) Identification of innate immune cell subsets in adenoids at different disease stages. (**B**) Expression levels of marker genes corresponding to innate immune cell subsets in adenoids, used to demonstrate the accuracy and specificity of cell type identification. (**C**) Differences in cell abundance of innate immune cell subsets in adenoids at different disease stages. (**D**–**F**) Changes in the activity of gene sets related to cell migration, phagocytosis, and antigen processing and presentation in innate immune cells of adenoids at different disease stages (normal snoring, mild, moderate, and severe OSA). Minima: lower limit of the whisker; maxima: upper limit of the whisker; center: median line inside the box; the upper and lower box bounds represent the 25th and 75th percentiles of the data. Gene set scoring only represents the overall activity of the transcriptional expression levels of these functional genes. Multiple Kruskal–Wallis tests were used to assess the overall significance of gene set scoring across four groups. (**G**) Dotplot showing the expression changes of different HLA families in innate immune cells of adenoids at different disease stages (normal snoring, mild, moderate, and severe OSA). (**H**–**K**) Q-PCR random verification of the expression levels of HLA families in innate immune cells of adenoids at different disease stages (normal snoring, mild, moderate, and severe OSA). Q-PCR data were shown as mean ± SEM, control = 4 samples, mild = 6 samples, moderate = 6 samples, severe = 6 samples. Statistical testing using one-way analysis of variance. (**L**) Heatmap showing the overall differences in cell-cell communication between the control group (normal snoring) and the mild OSA group. (**M**) Heatmap showing the overall differences in cell-cell communication between the mild OSA group and the severe OSA group. Red indicates that the communication signals between pairwise cells are enhanced, and blue indicates that they are downregulated. Source data are available online for this figure.

Although B and T cells constitute the majority of cells in the adenoid, their functions and activation are typically dependent on assistance and activation by innate immune cells. We performed a detailed subdivision, identifying 11 innate immune cell subsets, including five states of dendritic cells (DCs), monocytes, macrophages, natural killer (NK) cells, as well as mast cells and neutrophils (Fig. 5A,B; Appendix Fig. S4). Cell abundance analysis revealed an increase in macrophages and mast cells in the severe group (Fig. 5C). The main function of myeloid cells is to rapidly migrate to the lesion site and timely phagocytose harmful substances that the body does not need. Our analysis results showed that, the overall migratory score and phagocytic score of leukocytes (NK cells, monocytes, dendritic cells, and macrophages) in the severe OSA group were decreased, particularly when compared to the normal snoring group, with the exception of neutrophils (Fig. 5D,E). In addition, myeloid cells are primarily responsible for antigen uptake and presentation to activate T-cell or B-cell immunity. Surprisingly, the analysis results demonstrated that the antigen presentation and processing score of myeloid cells in the severe OSA group were significantly reduced (Fig. 5F). These results suggest that the transcription of genes related to the mobilization and antigen processing of innate immune cells is generally in an inactive state in children with higher OSA progression.

Remarkable, multiple class I and class II human leukocyte antigen (HLA) molecules were severely downregulated in severe OSA group, but the class I HLA in the normal snoring group was also decreased compared to the mild group, but class II HLA was not (Fig. 5G; Dataset EV9). This indicates that HLA may play a role in different stages of disease progression: the upregulation of class I HLA may drive immune activation or inflammation in the early onset of OSA, while the downregulation of both classes of HLA in the later stage jointly contributes to the deterioration of OSA. QPCR experimental validation results also showed that HLA-B and HLA-E, HLA-DRB1 and HLA-DQB1 were significantly upregulated in the mild OSA group compared with the normal snoring group, but decreased in the moderate and severe OSA groups (Fig. 5H–K). In conclusion, these results indicate that the antigen-processing capacity of innate immune cells in the adenoids of children with moderate and severe OSA is impaired at the mRNA level, which may be related to the aforementioned impairment of T cells and B cells.

Further, cell communication analysis showed that there were many different cell-cell communication signals between the normal snoring group and the mild OSA group. In particular, myeloid cells such as cDC2, macrophage, mast and NK cells mediated many upregulated cell communication intensities in the mild OSA group (Fig. 5L), which indicated that the information flow between immune cells was active in the early stage of OSA, corresponding to the lymphocyte activation and proliferation observed above in the early stage of OSA. However, in the comparison of mild and severe OSA, most of these upregulated myeloid cell-mediated cell interactions were significantly disappeared or even decreased (Fig. 5M), suggesting that the transition of myeloid cell-mediated intercellular communication is associated with the occurrence of OSA and disease aggravation. Overall, these results emphasize that although the proportion of myeloid cells is very low, they showed many important immune-related changes in the progression of OSA.

**Figure EV4:**
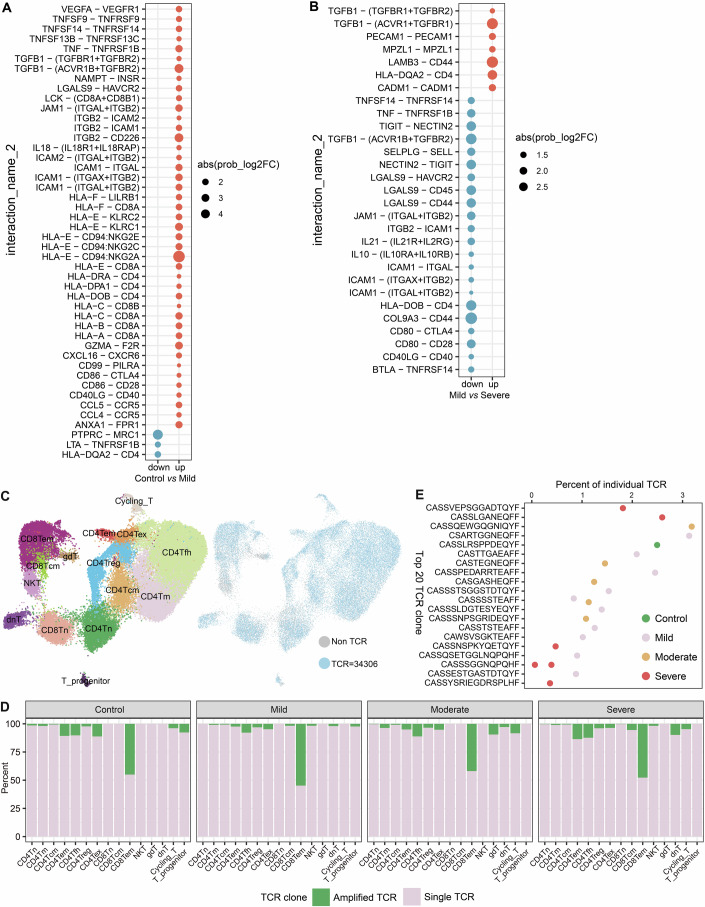
UMAP plot shows the overlap and distribution of detected TCRs with T cell subsets. (**A**) Ligand-receptor pairs that are differentially expressed between control and mild OSA. (**B**) Ligand-receptor pairs that are differentially expressed between mild and severe OSA. (**C**) UMAP plot shows the overlap and distribution of detected TCRs with T cell subsets. (**D**) The proportion of TCRs with clonal expansion in adenoid T cells of different disease courses (normal snoring, mild, moderate, and severe OSA). (**E**) The top 20 TCR clonotypes (heavy chain CDR3) with clonal expansion.

Finally, we conducted an in-depth identification of the ligand-receptor pairs that may drive differential cell-cell communication between different groups. Interestingly, the mild OSA group upregulated many ligand-receptor signaling molecules compared to the control group, such as HLA-E − CD94:NKG2A, ICAM1 − (ITGAL + ITGB2), TGFB1 − (TGFBR1 + TGFBR2), VEGFA − VEGFR1, ANXA1 − FPR1, and CD86 − CD28 (Fig. EV4A). These reflect the immune activation, increased inflammation, and proliferation signals that accompany the early onset of OSA. However, in the comparison between severe OSA and mild OSA, multiple groups of ligand receptor pairs were found to be downregulated, such as COL9A3 − CD44, HLA-DOB − CD4, LGALS9 − CD45, and TGFB1 − (ACVR1B + TGFBR2) (Fig. EV4B), indicating that severe OSA did undergo significant changes in cellular interactions.

### TCR/BCR characteristics with OSA disease progression and HIF1A signals suppressing immunity

**Figure 6:**
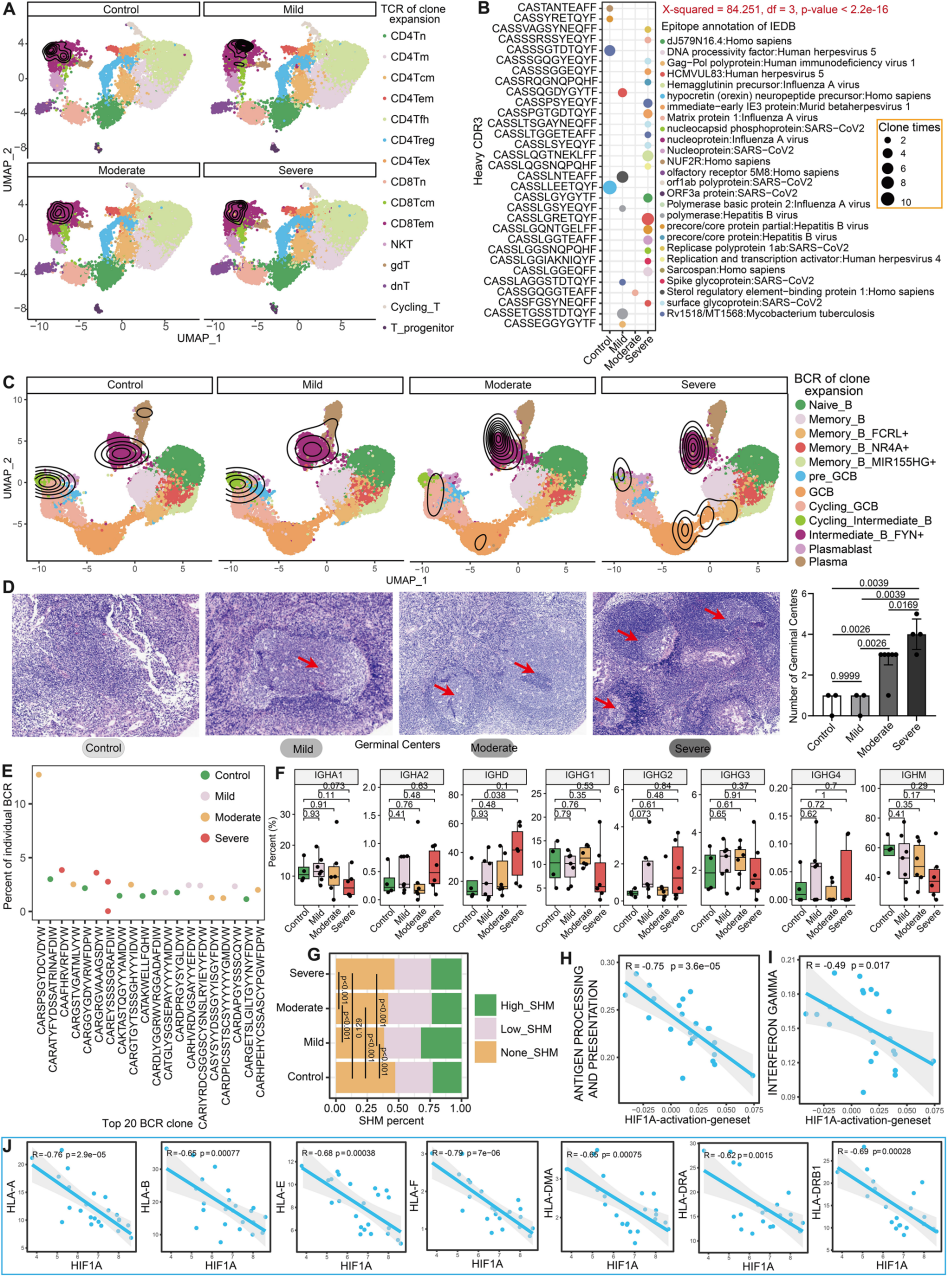
Characteristics of TCR and BCR changes and HIF1A-driven immune suppression in hypertrophic adenoids. (**A**) UMAP plot showing the distribution of clonally expanded TCRs in different T cell subsets. The contour density map represents the enrichment density and range of clonally expanded TCRs. (**B**) Dotplot showing the antigen epitopes annotated (based on the IEDB database) for clonally expanded TCRs, which serves as an indicator of past antigen exposure reflected by TCR imprinting responses in the samples. Multiple chi-square tests were used to evaluate the significance of TCR annotation rates in the IEDB database across different groups. (**C**) UMAP plot showing the distribution of clonally expanded BCRs in different B cell subsets. (**D**) HE staining results showing the number of germinal centers in adenoids at different disease stages (normal snoring, mild, moderate, and severe OSA) and their statistical significance. The red arrows indicate the germinal center areas. HE data were shown as mean ± SEM, control = 3 samples, mild = 3 samples, moderate = 6 samples, severe = 3 samples. Statistical testing using one-way analysis of variance. (**E**) Clonotype sequences (heavy chain CDR3) of the top 20 BCRs with clonal expansion. (**F**) Differences in the proportions of B cells with different class switches (IgD, IgM, IgA, IgD) in adenoids at different disease stages. The two-tailed rank-sum test was used to calculate statistical significance between each pair. (**G**) Proportion of B cells undergoing somatic hypermutation (SHM) in adenoids at different disease stages (normal snoring, mild, moderate, and severe OSA). Chi-square test was used to evaluate the significance of SHM occurrence counts across different groups. (**H**,** I**) Pearson correlation between the scores of antigen processing and presentation gene sets, interferon-gamma gene sets, and HIF1A hypoxia-activated gene set scores in hypertrophic adenoids, respectively. (**J**) Pearson correlation between the expression levels of HLA families and HIF1A gene expression in hypertrophic adenoids. Source data are available online for this figure.

TCR and BCR sequencing was performed to explore the immune repertoire characteristics of adenoid hypertrophy and its association with OSA. For T cells, we obtained 34,306 TCRs (Fig. EV4C). Among them, the clonally expanded T cells were mainly concentrated in CD8 Tem cells, followed by CD4 Tem and CD4 Tfh, suggesting that CD8 Tem may be the dominant T cell clone population driving adenoid hypertrophy (Figs. 6A and EV4D). Interestingly, the moderate and severe OSA groups were significantly enriched in clonally expanded dnT cells compared with the mild OSA group (Figs. 6A and EV4D), and they only appeared in the OSA group, indicating that they may be also related to the early transformation from normal snoring to OSA. For example, the top three clonally proliferating TCRs, CASSVEPSGGADTQYF, CASSLGANEQFF, and CASSQEWGQGNIQYF, were mainly present in the moderate and severe OSA groups (Fig. EV4E).

To annotate the epitopes of the detected TCRs, we compared them with the known IEDB database, which collected existing TCR and epitope information. The severe group had the largest number of TCRs annotated to existing epitopes, including those associated with pathogens such as SARS-CoV-2, influenza virus, herpes virus, and mycobacterium tuberculosis, etc. (Fig. 6B; Dataset EV10). These results suggest that children in the severe group are likely to have been infected with these pathogens, thus leaving TCR imprints. Thus, these TCR imprinting annotations in the severe group may also be related to their previously weakened immunity. In addition to pathogen epitopes, we also annotated some epitopes targeting human self-proteins, such as NUF2R, OR5M8, Sarcospan (SSPN), and hypocretin (orexin) neuropeptide precursor (HCRT) proteins (Figs. 6A; Dataset EV10). The role of these host-derived TCR epitopes in OSA remains to be elucidated.

**Figure EV5:**
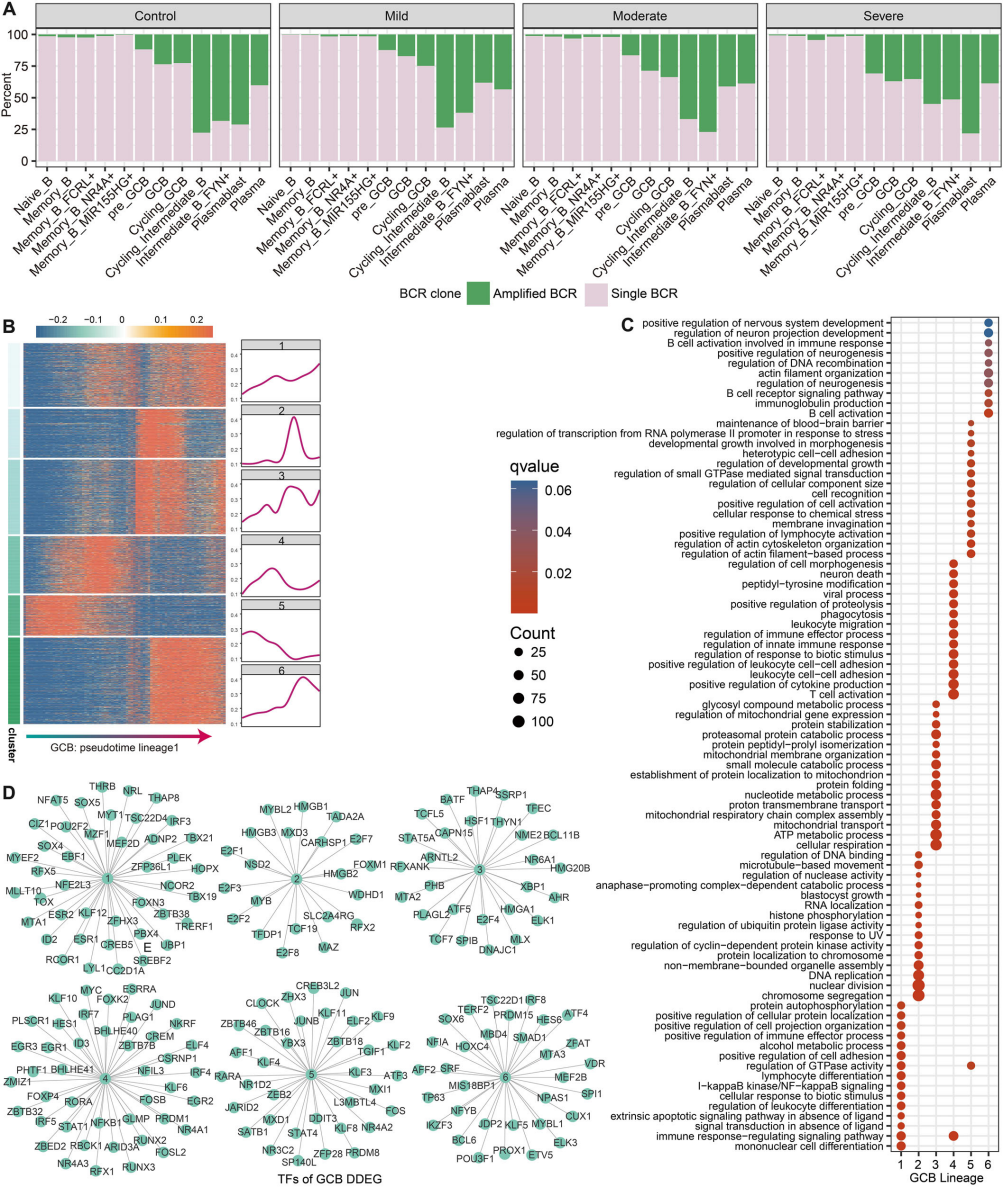
Distribution characteristics of BCR and differentiation trajectory of GCB. (**A**) The proportion of B cells with clonally expanded BCRs in adenoids of different disease courses (normal snoring, mild, moderate, and severe OSA). (**B**) Heatmap shows the clustering of dynamically differentially expressed genes (DDEGs) along the dynamic changes of germinal center B cells. (**C**) Functional enrichment analysis corresponding to DDEG clusters along the GCB differentiation trajectory. (**D**) The dynamic differential transcription factors are contained in the six clusters of GCB pseudotime differentiation trajectories.

For BCRs, a total of 66,688 BCRs were included in the analysis (Appendix Fig. S5). Clonally expanded BCRs were mainly concentrated in intermediate B cells, followed by GCB (Fig. EV5A). The distribution of clonally expanded BCRs showed significant inter-group differences. Compared with the control group and mild OSA group, the proportion of expanded intermediate B cells in the severe OSA group decreased significantly by 10–20%, while the proportion of clonally expanded GCB cells nearly doubled (Figs. 6C and EV5A). Considering the importance of germinal centers (GC) in the progression of OSA, we conducted HE verification and found that the number of GCs indeed increased significantly with the occurrence and severity of OSA (Fig. 6D). Six clusters of DDEGs were identified along the pseudotime of GCB (Fig. EV5B). Among them, cluster 1, whose expression starts to increase gradually in the early stage of GCB, is mainly enriched in immune response and the NF-kappa B signaling pathway (Fig. EV5C), which may imply that the expansion of GCBs is driven by early inflammation and immune activation. Cluster 6, which is upregulated at the terminal stage of GCB differentiation, is mainly enriched in B cell activation, BCR signaling, and DNA rearrangement, and contains transcription factors such as *TP63*, *IKZF3*, *BCL6*, and *MYBL1* (Fig. EV5C,D). In addition, the proportion of clonal plasmablasts increased in the severe OSA group. These changes were generally consistent with the changes in cell proportions, indicating that they might also be related to the severity of OSA. Specifically, CARSPSGYDCVDYW was the BCR with the highest degree of clonal expansion in the moderate group, CAAFHRVRFDYW in the severe group, and CARATYFYDSSATRINAFDIW in the control group (Fig. 6E). Moreover, the top clonally expanded BCRs mainly came from the moderate and severe OSA groups (Fig. 6E).

Furthermore, we compared the proportion of BCR class switching between different groups to reveal OSA-related antibody transition. Interestingly, the severe OSA group showed a significant enrichment of IgD^+^ B cells, which may be related to their impaired humoral immunity, as IgG and IgA typically provide stronger humoral and mucosal immune protection (Fig. 6F). Besides, the results of somatic hypermutation (SHM) in different groups indicated that compared with the control group, the proportion of SHM in the mild OSA group was increased (Fig. 6G). However, during the progression of OSA, SHM in the severe group decreased (Fig. 6G). The reduction in SHM may lead to impaired antibody affinity maturation in the severe OSA group, further weakening B cell-mediated humoral immunity in children with severe OSA.

Finally, the above results show that in addition to the activation of Notch, Hippo and Wnt signals related to growth and development, another typical feature of OSA is the upregulation of HIF1A hypoxia signaling, which reaches the highest level in the severe group. More importantly, the analysis results indicate that both T cell and B cell immunity are decreased in the severe OSA group at the transcriptional level, which is indirectly confirmed by the annotation of more TCR imprints of pathogenic infections. Previous literature has shown that HIF1A can directly regulate and inhibit the expression of multiple HLAs in monocyte-derived macrophages (Jeny et al, 2021). Furthermore, studies have shown that HIF1A induced by tumor hypoxia can bind to the antisense RNA HIF1A-AS2, thereby reducing the level of MHC proteins and the infiltration of CD8 T cells (Liao et al, 2024). To explore whether HIF1A is related to the inhibition of HLA and thus the downregulation of immunity in OSA, we calculated the correlation between the HIF1A-activated gene set score and the antigen presentation score, and the results showed a significant negative correlation between them (R = −0.75, *p* < 0.001) (Fig. 6H). Moreover, the score of the HIF1A-activated gene set was also significantly negatively correlated with the interferon signal (*R* = −0.49, *p* < 0.001) (Fig. 6I). Gene expression correlation analysis also confirmed that HIF1A was significantly negatively correlated with multiple class I and class II HLAs at the mRNA level (*R* < −0.6, *p* < 0.01) (Fig. 6J). These results suggest that hypoxia-induced HIF1A signaling is likely to inhibit HLA and interferon signaling, thereby contributing to the clinically observed downregulation of immunity and antiviral ability in children with OSA (Jeny et al, 2021; Liao et al, 2024). Therefore, the HIF1A signaling axis may become a key target for replenishing the weakened immunity and enhancing anti-infection in children with OSA.

## Discussion

The adenoids are important secondary lymphoid tissue during childhood, primarily composed of lymphocytes (Massoni-Badosa et al, 2024; Xu et al, 2023; Yu et al, 2025). They provide natural resistance and protection against pathogens and foreign bodies in the respiratory tract (Xu et al, 2023; Yu et al, 2025). However, abnormally enlarged adenoids can obstruct the airway, affect a child’s sleep breathing, and even progress to OSA. Clinically, children with abnormally enlarged adenoids often exhibit growth retardation, decreased physical stamina, and weakened immunity (Al-Iede et al, 2025; Yang et al, 2024). How these lymphocytes, which are supposed to enhance immunity, undergo changes and become detrimental remains an important scientific puzzle. Previous studies have analyzed the single-cell atlas of hypertrophic adenoids, revealing their cellular heterogeneity (Yu et al, 2025). However, which cell types primarily drive abnormal proliferation during adenoid hypertrophy remains an unexplored area. Furthermore, the clinical symptoms caused by adenoid hypertrophy exhibit heterogeneity, and disease progression varies, and comparative studies are still lacking. The result of this study indicates that within the adenoid tissues of children suffering from moderate-to-severe OSA, the expression levels of genes associated with innate immunity, including exhaustion-related genes and cytotoxic genes, are notably downregulated. Simultaneously, the migration capacity, phagocytic activity, and antigen processing and presentation functions of cells related to adaptive immunity also exhibit a downward tendency, which implies that the immune function of the adenoids in these children is compromised. These findings can offer a reference for clinical decision-making and assist in guiding parents and clinicians to make more scientific and reasonable judgments regarding whether to conduct an adenoidectomy.

In this study, we found a significant increase in eosinophil infiltration in the adenoid tissue of children with moderate and severe OSA, while neutrophils remained unchanged. This suggests that the progression of OSA may not be simply mechanical obstruction, but rather a complex process of immune, allergic, and inflammatory interactions, as eosinophils are often a hallmark of allergic inflammation and Th2 signaling (Kolbinger et al, 2023). Research has shown that toxic proteins and cytokines released by eosinophils can lead to tissue edema, increased vascular permeability, and exacerbate airway obstruction (Gulotta et al, 2019; Ikegami-Tanaka et al, 2024; Marcuccio et al, 2025). Then, we employed scRNA-seq combined with scTCR-seq/scBCR-seq to investigate the cellular composition and molecular differences of OSA occurrence and progress in adenoids. The single-cell atlas revealed that adenoids are predominantly composed of B and T cells, consistent with prior research (Xu et al, 2023; Yu et al, 2025). Furthermore, our analysis demonstrated that children with OSA exhibit aberrant activation of cell growth and development-related signaling pathways, including Hippo, Wnt, and MAPK, particularly in severe cases. Chronic intermittent hypoxia exposure in OSA significantly enhanced tissue activation of the p38 mitogen-activated protein kinase (MAPK) pathway, oxidative stress damage, and nuclear factor-kappa B (NF-κB) expression levels (Shakir et al, 2025; Torii et al, 2025). These severe cases also displayed abnormally activated hypoxia signaling (HIF1A) alongside reduced ATP levels and impaired oxidative phosphorylation metabolism, potentially explaining the frequent manifestation of physical debility in high-progression OSA children (Semenza, 2011; Vacek et al, 2022). HIF1A, the primary regulator of oxygen metabolism homeostasis, comprises alpha (α) and beta (β) subunits. The oxygen-sensitive HIF-1α subunit undergoes degradation under normoxia but stabilizes under hypoxia (Semenza, 2011; Zhang et al, 2025). Compared to healthy controls, HIF-1α protein levels were elevated in OSA, and intermittent hypoxia specifically upregulated HIF-1α, which exacerbated oxidative stress by increasing reactive oxygen species (ROS) generation through HIF-1-dependent activation of pro-oxidant enzyme genes (Martinez et al, 2019).

The comprehensive analysis comparing normal snoring with OSA of varying severities revealed significant heterogeneity during the progression from general adenoid hypertrophy to early-stage OSA and the subsequent worsening of OSA disease. First, their cytokine expression profiles differed markedly. Specifically, cytokines highly expressed in the severe OSA group, including *CXCL14*, *CXCL12*, *TGFBR1*, *IL33*, *IFNAR2*, and *TGFB1*—may serve as markers for severe disease monitoring. Research indicates that the chemokine CXCL12 exerts a strong chemotactic effect on lymphocytes and plays a crucial role in development (Murdamoothoo et al, 2021). Conversely, specific downregulation of *TNFRSF21*, *TNFRSF10D*, *TNFAIP8*, *TNFRSF10B*, *IL16*, and *IFNGR2* in the normal snoring group may serve as markers for the onset of early OSA. Moreover, results based on fuzzy clustering module analysis indicate that progression from normal snoring to early OSA involves immune cell proliferation, activation, or inflammatory responses. In contrast, progression to severe OSA is characterized by increased cellular proliferation and disordered cell morphology, accompanied by a decline in energy metabolism and immune activation. These results collectively demonstrate that the initial development of early OSA and the subsequent progression to severe OSA are governed by distinct molecular mechanisms.

In the analysis of B-cell and T-cell subpopulations, we identified downregulated B-cell and T-cell immunity in the severe OSA group at the mRNA level. Critically, results based on TCR repertoire analysis also support that the severe OSA group had experienced greater pathogen exposure, aligning with the clinically observed decline in immunity among OSA children. Furthermore, we found that the downregulated T-cell and B-cell immunity may be influenced by diminished antigen presentation and processing capabilities of innate immune cells in the severe OSA group. Moreover, integrating previous literature reports with our analytical results suggests that the downregulation of HLA molecules in adenoid tissue is likely impacted by the upregulated HIF1A hypoxia signaling pathway, as HIF1A shows significant negative correlations with multiple HLA molecules, as well as with antigen processing and interferon responses (Jeny et al, 2021; Liao et al, 2024). Overall, the study suggests that hypoxia-induced HIF1A inhibits immune activation and maturation of dendritic cells, monocytes, and T cells at least at the cell culture level (Jeny et al, 2021; Liao et al, 2024; Mancino et al, 2008). Consequently, the HIF1A hypoxia signaling triggered by adenoid hypertrophy may not only impair children’s respiration and energy metabolism but also suppress immunity in OSA children by downregulating HLA expression (Jeny et al, 2021; Liao et al, 2024; Mancino et al, 2008).

Interestingly, amplified TCR repertoire analysis also identified TCRs targeting multiple host self-proteins. Among these, Sarcospan, associated with the Kras oncogene, is primarily localized to the cytoskeleton and involved in muscle contraction and cell adhesion. It plays a crucial role in maintaining the structural integrity of skeletal and cardiac muscle cells, demonstrating particular efficacy in improving muscle structure integrity in patients with Duchenne muscular dystrophy (DMD) (Mokhonova et al, 2024). NUF2R is also an oncogene primarily involved in the cell division process (Jin et al, 2025). HCRT (Hypocretin/Orexin) is a protein with vital functions in the nervous system, primarily regulating physiological processes such as the sleep-wake cycle, appetite, energy balance, and mood (Bitsikas et al, 2024; Wang et al, 2025). The OR5M8 gene belongs to the olfactory receptor gene family and participates in odorant signal transduction (Malnic et al, 2004). Studies have found that patients with adenoid hypertrophy have autoantibodies or T-cell infiltration; therefore, autoimmune-targeted attacks may exacerbate tissue hyperplasia (Mirrakhimov et al, 2013; Vakil et al, 2018). Furthermore, TCR and BCR repertoire analysis definitively identified the major proliferative T-cell clones driving adenoid hypertrophy as CD8 Tem cells, followed by CD4 Tem and CD4 Tfh cells, a finding previously unreported. Besides, GCB cells were the predominant clonally expanded B-cell population, consistent with prior observations of GCs and cell proportion validation results, thus confirming a significant association between germinal center abnormalities and OSA (Moll and Baumjohann 2025; Shin et al, 2023). Another new discovery is that the severe OSA group also enriched intermediate B cells with clonal expansion, which may also be the source of adenoid hyperplasia cells.

DEG analysis further revealed a highly consistent downregulation of the heat shock protein family in the OSA group. As common molecular chaperones, HSP primarily participate in and assist protein folding and respond to external stress (van Neerven et al, 2022); consequently, the downregulation of these molecules is likely to impair respiratory function in children with severe OSA. Regarding the significantly enriched transcription factors in severe OSA, such as *RARA*, *SP4*, *CLOCK*, *ZBTB21*, *TCF12*, and *RFX1. RARA* encodes retinoic acid receptor alpha, a key transcription factor mediating the retinoic acid signaling pathway, regulating embryonic development, cell differentiation, and proliferation (Duan et al, 2023). SP4 is primarily involved in the development, functional maintenance, and synaptic plasticity regulation of the nervous system (Khayachi et al, 2021). *CLOCK* encodes a core circadian clock protein that, as a transcriptional activator, forms a heterodimer with BMAL1 to drive the expression of circadian rhythm-related genes (Chen et al, 2025; Cox and Takahashi, 2019). *ZBTB21* encodes zinc finger and BTB domain-containing protein 21, functioning in hematopoietic system development, immune cell function, and neurogenesis (Qiao et al, 2024). *TCF12* belongs to the basic helix-loop-helix (bHLH) protein family and is mainly involved in neural development, T-cell differentiation, and myogenesis (Borst et al, 2025). *RFX1* primarily recognizes and binds X-box promoter elements, regulating the cell cycle, DNA damage response, and ciliogenesis (Kubota et al, 2023). Collectively, these transcription factors are implicated in regulating immunity, neurology, proliferation, and biological rhythms. However, their direct relationship with OSA awaits further investigation.

In summary, this study firstly integrated clinical data with scRNA-seq and scTCR-seq/scBCR-seq to demonstrate the single-cell atlas dataset of adenoids in children varying from general adenoid hypertrophy to different severities of OSA. Our results elucidate that adenoids exhibit asynchronous cytokine profiles, functional modules, and transcriptional regulatory networks and immunometabolic balance during the initiation and development of OSA. Interestingly, these features are not synchronized during the onset and exacerbation of OSA. Consistent with clinical phenomena, children with severe OSA displayed significant declines in both T-cell and B-cell immunity, alongside reduced antigen processing and intercellular communication in innate immune cells at the transcriptional level. These deficits may collectively contribute to the children’s diminished immunity and impaired energy metabolism, ultimately leading to the clinically observed increases in infections and developmental issues. Mechanistically, the hypoxia signaling molecule HIF-1α is likely responsible for the downregulated immunity and holds promise as a potential therapeutic target for OSA intervention (Jeny et al, 2021; Liao et al, 2024; Mancino et al, 2008). Overall, these immune understandings of adenoids, especially in children with severe OSA, will also contribute to clinical surgical decision-making.

This study has the following limitations: First, the sample size investigated remains restricted due to the inherent difficulty in obtaining pediatric adenoid tissue. Second, due to factors such as minimal infiltration of granulocytes, insufficient sampling for single-cell preparation, low RNA content and filtration, this study did not detect available granulocytes for analyzing the association between transcriptional changes and OSA. Third, the primary conclusions and validation are based on mRNA-level analyses, while gene function is influenced by post-transcriptional and post-translational modifications. Finally, although this study identified and proposed many key molecules or pathways that may be associated with early onset or exacerbation of OSA, their causal relationships and underlying mechanisms still require further validation.

## Methods

**Table:** Reagents and tools table

Reagent/resource	Reference or source	Identifier or catalog number
**Antibodies**		
anti-HIF-1α	Servicebio	cat#GB151339
anti-Ki67	Servicebio	cat#GB121141
anti-CD3	Servicebio	cat#GB150004
anti-IFN-γ	Servicebio	cat#DF6045
DAPI	Servicebio	cat#G1012
**Oligonucleotides and other sequence-based reagents**		
Single Cell 5’ Library Preparation Kit	SeekOne	Catalog No. K00501
VAHTS DNA Clean Beads	Vazyme	N411-01
Qubit fluorometer	Thermo Fisher Scientific	Q33226
Bio-Fragment Analyzer	Bioptic	Qsep400
Illumina sequence platform	Illumina	NovaSeq 6000
Single Cell V(D)J Enrichment Kit	SeekGene	Catalog No. K00601 & K00701
PCR Primers	This study	Table EV3
**Chemicals, enzymes and other reagents**		
PBS	Hyclone	SH30256.01
Collagenase Ⅱ	Sigma	V900892-100MG
DNase Ⅰ	Sigma	9003-98-9
Erythrocyte depletion kit	Solarbio	R1010
RPMI1640	Gibco	11875119
2% FBS	Gibco	10100147 C
RNeasy kit	TAKARA	Cat No. 9109
Hifair® III 1st Strand cDNA Synthesis SuperMix	Yeasen	Cat No. 11141ES60
**Software**		
Fastp v0.23.1	https://github.com/OpenGene/fastp	NA
SeekSoul® Tools v1.0.0	http://seeksoul.seekgene.com/zh/v1.0.0/index.html	NA
STAR v2.7.1a	https://github.com/alexdobin/STAR/	NA
featureCounts	Liao et al	NA
Seurat v4.3.0	https://satijalab.org/seurat	NA
DoubletFinder v2.0.3	https://github.com/chris-mcginnis-ucsf/DoubletFinder	NA
Harmony v0.1.1	https://github.com/immunogenomics/harmony	NA
Msigdb v7.5.1	https://www.gsea-msigdb.org/gsea/msigdb	NA
PyScenic	https://github.com/aertslab/pySCENIC	NA
scRepertoire v1.7.1	https://github.com/BorchLab/scRepertoire	NA
Mfuzz	http://mfuzz.sysbiolab.eu/	NA
Slingshot v2.2.1	https://github.com/kstreet13/slingshot	NA
tradeSeq	https://github.com/statOmics/tradeSeq/	NA
Cellchat2	https://github.com/SiYangming/CellChat2	NA
ClusterProfiler v4.2.2	https://github.com/YuLab-SMU/clusterProfiler	NA
Change-O - Repertoire clonal assignment toolkit	https://github.com/immcantation/changeo	NA
GraphPad Prism 10.0	https://www.graphpad.com/demos/	NA
Cytoscape v3.8	https://cytoscape.org/	NA
**Other**		
Fluorescence Cell Analyzer	Countstar® Rigel S2	NA

### Research design and medical ethics

To elucidate the pathogenesis of OSA, we conducted a retrospective cross-sectional study utilizing a representative sample of pediatric patients. This study enrolled 19 children diagnosed with OSA by polysomnography (PSG) due to snoring at the otolaryngology and sleep clinics between August 2023 and January 2024. Furthermore, four children who underwent PSG during the same period but were not diagnosed with OSA were recruited as controls. The two groups were matched in terms of age, gender, and weight. The exclusion criteria for the study population were defined as follows: (1) severe pulmonary disease, cardiovascular disease, and hepatic or renal dysfunction; (2) various endocrine and metabolic disorders; (3) craniofacial deformities or congenital anatomical abnormalities of the oral cavity, nasal passages, pharynx, and airway; (4) a history of preterm birth or intrauterine growth restriction; (5) active inflammatory conditions, acute infections, malignancies, or long-term use of anti-inflammatory medications; and (6) the use of medications affecting sleep, such as sedatives and stimulants.

The degree of adenoid obstruction, assessed via fiberoptic nasopharyngoscopy based on the proportion of the nasopharyngeal cavity occupied by adenoid tissue, is classified into four grades. Grade I indicates ≤25% occupancy with no obstruction of the posterior nasal aperture. Grade II indicates >25% to ≤50% occupancy. Grade III indicates >50% to ≤75% occupancy, with partial obstruction of the posterior nasal aperture and Eustachian tube orifice. Grade IV indicates >75% occupancy, with complete obstruction of these structures (Cassano et al, 2003).

All adenoid samples were obtained from the Department of Otolaryngology, Shenzhen Children’s Hospital. Pathological sections of adenoids were borrowed from the Department of Pathology, Shenzhen Children’s Hospital. Ethical approval for the study was obtained from the Institutional Review Board of Shenzhen Children’s Hospital (IRB approval number: 202401002) prior to the commencement of data collection, and written informed consent was obtained from the guardians of all participants. All the experiments involving human participants conformed to the principles set out in the WMA Declaration of Helsinki and the Department of Health and Human Services Belmont Report.

### Sample collection and clinical information

OSA diagnosis was based on the Chinese guideline for the diagnosis and treatment of childhood obstructive sleep apnea (2020), a comprehensive assessment of sleep architecture and respiratory parameters via overnight PSG (≥6 h), performed using commercially available PSG systems (SOMNOmedics V5, Germany; Philips Alice 6, USA). Experienced sleep technologists conducted the PSG, while certified sleep specialists interpreted the recordings according to the American Academy of Sleep Medicine (AASM) Manual for the Scoring of Sleep and Associated Events. Observed indexes included respiratory events, blood oxygen saturation, obstructive and central sleep apnea-hypopnea events, and obstructive apnea-hypopnea index (OAHI). The total sleep period OAHI >1 events/h was taken as the diagnostic threshold of OSA in children (Mild: 1 events/h <OAHI ≤5 events/h; Moderate: 5 events/h <OAHI ≤10 events/h; Severe: OAHI >10 events/h).

### Histology and morphometric

Adenoid tissue samples were fixed immediately following surgical resection in 10% neutral buffered formalin for 24 h. The samples were subsequently dehydrated through a graded ethanol series, cleared with xylene, infiltrated with paraffin wax, and embedded. Serial sections of 4 μm thickness were prepared using a microtome for hematoxylin and eosin (H&E) staining and quantitative histopathological analysis (Control, *n* = 3; Mild OSA, *n* = 3; Moderate OSA, *n* = 6; Severe OSA, *n* = 4).

The analysis of sections focused on the quantification of follicular germinal centers (GCs) counts and inflammatory cell counts. To assess GC counts, five random, non-overlapping fields representative of adenoid parenchyma were selected per section under low-power magnification (30×). The number of germinal centers in each field was recorded, and the average count across the five fields was calculated as the germinal center count for the sample. For inflammatory cell counts (eosinophils and neutrophils), five random, non-overlapping high-power fields per section were selected (400×). The absolute numbers of each cell type within each field were recorded, and the mean counts per field were calculated as the eosinophil and neutrophil counts for each sample (Wang et al, 2023; Yu et al, 2025).

### RNA extraction and reverse transcription-quantitative PCR (RT-qPCR)

The transcription levels of functional genes in children with OSA were evaluated to identify their correlation in control, mild-OSA, moderate-OSA, and severe-OSA groups. Total RNA was extracted from adenoid samples by the RNeasy kit (Cat No. 9109, TAKARA, Japan) and reverse-transcribed into cDNA using Hifair® III 1st Strand cDNA Synthesis SuperMix (Cat No. 11141ES60, Yeasen, China). Real-time PCR amplification was conducted using the SYBR Green Q-PCR kit on an iCycler-iQ system, with the specific primer sequences detailed in Table 1. RT-qPCR analysis was conducted on the LineGene 9600 Plus fluorescence quantitative PCR detection system (FQD-96A, BIOER, China). The amount of cDNA templates per reaction was normalized using employing glyceraldehyde 3-phosphate dehydrogenase (GAPDH) expression as a reference, and the relative expression of the target gene as compared to the control samples was calculated by the ΔΔCT method (Livak and Schmittgen 2001). The primer sequences for all genes used in Q-PCR refer to Table EV3.

### Immunofluorescence experiment and analysis

Sections were cut from paraffin-embedded samples from the analyzed patients, which were collected from the Department of Pathology, Shenzhen Children’s Hospital. The antibodies used in the present study were anti-HIF-1α (Servicebio, cat#GB151339, 1:3000), anti-CD3 (Servicebio, cat#GB150004, 1:3000), anti-Ki67 (Servicebio, cat#GB121141,1:1000), anti-IFN-γ (Servicebio, cat#DF6045, 1:1000). Multiplex immunofluorescence staining was performed using Tyramide signal amplification (TSA) technology, and according to the manufacturer’s protocol. After dewaxing and rehydration, paraffin sections underwent antigen retrieval and endogenous peroxidase blocking. The sections were then incubated with the primary antibody at 4 °C overnight, followed by incubation with an HRP-conjugated secondary antibody and tyramide signal amplification (TSA) with a fluorophore. For multiplex labeling, the antibody complex was eluted before repeating the primary-secondary-TSA cycle for the next target. Finally, nuclei were counterstained with DAPI (Servicebio, cat#G1012), autofluorescence was quenched, and the sections were mounted with an anti-fade mounting medium (Servicebio, cat#G1401) for imaging. For immunofluorescence quantification of HIF-1α, CD3, Ki67, and IFN-γ, slides were scanned using the Slide Viewer platform and images were quantified using the Saiviewer software (V.2.2.2).

### Single cell preparation

After harvest, tissues were washed in ice-cold PBS (Hyclone SH30256.01) and dissociated using Collagenase Ⅱ (Sigma, V900892-100MG) according to instructions. DNase Ⅰ (Sigma 9003-98-9) treatment was optional according to the viscosity of the homogenate. Cell count and viability was estimated using Fluorescence Cell Analyzer (Countstar® Rigel S2) with AO/PI reagent after removal of erythrocytes (Solarbio R1010) and then debris and dead cells removal was decided to be performed or not (Miltenyi 130-109-398/130-090-101). Finally, fresh cells were washed twice in RPMI1640 (Gibco 11875119) and then resuspended at 1 × 10^6^ cells per ml in RPMI1640 and 2% FBS (Gibco 10100147 C).

### Single-cell RNA-seq library construction and sequencing

Single-cell RNA-seq libraries were constructed using the SeekOne® DD Single Cell 5’ Library Preparation Kit (SeekGene Catalog No. K00501) (Huang et al, 2025; Zhang et al, 2025). Briefly, an appropriate number of cells were mixed with reverse transcription reagents and loaded into the sample well of the SeekOne® DD Chip S3. Subsequently, Gel Beads and Partitioning Oil were dispensed into their respective wells on the chip. Following emulsion droplet generation, reverse transcription was carried out at 42 °C for 90 min and then inactivated at 85 °C for 5 min. Next, cDNA was purified from the disrupted droplets and amplified via PCR. The amplified cDNA products were subjected to fragmentation, end repair, A-tailing, and ligation with sequencing adapters. Finally, indexed PCR was performed to amplify the DNA, which also contained Cell Barcodes and Unique Molecular Indices. The indexed sequencing libraries were purified using VAHTS DNA Clean Beads (Vazyme N411-01) and quantified using a Qubit fluorometer (Thermo Fisher Scientific Q33226) and Bio-Fragment Analyzer (Bioptic Qsep400). These libraries were then sequenced on an Illumina NovaSeq 6000 platform with a paired-end 150 bp read length, generating at least 120G of data per sample.

### Single cell V(D)J-seq library construction and sequencing

Single-cell TCR and BCR libraries were constructed using the SeekOne® DD Single Cell 5’ Library Preparation Kit (SeekGene Catalog No. K00501) and SeekOne® DD Single Cell V(D)J Enrichment Kit (human TCR and BCR; SeekGene Catalog Nos. K00601 and K00701). Briefly, an appropriate number of cells were mixed with reverse transcription reagents and loaded into the sample well of the SeekOne® DD Chip S3. Subsequently, Gel Beads and Partitioning Oil were dispensed into their respective wells on the chip. Following emulsion droplet generation, reverse transcription was performed at 42 °C for 90 min and then inactivated at 85 °C for 5 min. Next, cDNA was purified from the disrupted droplets and amplified via PCR. The amplified 5ʹ cDNA products were then subjected to V(D)J enrichment amplification. The resulting V(D)J amplification products underwent fragmentation, end repair, A-tailing, and ligation with sequencing adapters. Finally, indexed PCR was conducted to amplify the DNA, which also contained cell barcodes and unique molecular indices. The indexed sequencing libraries were purified using VAHTS DNA Clean Beads (Vazyme N411-01), and their quality and quantity were analyzed using a Qubit fluorometer (Thermo Fisher Scientific Q33226) and Bio-Fragment Analyzer (Bioptic Qsep400). These libraries were subsequently sequenced on an Illumina NovaSeq 6000 platform with a paired-end read length of 150 bp.

### Single-cell data alignment

The downloaded data underwent quality control (QC) using Fastp software (v0.23.1), which involved removing low-quality reads, removing adapters, and calculating the sequencing error rate. Subsequently, the clean reads were further quality-controlled using SeekSoul® Tools (v1.0.0). STAR software (v2.7.1a) was used to align the sequencing data marked with Barcodes and UMI to the reference genome. Then, the featureCounts software was employed for statistical analysis of the expression profile (Liao et al, 2014). Reads with the same Barcode and UMI were considered as repeated sequencing data of the same molecule, and were merged during the statistical analysis to obtain a preliminary expression matrix. The preliminary expression matrix contained data from both cells and non-cells (background), and was further filtered to obtain the expression matrix of cells. The preliminary expression matrix was filtered by combining the analysis methods of Cell Ranger and EmptyDrops (Lun et al, 2019). First, a desired number of cells (N, defaulted to 3000) was specified. Then, Barcodes were sorted according to their total UMI counts in descending order. Filtering was performed using the following two methods: The first method involved taking the 99% quantile of the top N UMI values as the maximum estimated total UMI count (m), and considering Barcodes with UMI counts exceeding m/10 as the finally captured cells. The second method utilized RNA expression characteristics to distinguish between cells and background, which allowed for the identification of cells with low RNA expression levels. The cell expression data obtained from both methods were merged to obtain complete cell expression data for subsequent analysis.

### Single-cell immunome library assembly

Using SeekOne® Tools software, clean reads are aligned with known human V(D)J fragments (Shugay et al, 2015). Paired reads that have at least 15 bp aligned to the fragments in this region are retained. Then, Contigs are assembled and annotated for each cell. The retained Contigs from individual cells will be used for the subsequent assembly of consistent sequences in the sample. For high-quality Contigs in the sample, all Contigs from effective cells in the sample will be integrated for further assembly of consistent sequences, and the results of integrated consistent sequence assembly and annotation will be used for subsequent TCR/BCR typing (Shugay et al, 2015).

### Single-cell data analysis

The Seurat R package (v4.3.0) was used to integrate and analyze single-cell data clusters across different samples. Strict quality control criteria were implemented, including the removal of cells with mitochondrial gene proportions exceeding 5%, as well as those with UMI counts below 1000 or above 14,000, and gene counts below 500 or above 4500. Cells with ribosomal gene proportions exceeding 40% were also excluded. These criteria aimed to eliminate dead cells and RNA-degraded cells. Furthermore, the DoubletFinder R package (v2.0.3) was applied to identify and remove doublets. Normalization and standardization were performed using Seurat, and the top 2500 genes with the highest variance were selected for principal component analysis (PCA) and downstream clustering. The canonical correlation analysis (CCA) + anchor and Harmony algorithm was applied to mitigate batch effects during cell clustering (Korsunsky et al, 2019; Stuart et al, 2019). Following evaluation of principal components (PCs) and the inflection point, the top 30 PCs were selected for clustering, with varying resolutions used to identify distinct cell clusters. Dimensionality reduction and visualization were performed using uniform manifold approximation and projection (UMAP). A detailed workflow can be referenced in the tutorial at the following website (https://satijalab.org/seurat/v4.0/pbmc3k_tutorial.html).

### Cell type annotation

All cells underwent nonlinear dimensionality reduction via UMAP and were clustered based on shared features. The top 50 most highly expressed genes in each cluster of cells at different resolutions were identified using Seurat’s FindAllMarkers*()* function, and cell types were confirmed through literature review and a single-cell annotation database (Hu et al, 2023; Meng et al, 2023). Additionally, the “Azimuth” multimodal artificial intelligence algorithm was employed to aid in identifying cell types and enhance reliability (Hao et al, 2021). Clusters expressing two or more classical cell type markers were classified as bimodal and were subsequently removed.

### Differential gene identification

Differentially expressed genes (DEGs) between different cell groups were identified using the FindMarkers() function in the Seurat package. Only genes expressed in at least 25% of cells within any group were considered, which can reduce false positives caused by individual outlier samples. DEG thresholds were set at |logFC|>0.25 and *p*val.adjust <0.05.

### Gene set activity analysis of single cells

The Seurat function AddModuleScore() was employed to calculate gene set activity scores per cell. The algorithm operates as follows: (1) Compute the average expression of the entire gene set; (2) Partition the expression matrix into intervals based on this mean value; (3) Randomly select non-gene-set control genes (100 by default) from each interval; (4) The enrichment score is then obtained as the difference between the mean expression of the target genes and the mean expression of the sampled background genes. The gene sets were derived from the msigdb R package (v7.5.1). Additionally, the cytotoxic activity of T cells was defined by a gene set containing 12 genes (*PRF1*, *IFNG*, *GNLY*, *NKG7*, *GZMB*, *GZMA*, *GZMH*, *KLRK1*, *KLRB1*, *KLRD1*, *CTSW*, and *CST7*), while the T cell exhaustion includes (*LAG3*, *TIGIT*, *PDCD1*, *CTLA4*, *HAVCR2*, and *TOX*). Manually collected genes activated by the HIF1A hypoxia signaling pathway include *HIF1A*, *HIF3A*, *AKT1*, *AKT2*, *AKT3*, *ANGPT1*, *ANGPT2*, *ANGPT4, EGFR*, *EPO*, *FLT1*, *IGF1R*, *INS*, *INSR*, *MAPK1*, *MAPK3*, *PDHA1*, *PDHA2*, *PIK3CA*, *PIK3CB*, *PIK3CD*, *PIK3R1*, P*IK3R2*, *PIK3R3*, *PRKCA*, *PRKCB*, *PRKCG*, *RBX1*, *STAT3*, *TIMP1*, *VEGFA*, and *VHL*.

### Single-cell transcription factor and target gene analysis

We employed the single-cell regulatory network inference and clustering algorithm (SCENIC, v1.3.1) to identify enriched transcription factors (TFs) and their target genes at the single-cell resolution (Aibar et al, 2017). SCENIC reconstructs gene regulatory networks (GRNs) by integrating single-cell transcriptomic data with co-expression analysis and transcription factor binding motif analysis. The algorithm leverages the “RcisTarget” framework to infer enriched TF motifs and potential regulatory interactions, which are then assembled into regulatory modules (regulons). Cells with significantly active regulons were identified using an area-under-the-curve (AUC) scoring system, and all analyses were performed via the “PyScenic” software. Differential enrichment of TFs (based on AUC values) across groups was identified using a rank-sum test. Transcription factors that are enriched in at least 25% of the cells, with a False Discovery Rate (FDR) less than 0.05, and exhibit a differential fold change greater than 1.2 or less than 0.8 across any group, are considered differentially enriched transcription factors.

### TCR/BCR and analysis

For TCR analysis, we included only cells expressing a functional T cell receptor beta chain (TRB). Cells with multiple TRA or TRB chains were retained based on the highest-expressing chain. Clonotypes were defined by the unique CDR3 amino acid sequence of the TRB chain. A similar approach was applied to BCR analysis, where we retained cells with a functional immunoglobulin heavy chain (IGH) and identified clonotypes by unique IGH sequences. We utilized the scRepertoire R package (v1.7.1) to process and analyze single-cell immune repertoire data, computing clonotype diversity metrics and TCR/BCR gene usage frequencies (Borcherding et al, 2020). Clonotypes with TCR or BCR information were then mapped to their corresponding single-cell transcriptomic profiles for integrated analysis. For B cells, immunoglobulin isotype (IgA, IgD, IgG, IgM, or IgE) was determined based on the BCR constant region. To annotate the epitope information that TCR may target, we utilized the Integrated Experimental Biology Database (IEDB, https://www.iedb.org/home_v3.php), which provides validated TCR sequences and epitope information. Our workflow involved: (1) downloading and organizing validated CDR3 heavy chain sequences and epitope data from IEDB; (2) analyzing TCR sharing within our TCR data.

### Genes altered along with the severity of OSA disease

To globally identify genes that vary across cell types along the severity of OSA disease, we applied the Mfuzz fuzzy clustering algorithm (http://mfuzz.sysbiolab.eu/) to single-cell data. First, we extracted the average expression matrix for each sample and further included genes that were differentially expressed in at least one cell type across any two groups for subsequent clustering analysis to identify gene modules with different expression trends.

### Cell trajectory analysis

The slingshot algorithm was used to simulate the differentiation trajectory between cell types, and the pseudotime was mapped to the UMAP (Street et al, 2018). The tradeSeq algorithm is employed to compute differentially expressed genes along the pseudotime differentiation trajectory (Van den Berge et al, 2020), and all differentially expressed genes are clustered using the Mfuzz algorithm to identify patterns of change along the pseudotime axis.

### Cell-cell communication analysis

The Cellchat2 algorithm is used to calculate the overall differences in the quantity and intensity of cell-cell communication between different groups (Jin et al, 2025). The threshold values for ligands and receptors with differences are logFC >1 and *p* value <0.05.

### Functional enrichment analysis

Gene ontology (GO) and KEGG pathway enrichment analyses were performed on the genes using the ClusterProfiler R package (Xu et al, 2024) or the Metascape database (Zhou et al, 2019).

### Somatic hypermutation (SHM) analysis

The Change-O Repertoire clonal assignment toolkit was used to convert the BCR data into 10x VDJ format. Then, the Shazam R package was applied to align the detected BCR sequences to the germline BCR sequences and calculate the mutation sites and frequencies of each BCR.

### Statistical analysis

GraphPad Prism 10.0 was used for statistical analysis of the results. The differences among multiple groups, two-tailed ANOVA was first employed, followed by Bonferroni multiple comparison tests. Results are expressed as mean ± SEM, median [M (25 and 75%)] and qualitative variables as *n* (%); *p* < 0.05 was considered significant. Single-cell analysis was carried out using R software (version 4.3). Regulatory and functional enrichment networks were visualized with Cytoscape (version 3.8). Differentially expressed genes (DEGs) and differentially active transcription factors between groups were identified using the default Wilcoxon rank-sum test, with *p* values adjusted using the Bonferroni correction.

## Supplementary information

Appendix

Table EV1

Table EV2

Table EV3

Peer Review File

Dataset EV1

Dataset EV2

Dataset EV3

Dataset EV4

Dataset EV5

Dataset EV6

Dataset EV7

Dataset EV8

Dataset EV9

Dataset EV10

Source data Fig. 1

Source data Fig. 2

Source data Fig. 3

Source data Fig. 4

Source data Fig. 5

Source data Fig. 6

Figure EV3 Source Data

Expanded View Figures

## Data Availability

The raw data reported in this paper have been deposited in the Zenodo database (https://zenodo.org/records/16744164) and supplementary attachments. The source data of this paper are collected in the following database record: biostudies:S-SCDT-10_1038-S44321-026-00419-3.

## Code availability

The raw data reported in this paper have been deposited in the Zenodo database (https://zenodo.org/records/16744164) and supplementary attachments.
