# Supplementary material for: Disease-stage-specific immunometabolic remodeling in pediatric obstructive sleep apnea: a single-cell transcriptomic atlas of adenoid tissue
Source: EMBO Mol Med. 2026 Apr 27;18(6):2483–513. doi: 10.1038/s44321-026-00419-3 (PMC13270133; doi:10.1038/s44321-026-00419-3)
Supplement: Supplementary file 1 — Appendix [file 44321_2026_419_MOESM1_ESM.pdf]

Contents:

Appendix Figure S1……………………………………………………………………….2

Appendix Figure S2……………………………………………………………………….3

Appendix Figure S3……………………………………………………………………….4

Appendix Figure S4……………………………………………………………………….5

Appendix Figure S5……………………………………………………………………….6

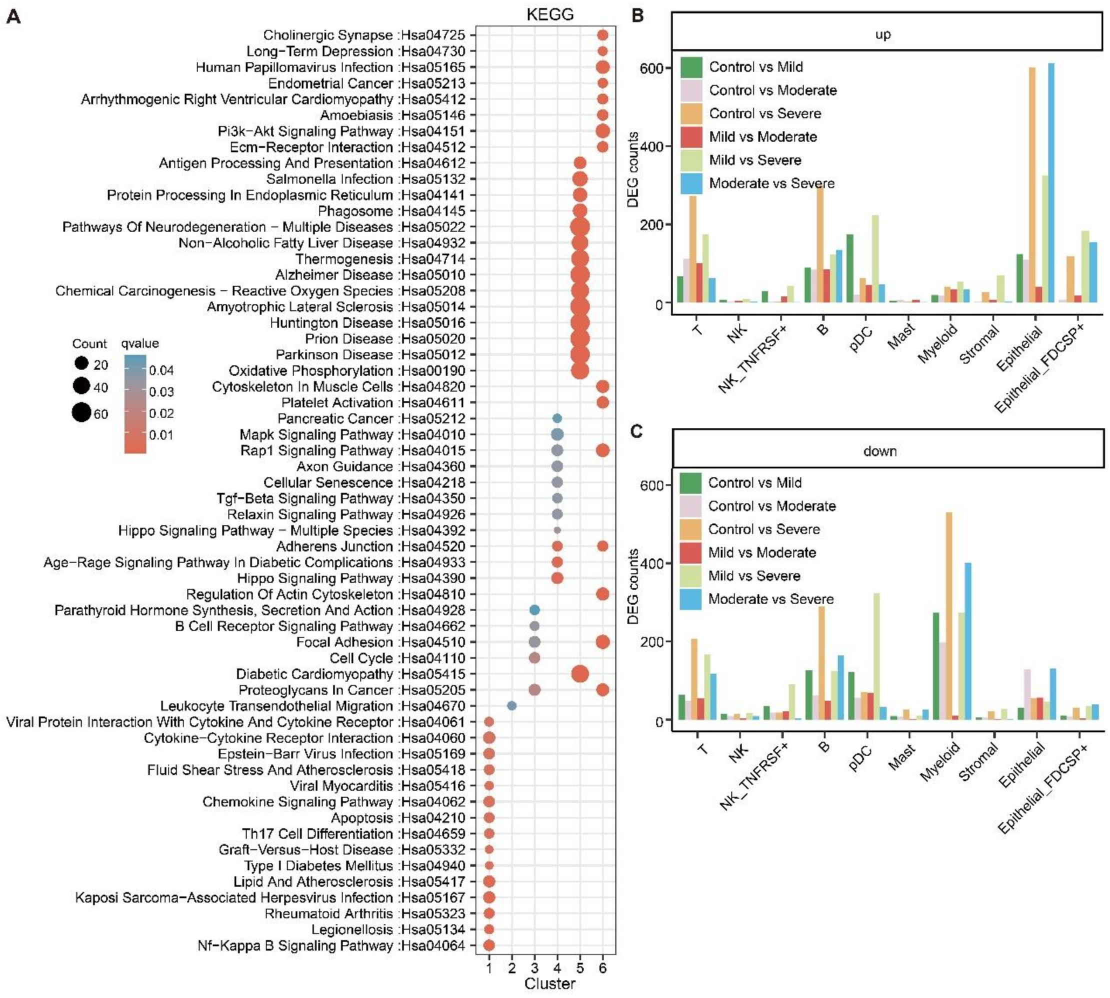

Appendix Figure S1. The enriched pathways related to the progression of OSA disease and the differential gene numbers among different main cell populations.

(A) KEGG functional enrichment analysis of 6 gene clusters identified by the fuzzy clustering algorithm along the progression of OSA. (B-C) The number of up-regulated and down-regulated differentially expressed genes in major cell populations of adenoids with different disease courses, reflecting the overall degree of disordered expression in different cell populations between groups.

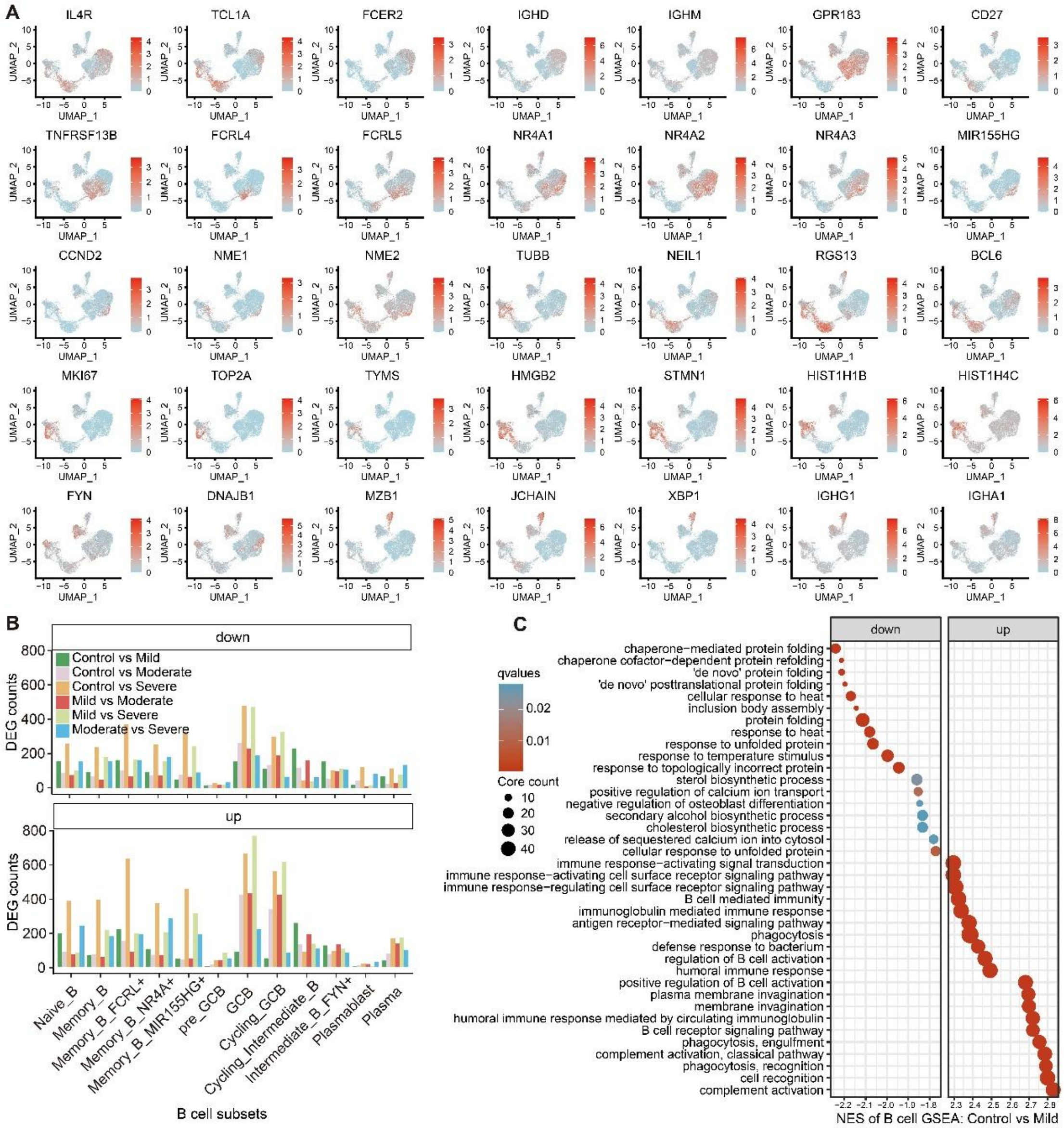

Appendix Figure S2. The markers related to B cell subpopulations, as well as the number of differentially expressed genes and GSEA functional differences between groups.

(A) UMAP plot shows the expression level distribution of marker genes corresponding to different B cell subsets. (B) The number of up-regulated and down-regulated differentially expressed genes in B cell subsets of adenoids with different disease courses. (C) GSEA enrichment analysis of the total B cell expression profiles between the control and mild OSA groups, used to show the biological processes that are significantly upregulated or downregulated between the groups.

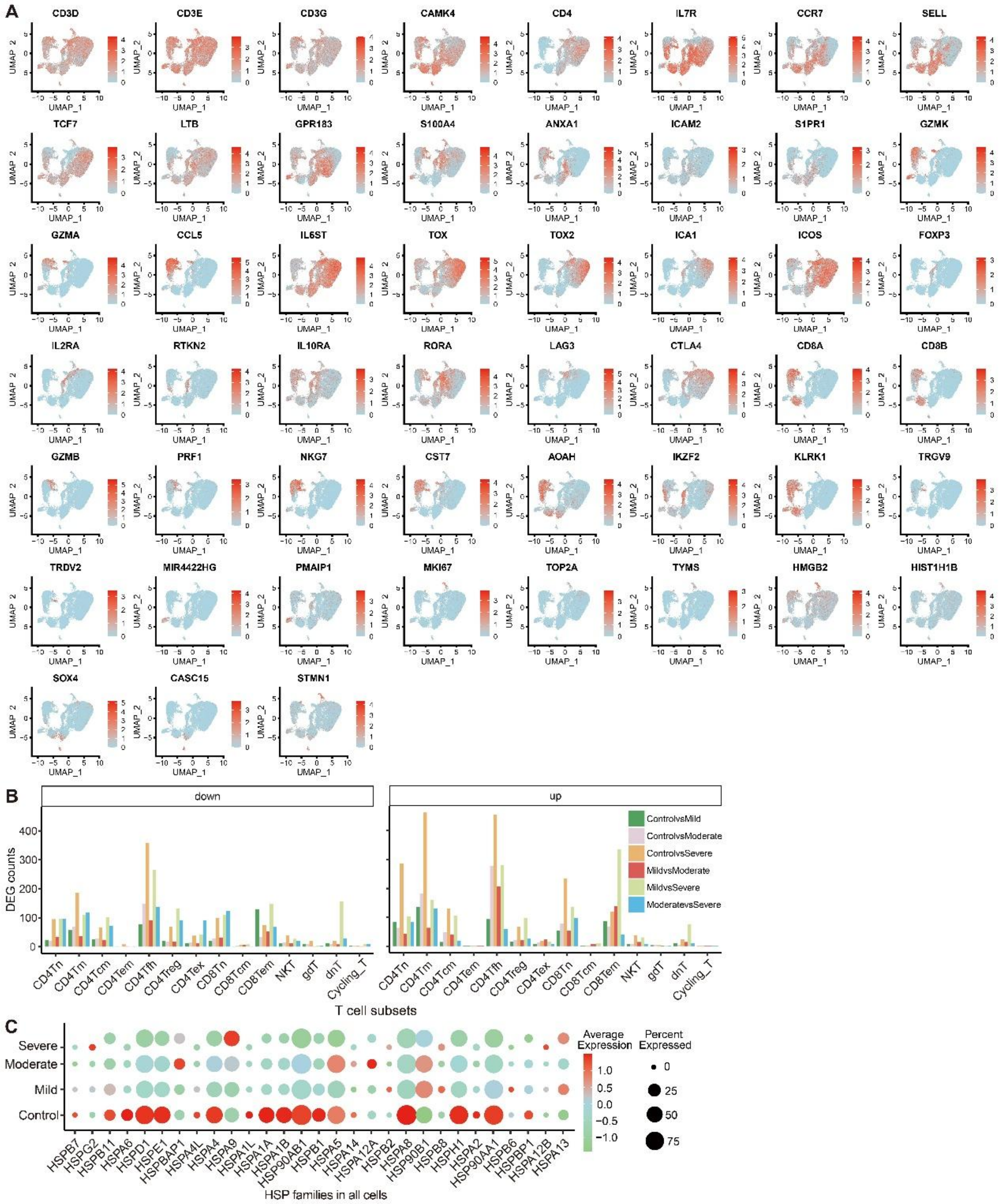

Appendix Figure S3. The markers related to B cell subpopulations, as well as the number of differentially expressed genes and GSEA functional differences between groups.

(A) UMAP plot shows the expression level distribution of marker genes corresponding to different T cell subsets. (B) The number of up-regulated and down-regulated differentially expressed genes in T cell subsets of adenoids with different disease courses. (C) The bubble plot displays the expression of heat shock protein encoding genes among four groups.

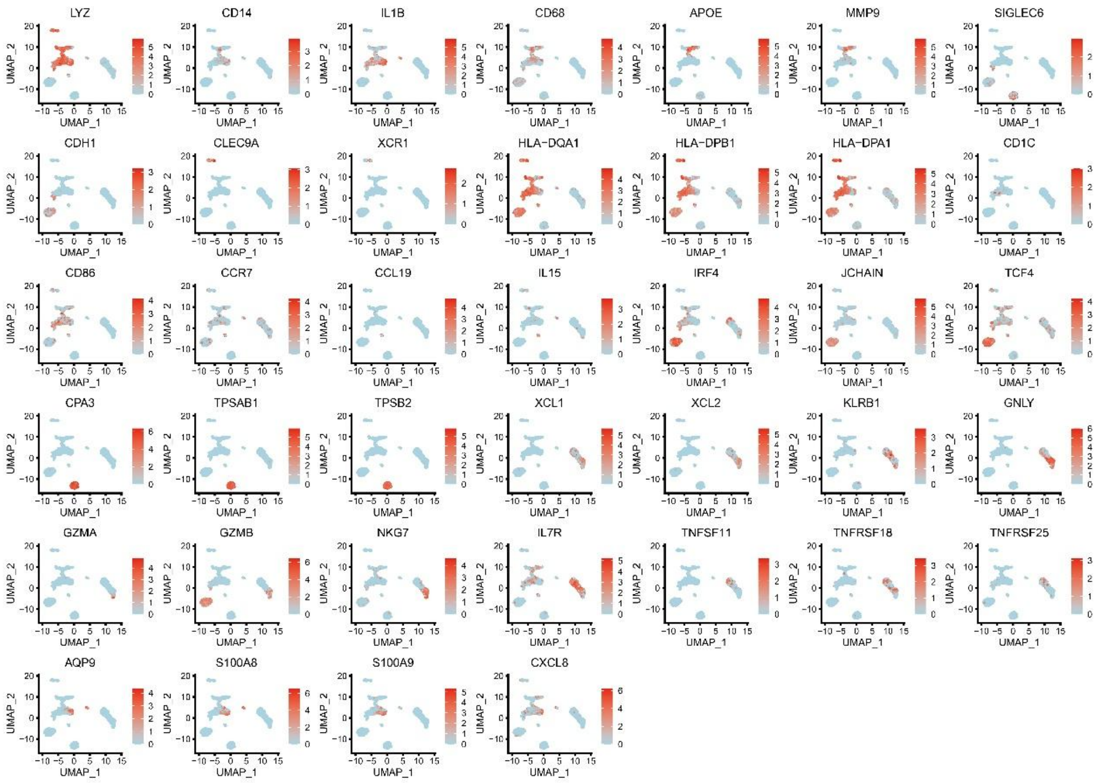

Appendix Figure S4. UMAP plots show the expression levels of markers corresponding to innate immune cell subsets in hypertrophic adenoids.

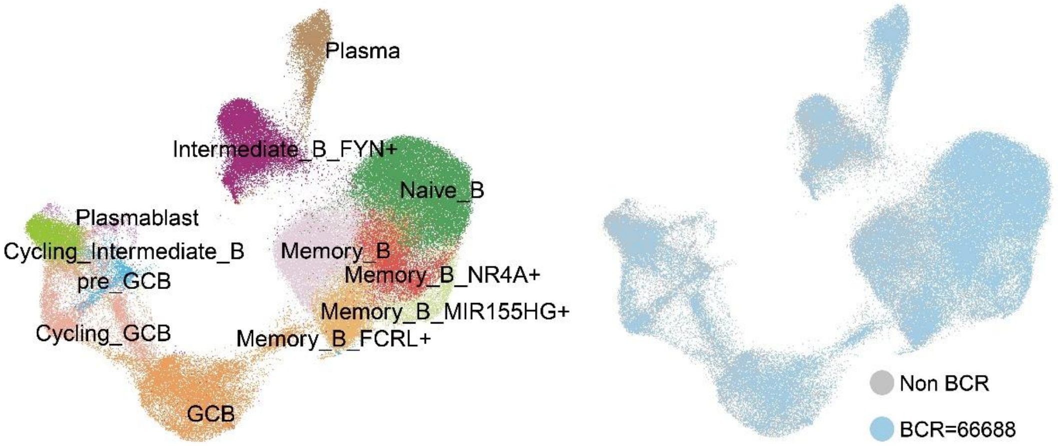

Appendix Figure S5. UMAP plot shows the overlap and distribution of detected BCRs with B cell subsets.
