# Supplementary figures and images for: Disease-stage-specific immunometabolic remodeling in pediatric obstructive sleep apnea: a single-cell transcriptomic atlas of adenoid tissue
Source: EMBO Mol Med. 2026 Apr 27;18(6):2483–513. doi: 10.1038/s44321-026-00419-3 (PMC13270133; doi:10.1038/s44321-026-00419-3)

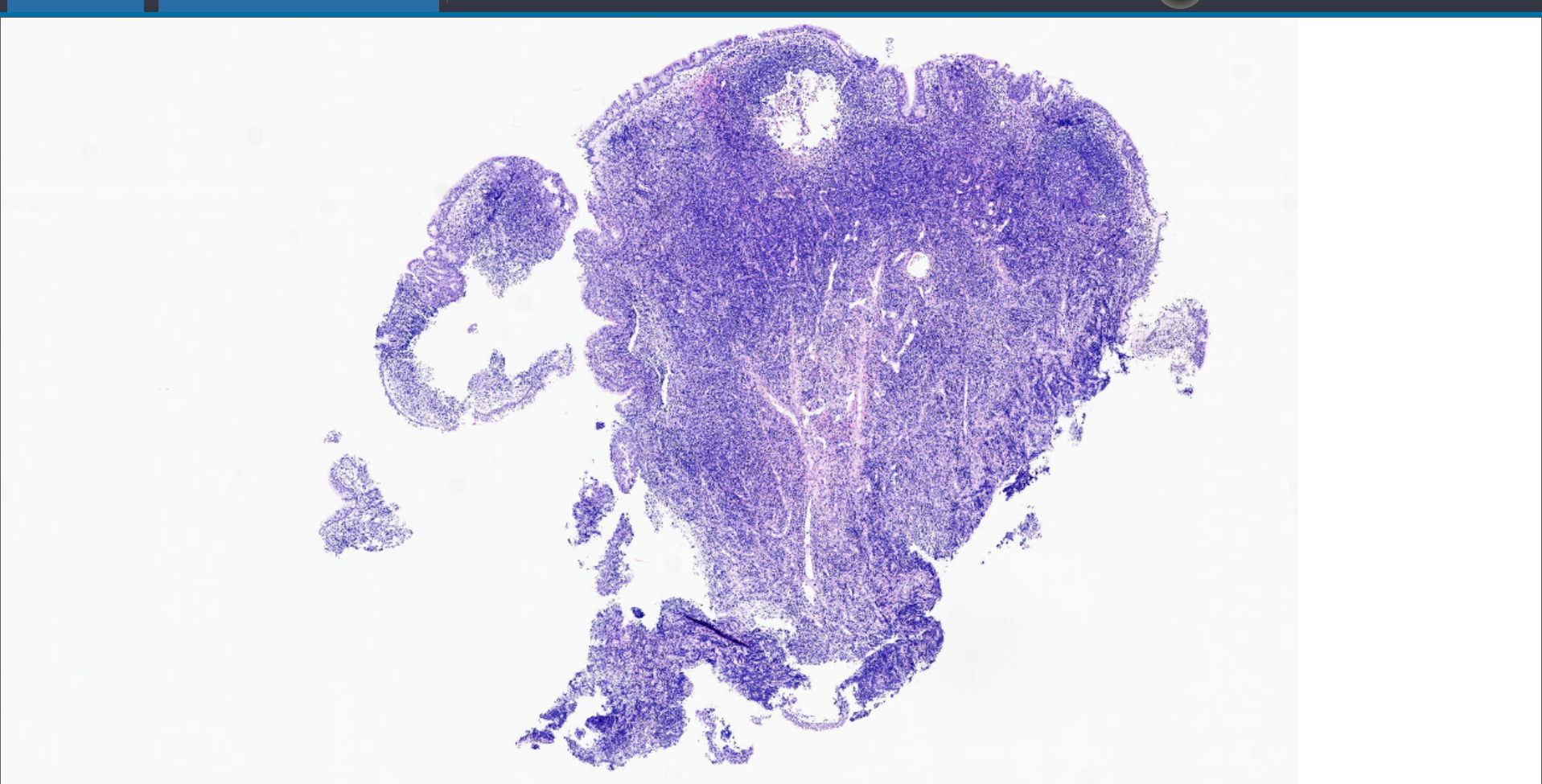

Supplement: Supplementary file 16 — Source data Fig. 1 [file 44321_2026_419_MOESM16_ESM.zip › Source data Fig.1/Fig 1B/202308905.jpg]

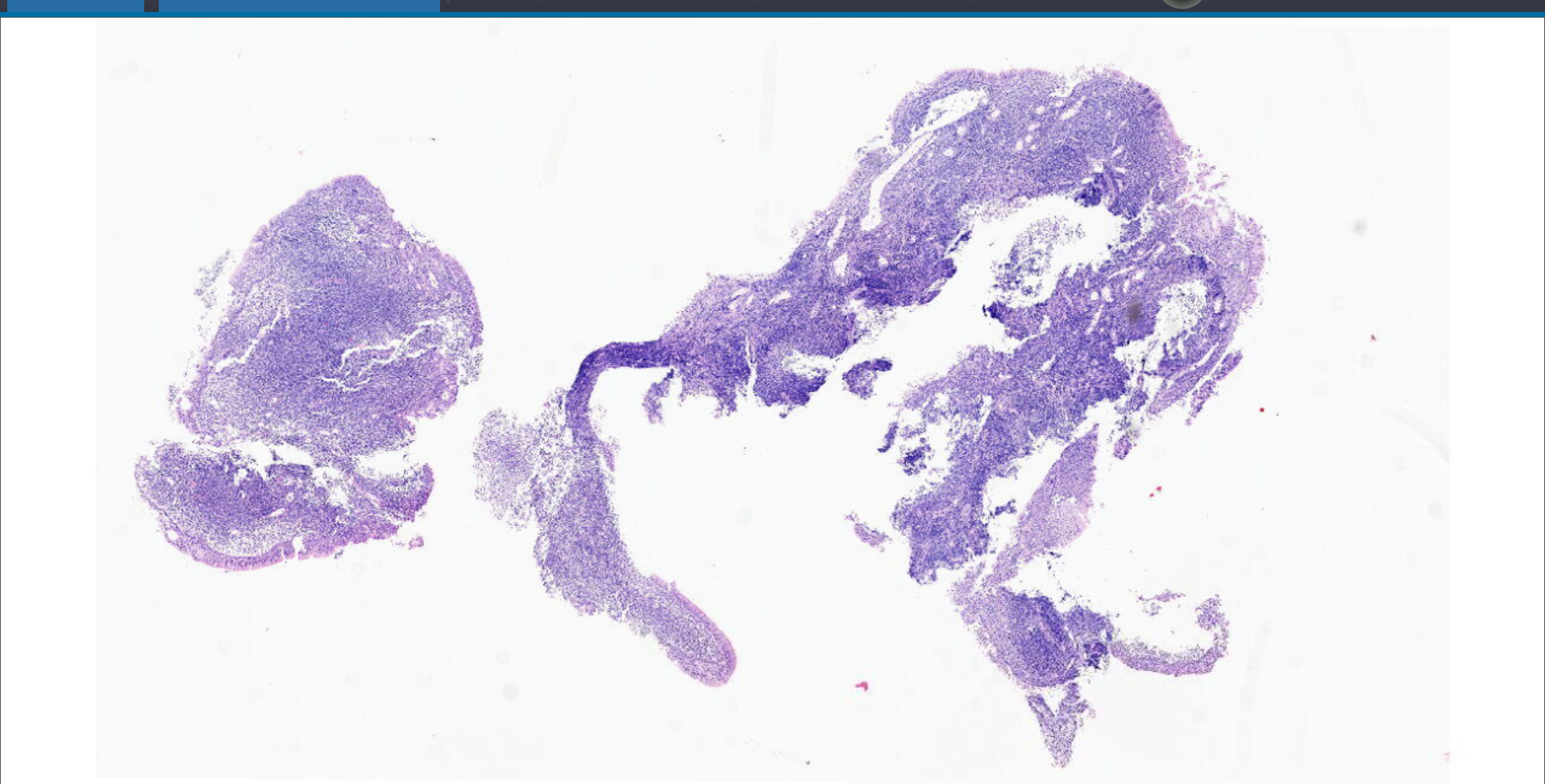

Supplement: Supplementary file 16 — Source data Fig. 1 [file 44321_2026_419_MOESM16_ESM.zip › Source data Fig.1/Fig 1B/202300439.jpg]

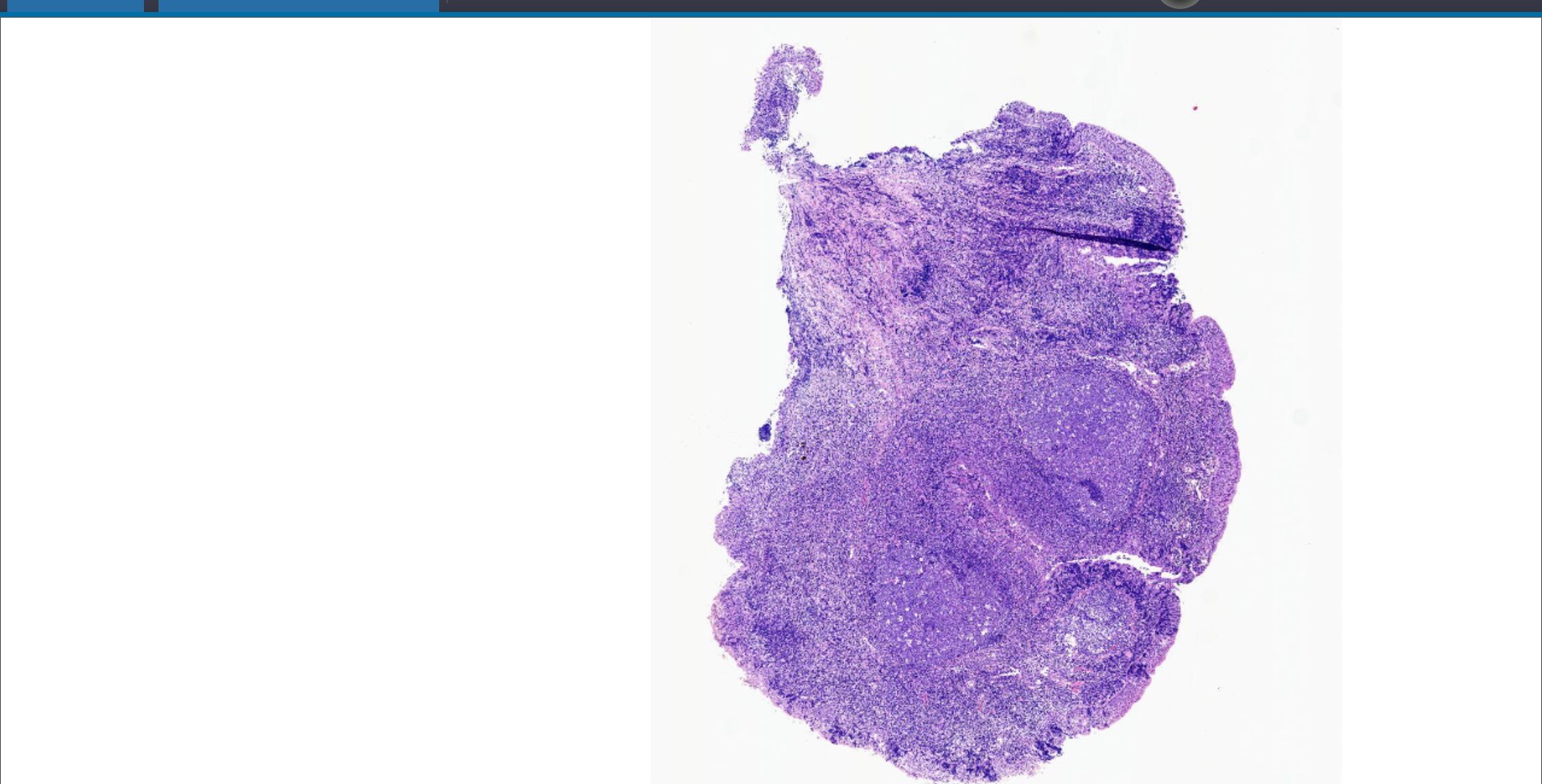

Supplement: Supplementary file 16 — Source data Fig. 1 [file 44321_2026_419_MOESM16_ESM.zip › Source data Fig.1/Fig 1B/202301700.jpg]

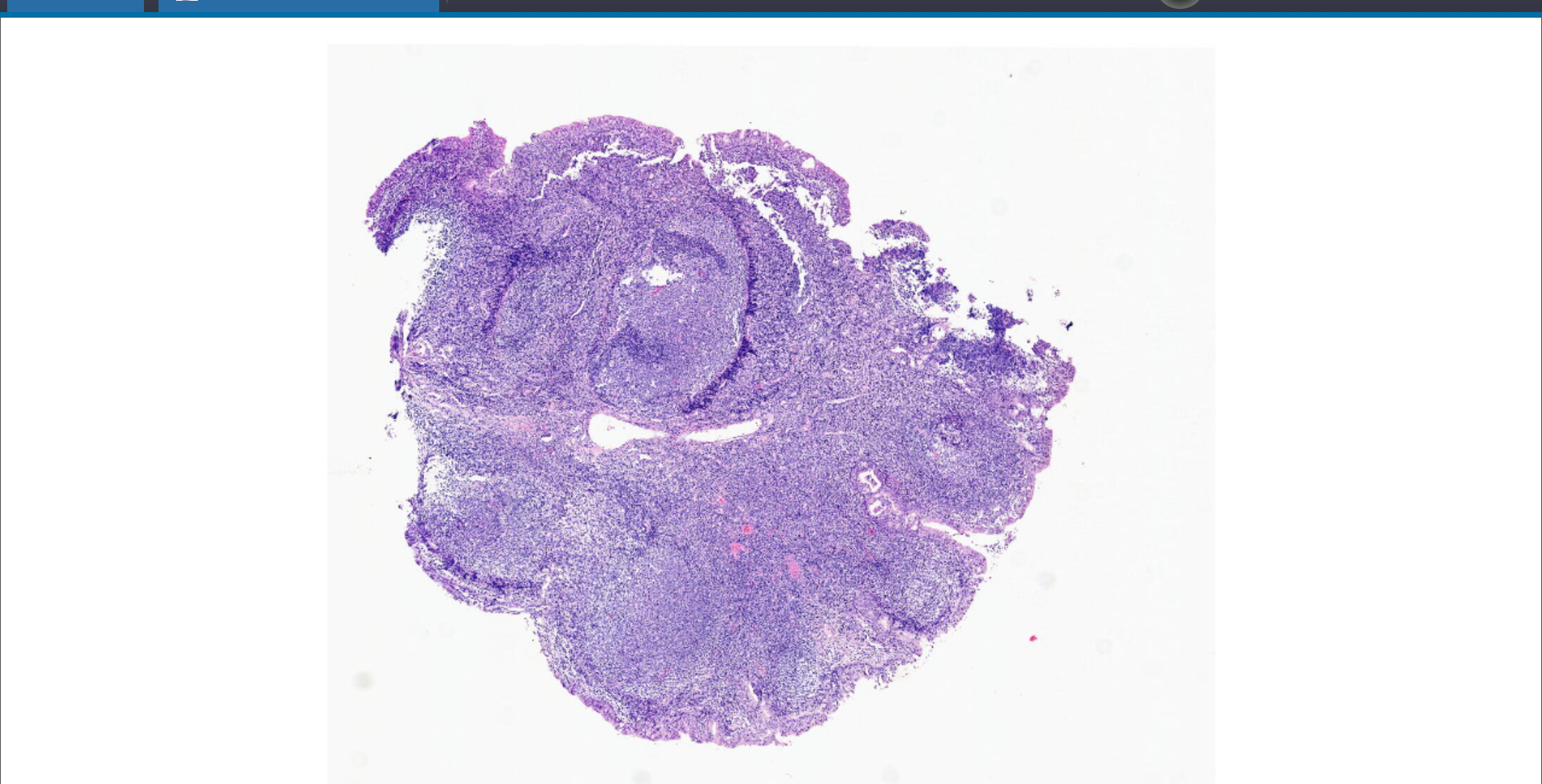

Supplement: Supplementary file 16 — Source data Fig. 1 [file 44321_2026_419_MOESM16_ESM.zip › Source data Fig.1/Fig 1B/202301989.jpg]

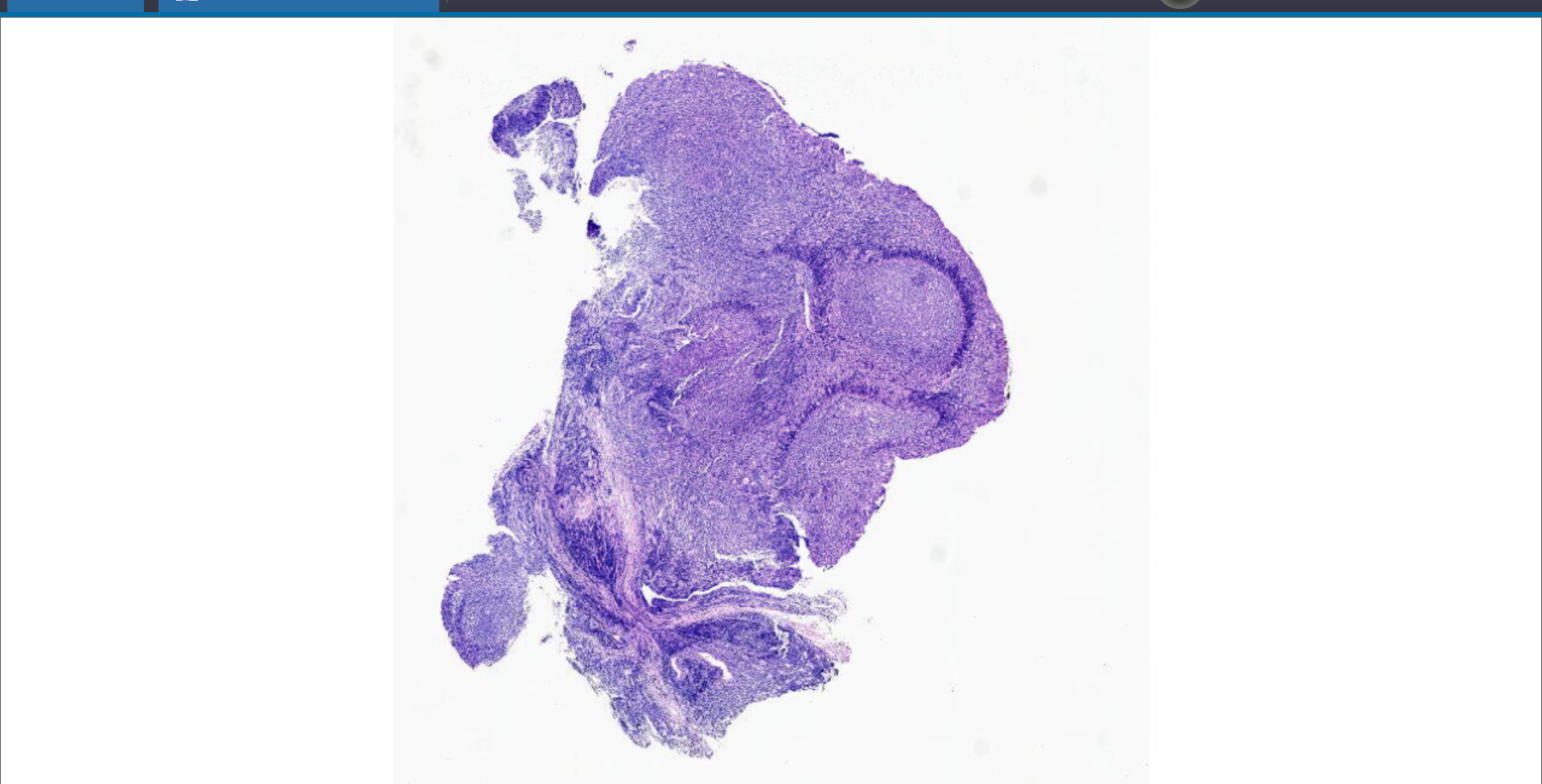

Supplement: Supplementary file 16 — Source data Fig. 1 [file 44321_2026_419_MOESM16_ESM.zip › Source data Fig.1/Fig 1B/202303465.jpg]

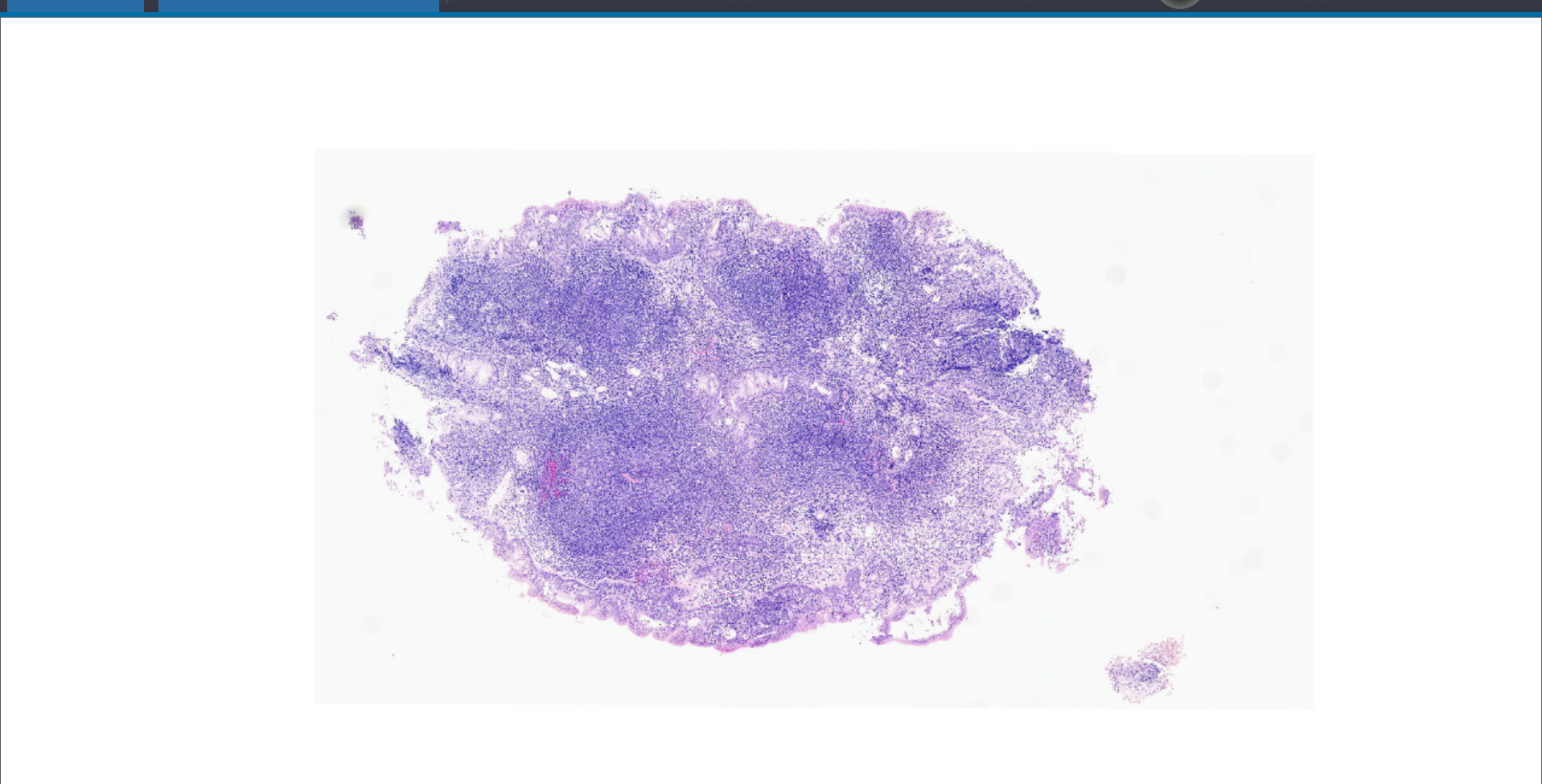

Supplement: Supplementary file 16 — Source data Fig. 1 [file 44321_2026_419_MOESM16_ESM.zip › Source data Fig.1/Fig 1B/202303495.jpg]

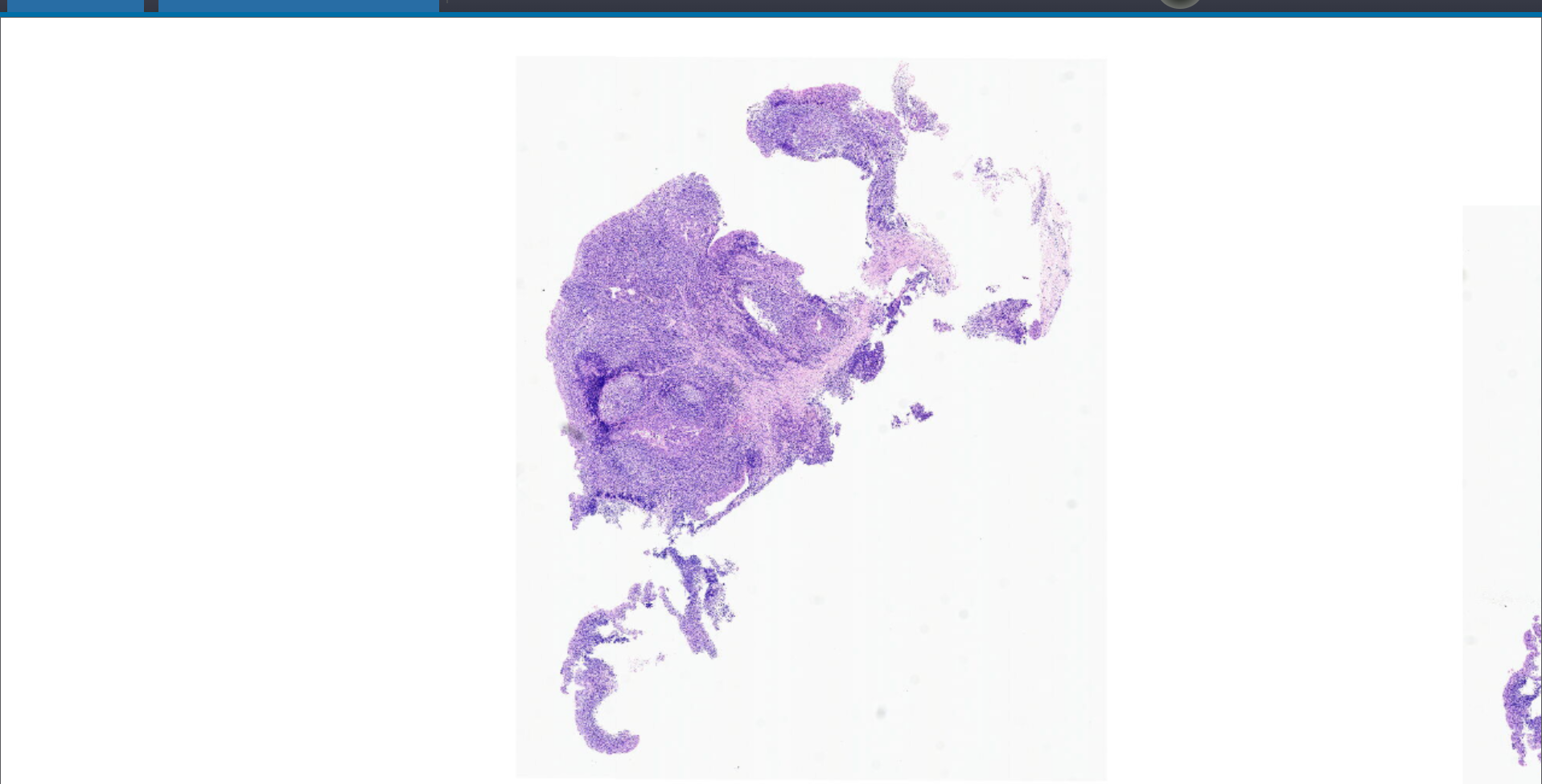

Supplement: Supplementary file 16 — Source data Fig. 1 [file 44321_2026_419_MOESM16_ESM.zip › Source data Fig.1/Fig 1B/202303496.jpg]

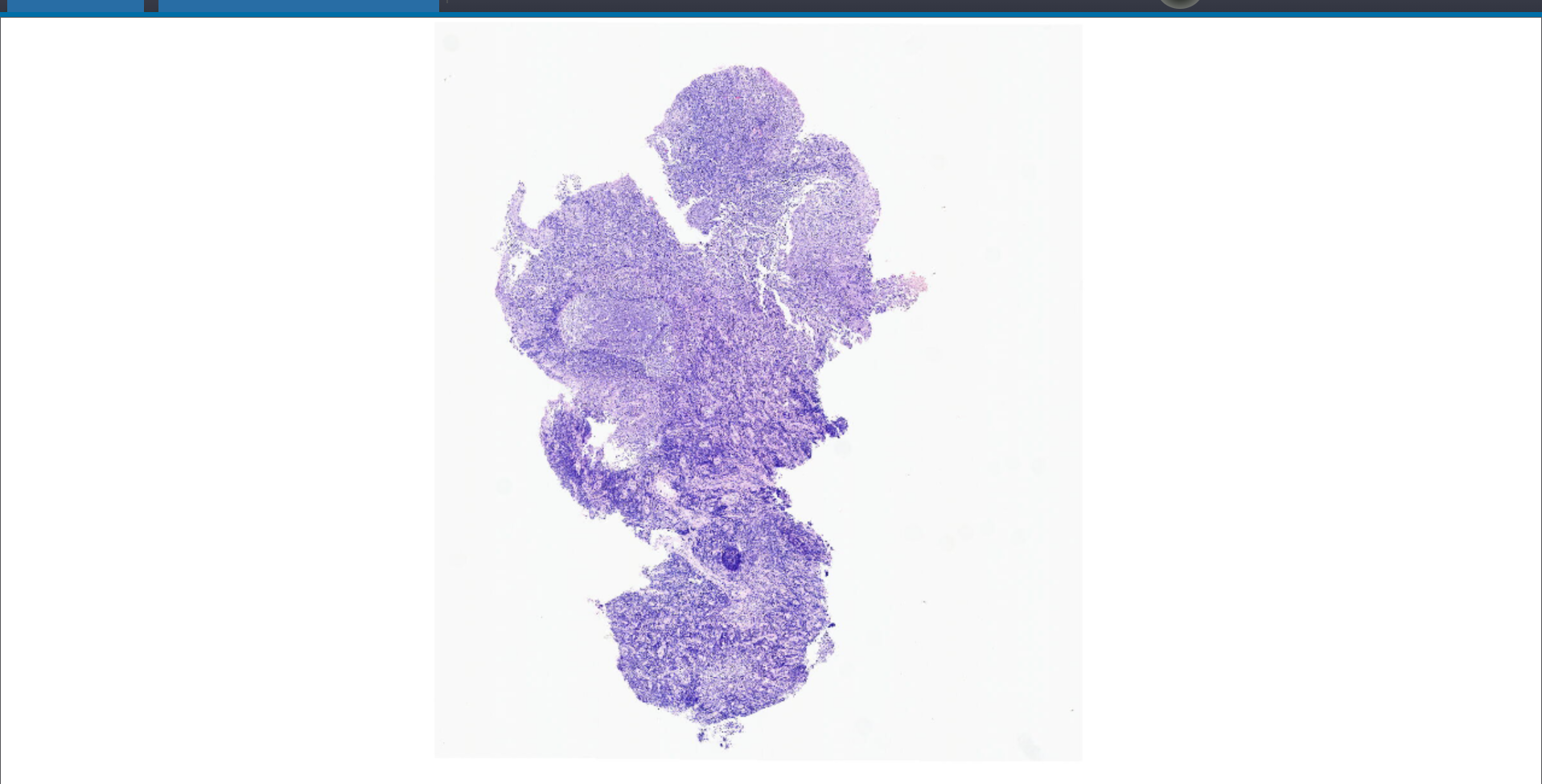

Supplement: Supplementary file 16 — Source data Fig. 1 [file 44321_2026_419_MOESM16_ESM.zip › Source data Fig.1/Fig 1B/202303497.jpg]

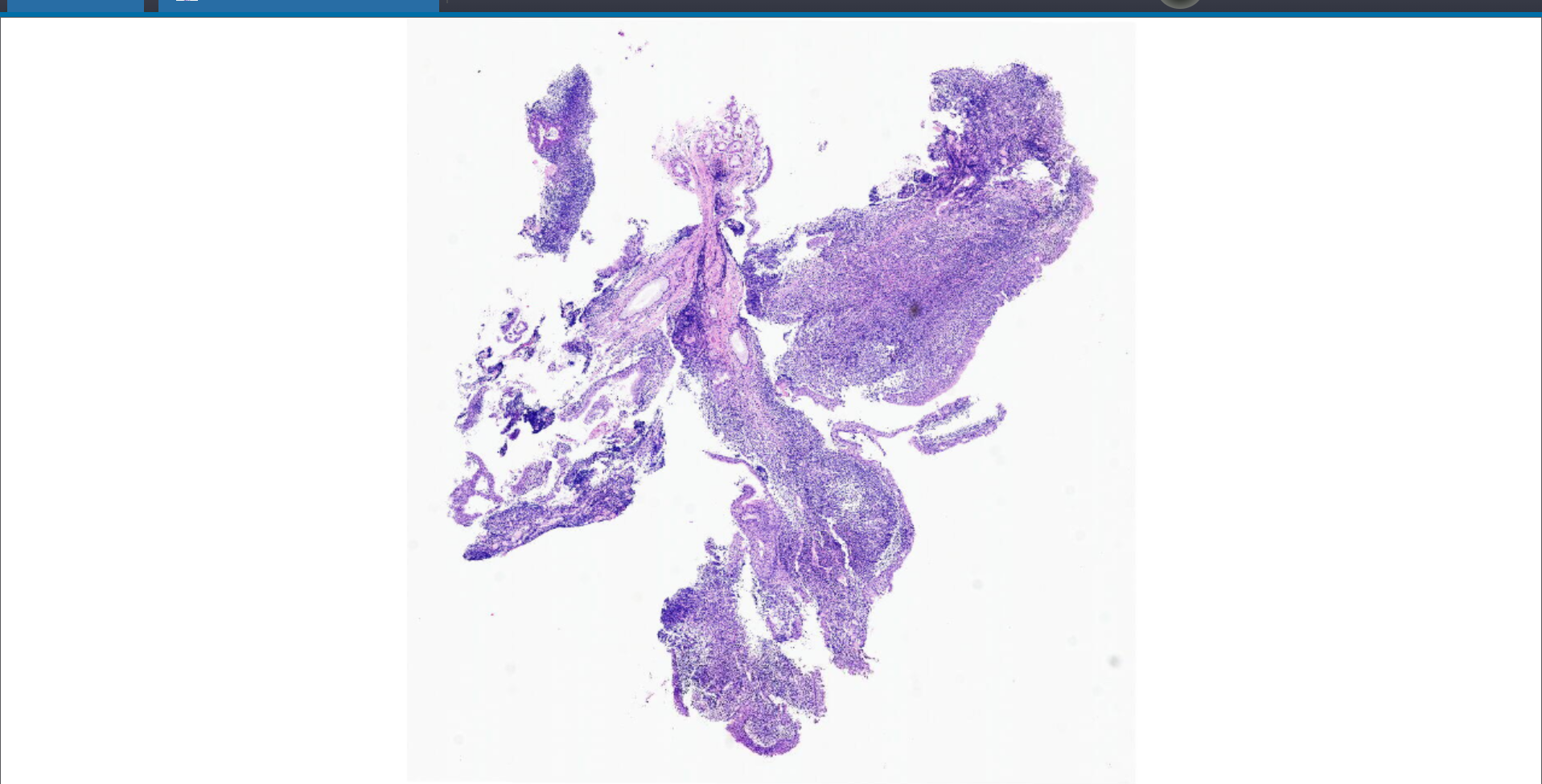

Supplement: Supplementary file 16 — Source data Fig. 1 [file 44321_2026_419_MOESM16_ESM.zip › Source data Fig.1/Fig 1B/202304213.jpg]

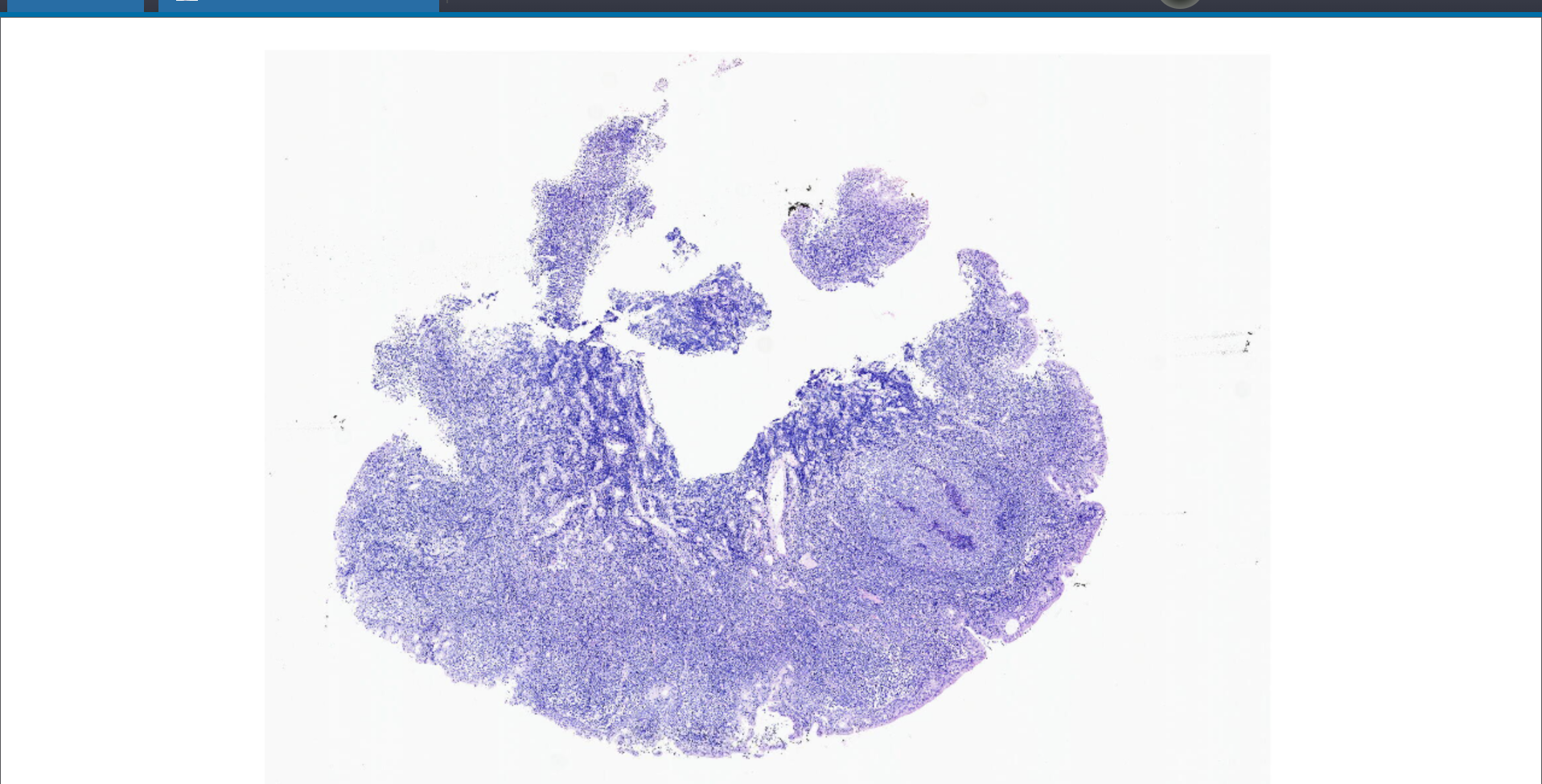

Supplement: Supplementary file 16 — Source data Fig. 1 [file 44321_2026_419_MOESM16_ESM.zip › Source data Fig.1/Fig 1B/202304235.jpg]

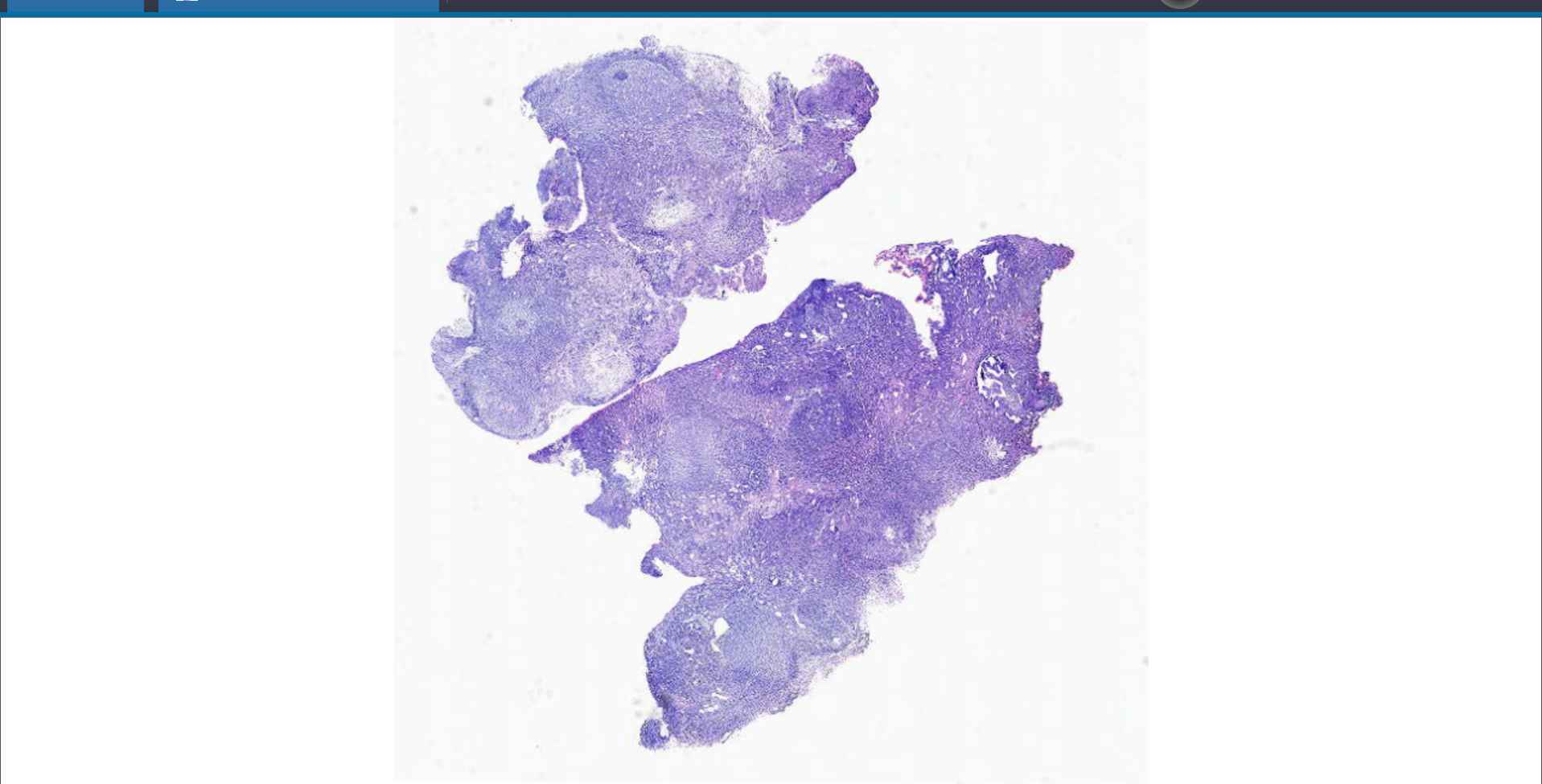

Supplement: Supplementary file 16 — Source data Fig. 1 [file 44321_2026_419_MOESM16_ESM.zip › Source data Fig.1/Fig 1B/202305889.jpg]

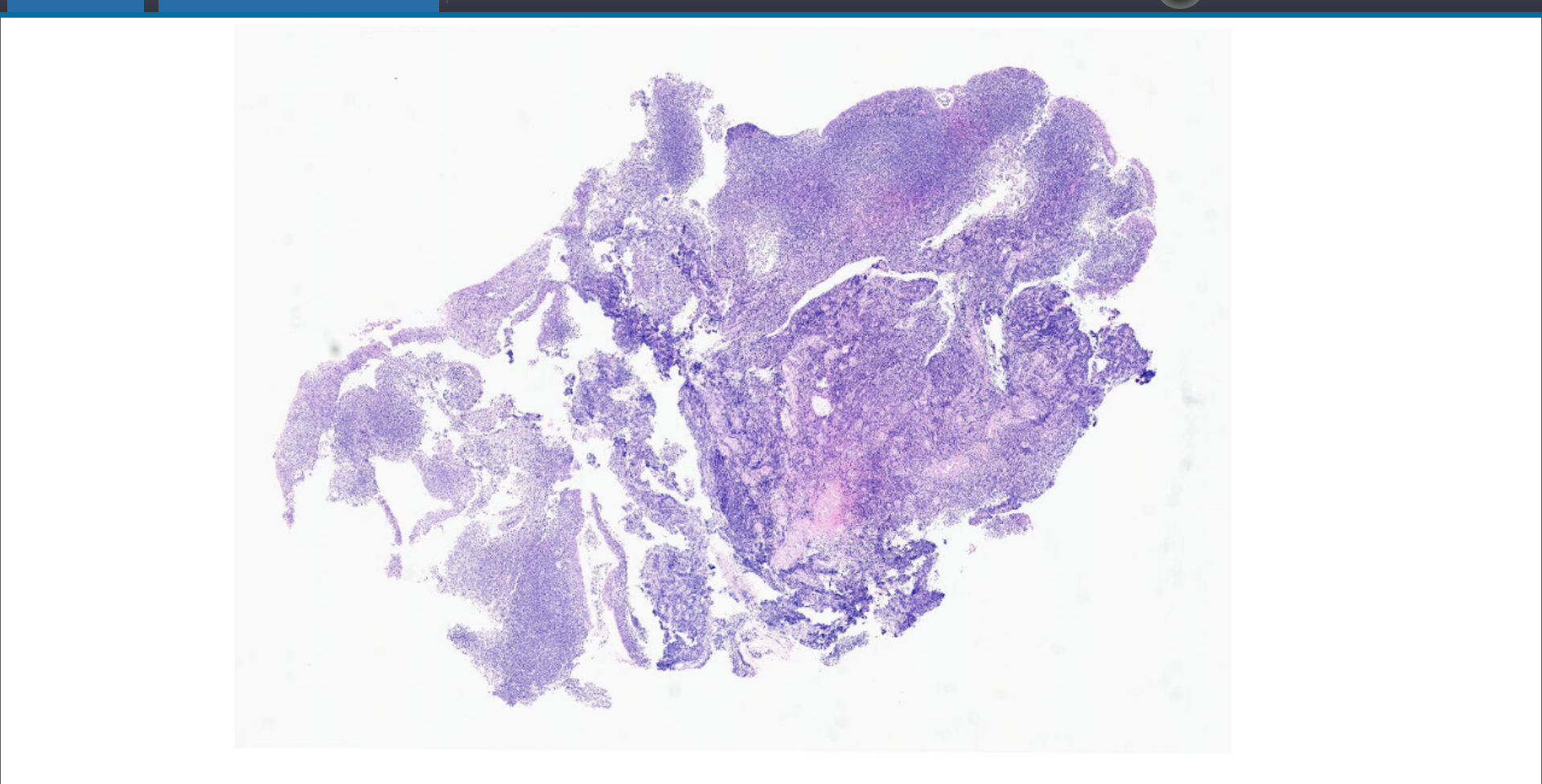

Supplement: Supplementary file 16 — Source data Fig. 1 [file 44321_2026_419_MOESM16_ESM.zip › Source data Fig.1/Fig 1B/202306537.jpg]

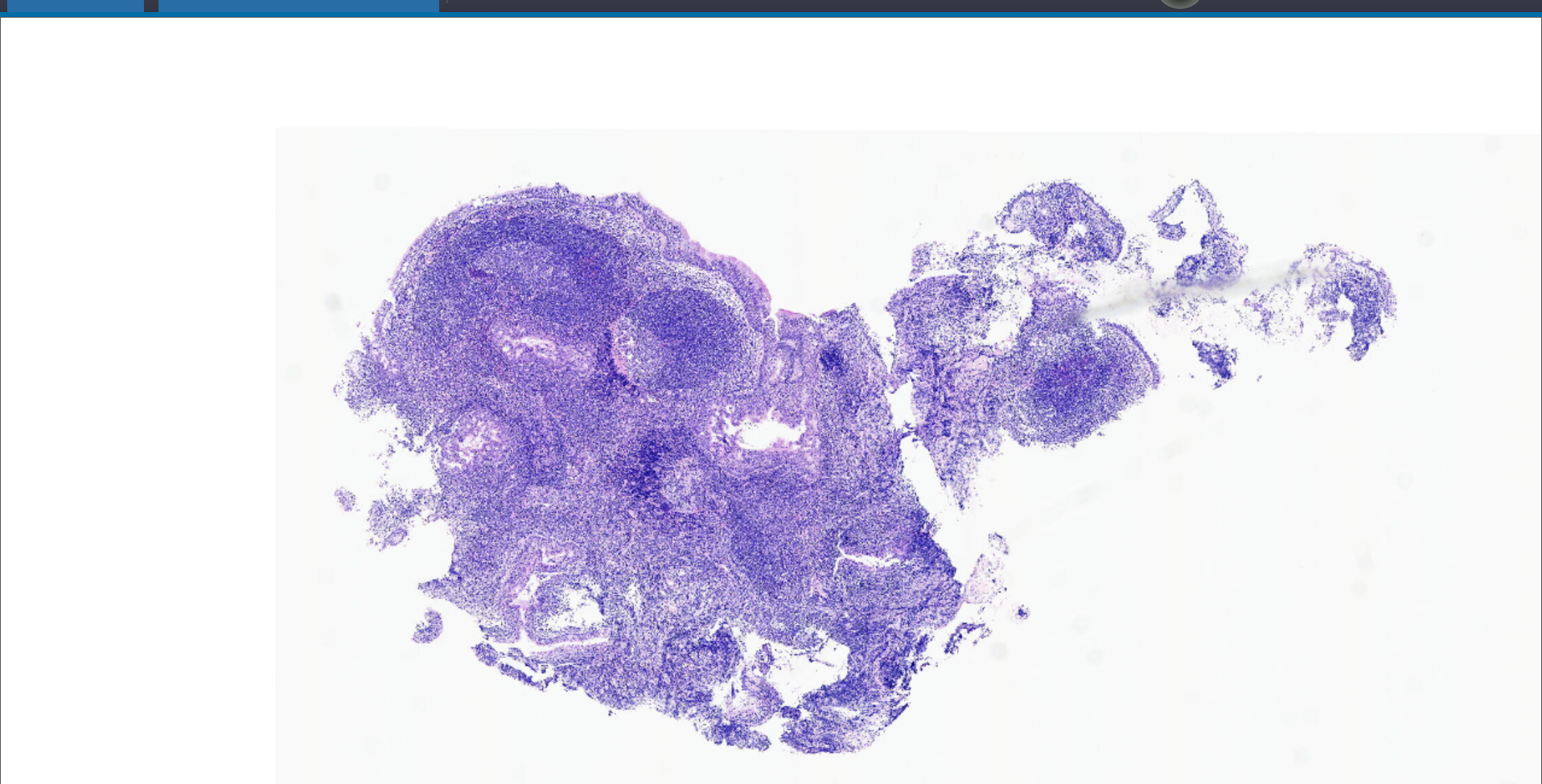

Supplement: Supplementary file 16 — Source data Fig. 1 [file 44321_2026_419_MOESM16_ESM.zip › Source data Fig.1/Fig 1B/202307864.jpg]

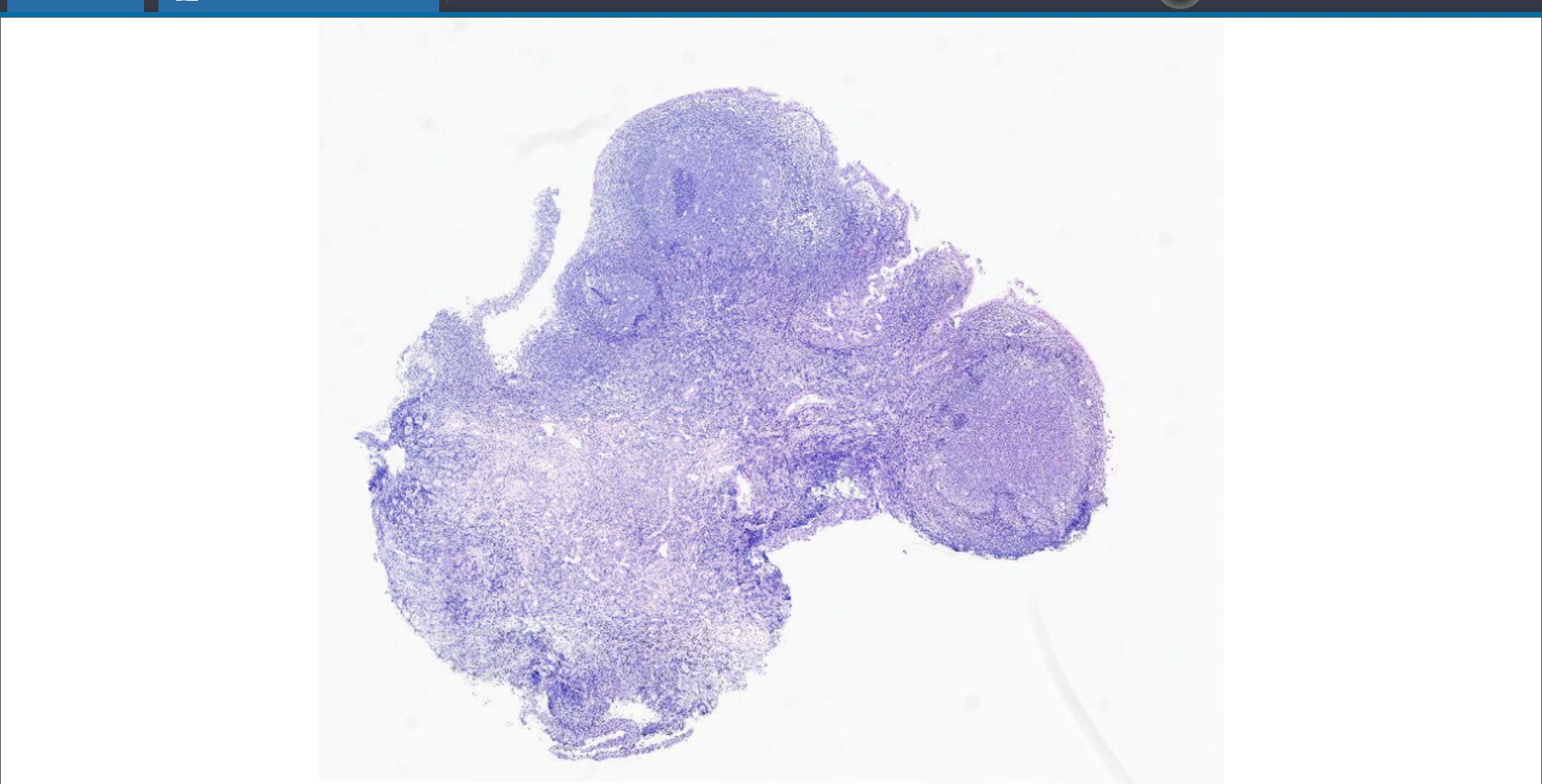

Supplement: Supplementary file 16 — Source data Fig. 1 [file 44321_2026_419_MOESM16_ESM.zip › Source data Fig.1/Fig 1B/202308201.jpg]

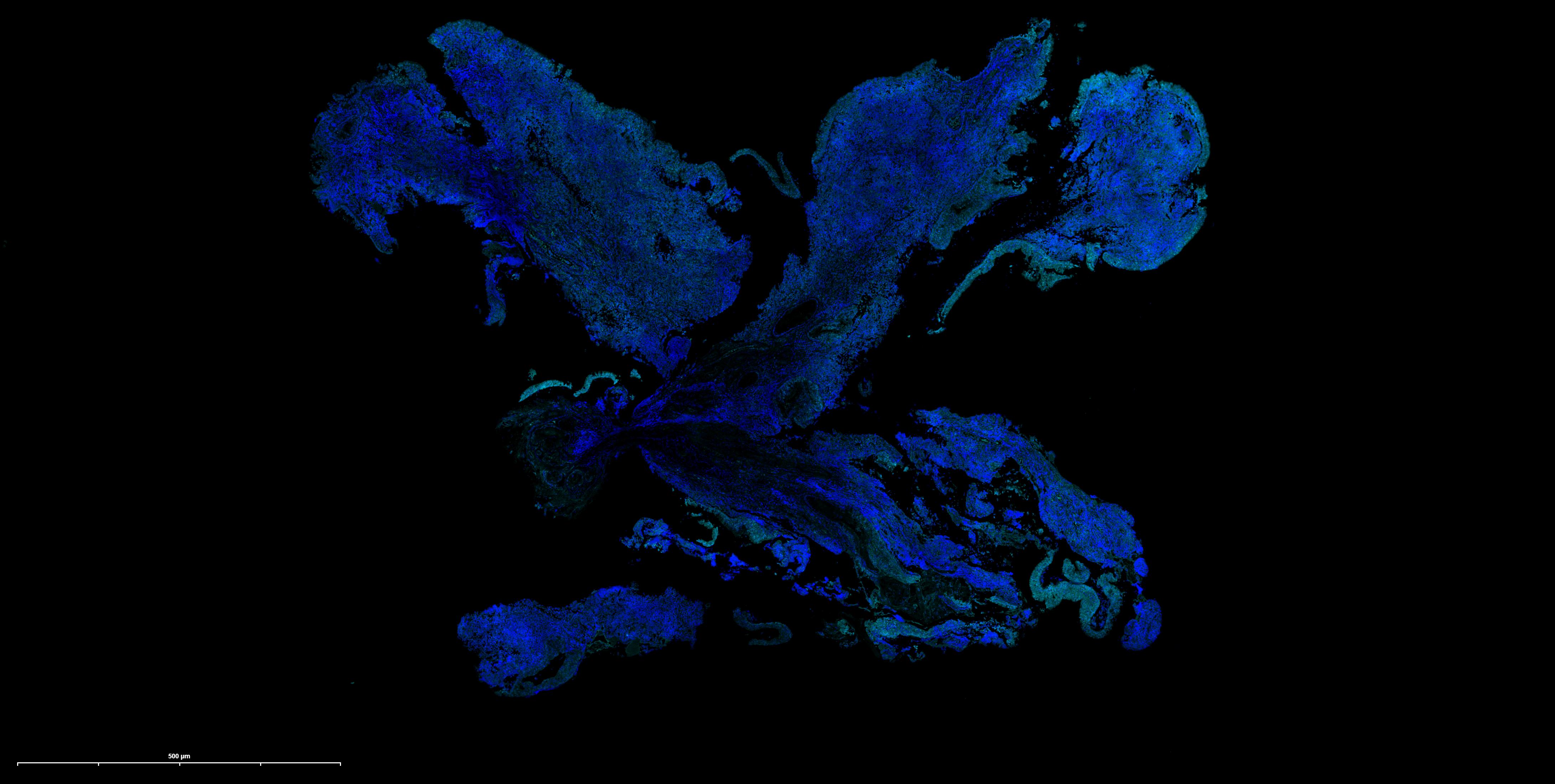

Supplement: Supplementary file 16 — Source data Fig. 1 [file 44321_2026_419_MOESM16_ESM.zip › Source data Fig.1/Fig 1J/HIF1α-Mild1.jpg]

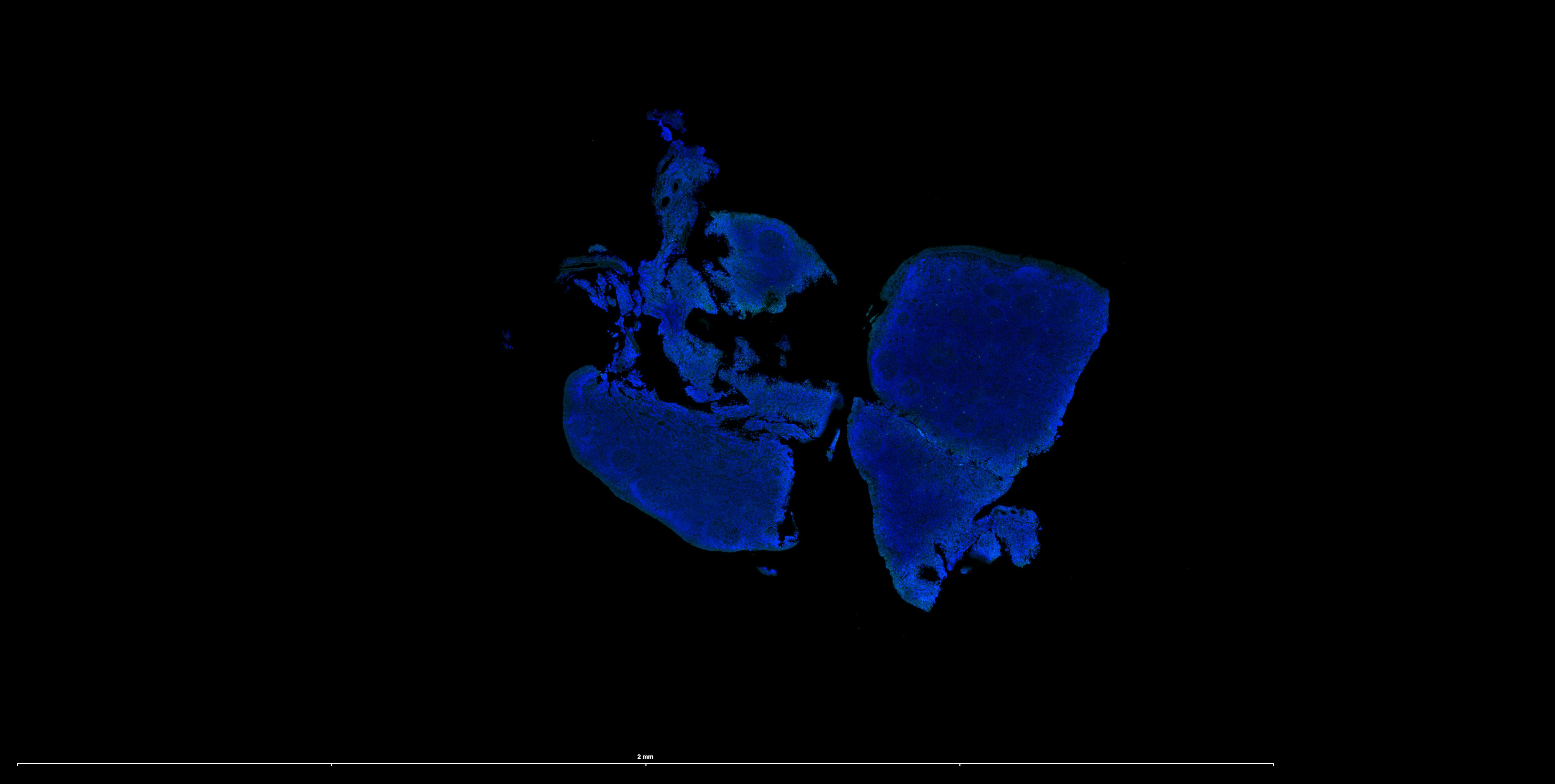

Supplement: Supplementary file 16 — Source data Fig. 1 [file 44321_2026_419_MOESM16_ESM.zip › Source data Fig.1/Fig 1J/HIF1α-Mild2.jpg]

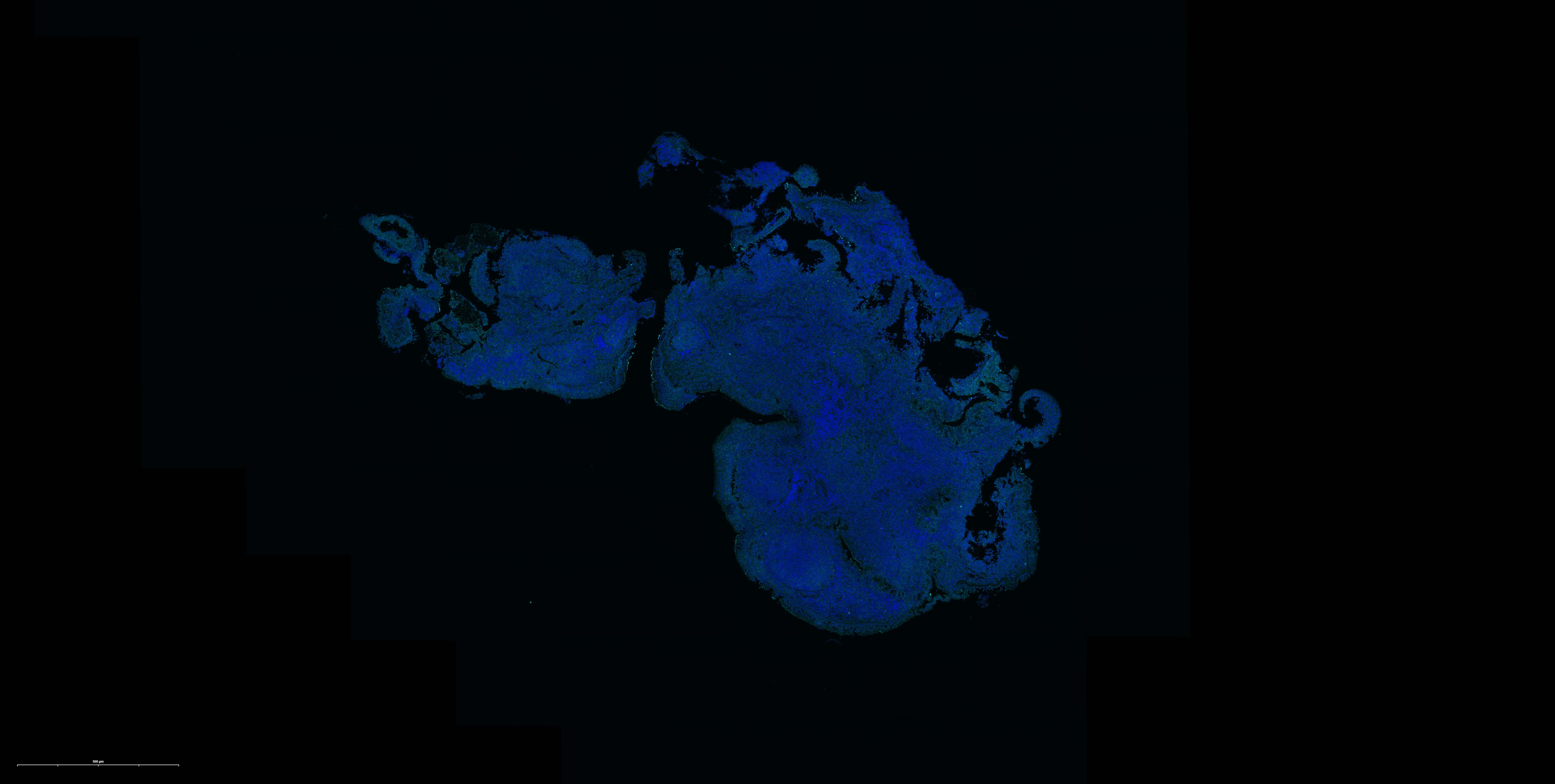

Supplement: Supplementary file 16 — Source data Fig. 1 [file 44321_2026_419_MOESM16_ESM.zip › Source data Fig.1/Fig 1J/HIF1α-Severe1.jpg]

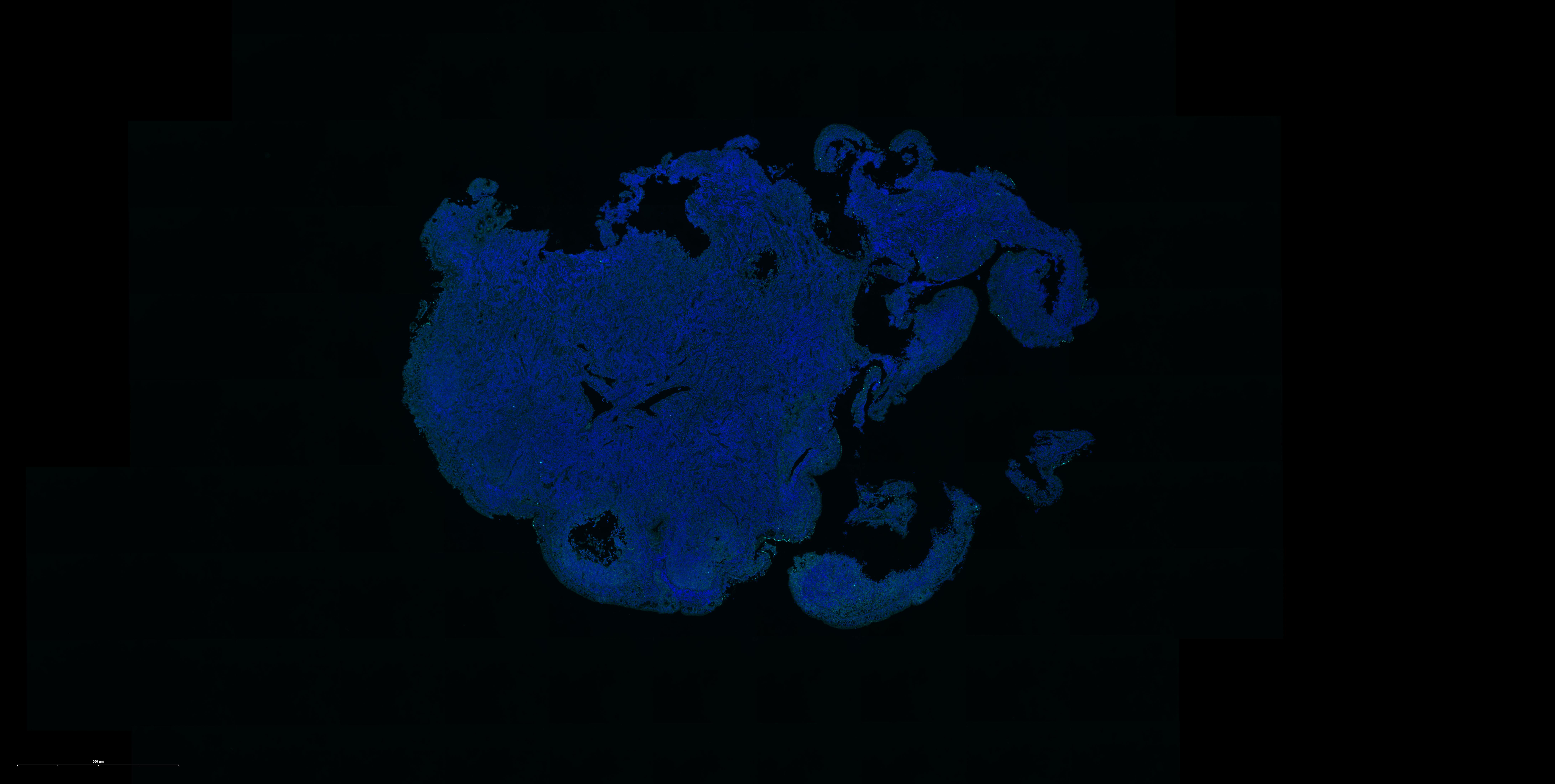

Supplement: Supplementary file 16 — Source data Fig. 1 [file 44321_2026_419_MOESM16_ESM.zip › Source data Fig.1/Fig 1J/HIF1α-Severe2.jpg]

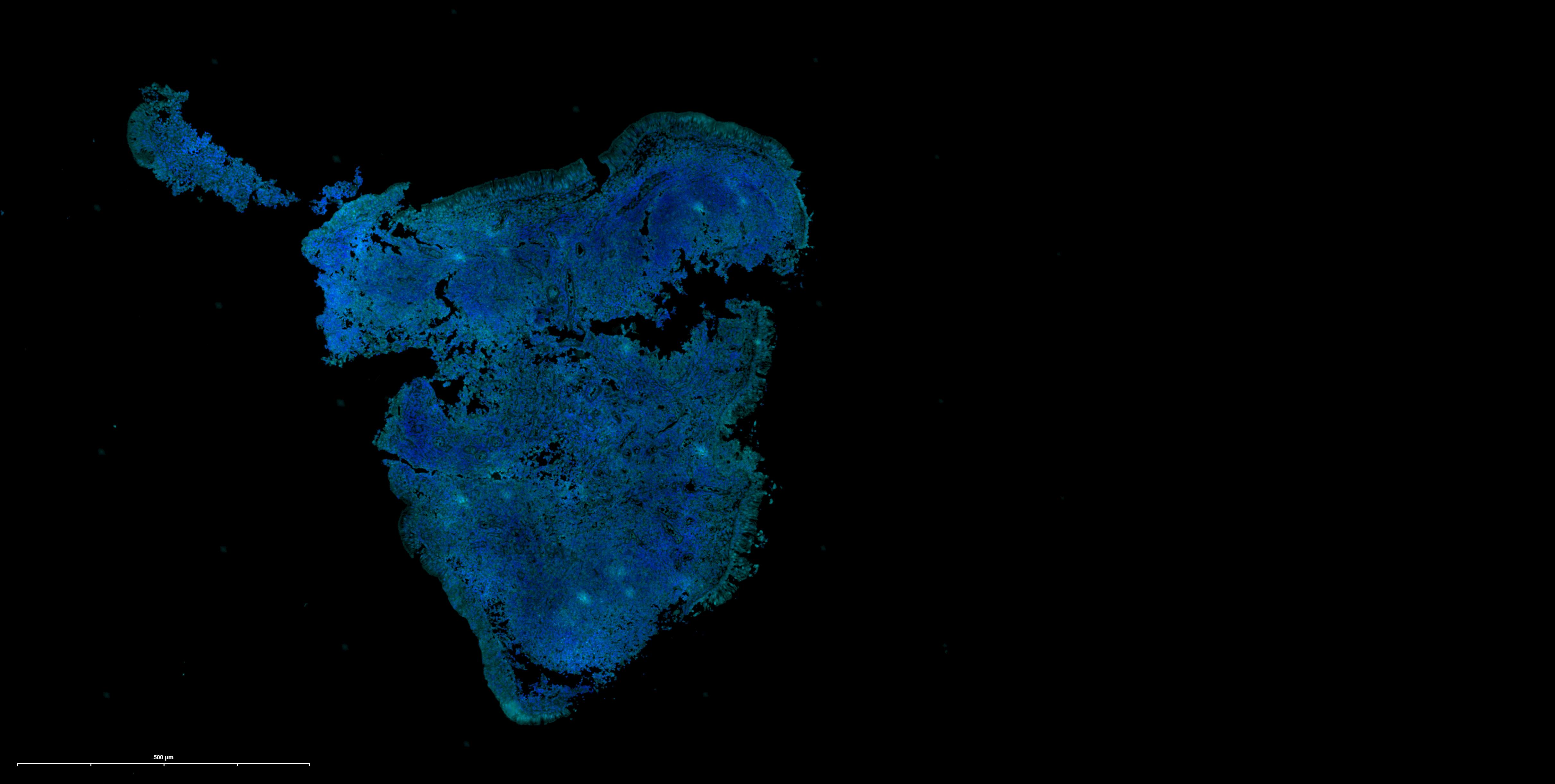

Supplement: Supplementary file 16 — Source data Fig. 1 [file 44321_2026_419_MOESM16_ESM.zip › Source data Fig.1/Fig 1J/HIF1α-Severe3.jpg]

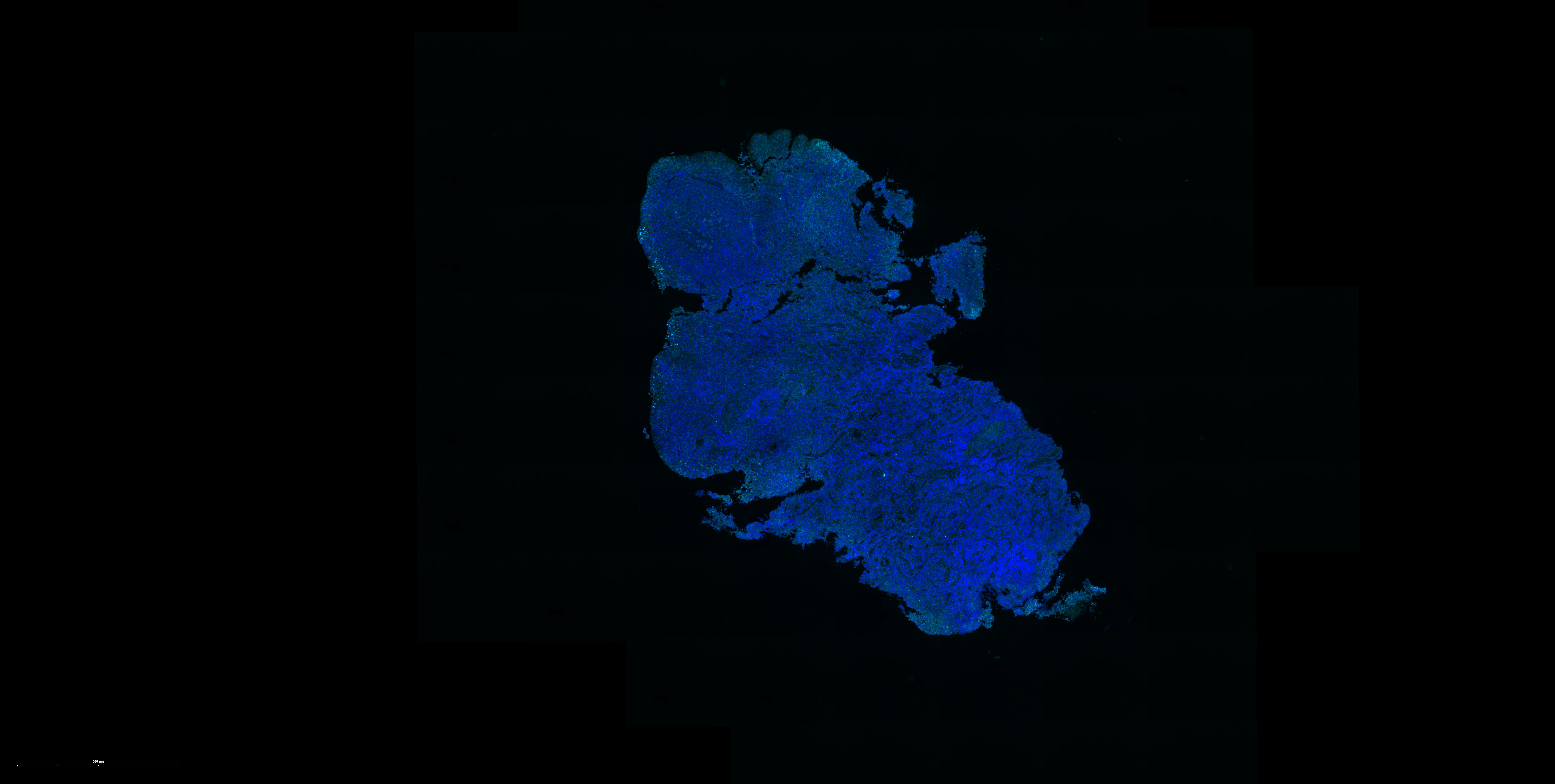

Supplement: Supplementary file 16 — Source data Fig. 1 [file 44321_2026_419_MOESM16_ESM.zip › Source data Fig.1/Fig 1J/HIF1α-Control1.jpg]

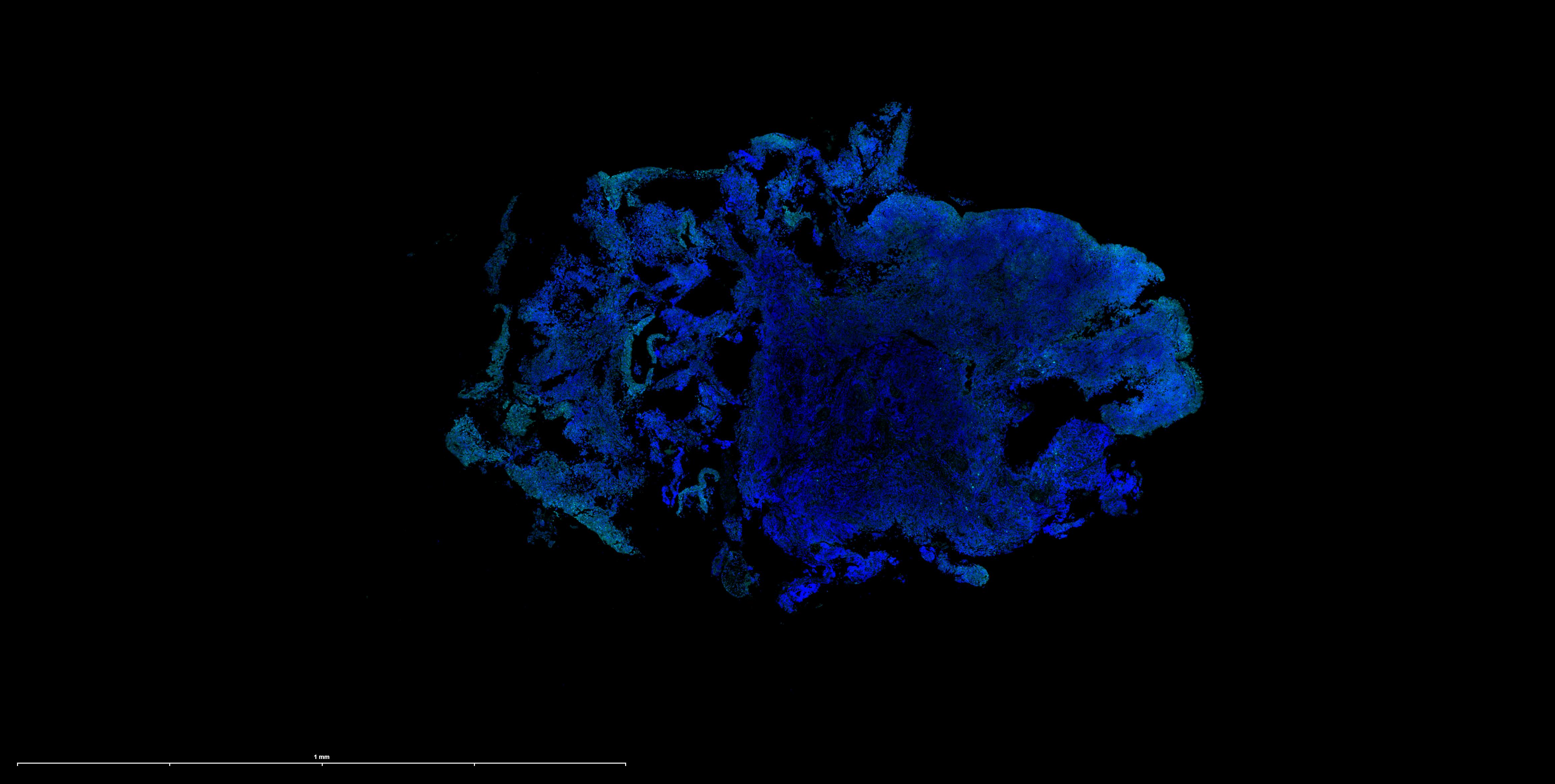

Supplement: Supplementary file 16 — Source data Fig. 1 [file 44321_2026_419_MOESM16_ESM.zip › Source data Fig.1/Fig 1J/HIF1α-Control2.jpg]

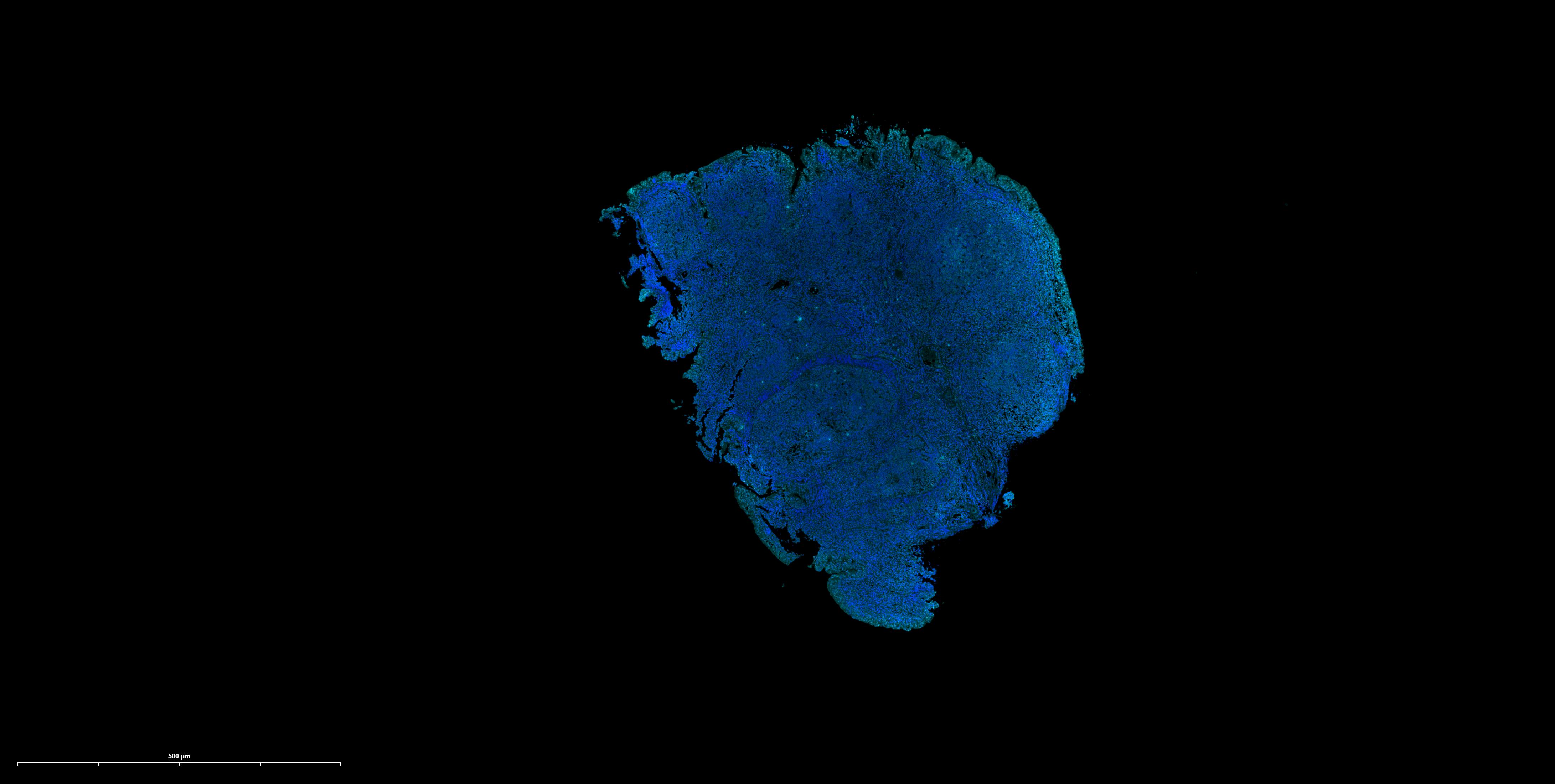

Supplement: Supplementary file 16 — Source data Fig. 1 [file 44321_2026_419_MOESM16_ESM.zip › Source data Fig.1/Fig 1J/HIF1α-Control3.jpg]

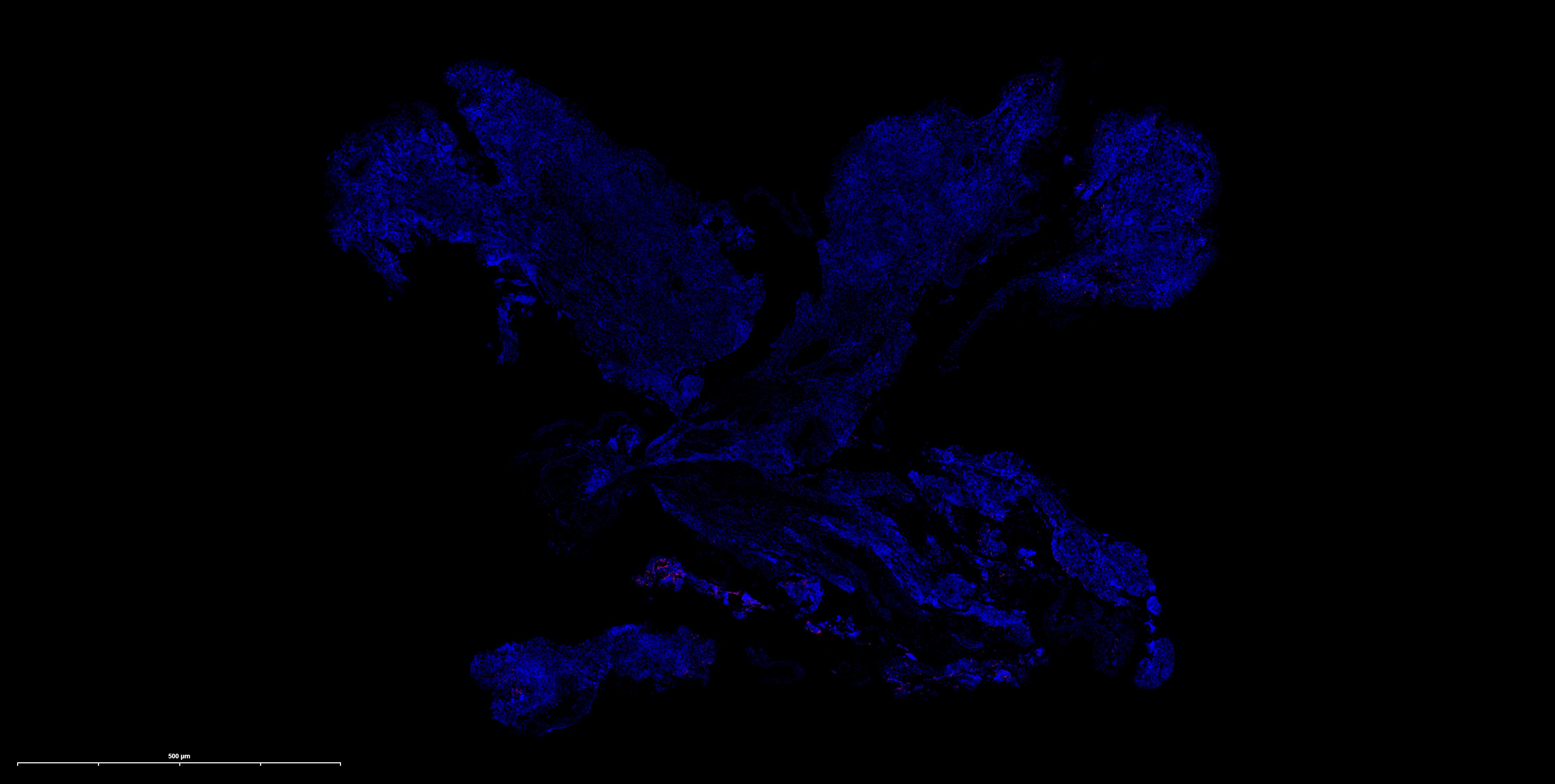

Supplement: Supplementary file 17 — Source data Fig. 2 [file 44321_2026_419_MOESM17_ESM.zip › Source data Fig.2/Fig 2B/Ki67-Mild1.jpg]

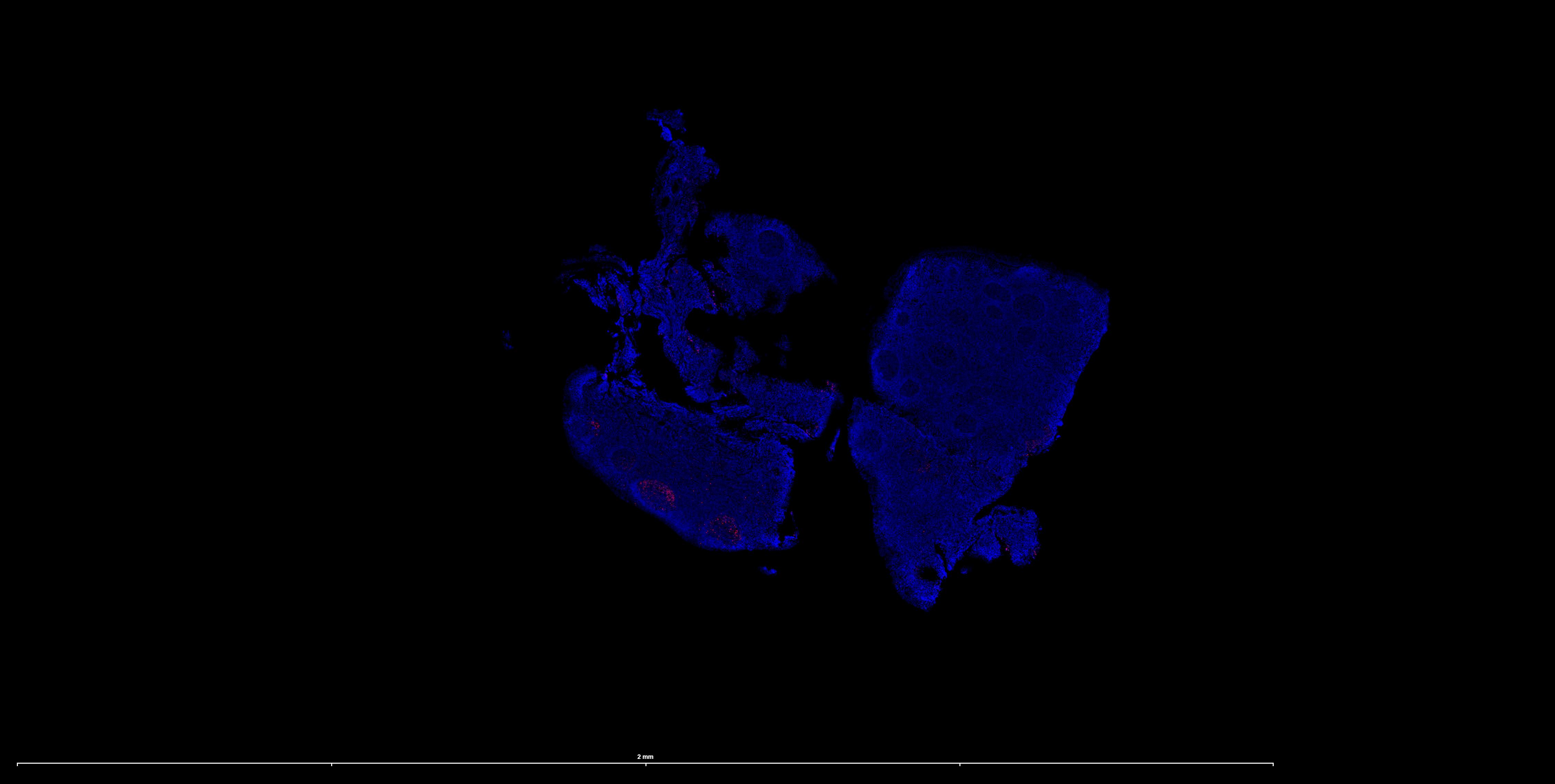

Supplement: Supplementary file 17 — Source data Fig. 2 [file 44321_2026_419_MOESM17_ESM.zip › Source data Fig.2/Fig 2B/Ki67-Mild2.jpg]

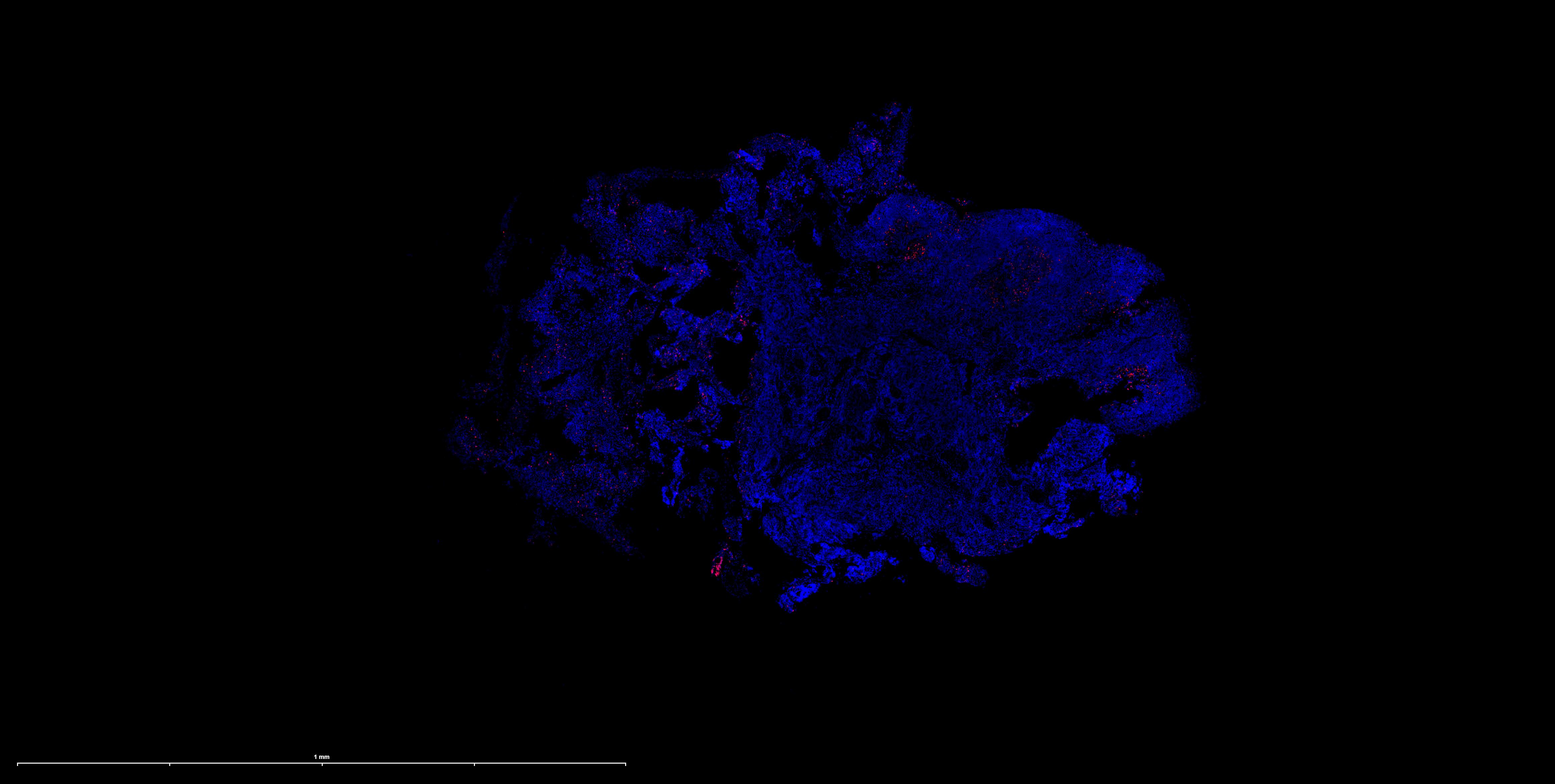

Supplement: Supplementary file 17 — Source data Fig. 2 [file 44321_2026_419_MOESM17_ESM.zip › Source data Fig.2/Fig 2B/Ki67-Control2.jpg]

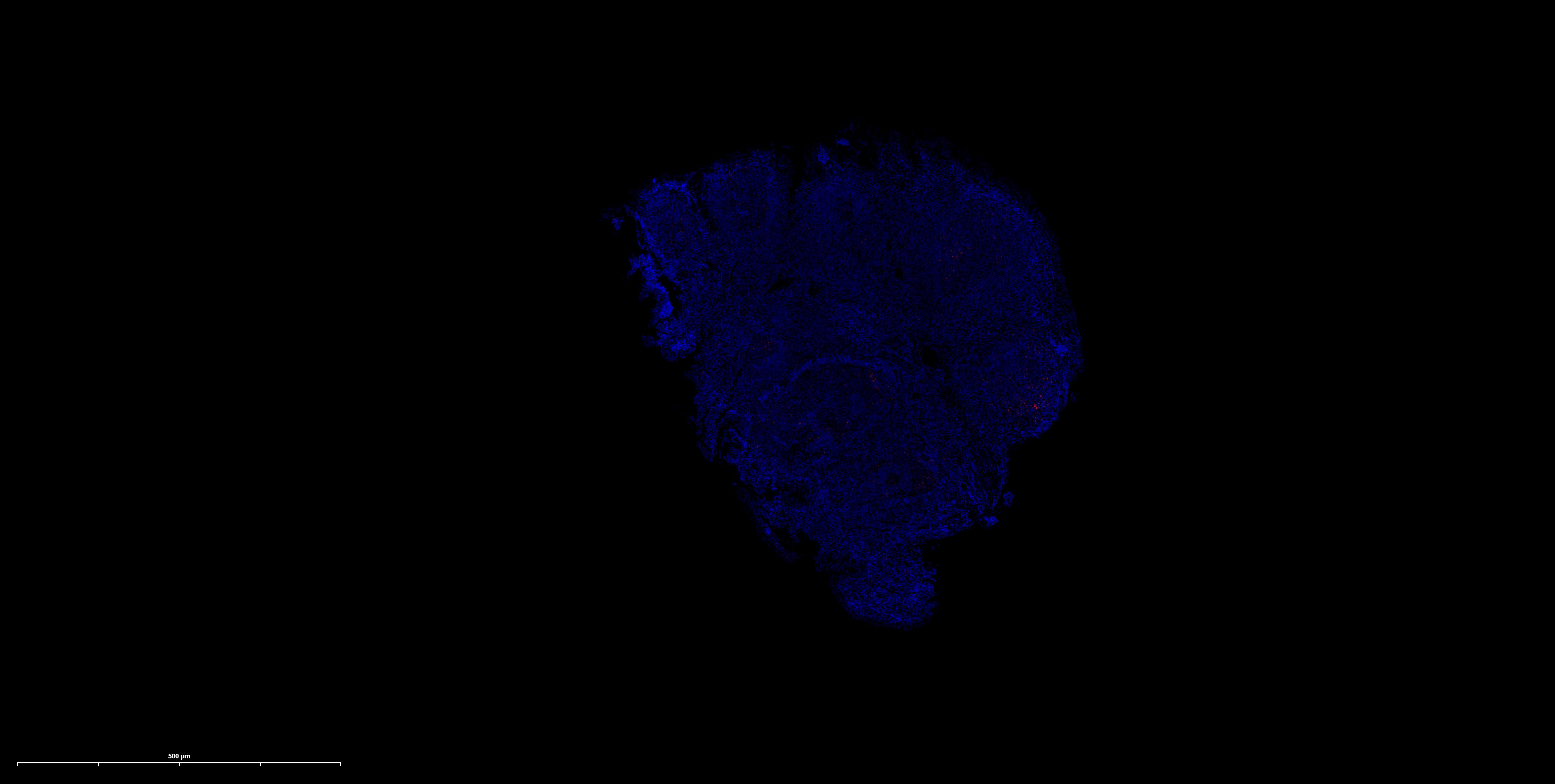

Supplement: Supplementary file 17 — Source data Fig. 2 [file 44321_2026_419_MOESM17_ESM.zip › Source data Fig.2/Fig 2B/Ki67-Control3.jpg]

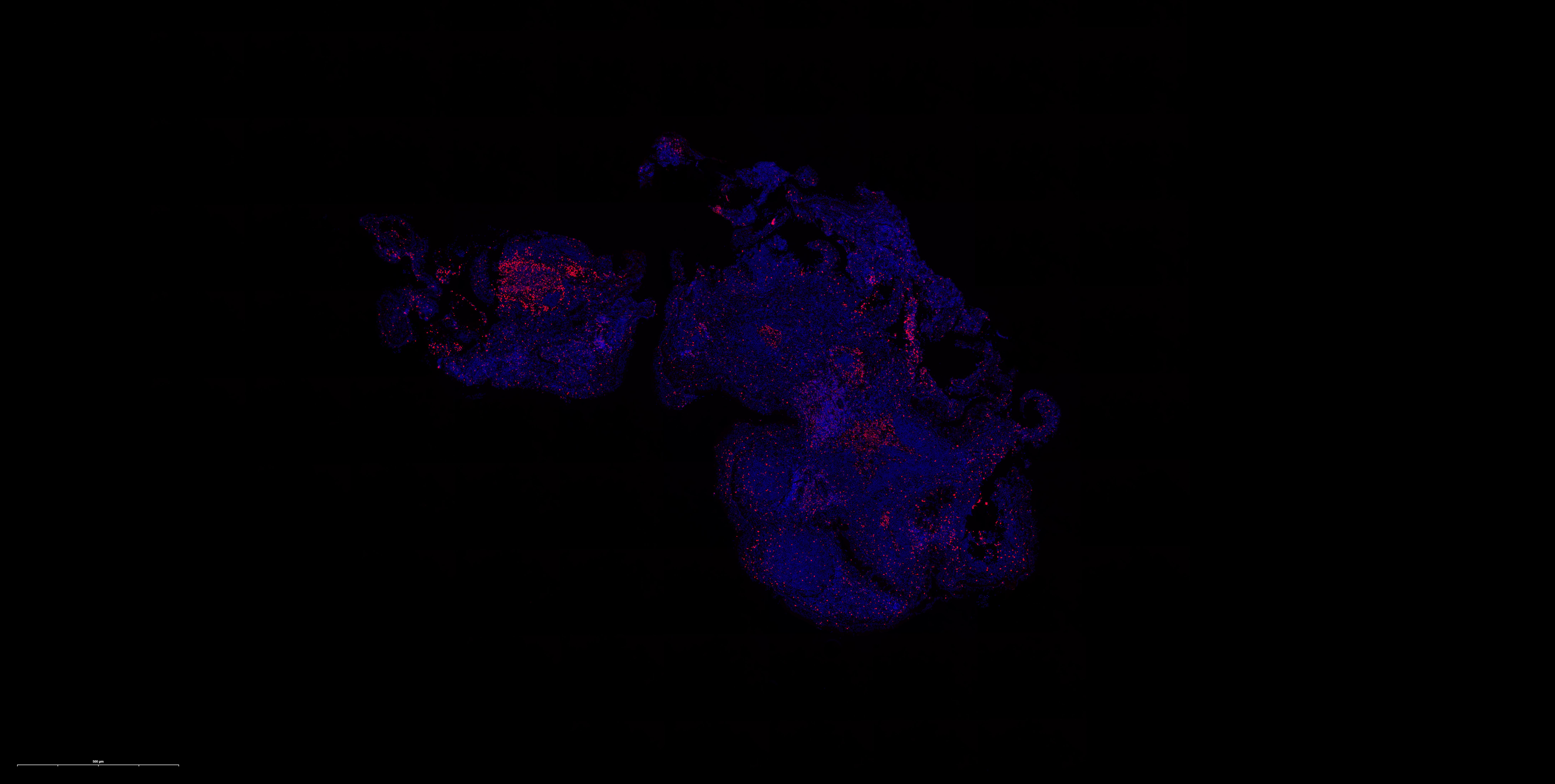

Supplement: Supplementary file 17 — Source data Fig. 2 [file 44321_2026_419_MOESM17_ESM.zip › Source data Fig.2/Fig 2B/Ki67-Severe1.jpg]

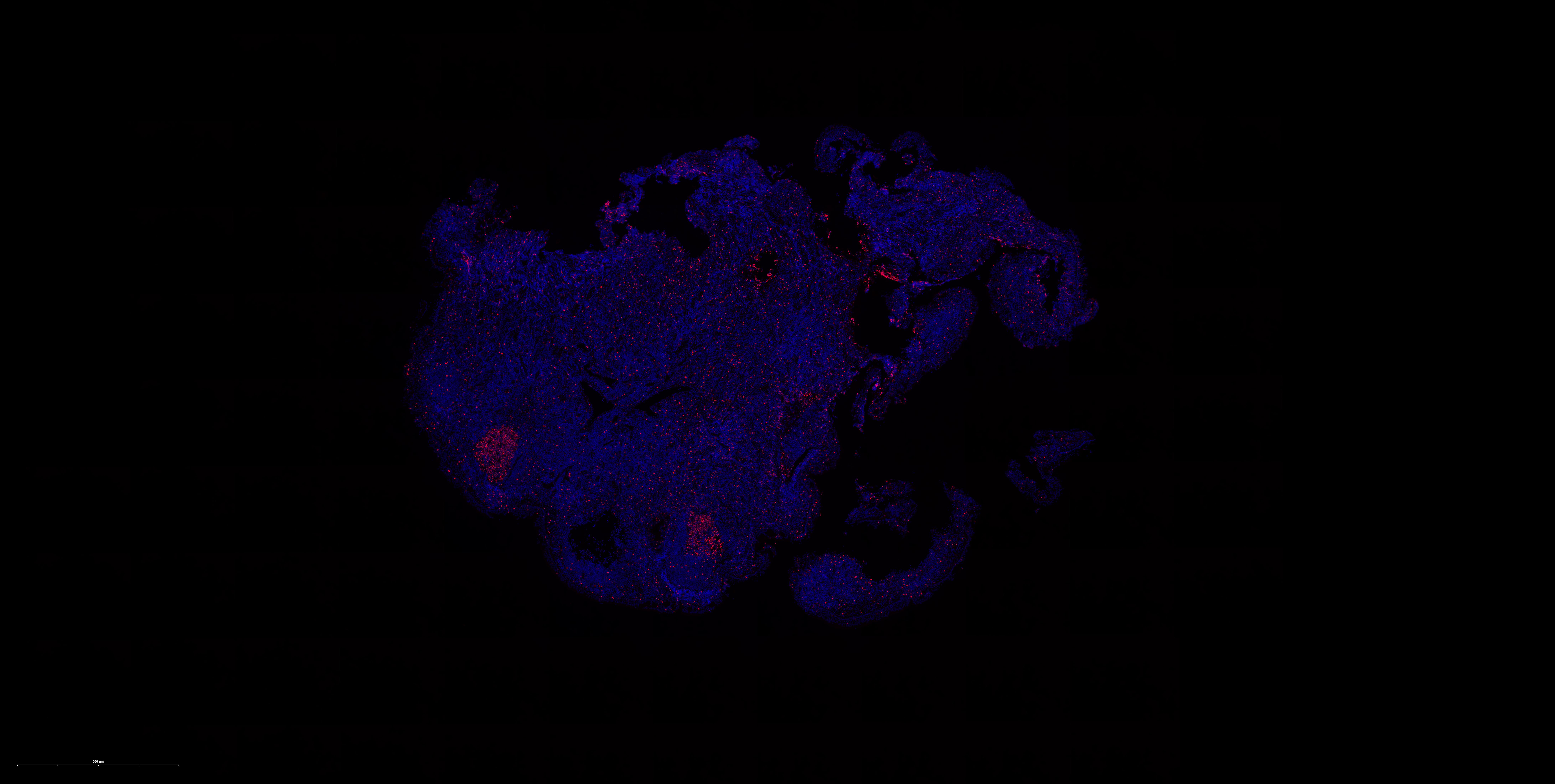

Supplement: Supplementary file 17 — Source data Fig. 2 [file 44321_2026_419_MOESM17_ESM.zip › Source data Fig.2/Fig 2B/Ki67-Severe2.jpg]

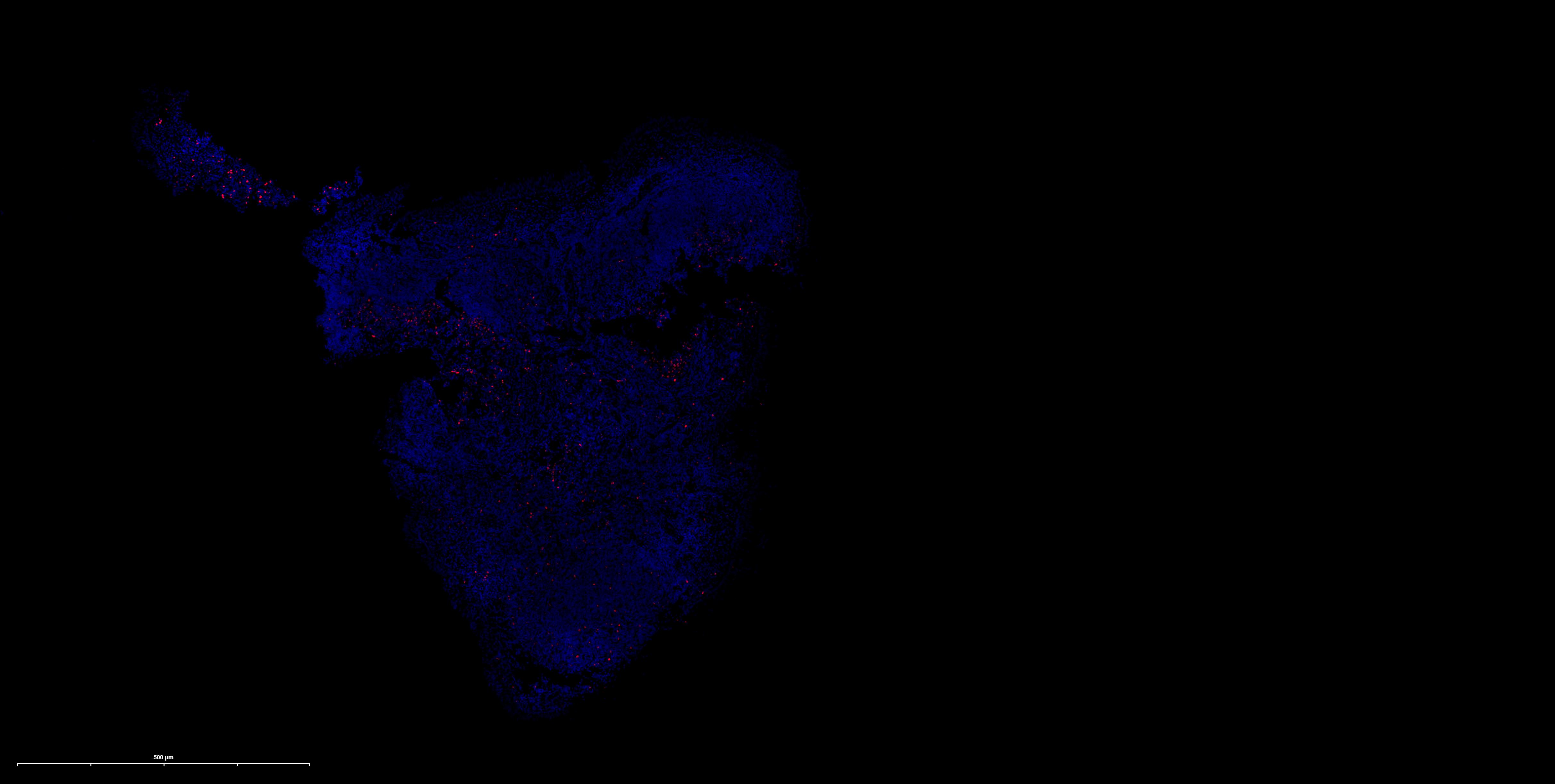

Supplement: Supplementary file 17 — Source data Fig. 2 [file 44321_2026_419_MOESM17_ESM.zip › Source data Fig.2/Fig 2B/Ki67-Severe3.jpg]

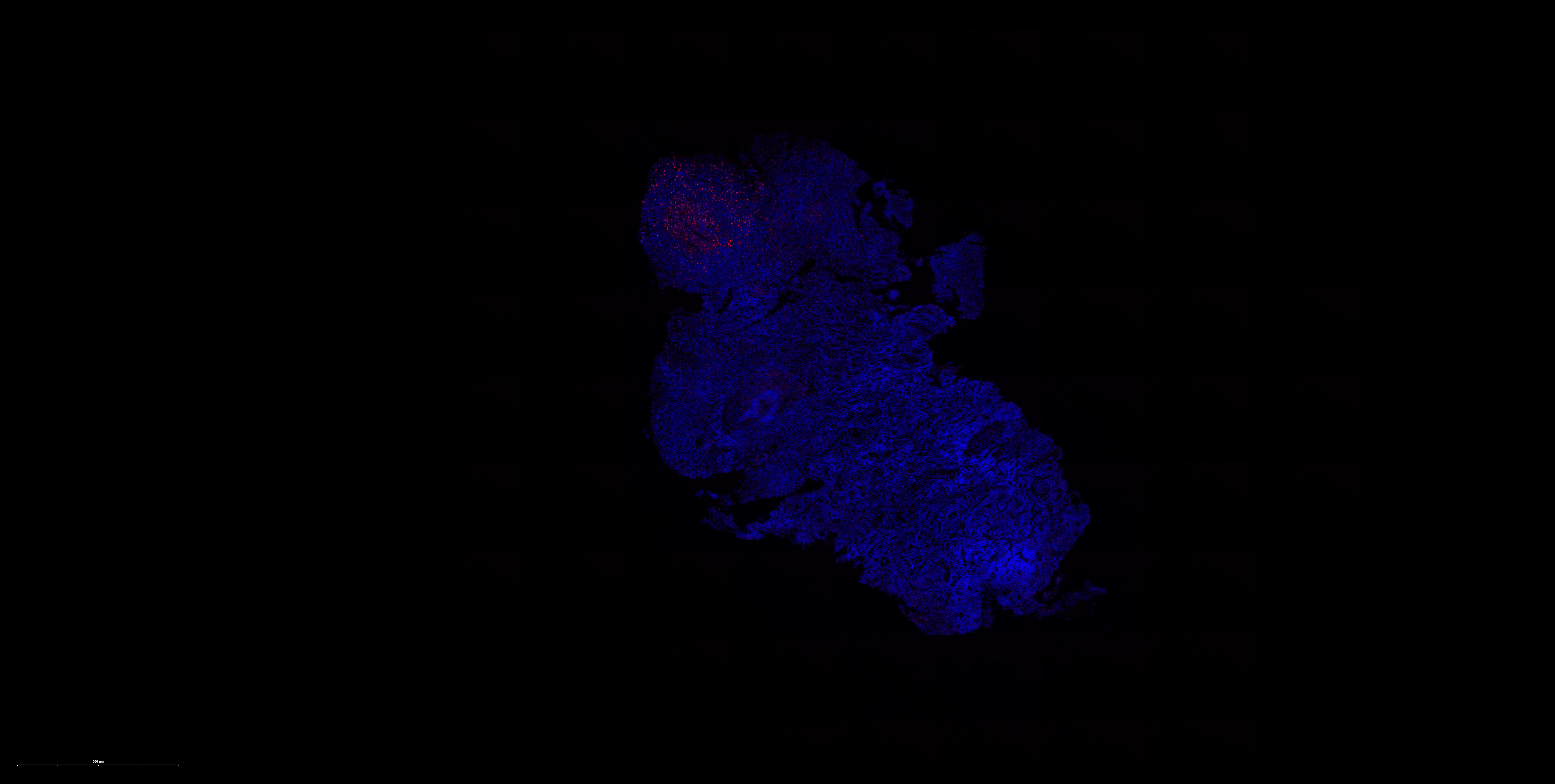

Supplement: Supplementary file 17 — Source data Fig. 2 [file 44321_2026_419_MOESM17_ESM.zip › Source data Fig.2/Fig 2B/Ki67-Control1.jpg]

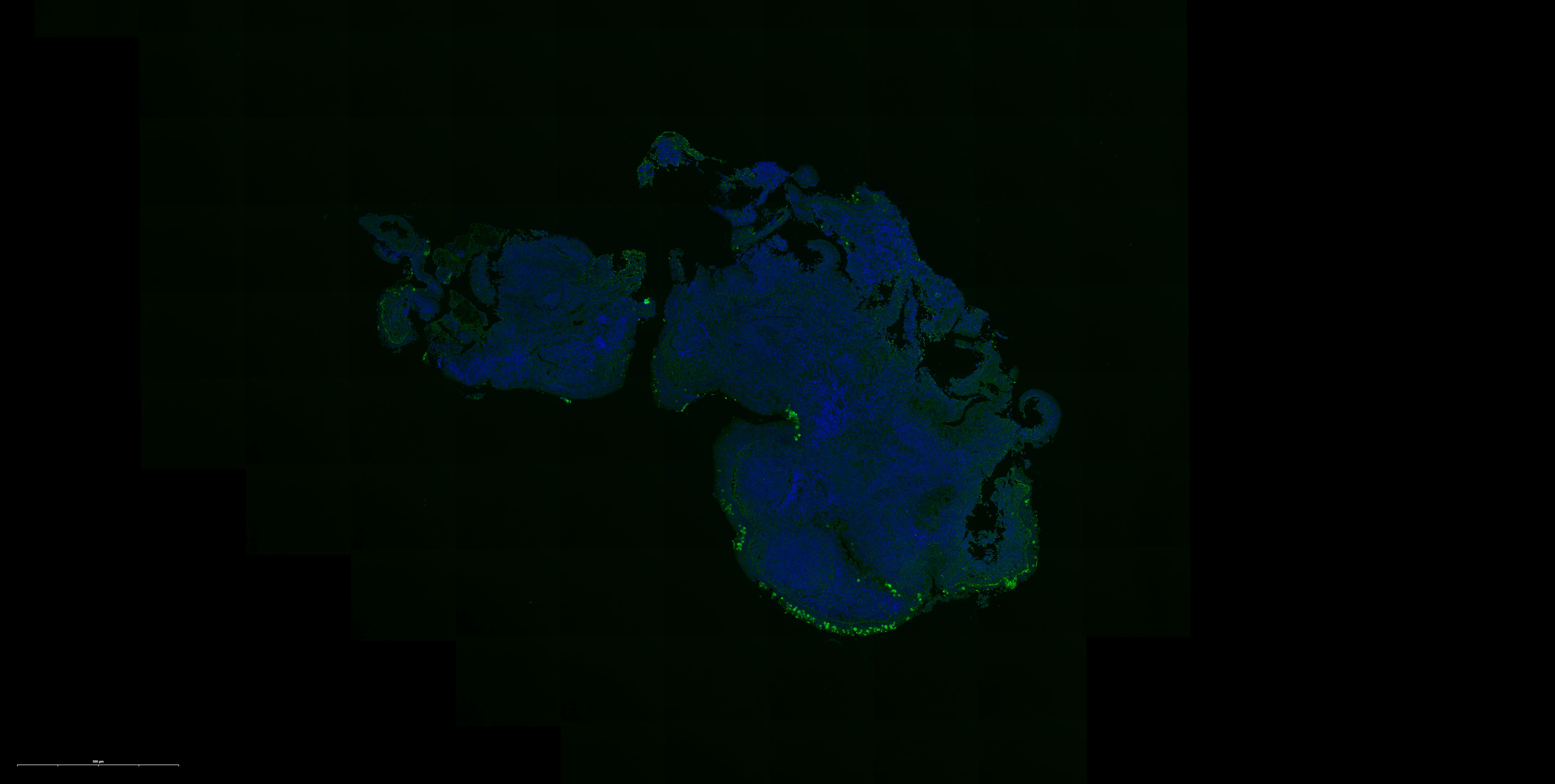

Supplement: Supplementary file 22 — Figure EV3 Source Data [file 44321_2026_419_MOESM22_ESM.zip › Source data Fig.EV3/CD3-Severe1.jpg]

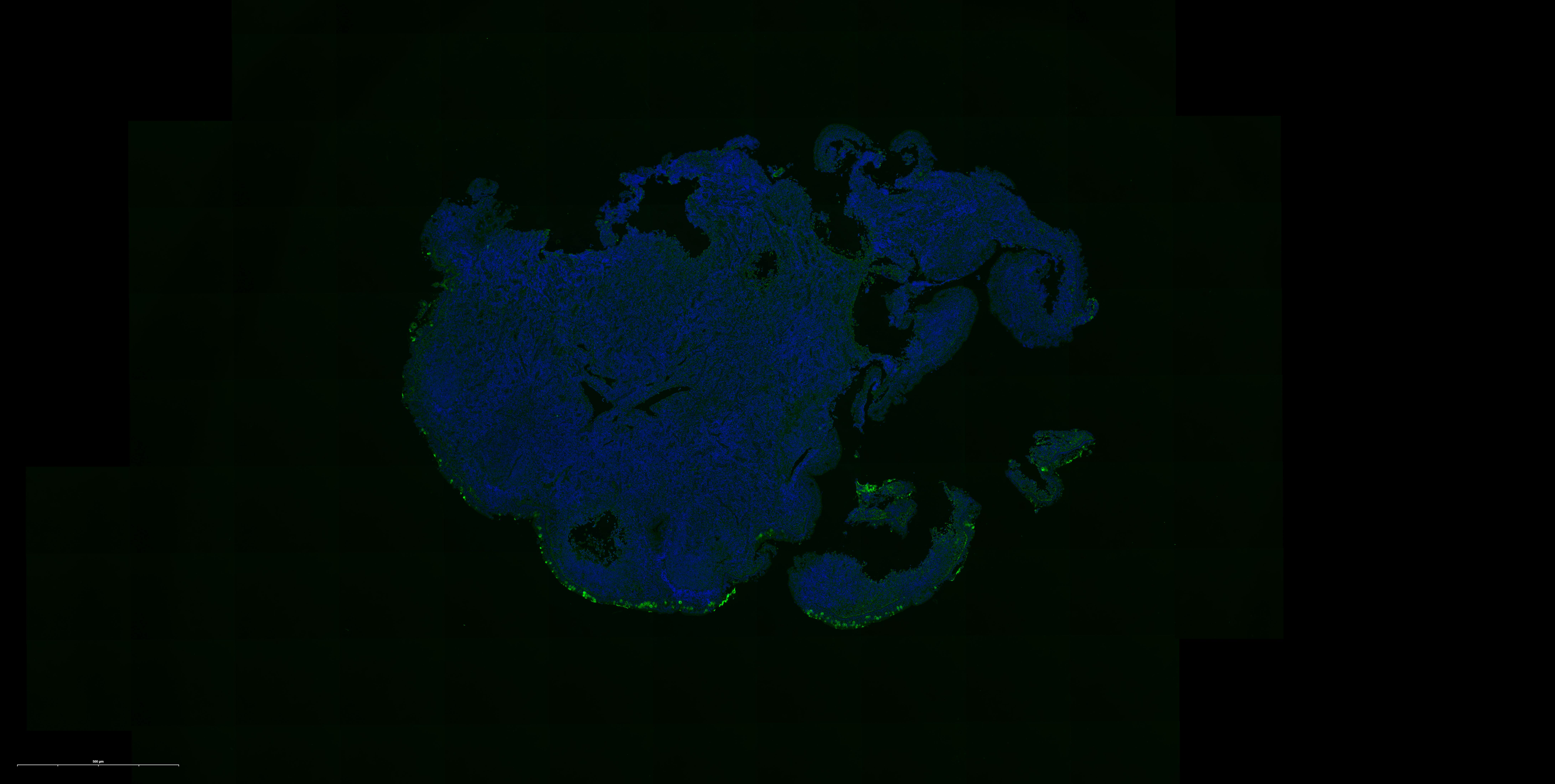

Supplement: Supplementary file 22 — Figure EV3 Source Data [file 44321_2026_419_MOESM22_ESM.zip › Source data Fig.EV3/CD3-Severe2.jpg]

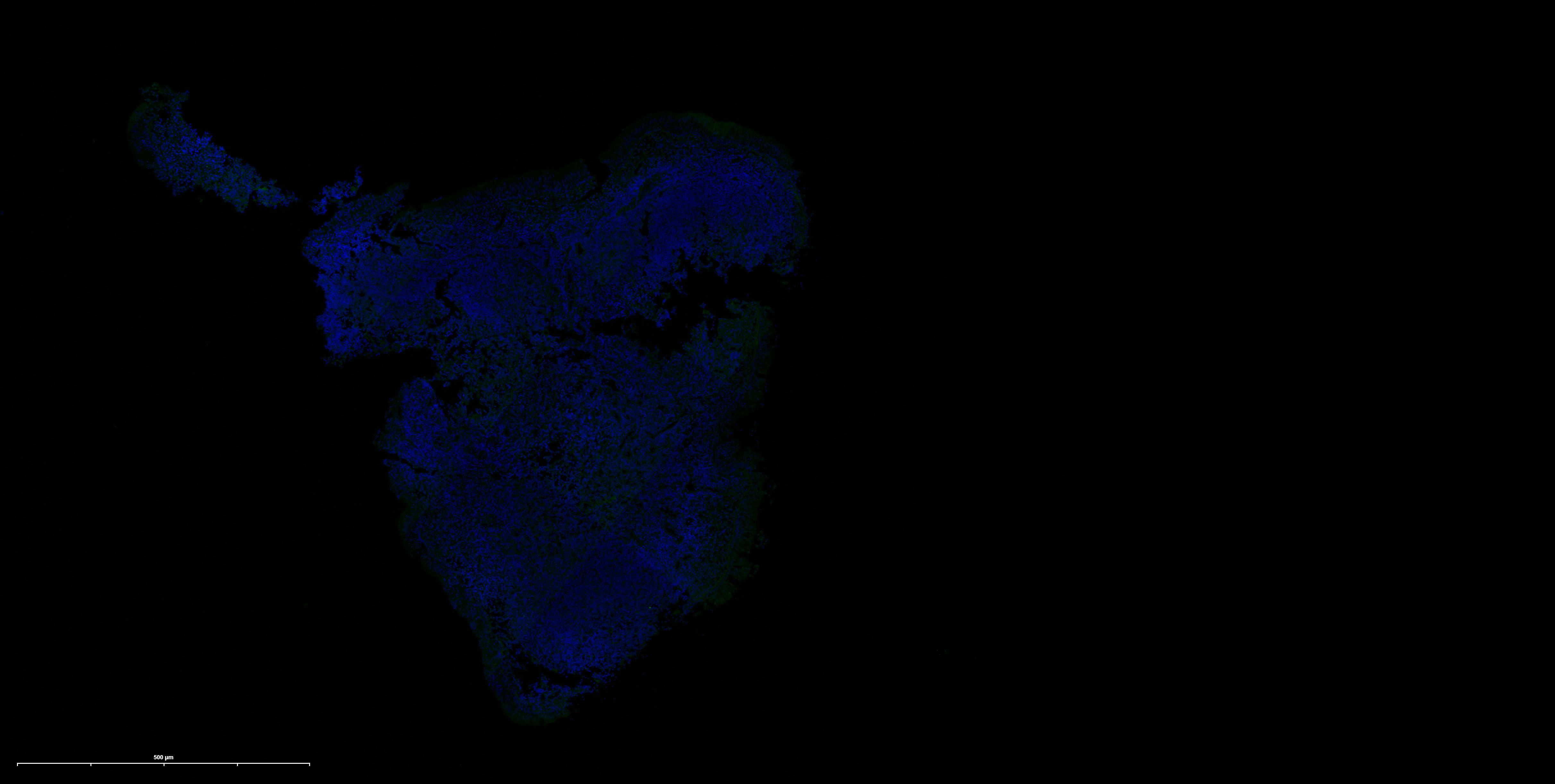

Supplement: Supplementary file 22 — Figure EV3 Source Data [file 44321_2026_419_MOESM22_ESM.zip › Source data Fig.EV3/CD3-Severe3.jpg]

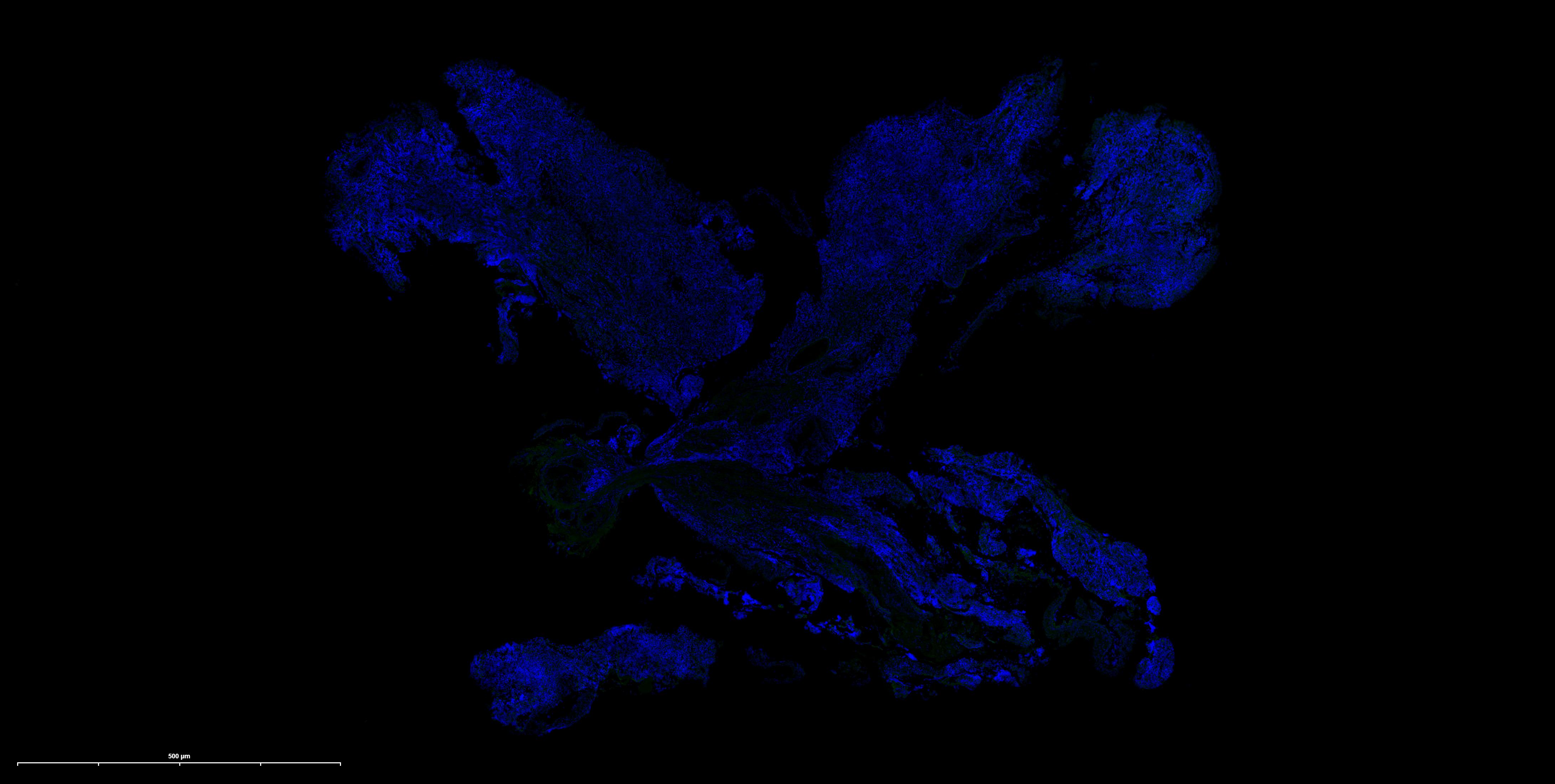

Supplement: Supplementary file 22 — Figure EV3 Source Data [file 44321_2026_419_MOESM22_ESM.zip › Source data Fig.EV3/CD3-Mild1.jpg]

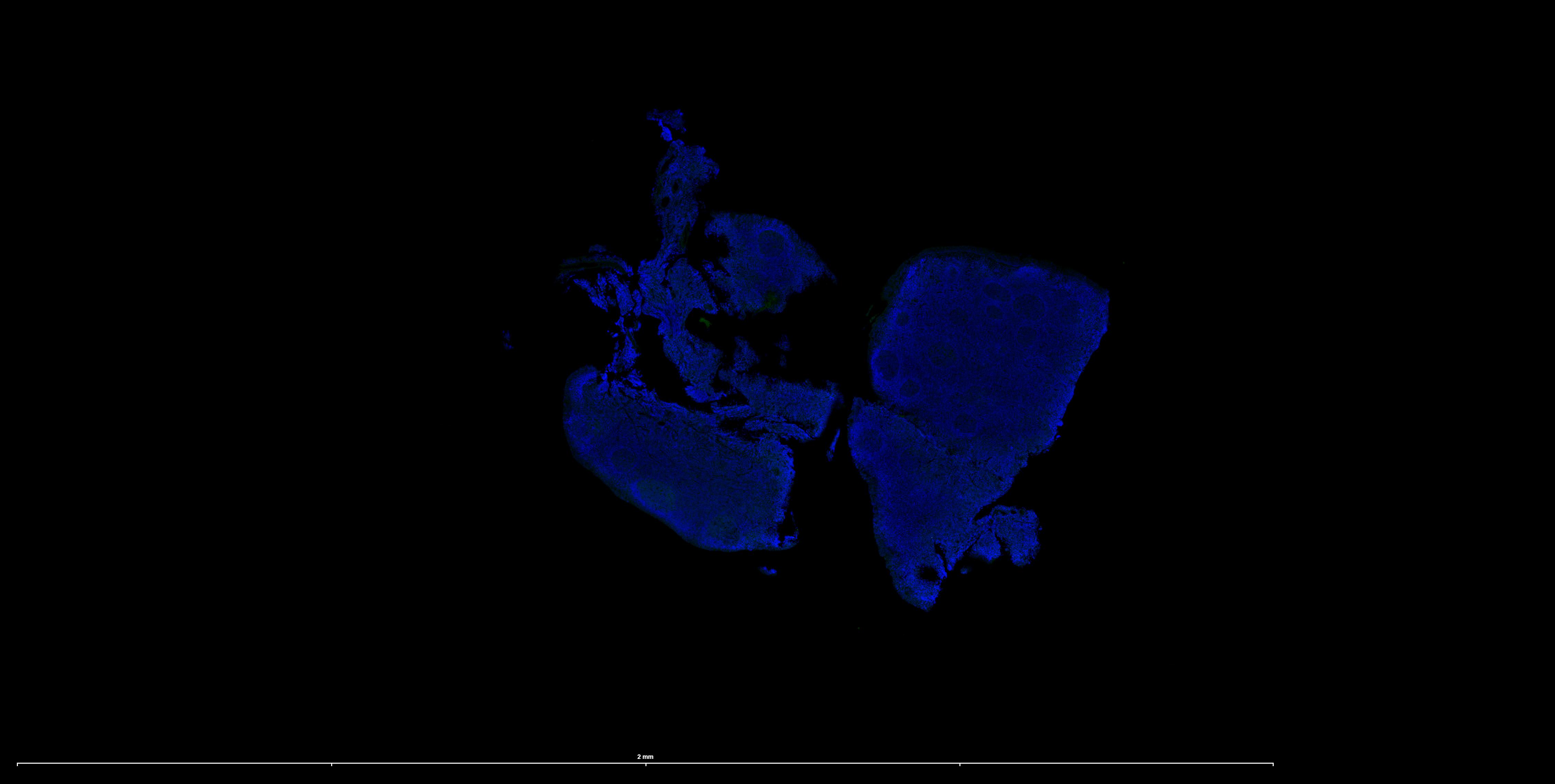

Supplement: Supplementary file 22 — Figure EV3 Source Data [file 44321_2026_419_MOESM22_ESM.zip › Source data Fig.EV3/CD3-Mild2.jpg]

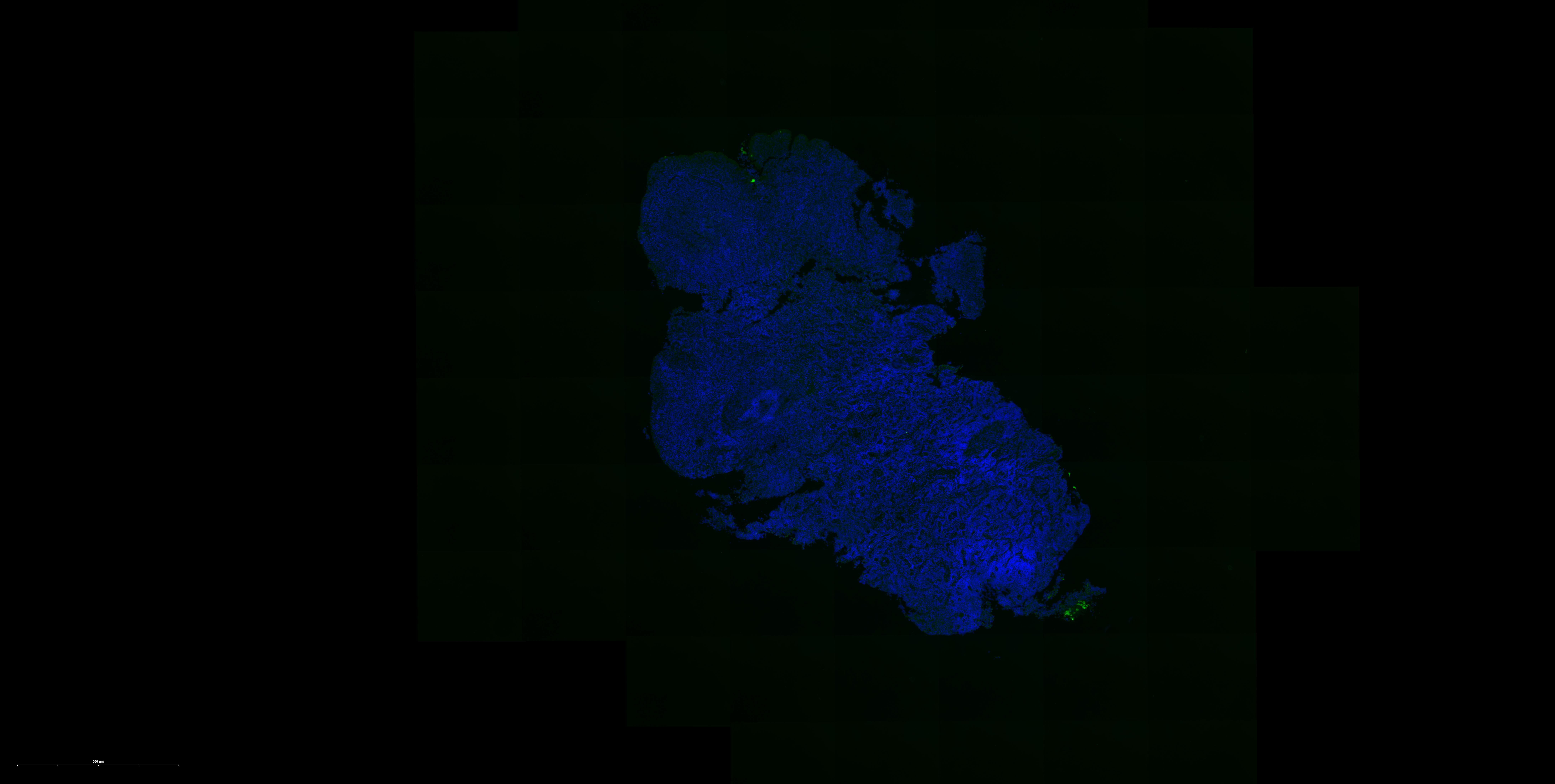

Supplement: Supplementary file 22 — Figure EV3 Source Data [file 44321_2026_419_MOESM22_ESM.zip › Source data Fig.EV3/CD3-Control1.jpg]

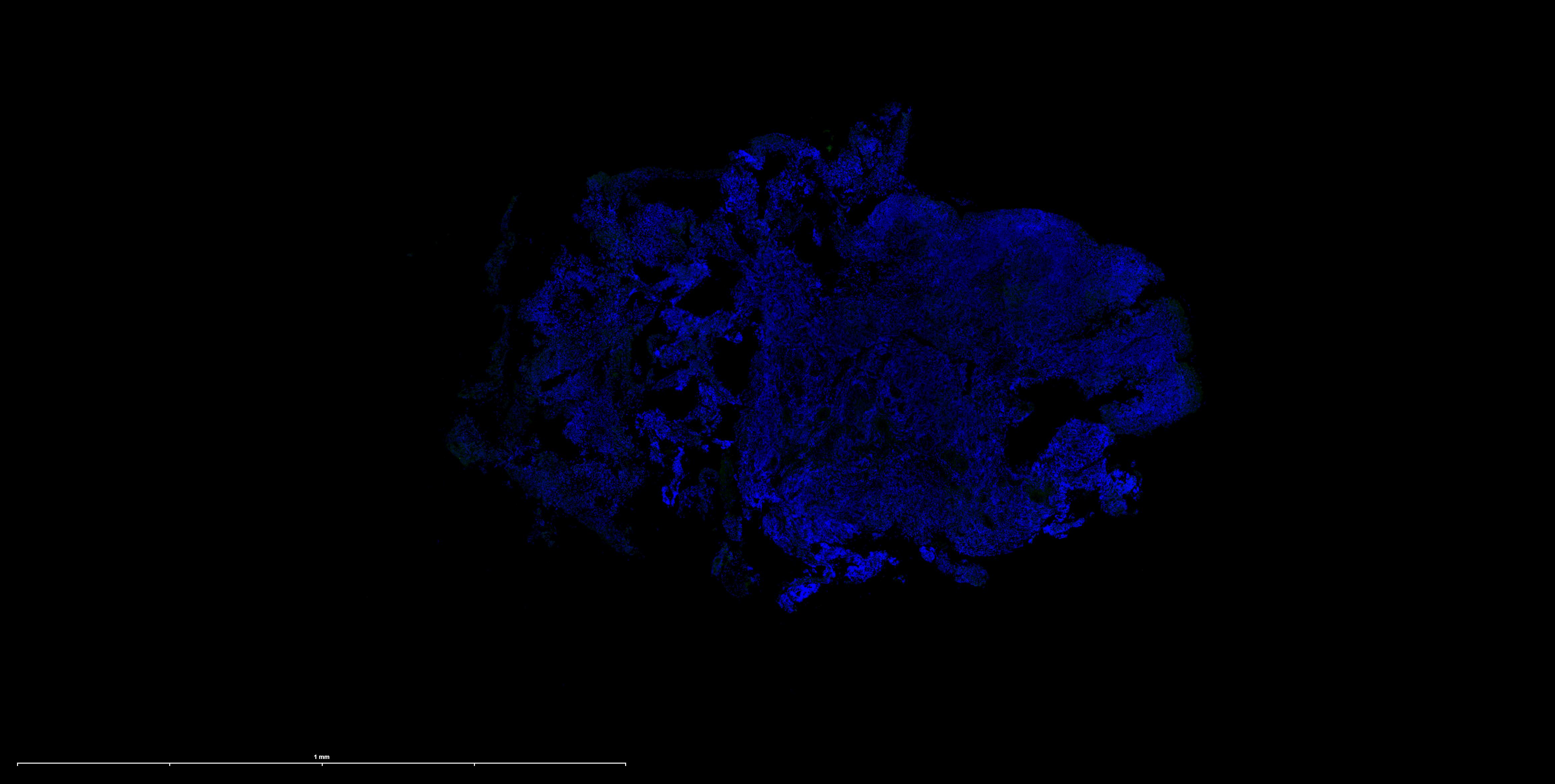

Supplement: Supplementary file 22 — Figure EV3 Source Data [file 44321_2026_419_MOESM22_ESM.zip › Source data Fig.EV3/CD3-Control2.jpg]

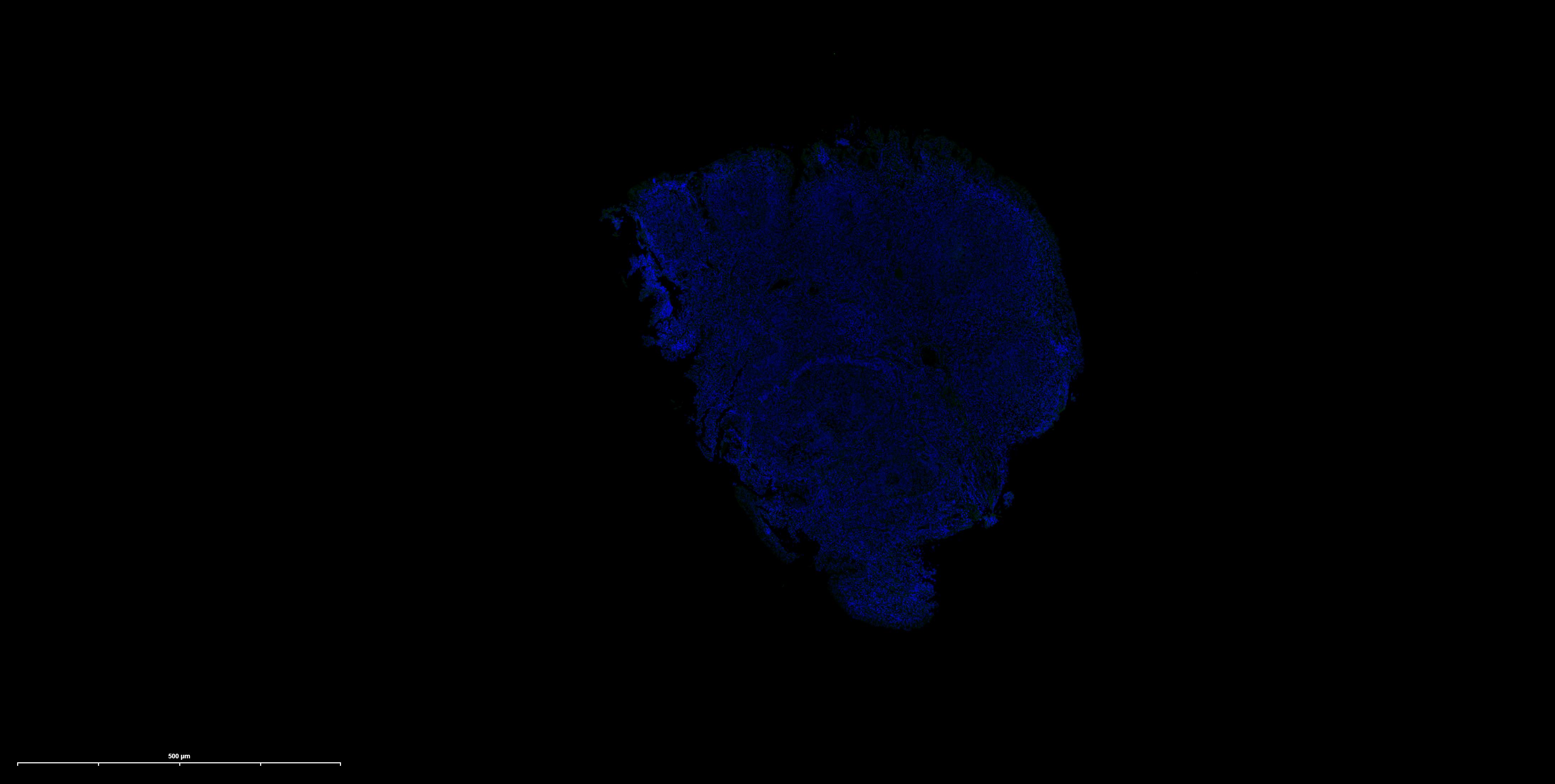

Supplement: Supplementary file 22 — Figure EV3 Source Data [file 44321_2026_419_MOESM22_ESM.zip › Source data Fig.EV3/CD3-Control3.jpg]

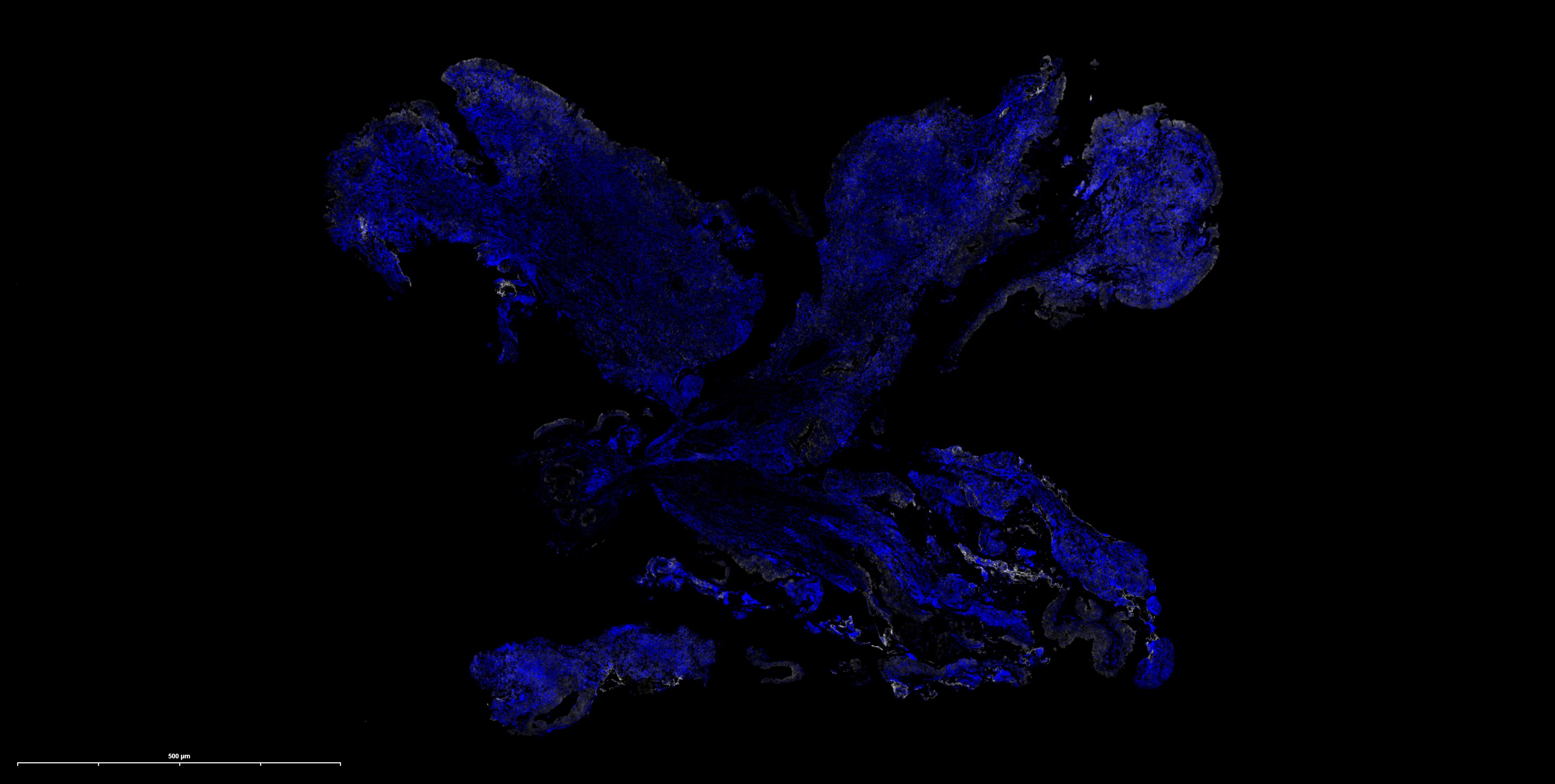

Supplement: Supplementary file 22 — Figure EV3 Source Data [file 44321_2026_419_MOESM22_ESM.zip › Source data Fig.EV3/IFNγ-Mild1.jpg]

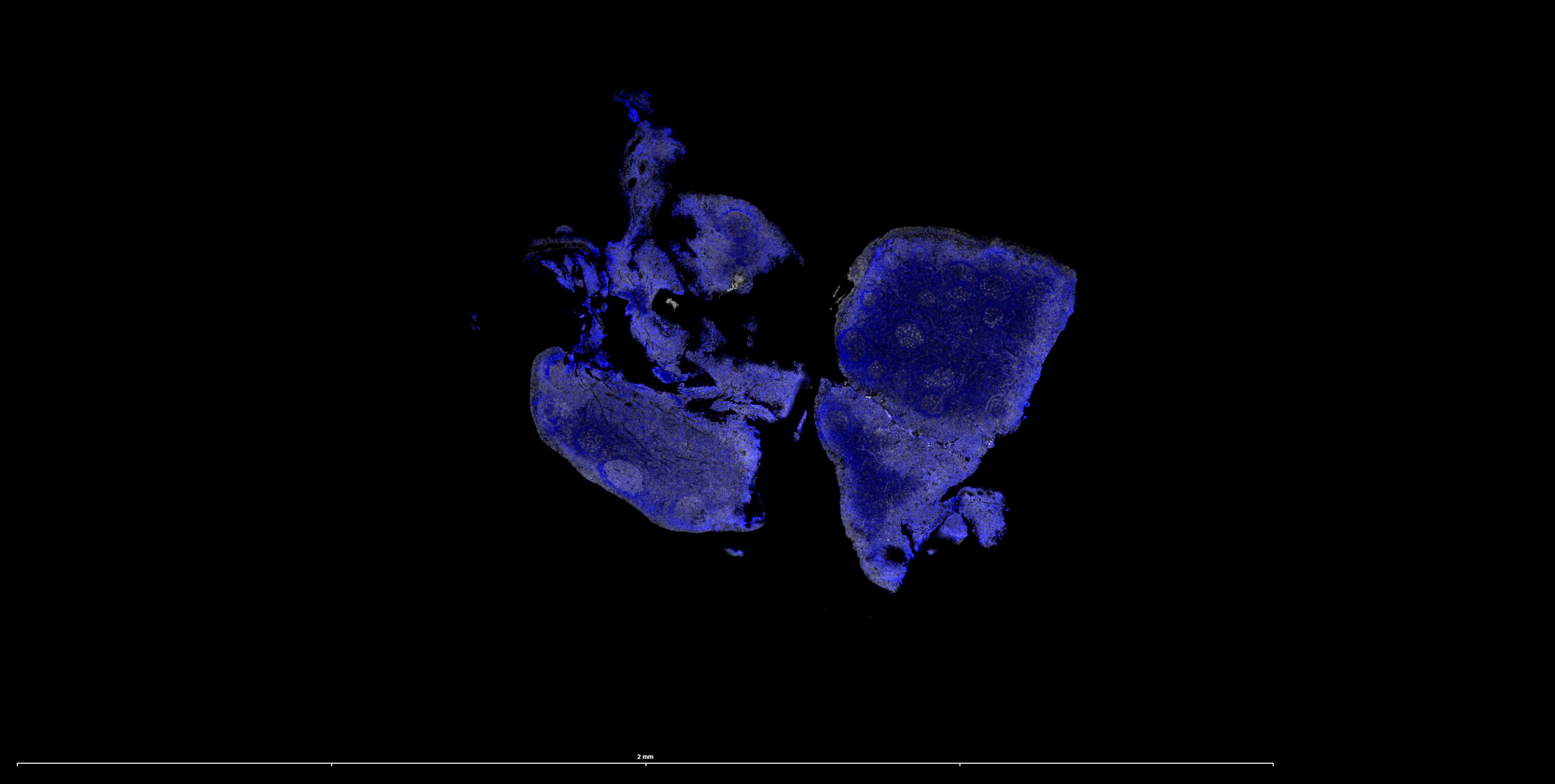

Supplement: Supplementary file 22 — Figure EV3 Source Data [file 44321_2026_419_MOESM22_ESM.zip › Source data Fig.EV3/IFNγ-Mild2.jpg]

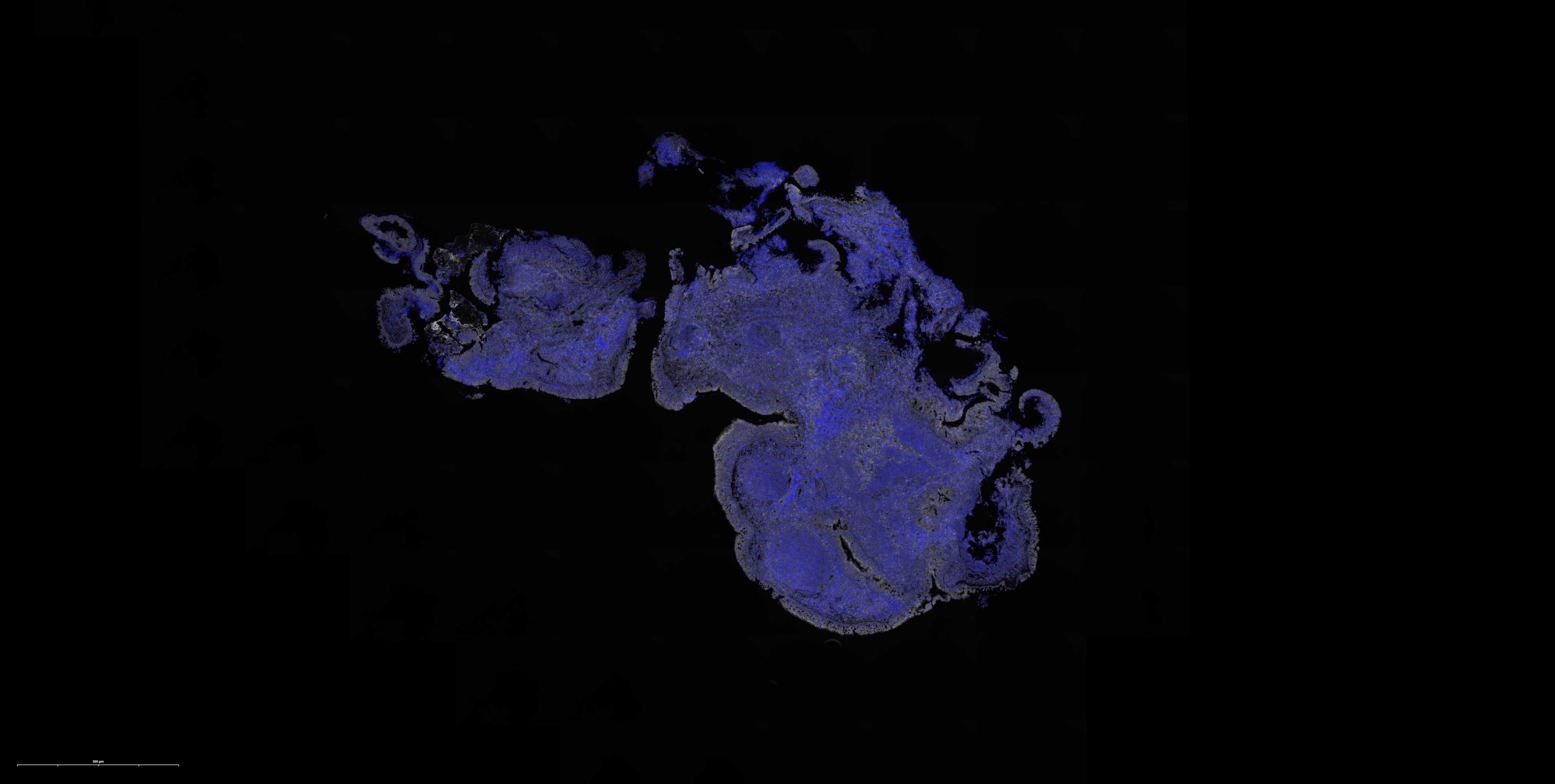

Supplement: Supplementary file 22 — Figure EV3 Source Data [file 44321_2026_419_MOESM22_ESM.zip › Source data Fig.EV3/IFNγ-Severe1.jpg]

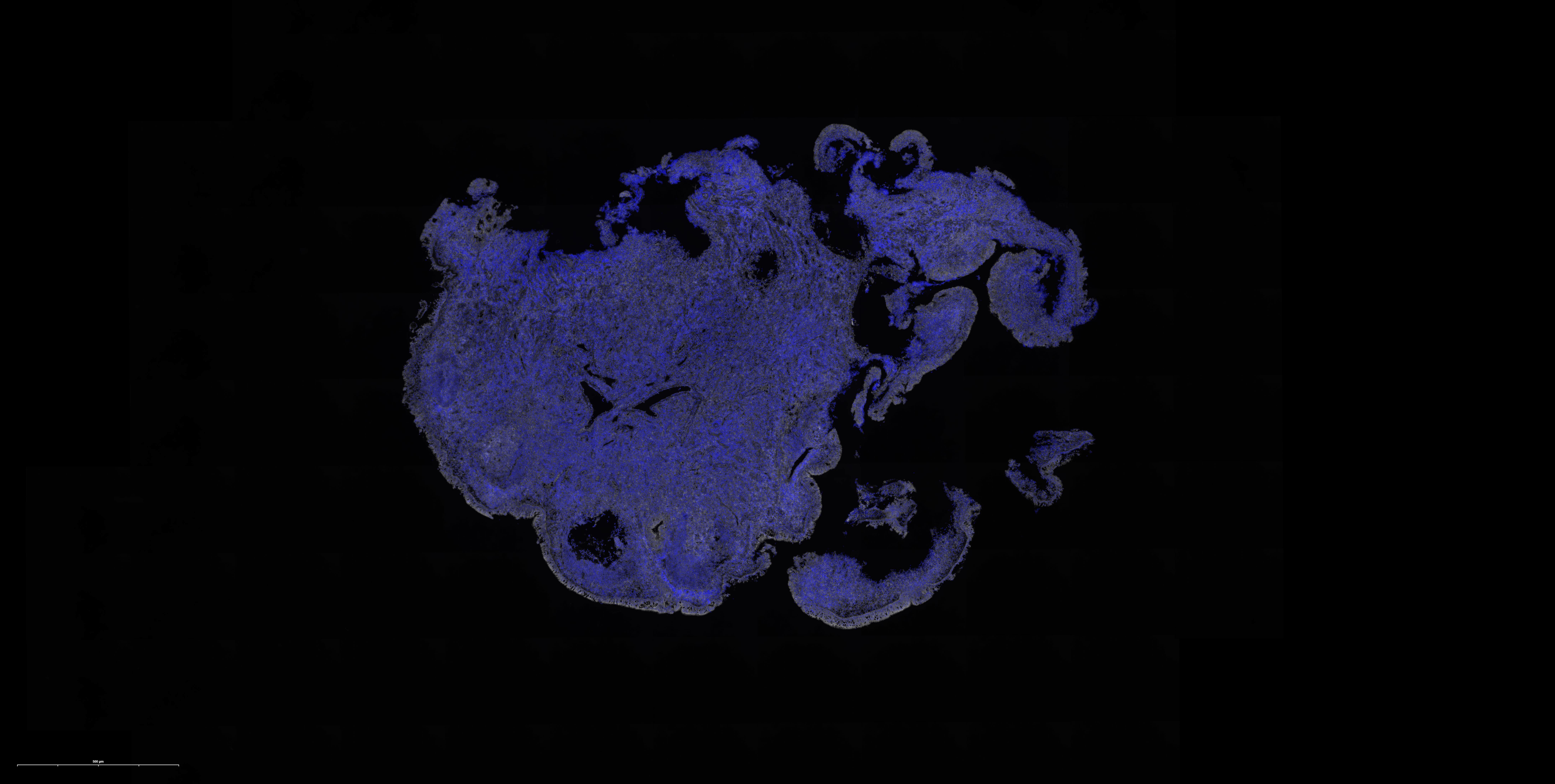

Supplement: Supplementary file 22 — Figure EV3 Source Data [file 44321_2026_419_MOESM22_ESM.zip › Source data Fig.EV3/IFNγ-Severe2.jpg]

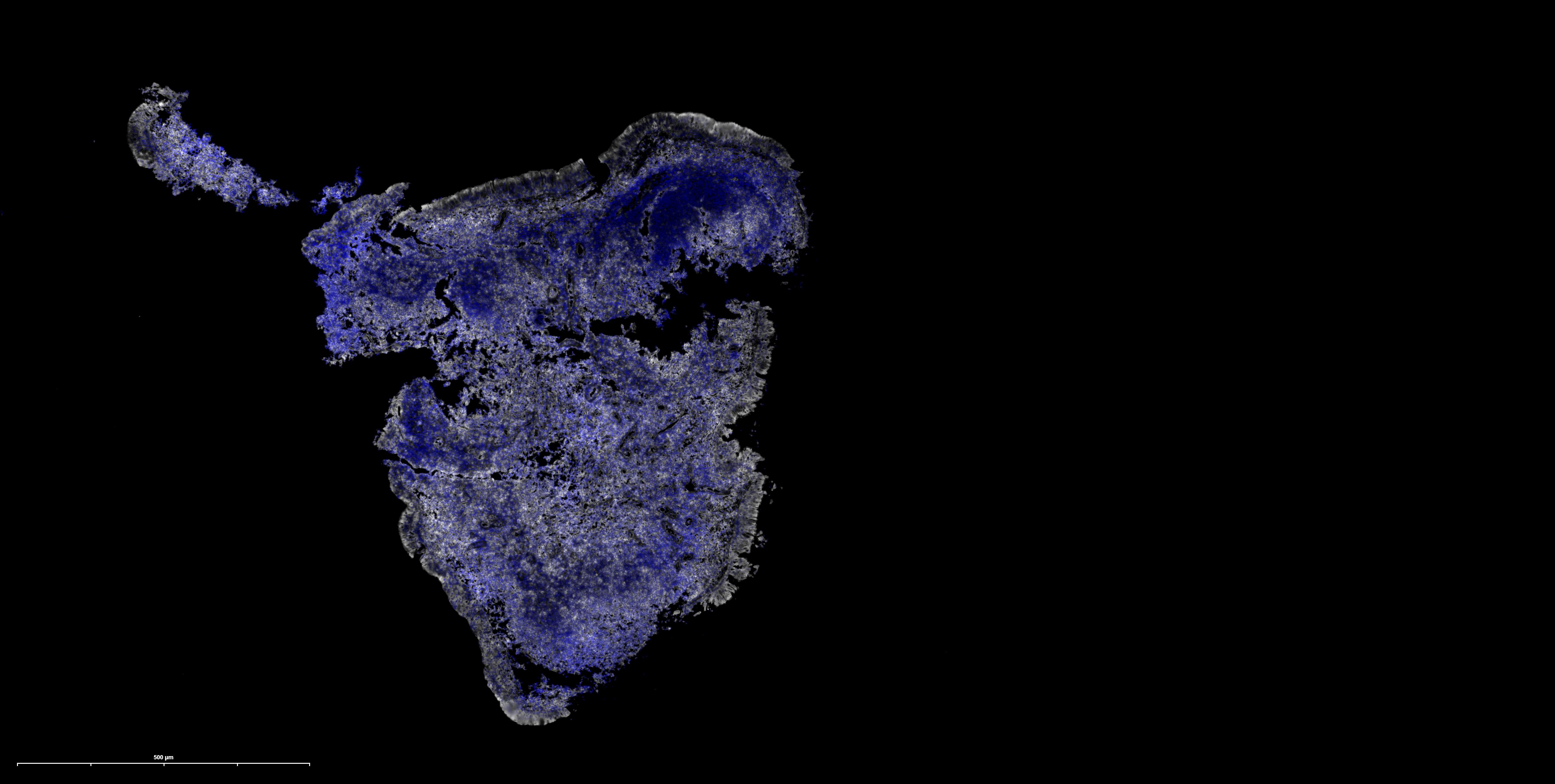

Supplement: Supplementary file 22 — Figure EV3 Source Data [file 44321_2026_419_MOESM22_ESM.zip › Source data Fig.EV3/IFNγ-Severe3.jpg]

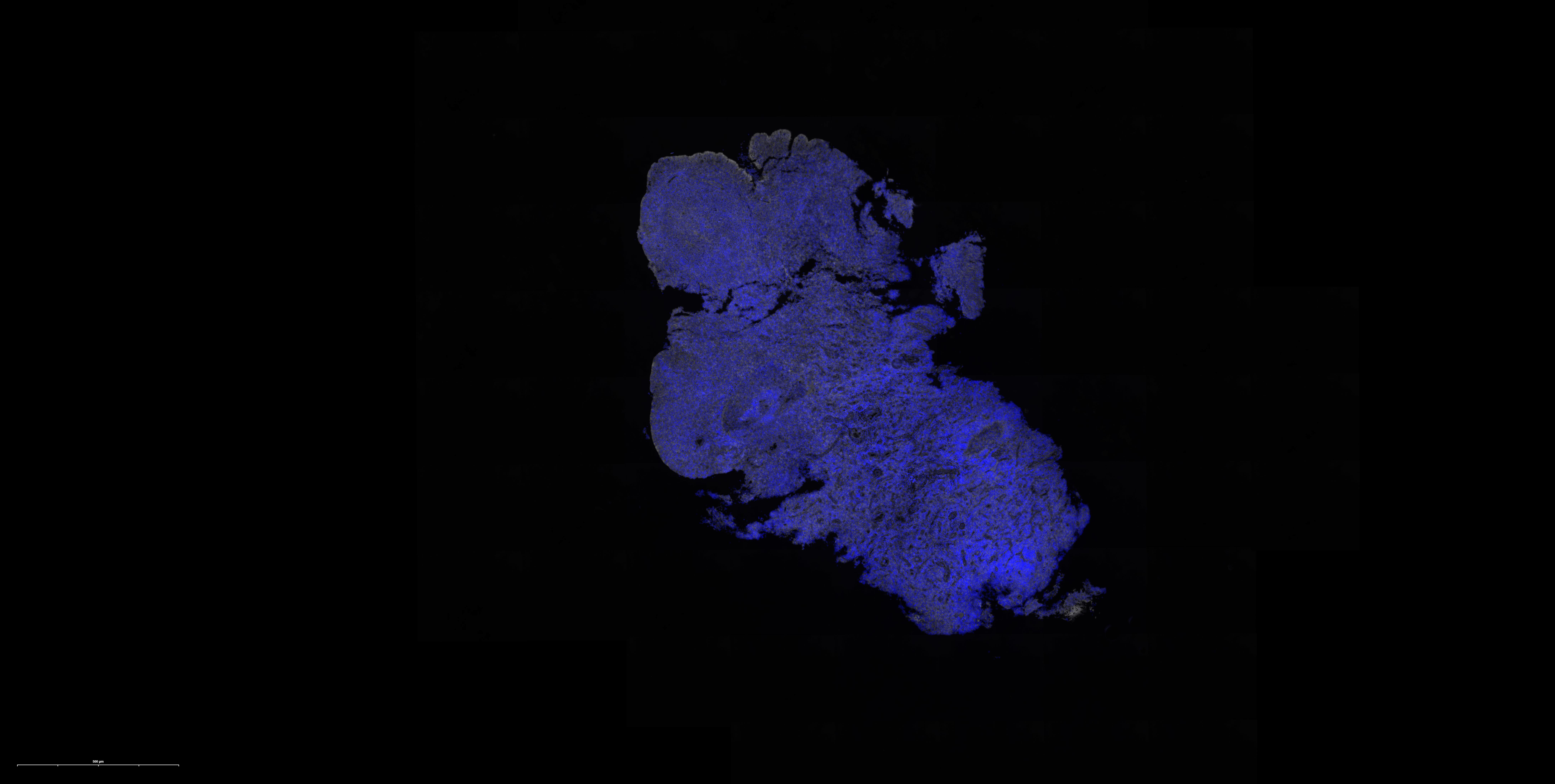

Supplement: Supplementary file 22 — Figure EV3 Source Data [file 44321_2026_419_MOESM22_ESM.zip › Source data Fig.EV3/IFNγ-Control1.jpg]

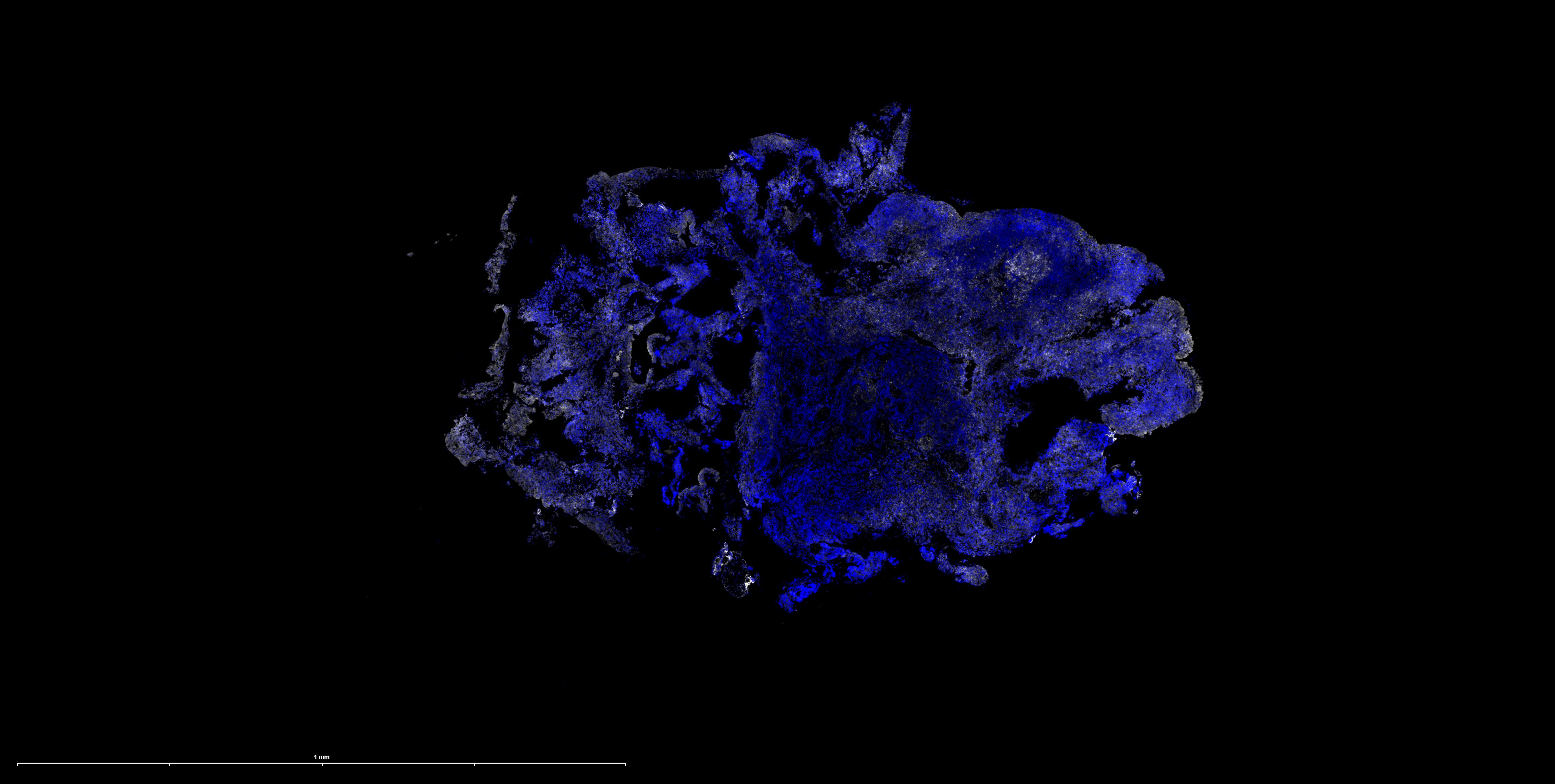

Supplement: Supplementary file 22 — Figure EV3 Source Data [file 44321_2026_419_MOESM22_ESM.zip › Source data Fig.EV3/IFNγ-Control2.jpg]

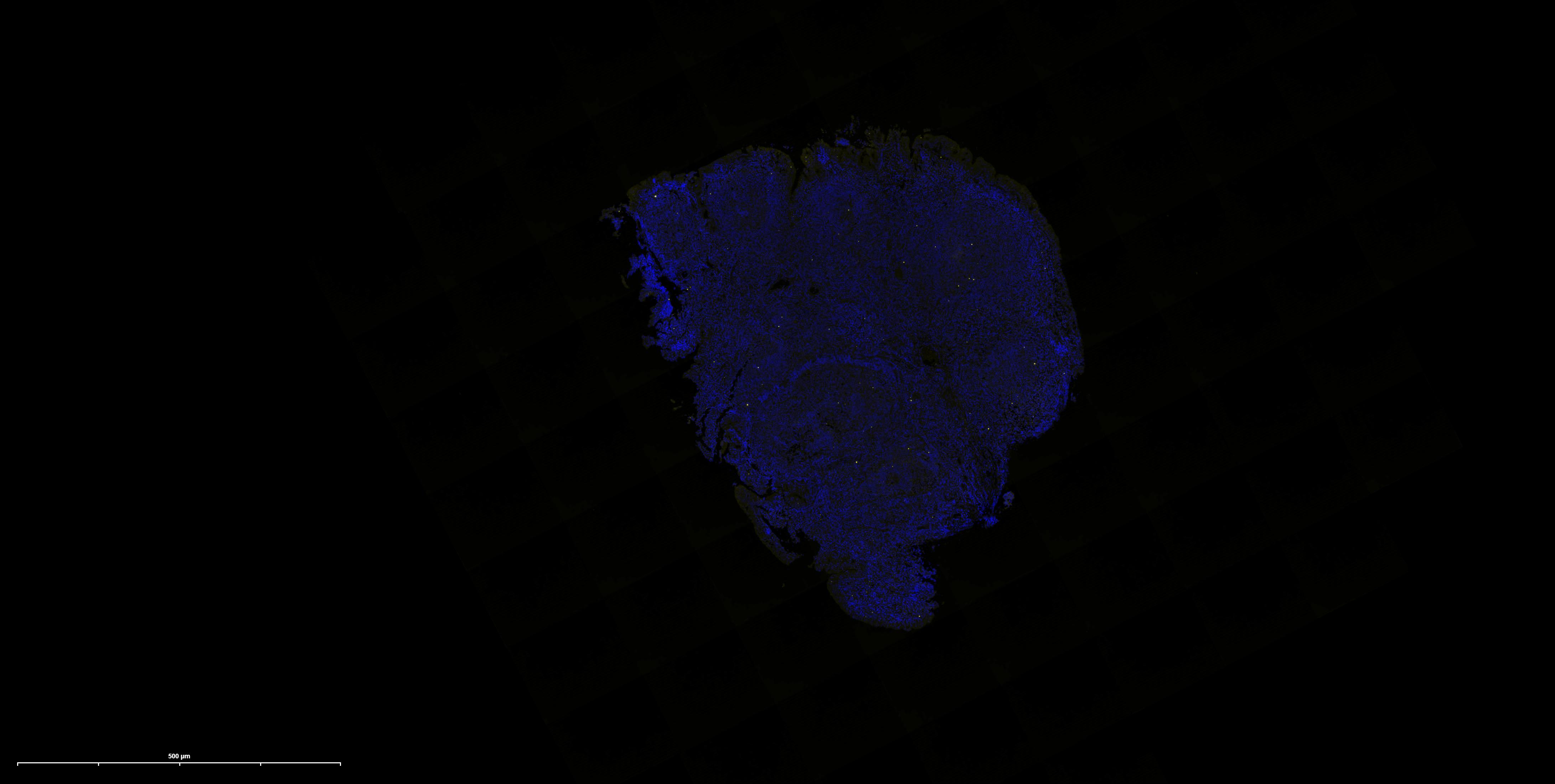

Supplement: Supplementary file 22 — Figure EV3 Source Data [file 44321_2026_419_MOESM22_ESM.zip › Source data Fig.EV3/IFNγ-Control3.jpg]
