# Supplementary material for: Disease-stage-specific immunometabolic remodeling in pediatric obstructive sleep apnea: a single-cell transcriptomic atlas of adenoid tissue
Source: EMBO Mol Med. 2026 Apr 27;18(6):2483–513. doi: 10.1038/s44321-026-00419-3 (PMC13270133; doi:10.1038/s44321-026-00419-3)
Supplement: Supplementary file 23 — Expanded View Figures [file 44321_2026_419_MOESM23_ESM.pdf]

Expanded View Figures

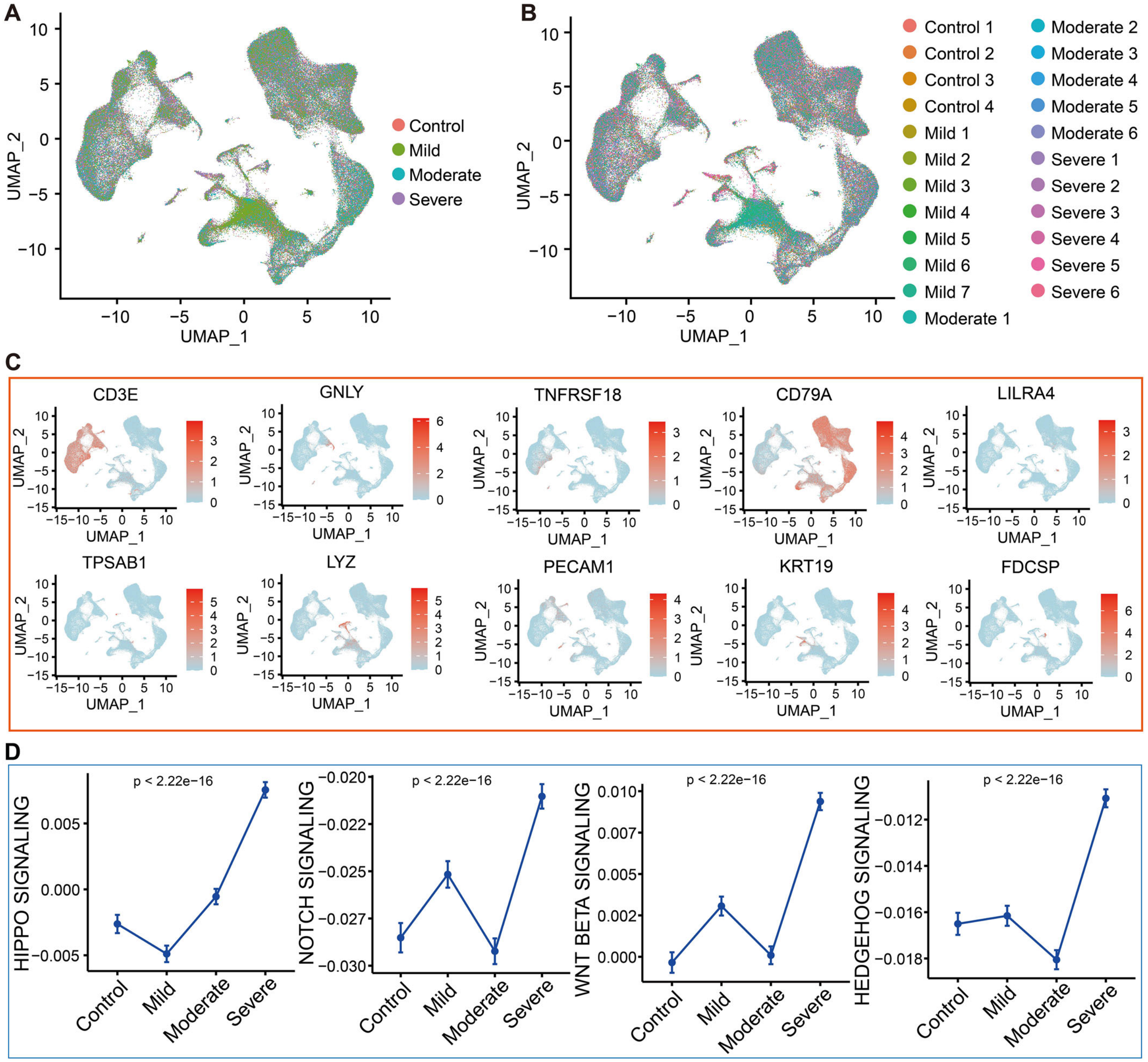

Figure EV1. Identification of the main cell population of adenoid tissue and changes in the gene set related to growth and development.

(**A**, **B**) UMAP plots show whether there are batch effects among major cell populations of hypertrophic adenoids between different groups and different samples. A uniform distribution indicates that batch effects between groups and samples have been removed. (**C**) The UMAP plot shows the expression levels of marker genes corresponding to major cell populations of hypertrophic adenoids. (**D**) Activity scores of growth and development-related gene sets in adenoids at different disease stages, including Hippo, Notch, Wnt, and Hedgehog gene sets. Gene set scoring only represents the overall activity of the transcriptional expression levels of these functional genes. Multiple Kruskal–Wallis tests were used to assess the overall significance of gene set scoring across four groups. The above error bars represent the median absolute deviation (MAD) of gene set activity.

 © The Author(s)

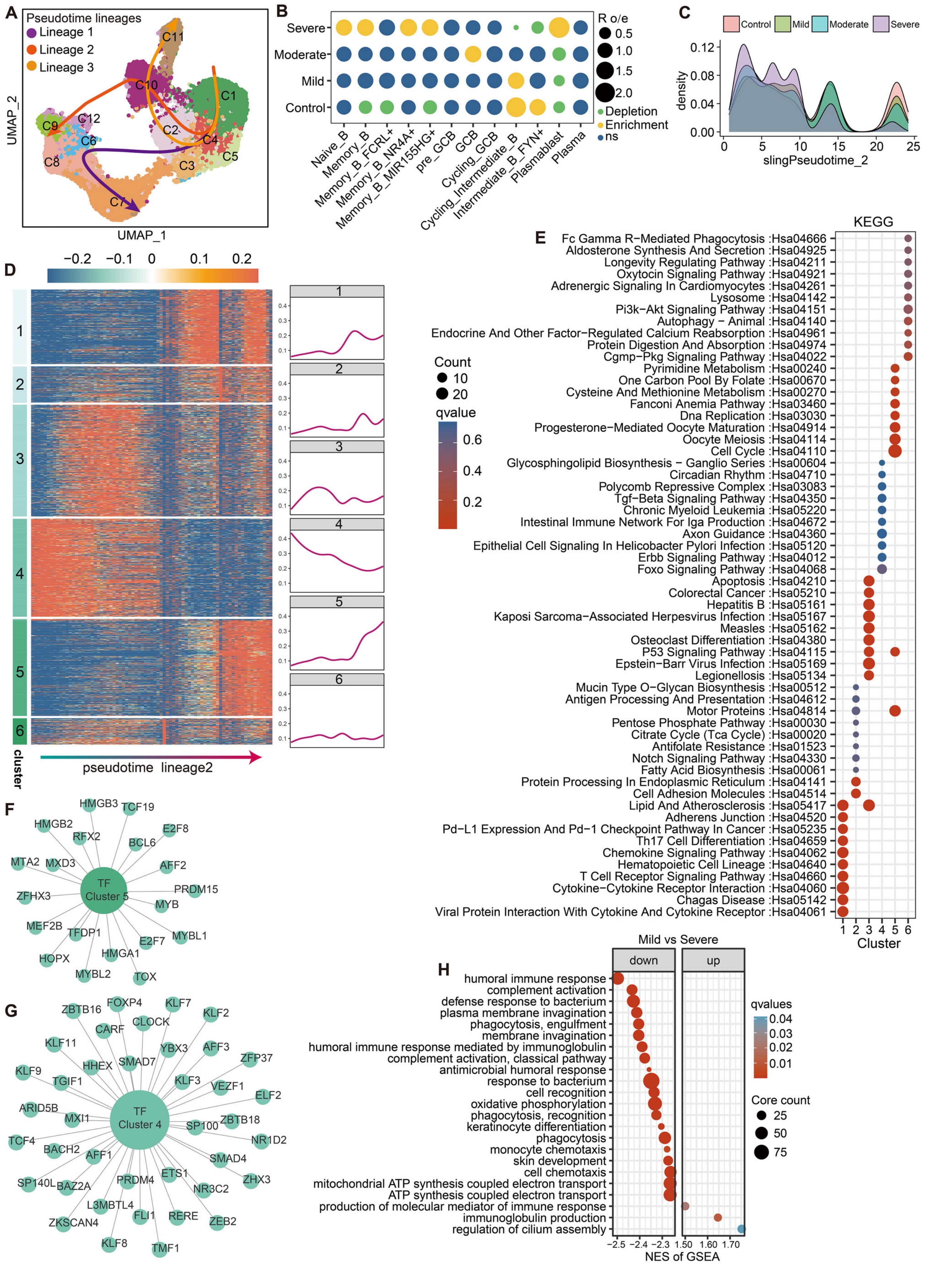
A
Pseudotime lineages
Lineage 1
Lineage 2
Lineage 3
UMAP_1
UMAP_2
B
Severe
Moderate
Mild
Control
R o/e
Depletion
Enrichment
ns
Naive_B
Memory_B
Memory_B_FCRL+
Memory_B_NR4A+
Memory_B_MIR155HG+
pre_GCB
GCB
Cycling_GCB
Cycling_Intermediate_B
Intermediate_B_FYN+
Plasmablast
Plasma
C
Control
Mild
Moderate
Severe
density
slingPseudotime_2
D
cluster
pseudotime lineage2
E
KEGG
Count
qvalue
Cluster
F
TF Cluster 5
G
TF Cluster 4
H
Mild vs Severe
down
up
qvalues
Core count
NES of GSEA

Figure EV2. Identification of B cell subsets and results of branch trajectory analysis.

(**A**) UMAP plot shows three B-cell differentiation trajectories identified based on pseudotime analysis. (**B**) Enrichment of the abundance of different B cell subsets in adenoid groups with different disease courses. (**C**) The density plot shows the proportion of cells along the second differentiation trajectory (intermediate B cells) in different disease course groups, and it is found that the severe OSA group is significantly reduced at the end of differentiation. (**D**) The heatmap shows the clustering and expression changes of dynamically differentially expressed genes (DDEGs) during the second differentiation trajectory (intermediate B cells). (**E**) KEGG enrichment analysis of DDEGs during the second differentiation trajectory (intermediate B cells). (**F**) Transcription factors included in the gradient upregulated genes (Cluster 5) during the second differentiation trajectory (intermediate B cells). (**G**) Transcription factors included in the gradient downregulated genes (Cluster 4) during the second differentiation trajectory (intermediate B cells). (**H**) GSEA enrichment analysis of the total B cell expression profiles between the mild and severe OSA groups, used to show the biological processes that are significantly upregulated or downregulated between the groups.

 © The Author(s)

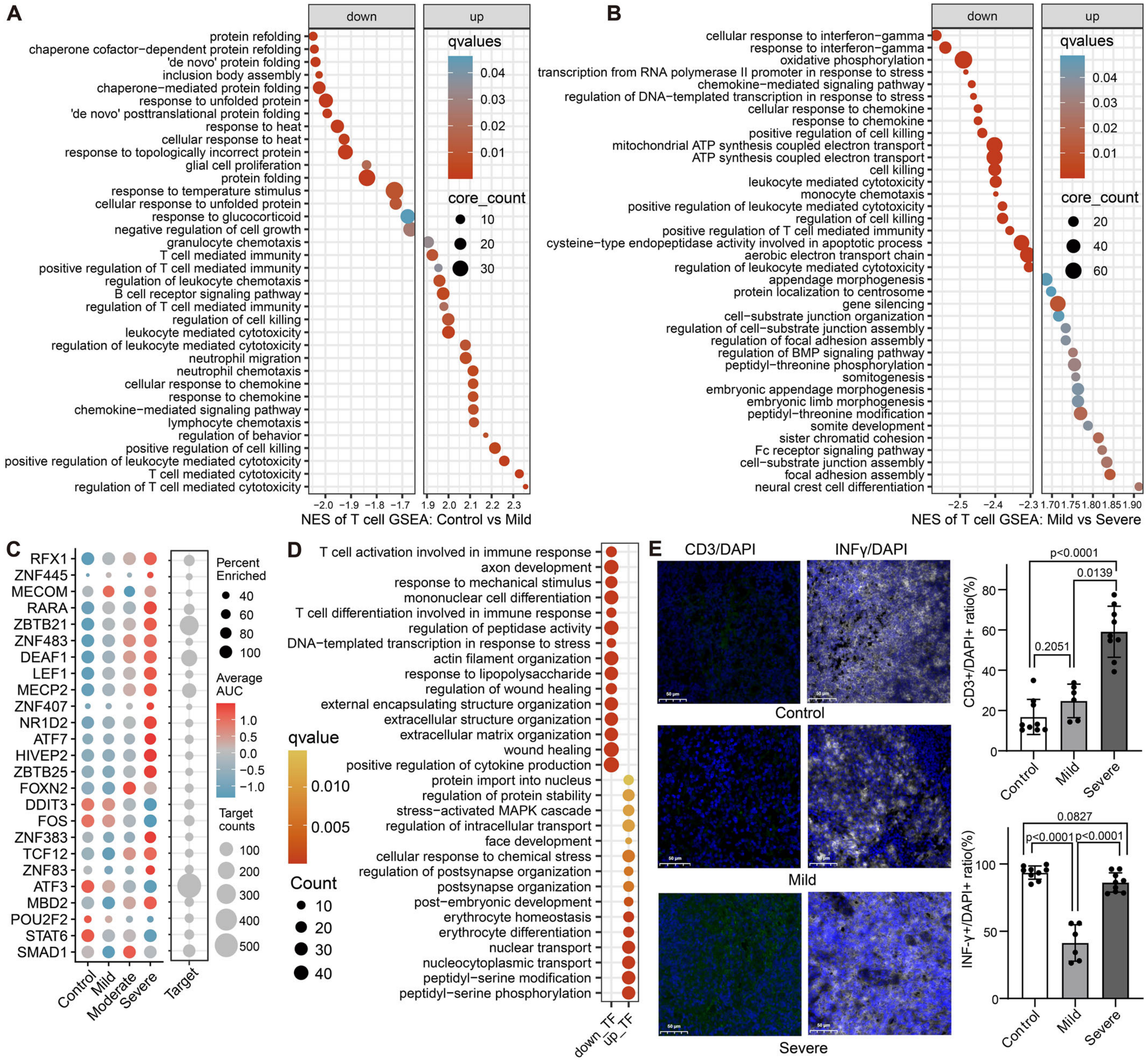

Figure EV3. GSEA enrichment results and transcription factors associated with T-cell activity among different groups.

(**A**) GSEA enrichment analysis results of the overall T cell expression profiles between the control and mild OSA groups. (**B**) GSEA enrichment analysis results of the overall T cell expression profiles between the mild and severe OSA groups. (**C**) Dotplot shows key transcription factors with gradient changes along the progression of different adenoid diseases. The color represents the AUC enrichment degree of transcription factors in different modules, the size of the circle represents the percentage of enriched cells, and the size of the gray circle corresponds to the number of target genes regulated by transcription factors. (**D**) Functional enrichment analysis of transcription factors and their target genes that are upregulated or downregulated in T cells of the severe OSA group. (**E**) Immunofluorescence showed the protein expression levels of CD3 and IFNγ in adenoid tissues of the control, mild and severe OSA groups. Representative immunofluorescence images were shown, DAPI (blue), CD3 (green), IFNγ (white), 20×, scale bar: 50 μm. Samples were collected from the control group ($n = 3$), the mild group ($n = 2$), and the severe group ($n = 3$). For each sample within each group, three visual fields of equal area were randomly selected for quantification, and showed as mean ± SEM. Statistical testing using one-way analysis of variance. Source data are available online for this figure.

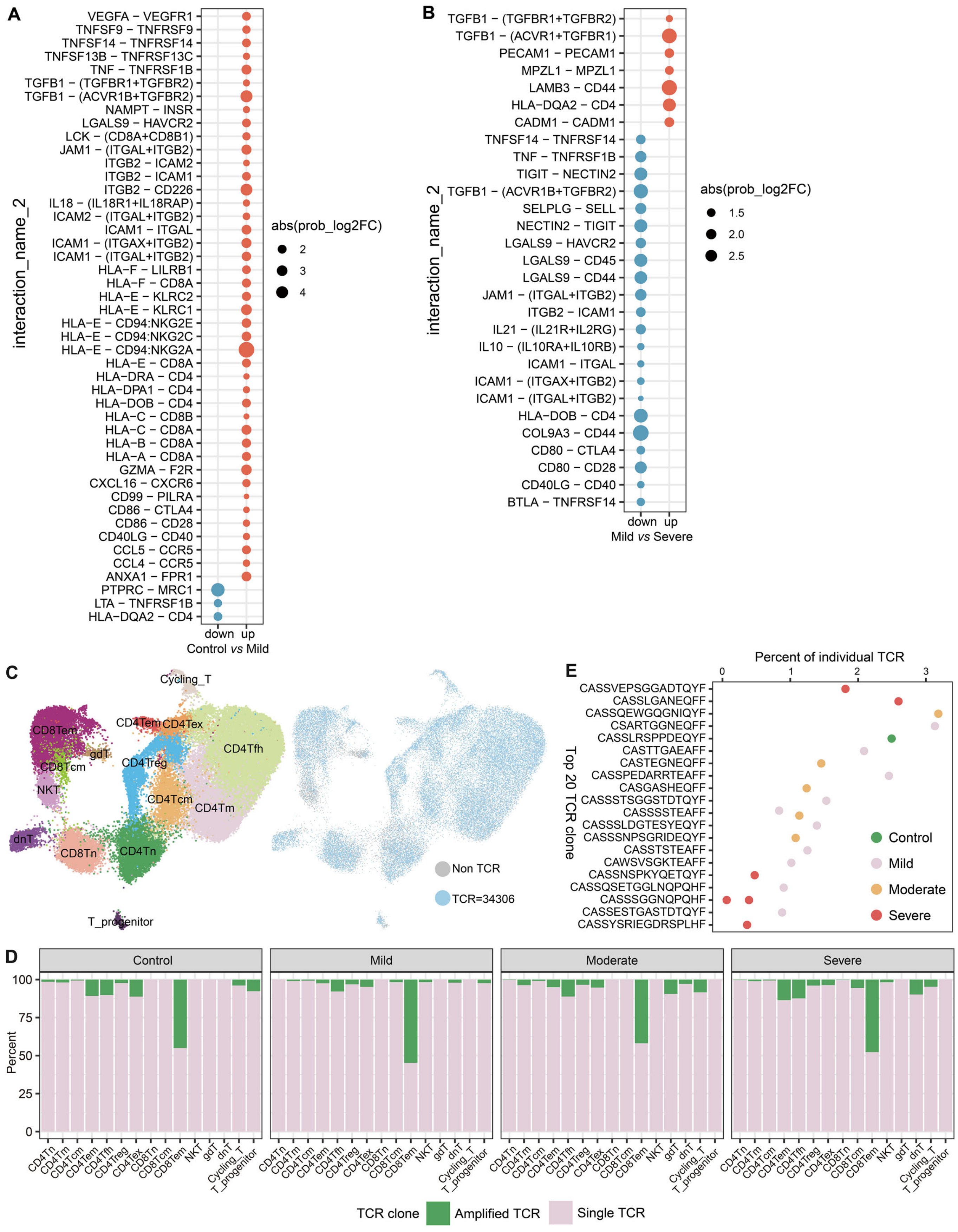
A
VEGFA − VEGFR1
TNFSF9 − TNFRSF9
TNFSF14 − TNFRSF14
TNFSF13B − TNFRSF13C
TNF − TNFRSF1B
TGFB1 − (TGFBR1+TGFBR2)
TGFB1 − (ACVR1B+TGFBR2)
NAMPT − INSR
LGALS9 − HAVCR2
LCK − (CD8A+CD8B1)
JAM1 − (ITGAL+ITGB2)
ITGB2 − ICAM2
ITGB2 − ICAM1
ITGB2 − CD226
IL18 − (IL18R1+IL18RAP)
ICAM2 − (ITGAL+ITGB2)
ICAM1 − ITGAL
ICAM1 − (ITGAX+ITGB2)
ICAM1 − (ITGAL+ITGB2)
HLA−F − LILRB1
HLA−F − CD8A
HLA−E − KLRC2
HLA−E − KLRC1
HLA−E − CD94:NKG2E
HLA−E − CD94:NKG2C
HLA−E − CD94:NKG2A
HLA−E − CD8A
HLA−DRA − CD4
HLA−DPA1 − CD4
HLA−DOB − CD4
HLA−C − CD8B
HLA−C − CD8A
HLA−B − CD8A
HLA−A − CD8A
GZMA − F2R
CXCL16 − CXCR6
CD99 − PILRA
CD86 − CTLA4
CD86 − CD28
CD40LG − CD40
CCL5 − CCR5
CCL4 − CCR5
ANXA1 − FPR1
PTPRC − MRC1
LTA − TNFRSF1B
HLA−DQA2 − CD4
interaction_name_2
down up
Control vs Mild
abs(prob_log2FC)
2
3
4
B
TGFB1 − (TGFBR1+TGFBR2)
TGFB1 − (ACVR1+TGFBR1)
PECAM1 − PECAM1
MPZL1 − MPZL1
LAMB3 − CD44
HLA−DQA2 − CD4
CADM1 − CADM1
TNFSF14 − TNFRSF14
TNF − TNFRSF1B
TIGIT − NECTIN2
TGFB1 − (ACVR1B+TGFBR2)
SELPLG − SELL
NECTIN2 − TIGIT
LGALS9 − HAVCR2
LGALS9 − CD45
LGALS9 − CD44
JAM1 − (ITGAL+ITGB2)
ITGB2 − ICAM1
IL21 − (IL21R+IL2RG)
IL10 − (IL10RA+IL10RB)
ICAM1 − ITGAL
ICAM1 − (ITGAX+ITGB2)
ICAM1 − (ITGAL+ITGB2)
HLA−DOB − CD4
COL9A3 − CD44
CD80 − CTLA4
CD80 − CD28
CD40LG − CD40
BTLA − TNFRSF14
interaction_name_2
down up
Mild vs Severe
abs(prob_log2FC)
1.5
2.0
2.5
C
Cycling_T
CD8Tem
CD4Tem
CD4Tex
gdT
CD4Tfh
CD4Treg
CD8Tcm
NKT
CD4Tcm
CD4Tm
dnT
CD8Tn
CD4Tn
T_progenitor
Non TCR
TCR=34306
E
Percent of individual TCR
0
1
2
3
Top 20 TCR clone
CASSVEPSGGADTQYF
CASSLGANEQFF
CASSQEWGQGNIQYF
CSARTGGNEQFF
CASSLRSPPDEQYF
CASTTGAEAFF
CASTEGNEQFF
CASSPEDARRTEAFF
CASGASHEQFF
CASSSTSGGSTDTQYF
CASSSSTEAFF
CASSSLDGTESYEQYF
CASSSNPSGRIDEQYF
CASSTSTEAFF
CAWSVSGKTEAFF
CASSNSPKYQETQYF
CASSQSETGGLNQPQHF
CASSSGGNQPQHF
CASSESTGASTDTQYF
CASSYSRIEGDRSPLHF
Control
Mild
Moderate
Severe
D
Control
Mild
Moderate
Severe
Percent
100
75
50
25
0
CD4Tn
CD4Tm
CD4Tcm
CD4Tem
CD4Tfh
CD4Treg
CD4Tex
CD8Tn
CD8Tcm
CD8Tem
NKT
gdT
dnT
Cycling_T
T_progenitor
TCR clone
Amplified TCR
Single TCR

 © The Author(s)

◀ **Figure EV4. UMAP plot shows the overlap and distribution of detected TCRs with T cell subsets.**

(**A**) Ligand-receptor pairs that are differentially expressed between control and mild OSA. (**B**) Ligand-receptor pairs that are differentially expressed between mild and severe OSA. (**C**) UMAP plot shows the overlap and distribution of detected TCRs with T cell subsets. (**D**) The proportion of TCRs with clonal expansion in adenoid T cells of different disease courses (normal snoring, mild, moderate, and severe OSA). (**E**) The top 20 TCR clonotypes (heavy chain CDR3) with clonal expansion.

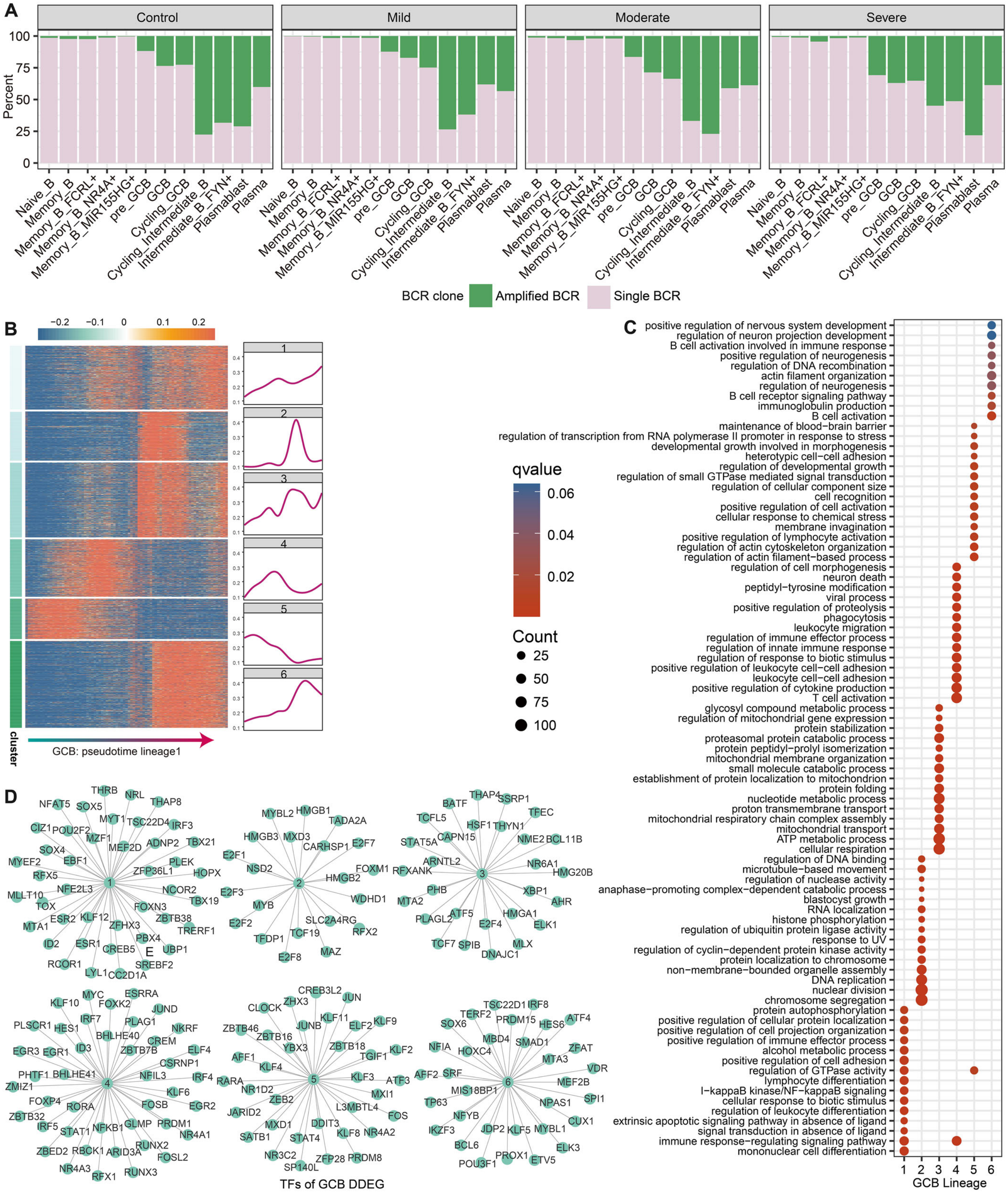
A
Control
Mild
Moderate
Severe
Percent
100
75
50
25
0
Naive_B
Memory_B
Memory_B_FCRL+
Memory_B_NR4A+
Memory_B_MIR155HG+
pre_GCB
GCB
Cycling_GCB
Cycling_Intermediate_B
Intermediate_B_FYN+
Plasmablast
Plasma
BCR clone
Amplified BCR
Single BCR
B
−0.2 −0.1 0 0.1 0.2
1
2
3
4
5
6
cluster
GCB: pseudotime lineage1
C
positive regulation of nervous system development
regulation of neuron projection development
B cell activation involved in immune response
positive regulation of neurogenesis
regulation of DNA recombination
actin filament organization
regulation of neurogenesis
B cell receptor signaling pathway
immunoglobulin production
B cell activation
maintenance of blood−brain barrier
regulation of transcription from RNA polymerase II promoter in response to stress
developmental growth involved in morphogenesis
heterotypic cell−cell adhesion
regulation of developmental growth
regulation of small GTPase mediated signal transduction
regulation of cellular component size
cell recognition
positive regulation of cell activation
cellular response to chemical stress
membrane invagination
positive regulation of lymphocyte activation
regulation of actin cytoskeleton organization
regulation of actin filament−based process
regulation of cell morphogenesis
neuron death
peptidyl−tyrosine modification
viral process
positive regulation of proteolysis
phagocytosis
leukocyte migration
regulation of immune effector process
regulation of innate immune response
regulation of response to biotic stimulus
positive regulation of leukocyte cell−cell adhesion
leukocyte cell−cell adhesion
positive regulation of cytokine production
T cell activation
glycosyl compound metabolic process
regulation of mitochondrial gene expression
protein stabilization
proteasomal protein catabolic process
protein peptidyl−prolyl isomerization
mitochondrial membrane organization
small molecule catabolic process
establishment of protein localization to mitochondrion
protein folding
nucleotide metabolic process
proton transmembrane transport
mitochondrial respiratory chain complex assembly
mitochondrial transport
ATP metabolic process
cellular respiration
regulation of DNA binding
microtubule−based movement
regulation of nuclease activity
anaphase−promoting complex−dependent catabolic process
blastocyst growth
RNA localization
histone phosphorylation
regulation of ubiquitin protein ligase activity
response to UV
regulation of cyclin−dependent protein kinase activity
protein localization to chromosome
non−membrane−bounded organelle assembly
DNA replication
nuclear division
chromosome segregation
protein autophosphorylation
positive regulation of cellular protein localization
positive regulation of cell projection organization
positive regulation of immune effector process
alcohol metabolic process
positive regulation of cell adhesion
regulation of GTPase activity
lymphocyte differentiation
I−kappaB kinase/NF−kappaB signaling
cellular response to biotic stimulus
regulation of leukocyte differentiation
extrinsic apoptotic signaling pathway in absence of ligand
signal transduction in absence of ligand
immune response−regulating signaling pathway
mononuclear cell differentiation
1 2 3 4 5 6
GCB Lineage
qvalue
0.06
0.04
0.02
Count
25
50
75
100
D
TFs of GCB DDEG

Figure EV5. Distribution characteristics of BCR and differentiation trajectory of GCB.

(**A**) The proportion of B cells with clonally expanded BCRs in adenoids of different disease courses (normal snoring, mild, moderate, and severe OSA). (**B**) Heatmap shows the clustering of dynamically differentially expressed genes (DDEGs) along the dynamic changes of germinal center B cells. (**C**) Functional enrichment analysis corresponding to DDEG clusters along the GCB differentiation trajectory. (**D**) The dynamic differential transcription factors are contained in the six clusters of GCB pseudotime differentiation trajectories.

© The Author(s)
