# Supplementary material for: Disease-stage-specific immunometabolic remodeling in pediatric obstructive sleep apnea: a single-cell transcriptomic atlas of adenoid tissue
Source: EMBO Mol Med. 2026 Apr 27;18(6):2483–513. doi: 10.1038/s44321-026-00419-3 (PMC13270133; doi:10.1038/s44321-026-00419-3)
Supplement: Supplementary file 5 — Peer Review File [file 44321_2026_419_MOESM5_ESM.pdf]

EMBO Molecular Medicine

Immunometabolic Rewiring in Pediatric Obstructive Sleep Apnea Adenoids Across Disease Stages: A Single-Cell Atlas

Qin Yang, Yunfei Cui, Xiao Huang, Junlin Liu, Xiaopeng Ma, George Gao, Hongguang Pan, and Shijie Qin

Corresponding authors: Shijie Qin (qinshijie1994@zju.edu.cn) , Hongguang Pan (23xylai1@stu.edu.cn)

Review Timeline:

Submission Date:	22nd Sep 25
Editorial Decision:	17th Oct 25
Revision Received:	22nd Jan 26
Editorial Decision:	16th Feb 26
Revision Received:	17th Mar 26
Accepted:	18th Mar 26

Editor: Zeljko Durdevic

Transaction Report:

(Note: With the exception of the correction of typographical or spelling errors that could be a source of ambiguity, letters and reports are not edited. Depending on transfer agreements, referee reports obtained elsewhere may or may not be included in this compilation. Referee reports are anonymous unless the Referee chooses to sign their reports.)

17th Oct 2025

Dear Dr. Qin,

Thank you for the submission of your manuscript to EMBO Molecular Medicine. We have now received feedback from the two reviewers who agreed to evaluate your manuscript.

As you will see from the reports pasted below, both referees recognize potential interest of the manuscript but also raise serious and partially overlapping concerns particularly regarding descriptive nature of the study, the lack of functional validation and overinterpretation of the data. After the cross-commenting discussion it became clear that the focus of the major revision should be on providing in vitro validation of the main claims of the study. Please pay attention that your conclusions are supported by the data presented. If you would like to discuss further the points raised by the referees, I am available to do so via email or video. Let me know if you are interested in this option.

Further consideration of a revision that addresses reviewers' concerns in full will entail a second round of review. EMBO Molecular Medicine encourages a single round of revision only and therefore, acceptance or rejection of the manuscript will depend on the completeness of your responses included in the next, final version of the manuscript. For this reason, and to save you from any frustrations in the end, I would strongly advise against returning an incomplete revision.

We would welcome the submission of a revised version within three months for further consideration. Please let us know if you require longer to complete the revision.

I look forward to receiving your revised manuscript.

Yours sincerely,

Zeljko Durdevic

Zeljko Durdevic
Senior Editor
EMBO Molecular Medicine

*****

When submitting your revised manuscript, please carefully review the instructions that follow below. We perform an initial quality control of all revised manuscripts before re-review; failure to include requested items will delay the evaluation of your revision.

We require:

1) A .docx formatted version of the manuscript text (including legends for main figures, EV figures and tables). Please make sure that the changes are highlighted to be clearly visible.

2) Individual production quality figure files as .eps, .tif, .jpg (one file per figure). For guidance, download the 'Figure Guide PDF': (https://www.embopress.org/page/journal/17574684/authorguide#figureformat).

3) A .docx formatted letter INCLUDING the reviewers' reports and your detailed point-by-point responses to their comments. As part of the EMBO Press transparent editorial process, the point-by-point response is part of the Review Process File (RPF), which will be published alongside your paper.

4) A complete author checklist, which you can download from our author guidelines (https://www.embopress.org/page/journal/17574684/authorguide#submissionofrevisions). Please insert information in the checklist that is also reflected in the manuscript. The completed author checklist will also be part of the RPF.

5) Please note that all corresponding authors are required to supply an ORCID ID for their name upon submission of a revised manuscript.

6) It is mandatory to include a 'Data Availability' section after the Materials and Methods. Before submitting your revision, primary datasets produced in this study need to be deposited in an appropriate public database, and the accession numbers and database listed under 'Data Availability'. Please remember to provide a reviewer password if the datasets are not yet public (see https://www.embopress.org/page/journal/17574684/authorguide#dataavailability).

In case you have no data that requires deposition in a public database, please state so in this section. Note that the Data Availability Section is restricted to new primary data that are part of this study.

7) For data quantification: please specify the name of the statistical test used to generate error bars and P values, the number (n) of independent experiments (specify technical or biological replicates) underlying each data point and the test used to calculate p-values in each figure legend. The figure legends should contain a basic description of n, P and the test applied. Graphs must include a description of the bars and the error bars (s.d., s.e.m.). See also 'Figure Legend' guidelines: https://www.embopress.org/page/journal/17574684/authorguide#figureformat

8) At EMBO Press we ask authors to provide source data for the main manuscript figures. You will receive a separate email with instructions for providing source data with your revised manuscript, including how to upload and organize the files.

9) Our journal encourages inclusion of *data citations in the reference list* to directly cite datasets that were re-used and obtained from public databases. Data citations in the article text are distinct from normal bibliographical citations and should directly link to the database records from which the data can be accessed. In the main text, data citations are formatted as follows: "Data ref: Smith et al, 2001" or "Data ref: NCBI Sequence Read Archive PRJNA342805, 2017". In the Reference list, data citations must be labeled with "[DATASET]". A data reference must provide the database name, accession number/identifiers and a resolvable link to the landing page from which the data can be accessed at the end of the reference. Further instructions are available at .

10) We replaced Supplementary Information with Expanded View (EV) Figures and Tables that are collapsible/expandable online. A maximum of 5 EV Figures can be typeset. EV Figures should be cited as 'Figure EV1, Figure EV2" etc... in the text and their respective legends should be included in the main text after the legends of regular figures.

- For the figures that you do NOT wish to display as Expanded View figures, they should be bundled together with their legends in a single PDF file called *Appendix*, which should start with a short Table of Content. Appendix figures should be referred to in the main text as: "Appendix Figure S1, Appendix Figure S2" etc.

- Additional Tables/Datasets should be labeled and referred to as Table EV1, Dataset EV1, etc. Legends have to be provided in a separate tab in case of .xls files. Alternatively, the legend can be supplied as a separate text file (README) and zipped together with the Table/Dataset file.

See detailed instructions here:

.

11) The paper explained: EMBO Molecular Medicine articles are accompanied by a summary of the articles to emphasize the major findings in the paper and their medical implications for the non-specialist reader. Please provide a draft summary of your article highlighting

- the medical issue you are addressing,

- the results obtained and

- their clinical impact.

This may be edited to ensure that readers understand the significance and context of the research. Please refer to any of our published articles for an example.

12) Author contributions: You will be asked to provide CRediT (Contributor Role Taxonomy) terms in the submission system. These replace a narrative author contribution section in the manuscript.

13) A Conflict of Interest statement should be provided in the main text.

14) Every published paper now includes a 'Synopsis' to further enhance discoverability. Synopses are displayed on the journal

webpage and are freely accessible to all readers. They include a short stand first (maximum of 300 characters, including space) as well as 2-5 one-sentences bullet points that summarizes the paper. Please write the bullet points to summarize the key NEW findings. They should be designed to be complementary to the abstract - i.e. not repeat the same text. We encourage inclusion of key acronyms and quantitative information (maximum of 30 words / bullet point). Please use the passive voice. Please attach these in a separate file or send them by email, we will incorporate them accordingly.

Please also suggest a striking image or visual abstract to illustrate your article as a PNG file 550 px wide x 300-600 px high.

15) Include a Reagents and Tools Table as part of the Methods section, which can be downloaded from our author guidelines (https://www.embopress.org/page/journal/17574684/authorguide#structuredmethods)

EMBO Molecular Medicine has a "scooping protection" policy, whereby similar findings that are published by others during review or revision are not a criterion for rejection. Should you decide to submit a revised version, I do ask that you get in touch after three months if you have not completed it, to update us on the status.

***** Reviewer's comments *****

Referee #1 (Comments on Novelty/Model System for Author):

This manuscript contains a large data set of mostly single-cell RNAseq-based gene expression analyses of patient-derived adenoid samples. While this human data is certainly relevant, the manuscript remains entirely descriptive. Hence, one needs to be careful with the (over)interpretation of data without the use of functional assays that would actually prove (at least parts) of the observations and conclusions made by the authors.

Referee #1 (Remarks for Author):

This study presents a detailed single-cell transcriptomic and immune repertoire analysis of adenoid tissues from children, covering the spectrum from normal snoring to mild, moderate, and severe obstructive sleep apnea (OSA). The approach integrates histological, mRNA, and pathway-level data, providing a comprehensive view of immune and metabolic changes that occur with increasing disease severity.
The authors report asynchronous cytokine patterns, alterations in transcriptional regulatory networks, and disruptions in developmental signaling pathways such as Hippo, Wnt, and Notch in severe OSA, along with reduced energy metabolism and ATP synthesis. Immune profiling suggests changes in T cell and B cell function, decreased antigen presentation by innate immune cells, and altered cell-cell communication. T cell and B cell receptor sequencing indicates more frequent infection imprinting and increased germinal center activity, alongside antibody class switching. The authors propose that HIF1A-mediated hypoxic signaling may contribute to the downregulation of key immune molecules, identifying this pathway as a possible therapeutic target. While the dataset provides a valuable reference for understanding cellular processes in adenoid hypertrophy and OSA on the transcriptional scale, several aspects would need to be considered to improve the accuracy and impact of the described findings.

Major Comments:
1. The purely descriptive findings of the manuscript, based on extensive (single-cell) gene expression analyses, are largely consistent with known features of chronic hypoxia and immune modulation. However, the mechanistic interpretations, in particular regarding the role of HIF1A, would require functional validation with data on the protein level or in cell culture setups to strengthen their findings. In line with this, many interpretations and conclusions need to be toned down to avoid overinterpretation of the gene expression data without functional prove.

2. The figures are way too packed with data and the authors are urged to simplify data presentation to the essential minimum and to consider moving parts of the data to the supplement.

3. In Figure 2 A-F as well as page 6 the authors describe changes in six gene modules associated with OSA progression that impact cell proliferation, morphological disorder, and an increase of epithelial cell morphology. This result would benefit from histological assessment of the adenoids resected to visually demonstrate changes in the morphology of the adenoids to validate their findings.

4. The authors state on page 7 of the manuscript that the changes in cytokine expression may drive the transformation from normal snoring to OSA. However, it is not clear how this conclusion is drawn. Changes in cytokine expression my also be the result of OSA rather than the driving factor. Further, the sole assessment of mRNA levels of cytokines may not be enough to draw firm conclusions, as this is lacking protein expression data, which could be obtained from the sera of patients using ELISA or bead-arrays or by flow cytometry on a per cell basis. Cytokines are well-known for their posttranscriptional regulation (Pubmed ID: 18349815).

5. On page 9 line 18 of the manuscript the authors draw the conclusion that the downregulation of IGHA2 is a driver for OSA,

however the authors do not provide enough evidence for this conclusion, and it can't be ruled out that this is the result of OSA rather than a driver. The authors should revise this part.

6. On page 9 line 25 the authors discuss the identification of 15 T cell subsets presented in Figure 4B. How were these genes selected for the assessment of the subtypes? Because, for example, the genes PDCD1 (encoding PD-1), BCL6 and CXCR5 are missing as key identifiers for Tfh cells.

7. Figure 4C shows the change in T cell subsets between different OSA severities and the control group, however it is not evident for the reader which OSA groups are compared to another OSA group and which group shows significant changes in comparison to another group. The authors should improve the description of the figure legend for a better understanding of the context shown in the graph and/or an improved formatting of the shown graph for a better visualization of which group is different compared to which.
7. Interestingly the CD4Tex cells (exhausted cells) are enriched in the control group but show no significant difference in the OSA groups. This is not discussed properly by the authors. Why would healthy controls have an enriched exhausted T cell subcluster?

8. On page 10 line 9 the authors conclude that in the severe OSA group T cell proliferation, activation and differentiation is downregulated, and T cell immunity is weakend only based on the GSEA and RNA-seq data. While this may be the case on the transcriptional level, these findings require confirmation by in vitro assessment of T cells from OSA patients on their capability to mount immune responses. Such relatively simpleexperiments would allow a direct assessment of changes in the function of T cells from different disease severities.

9. The authors describe on page 11 line 4 the upregulation of exhaustion marks such as TOX, TIGIT and CTLA4. It is correct that these markers are associated with exhaustion, however, they are also upregulated on activated cells.

10. On page 11 line 13 DEGs are compared between the mild OSA and severe OSA groups. It stays unclear why only the mild OSA group is compared to the severe and not also the control group. This pertains to the entire manuscript. Why are not all groups compared against each other.

11. Figure 5D-F show changes in the activity of gene sets related to cell migration, phagocytosis, and antigen processing and presentation. However, the authors use for their axis labeling and description of the results in the manuscript (page 12 lines 1-9) the terms leukocyte migration, phagocytosis and antigen processing and presentation. The provided results only show a down or up regulation of gene sets related to these processes but not the processes themselves. Again, in vitro experiments would be required to portray these results as such. The authors should revise the description of the data to accurately depict the shown information or actually include functional data. The authors should also include the description of the statistical comparison in the figure ledgend of figure 5D-F.

12. On page 12, line 30 and following, the authors state that:" although the proportion of myeloid cells is very low, they showed many important immune related changes in the progression of OSA". Again, this interpretation is only based on gene expression changes. It remains unknown if myeloid cells play an actual role in the formation of OSA or changes in their gene sets are the result of OSA rather than a driver of it.

13. On page 13 lines 20-22 the authors conclude that the increased diversity of pathogen epitopes from TCRs means that they are more susceptible to infections. This finding can't be drawn in such a direct manner as the authors are not aware of the complete disease history of the assessed children. Furthermore, it can't be ruled out that infections with these pathogens themselves induced the hypoplasia of the adenoids.

14. The authors assess mRNA levels by qPCR in several different figures (Figure 2-5); however, the analysis of the different groups stays unclear. Between different bar graphs in the same figure (see Figure 4 K and N) the comparison of groups changes without stating it in the manuscript or the figure legend. In some plots ns results are shown in other not. This leaves the reader confused and unclear on what has been compared by statistical testing.

Minor Comments
15. Please include page and line numbers in the manuscript file.
16. Page 5, line 19: Please revise the sentence for proper English.
17. The results part would benefit from a better description of the B cell type "intermediate B cell".
18. In Figure 3C, D the authors show enrichment analysis network diagrams. The figure legend should be revised for better clarity on what is shown and how.
19. Page 8 line 21: The authors recapitulate their findings and state that "the energy required by cells are impaired". Energy itself can't be impaired, Please revise, e.g. to "energy balance".
20. On page 9 line 24 the section starts with "This result identified...". However, it stays unclear which result is meant in this sentence. Please revise.
21. The authors describe on page 11 line 3 the change in exhaustion markers, however it is not clear what "it" is in the following sentence:" ..., but it up-regulated exhaustion markers...".

22. Pages 5 line 19: Please revise the sentence for proper English grammar.
23. Page 9 line 31: Please correct "CD4fh" to "CD4Tfh".
24. The authors mix their terminology for genes and proteins throughout the manuscript. Human genes are written using HGNC-approved symbols in italics and uppercase (for example PDCD1, CXCR5), while the corresponding proteins use the same symbols or established antigen names in non-italic uppercase (for example PD-1/CD279).
25. In Figure 5 J the data points and error bar of the mild group seem to disappear behind the bar. Please adjust the graph. This should be revised again thought the whole manuscript.

Referee #2 (Comments on Novelty/Model System for Author):

Yang et al.'s study provided the single-cell transcriptome and immune repertoire data of OSA with different severity levels. They found that children with severe OSA exhibit significant immune and metabolic disorders, especially the increased infection imprints and impaired antibody maturation provided by BCR and TCR data, which seem to explain their immune decline. The study also proposed potential mechanisms for cellular communication dysfunction and HIF1A-HLA inhibition axis. These data results seem to have a good match and assistance in explaining the clinical symptoms of OSA in children, and could be considered for publication. However, there are some areas in the study that need to be revised or further explained.

(1)The author describes the HE results of eosinophils and neutrophils, but why are these two types of cells not included in the single-cell sequencing results? This requires a reasonable explanation.
(2)As mentioned above, there is a lack of necessary discussion on the impact of changes in granulocytes on the development of OSA, which at least needs to be strengthened in the discussion section.
(3)Why was the mfuzz trend analysis in Figure 2 not included in the control group for analysis.
(4)What is the reference for the changes in cell proportion involved in the study, and why is it not compared with the control?
(5)The decrease in immune cell communication in severe OSA shown in Figure 5 is interesting, but there seems to be a lack of specific changes in ligand receptor molecular results.
(6)The TCR imprint analysis results in Figure 6 seem to lack significant statistical significance, and the same issue also appears in the SHM analysis results.
(7)The author mentioned that HIF1A inhibits HLA or interferon signaling, but this is mainly based on correlation and some literature results. At least more discussion is needed here to support this result.
(8)Although important and novel enough, the results of this study are mainly descriptive analysis, so excessive interpretation needs to be improved.
(9)The terms "adenoidal hypertrophy" and "adenoid hypertrophy" are used interchangeably. Please use abbreviation or choose one and use it consistently throughout the manuscript.Similarly, "scRNA-seq", "single-cell RNA-seq" and "single cell RNA-seq" are all used. "scRNA-seq", "scTCR/BCR-seq" and "scTCR/scBCR sequencing" and "Single cell V(D)J-seq" are all used. "scRNA-seq".
(10)Check the usage specifications of acronyms (e.g., polysomnography, HSP and PBMCs) and providing a acronyms list is better.
(11)In the discussion section of the article, the results of differentially expressed genes and other articles have been explored and studied in existing literature. Currently, the main focus is on gene functions, but the association value with diseases such as OSA is not clear.
(12) Considering that the article has collected and studied samples of different disease degrees, it is suggested that the discussion should strengthen the exploration of the occurrence and progression of OSA to highlight the value of this research in the fine classification of disease degrees.

Referee #1 (Comments on Novelty/Model System for Author):

This manuscript contains a large data set of mostly single-cell RNAseq-based gene expression analyses of patient-derived adenoid samples. While this human data is certainly relevant, the manuscript remains entirely descriptive. Hence, one needs to be careful with the (over)interpretation of data without the use of functional assays that would actually prove (at least parts) of the observations and conclusions made by the authors.

Response: Thank you for your constructive comments. Based on your suggestions, we have made significant revisions to the manuscript. On one hand, we have removed many excessive descriptions and interpretations, and have drawn conclusions as carefully as possible. On the other hand, we have added as many experimental validations as possible using the remaining precious and limited adenoid samples. Looking forward to these revisions meeting your requirements, thank you.

Referee #1 (Remarks for Author):

This study presents a detailed single-cell transcriptomic and immune repertoire analysis of adenoid tissues from children, covering the spectrum from normal snoring to mild, moderate, and severe obstructive sleep apnea (OSA). The approach integrates histological, mRNA, and pathway-level data, providing a comprehensive view of immune and metabolic changes that occur with increasing disease severity.

The authors report asynchronous cytokine patterns, alterations in transcriptional regulatory networks, and disruptions in developmental signaling pathways such as Hippo, Wnt, and Notch in severe OSA, along with reduced energy metabolism and ATP synthesis. Immune profiling suggests changes in T cell and B cell function, decreased antigen presentation by innate immune cells, and altered cell-cell communication. T cell and B cell receptor sequencing indicates more frequent infection imprinting and increased germinal center activity, alongside antibody class switching. The authors propose that HIF1A-mediated hypoxic signaling may contribute to the downregulation of key immune molecules, identifying this pathway as a possible therapeutic target. While the dataset provides a valuable reference for understanding cellular processes in adenoid hypertrophy and OSA on the transcriptional scale, several aspects would need to be considered to improve the accuracy and impact of the described findings.

Response: Thank you very much for your detailed and constructive comments on the manuscript and your interest in our research. We have made revisions one by one according to your suggestions, which have greatly improved the manuscript. We look forward to meeting your requirements. Thank you again.

Major Comments:

1. The purely descriptive findings of the manuscript, based on extensive (single-cell) gene expression analyses, are largely consistent with known features of chronic hypoxia and immune modulation. However, the mechanistic interpretations, in particular regarding the role of HIF1A, would require functional validation with data on the protein level or in cell culture setups to strengthen their findings. In line with this, many interpretations and conclusions need to be toned down to avoid overinterpretation of the gene expression data without functional prove.

Response: Thank you for your comment. We have supplemented the protein level experiment of HIF1A (immunofluorescence) and indeed found that HIF1A is significantly upregulated in the severe OSA group compared to the control and mild OSA groups. We have added a detailed discussion on the immune suppression mechanism mediated by HIF1A, as this part of the results has been confirmed in previous published studies that hypoxia induced upregulation of HIF1A at the cellular level can lead to downregulation of HLA molecules and immune signals (PMID: 34456926, PMID: 38920249, PMID: 18694997). These studies overall indicate that hypoxia induced HIF1A inhibits immune activation and maturation of dendritic cells, monocytes, and T cells at least at the cell culture level (PMID: 34456926, PMID: 38920249, PMID: 18694997). Our adenoid sequencing data indeed showed similar mRNA levels, and HIF1A showed a significant negative correlation with immune signaling. Therefore, we propose that similar HIF1A-mediated immune suppression may exist in children with real adenoid hypertrophy. However, considering that our focus is mainly on analyzing the correlation between single-cell omics data of different OSA severity levels and clinical outcomes, we can only provide a reasonable and sufficient discussion on this mechanism, and look forward to further mechanism research along these clues by future researchers. Finally, we have removed excessive descriptions and interpretations of gene expression data as much as possible. Please refer to the revised manuscript for details, thanks again.

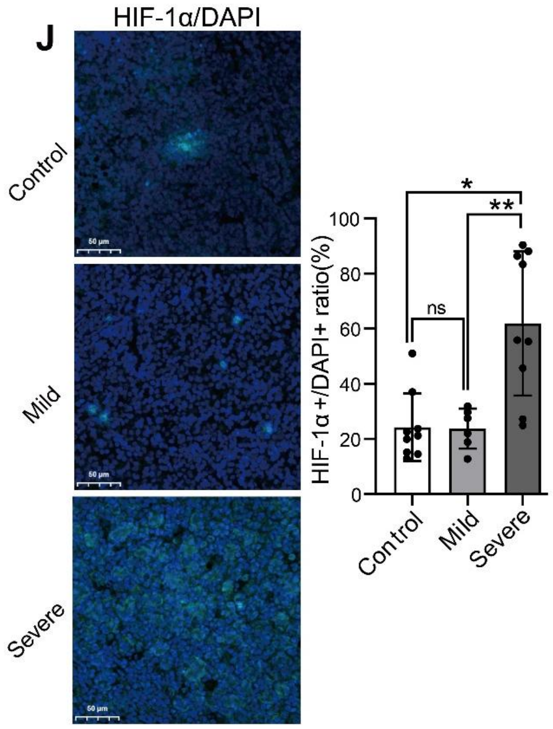

Revised Figure 1J: Immunofluorescence assay showed the protein expression level of HIF1A in adenoid tissue.

2. The figures are way too packed with data and the authors are urged to simplify data presentation to the essential minimum and to consider moving parts of the data to the supplement.

Response: Thank you for your comment. We have simplified the main image as much as possible and moved some results to the supplementary section. Please refer to the revised manuscript and supplementary materials.

3. In Figure 2 A-F as well as page 6 the authors describe changes in six gene modules associated with OSA progression that impact cell proliferation, morphological disorder, and an increase of epithelial cell morphology. This result would benefit from histological assessment of the adenoids resected to visually demonstrate changes in the morphology of the adenoids to validate their findings.

Response: Thank you for your comment. We have supplemented the detailed histological evaluation and statistics of the pathological sections, and combined these results with the discovery of the gene module to validate the results. Please refer to the revised manuscript for details, thank you.

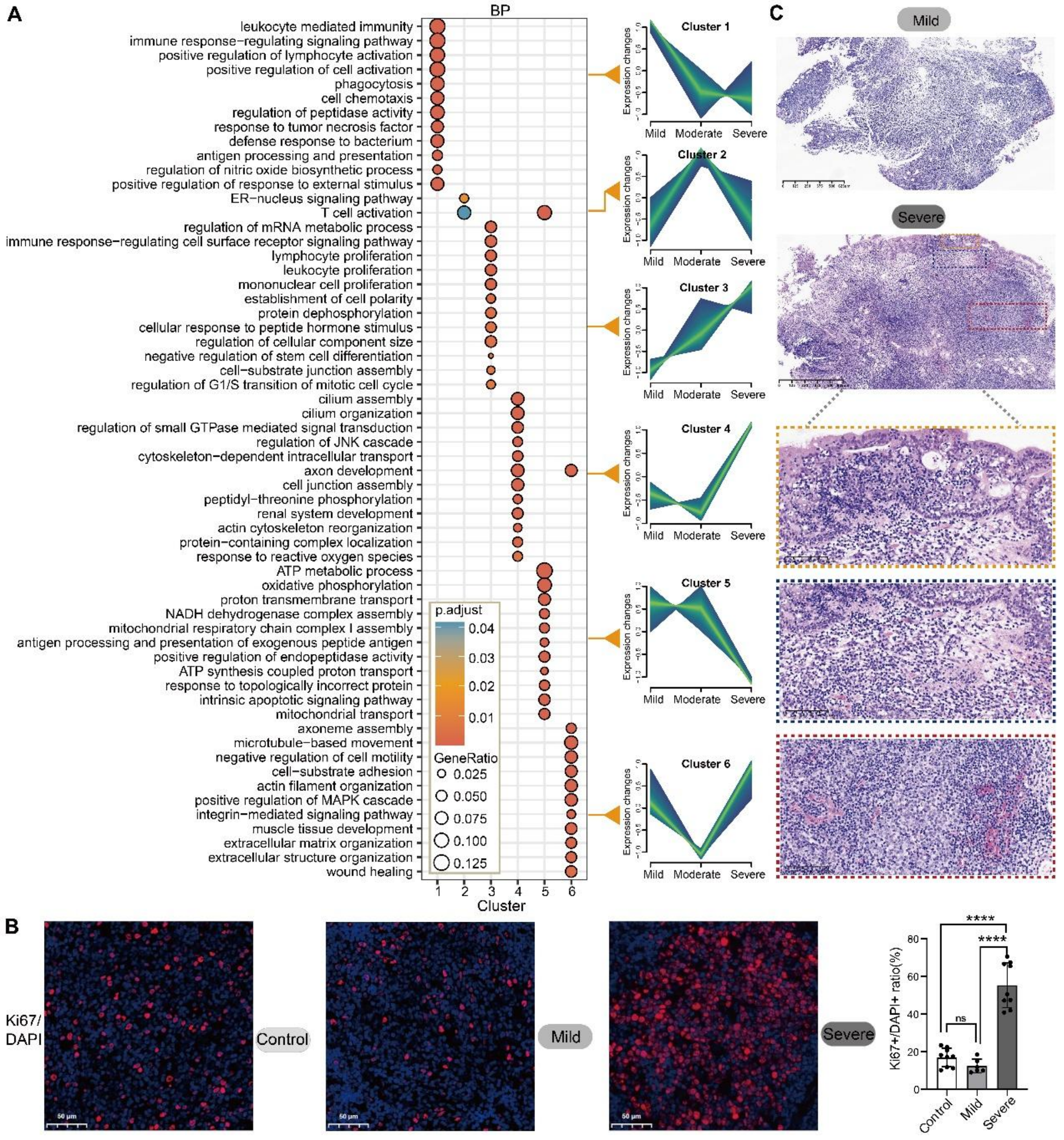

Revised Figure 2A-C: HE staining and immunofluorescence assay showed the tissue disorder and proliferation of severe adenoids. (A) Clustering and functional enrichment analysis of expression profiles of adenoids with different severities of OSA based on fuzzy clustering algorithm. Six clusters of dynamically differentially expressed genes and functional modules were identified along the progression of OSA. (B) Immunofluorescence showed the protein expression levels of Ki67 in adenoid tissues of the control, mild and severe OSA groups. Representative immunofluorescence images were shown, DAPI (blue), HIF-1α (red), 20×, scale bar: 50 μm. Samples were collected from the control group (n=3), the mild group (n=2), and the severe group (n=3). For each sample within each group, three visual fields of equal area were randomly selected for quantification, and showed as mean ± SEM, ns > 0.05, ****$p < 0.0001$. Statistical testing

using one-way analysis of variance. (C) Histopathological features of adenoid tissues with varying degrees of OSA. Low-magnification (4×) views demonstrating mild and moderate/severe OSA in the upper part. High-magnification (20×) views of areas outlined, yellow box: epithelial hyperplasia with neutrophilic infiltration and focal cystic change; blue box: subepithelial edema infiltrated by lymphocytes and plasma cells; red box: hyperemia and hemorrhage within the adenoid parenchyma.

4. The authors state on page 7 of the manuscript that the changes in cytokine expression may drive the transformation from normal snoring to OSA. However, it is not clear how this conclusion is drawn. Changes in cytokine expression my also be the result of OSA rather than the driving factor. Further, the sole assessment of mRNA levels of cytokines may not be enough to draw firm conclusions, as this is lacking protein expression data, which could be obtained from the sera of patients using ELISA or bead-arrays or by flow cytometry on a per cell basis. Cytokines are well-known for their posttranscriptional regulation (Pubmed ID: 18349815).

Response: Thank you for your comment. We compared the cytokine differences between control and mild OSA with severe OSA through analysis. Although we speculate that the differences in cytokines between the control and mild OSA groups may drive the occurrence of early OSA. But as you said, these cytokines are indeed likely only a result of early OSA, rather than driving factors. We fully agree with your opinion. Due to the lack of more in-depth causal evidence experiments and corresponding serum samples, we have revised this description. The revised description only emphasizes the association between these cytokines and the occurrence of early OSA, rather than a causal relationship. Thank you again for your suggestion. Please refer to the revised manuscript.

5. On page 9 line 18 of the manuscript the authors draw the conclusion that the downregulation of IGHA2 is a driver for OSA, however the authors do not provide enough evidence for this conclusion, and it can't be ruled out that this is the result of OSA rather than a driver. The authors should revise this part.

Response: Thank you for your comment. We fully agree with your comment and have made revisions to similar excessive descriptions. The revised manuscript only highlights the association

between IGHA2 and OSA, rather than directly describing it as a driving factor for OSA occurrence, without further causal evidence. Meanwhile, we also look forward to future researchers conducting more in-depth research and causal verification based on these clues. Please refer to the revised manuscript, thanks again.

6. On page 9 line 25 the authors discuss the identification of 15 T cell subsets presented in Figure 4B. How were these genes selected for the assessment of the subtypes? Because, for example, the genes PDCD1 (encoding PD-1), BCL6 and CXCR5 are missing as key identifiers for Tfh cells.

Response: Thank you for your comment. When identifying cell subpopulations, we integrated the Azimuth multimodal artificial intelligence algorithm, cluster specific highly expressed genes, and classical T cell markers to determine. As you mentioned, PDCD1, BCL6, and CXCR5 are indeed biomarkers of Tfh cells, and in our supplementary analysis results, they are also specifically expressed in CD4Tfh. We have added three markers to Figure 4B. It should be pointed out that the IL6ST, TOX, TOX2, and ICA1 we used are also CD4Tfh markers (PMID: 30479382, PMID: 34914499), and their baseline expression levels rank at the top of cluster specific highly expressed genes.

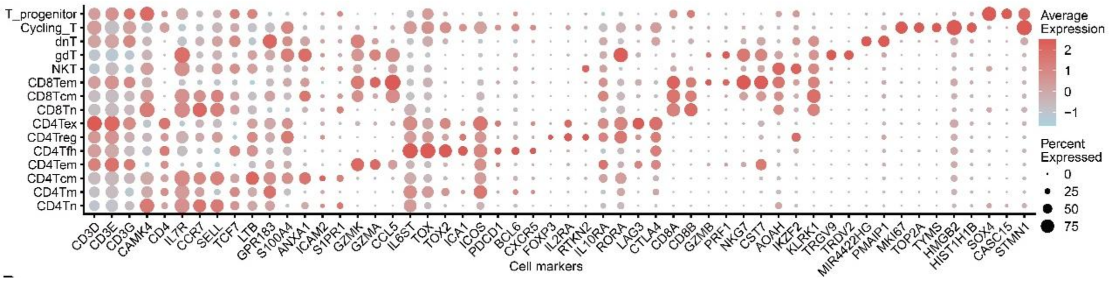

Revised Figure 4B: Distribution of marker gene expression corresponding to CD4tfh.

7. Figure 4C shows the change in T cell subsets between different OSA severities and the control group, however it is not evident for the reader which OSA groups are compared to another OSA group and which group shows significant changes in comparison to another group. The authors should improve the description of the figure legend for a better understanding of the context shown in the graph and/or an improved formatting of the shown graph for a better visualization of which group is different compared to which.

7. Interestingly the CD4Tex cells (exhausted cells) are enriched in the control group but show no significant difference in the OSA groups. This is not discussed properly by the authors. Why

would healthy controls have an enriched exhausted T cell subcluster?

Response: Thank you for your comment. We apologize for the unclear caption description. Figure 4C mainly calculates the ratio of the expected cell number to the actual cell number, and then calculates the degree of cell enrichment (PMID: 30479382). For example, for a certain cell type in a certain group, the number of this cell type in the remaining three groups is used as a control for calculation. In a word, it calculates the degree of enrichment or reduction of a single group relative to the other three groups. We have revised the detailed description in the figure legend.

In addition, the control group did observe a slight enrichment of CD4Tex cells compared to the other three groups. This may be because our control group samples also experienced adenoid hypertrophy but did not progress to OSA (normal snoring group), rather than truly healthy samples (healthy children samples cannot have adenoid tissue removed). This means that these control samples themselves also have certain disease states, so the enrichment of CD4Tex cells may reflect the characteristics of children with simple adenoid hypertrophy. We have added this description to the revised manuscript. Please refer to the revised manuscript. Thank you.

8. On page 10 line 9 the authors conclude that in the severe OSA group T cell proliferation, activation and differentiation is downregulated, and T cell immunity is weakend only based on the GSEA and RNA-seq data. While this may be the case on the transcriptional level, these findings require confirmation by in vitro assessment of T cells from OSA patients on their capability to mount immune responses. Such relatively simple experiments would allow a direct assessment of changes in the function of T cells from different disease severities.

Response: Thank you for your comment. We fully agree with your viewpoint that changes in mRNA levels may not be consistent with changes in protein levels, and even based on our experience and previous literature, only about 30%~40% of mRNA and protein levels exhibit a consistent pace of change across the entire genome. In our results, we did use methods such as gene set scoring, GSEA analysis, and direct visualization comparison of key genes (T cell toxicity markers) to conclude that T cell activity in the severe OSA group decreased at the mRNA level. Based on your suggestion, we conducted immunofluorescence experiments on CD3, KI67, and IFNγ using the remaining adenoid sample tissues. The results were not completely consistent with mRNA, and immunofluorescence experiments showed that the levels of CD3 and KI67 proteins

were upregulated in the severe OSA group, indicating that there was indeed T lymphocyte proliferation in the severe OSA group, which was expected. IFNγ, which is used to indicate T cell functional activity, was significantly downregulated in mild OSA compared to the control group and severe OSA group, while only slightly downregulated in severe OSA group compared to the control group. In summary, these results indicate that the pace of expression changes at the mRNA and protein levels is indeed not completely consistent, suggesting that there may be strong post transcriptional modifications of mRNA or possible feedback and compensation mechanisms in the development of OSA. In addition, this inconsistency may also be related to the fact that immunofluorescence detection results cannot fully represent the protein expression levels of single-cell populations. Nevertheless, we still believe that when the faucet mRNA upstream of the protein is continuously down regulated, it is difficult to maintain the upregulation or stable expression of the protein level for a long time despite the existence of post transcriptional regulatory factors. Finally, in order to reduce excessive description, we have emphasized again in the result description that it is only at the mRNA level. Please refer to the revised manuscript for details. Thank you.

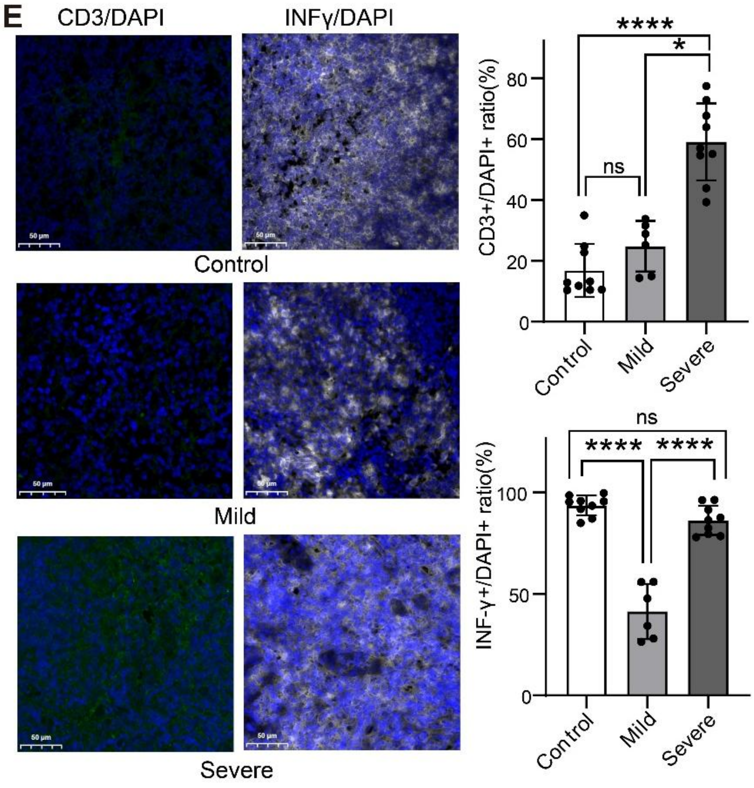

Revised Figure EV3E: Immunofluorescence assay showed the protein expression levels of CD3 and IFNγ (markers of T cell function and activity) in different OSA groups.

9. The authors describe on page 11 line 4 the upregulation of exhaustion marks such as TOX, TIGIT and CTLA4. It is correct that these markers are associated with exhaustion, however, they are also upregulated on activated cells.

Response: Thank you for your comment. We fully agree with your perspective. Typically, markers associated with T cell exhaustion are not only upregulated in exhausted T cells but are also often increased in activated or inflammatory T cells. In many cases, T cell exhaustion markers actually accompany T cell activation, this is a normal biological phenomenon, as activated T cells primarily undergo apoptosis or enter an exhausted state after fulfilling their functions. According to our results, TOX, TIGIT, and CTLA4 are upregulated in the moderate OSA group, while in the severe OSA group, their expression decreases again to levels close to those in the normal snoring and mild OSA groups. This precisely reflects the reduced functional activity and immune decline of T cells in the severe OSA group, at least at the mRNA level. These findings may also explain the clinically observed decline in immunity and increased susceptibility to infections among children with severe OSA. Finally, following your suggestions, we have revised the description of these results in the manuscript accordingly.

10. On page 11 line 13 DEGs are compared between the mild OSA and severe OSA groups. It stays unclear why only the mild OSA group is compared to the severe and not also the control group. This pertains to the entire manuscript. Why are not all groups compared against each other.

Response: Thank you for your comment. We apologize for the incomplete description of the results caused by the complex grouping of differentially expressed genes and have provided detailed supplements. In fact, we did calculate the differentially expressed genes compared between two groups, and these complete results are presented in Supplementary Tables S5, 6, and 9. However, due to the large number of groups (6 differentially expressed genes), a complete display requires more space and can easily lead to redundancy and complexity. Therefore, we chose to focus on describing the differentially expressed genes between the control group and the mild OSA group, as well as between the mild and severe OSA groups, in Figure 4 (On page 11 line). We focus on describing these two groups because the differences between the control group (normal snoring) and the mild OSA group better reflect events related to early onset of OSA, while the differential genes between the mild and severe OSA groups better reflect events or biological

processes related to the aggravation of OSA disease. We did not focus on describing the results of the moderate OSA group because they were between mild and severe, and were not very stable or the differences may not be significant enough. Finally, considering that as a resource type article, we strive to provide a complete differential gene dataset in the supplementary materials for researchers in the OSA field to review and use. Thanks again.

11. Figure 5D-F show changes in the activity of gene sets related to cell migration, phagocytosis, and antigen processing and presentation. However, the authors use for their axis labeling and description of the results in the manuscript (page 12 lines 1-9) the terms leukocyte migration, phagocytosis and antigen processing and presentation. The provided results only show a down or up regulation of gene sets related to these processes but not the processes themselves. Again, in vitro experiments would be required to portray these results as such. The authors should revise the description of the data to accurately depict the shown information or actually include functional data. The authors should also include the description of the statistical comparison in the figure legend of figure 5D-F.

Response: Thank you for your comment. We agree with your point that the upregulation or downregulation of gene set activity at the mRNA level cannot fully represent the upregulation or downregulation of the biological processes represented by these gene sets themselves. Therefore, we have revised the result description to limit the relevant inferences to the mRNA level.

It should be pointed out that although there are various possible regulatory factors between mRNA and protein levels, according to the central dogma and logical inference, we still insist that when mRNA levels decrease, protein levels are difficult to maintain stable expression or upregulation. Therefore, it is largely reasonable to use changes in mRNA levels to characterize the biological processes represented by a certain gene or gene set. In fact, even protein levels cannot fully represent changes in biological processes themselves, especially changes in individual or few proteins. In addition, gene set scoring or GSEA analysis, as common and widely recognized analytical methods, are often used to evaluate the final direction of changes in a biological process or signaling pathway. This is mainly because many biological processes or pathways are often regulated by a group of related genes, so changes based on a single gene or protein are difficult to represent changes in the entire biological process or pathway. For example, it is difficult to

imagine that when most genes in the antigen presentation and processing pathway are downregulated or inhibited at the mRNA level, the actual antigen processing efficiency remains unchanged or even upregulated.

In addition, we have added statistical descriptions of Figures 5D-F in the caption. Please refer to the revised manuscript, thank you.

12. On page 12, line 30 and following, the authors state that:" although the proportion of myeloid cells is very low, they showed many important immune related changes in the progression of OSA". Again, this interpretation is only based on gene expression changes. It remains unknown if myeloid cells play an actual role in the formation of OSA or changes in their gene sets are the result of OSA rather than a driver of it.

Response: Thank you for your comment. Based on your suggestion, we have revised the relevant results to limit their description and inference to the mRNA level, and weakened the description of causal relationships. In the future, we also look forward to more protein level related research being conducted to support and further deepen these studies based on our single-cell atlas clues at the mRNA level. Thanks again.

13. On page 13 lines 20-22 the authors conclude that the increased diversity of pathogen epitopes from TCRs means that they are more susceptible to infections. This finding can't be drawn in such a direct manner as the authors are not aware of the complete disease history of the assessed children. Furthermore, it can't be ruled out that infections with these pathogens themselves induced the hypoplasia of the adenoids.

Response: Thank you for your comment. It is reasonable to infer that children with severe OSA have experienced more infections based on more pathogen related TCR annotations and imprints. Because when infected with viruses or bacteria, the TCR in the body forms memories and gradually accumulates. However, we also agree with your point of view that this only represents that they have indeed experienced more infections compared to other groups, and does not mean that these severely OSA children have been more susceptible to infections due to weakened immunity. What we observed was only a potential correlation between TCR infection imprints and the severe group, rather than a direct driving factor. Therefore, we have revised the excessive

description and only provided appropriate descriptions in the discussion section.

Furthermore, it is reasonable that the pathogen infection you mentioned may itself lead to underdeveloped adenoids. In fact, one of the main driving factors for adenoid hypertrophy is repeated pathogenic infections or other stimuli, which lead to the accumulation of chronic inflammation and abnormal proliferation of adenoids. We have discussed this in the revised manuscript. Please refer to the revised manuscript. Thank you.

14. The authors assess mRNA levels by qPCR in several different figures (Figure 2-5); however, the analysis of the different groups stays unclear. Between different bar graphs in the same figure (see Figure 4 K and N) the comparison of groups changes without stating it in the manuscript or the figure legend. In some plots ns results are shown in other not. This leaves the reader confused and unclear on what has been compared by statistical testing.

Response: Thank you for your comment. We apologize for the unclear caption description. Based on your suggestion, we have revised the inter group statistics of all qPCR validation results and made unified revisions to the captions. Please refer to the revised content, thank you.

Minor Comments

15. Please include page and line numbers in the manuscript file.

Response: Thank you for your comment. We have added line numbers and page numbers.

16. Page 5, line 19: Please revise the sentence for proper English.

Response: Thank you for your comment. We have revised this, please refer to the revised manuscript.

17. The results part would benefit from a better description of the B cell type "intermediate B cell".

Response: Thank you for your comment. We have rephrased the results of this section as follows: “As the main component of adenoids, we identified 12 B-cell subpopulations, including germinal center B (GCB) ($NEIL1^+$/$RGS13^+$), memory B ($CD27^+$/$TNFRSF13B^+$), plasma cells ($MZB1^+$/$JCHAIN^+$), intermediate B cells (IGHD+/$CD27^+$), and naive B cells ($IL4R^+$/$TCL1A^+$) in

different states (Fig. 3A, B and Fig. S3A). These B-cell subpopulations exhibit three differentiation trajectories: from naive B to GCB, from naive B to intermediate B, and from naive B to plasma cells (Fig. S3B). Cell abundance analysis indicates that the intermediate B cells are decreased in the severe OSA group, while GCB shows an increasing trend in the moderate OSA group (Fig. S3C). Pseudo-time analysis also reveals that the decline along the intermediate B cell differentiation trajectory is most pronounced in the severe OSA group (Fig. S3D). Importantly, intermediate B cells are considered to have versatile functions due to their potential to differentiate into memory B or plasma cells, and can also directly become terminally differentiated but low neutralizing cells in certain disease environments. Thus, the reduction of intermediate B cells may be associated with the progression of OSA."

18. In Figure 3C, D the authors show enrichment analysis network diagrams. The figure legend should be revised for better clarity on what is shown and how.

Response: Thank you for your comment. The revised captions for Figures 3C, D are as follows: "The network diagram displays the functional modules enriched by the top shared differentially expressed genes that are upregulated (C) or downregulated (D) between B-cell subsets in the control versus the mild OSA or the mild OSA versus the severe OSA. The upper part of the network represents the enriched biological processes or pathways and their interrelationships. The lower part of the network illustrates the overlap of functional networks enriched from the two sets of differentially expressed genes (red: control vs. mild OSA; blue: mild OSA vs. severe OSA)."

19. Page 8 line 21: The authors recapitulate their findings and state that "the energy required by cells are impaired". Energy itself can't be impaired, Please revise, e.g. to "energy balance".

Response: Thank you for your comment. According to your suggestion, the revised description is as follows: "These results strongly suggest that the B cell immune function of children with severe OSA is impaired at the mRNA level, and their intracellular ATP production and energy balance may be disrupted"

20. On page 9 line 24 the section starts with "This result identified...". However, it stays unclear which result is meant in this sentence. Please revise.

Response: Thank you for your comment. The revised description is as follows: "In the analysis of T cell subsets in children's adenoids, 15 subsets were identified, including CD4T ($CD3D^{+}/CD4^{+}$) and CD8T ($CD3D^{+}/CD8^{+}$) cells in different states, double-negative T cells (dnT) ($CD3D^{+}/CD4^{-}/CD8^{-}$), cycling T cells ($CD3D^{+}/MKI67^{+}$), NKT cells ($AOAH^{+}/IKZF2^{+}$), and γδT cells ($TRGV9^{+}/TRDV2^{+}$) and other non-classical T cells."

21. The authors describe on page 11 line 3 the change in exhaustion markers, however it is not clear what "it" is in the following sentence:" ..., but it up-regulated exhaustion markers...".

Response: Thank you for your comment. The revised description is as follows: "The mRNA levels of T cell cytotoxicity-associated markers in the moderate OSA group were overall comparable to those in the control group. However, the moderate OSA group exhibited upregulation of multiple T cell exhaustion-related genes such as TOX, TIGIT, and CTLA4 compared to the control group."

22. Pages 5 line 19: Please revise the sentence for proper English grammar.

Response: Thank you for your comment. The revised description is as follows: "In addition, we also identified a subset of conventional epithelial cells ($KRT19^{+}/KRT17^{+}$) and epithelial cells with high expression of FDCSP, the latter of which has been reported in the tonsillar single-cell atlas. (Fig. 1E, F) [9]. Interestingly, these epithelial cells showed enrichment in the severe group (Fig. 1G), and studies have found that proliferation of adenoid epithelial cells can lead to hypertrophy and OSA, although the proportion is lower [4, 10]."

23. Page 9 line 31: Please correct "CD4fh" to "CD4Tfh".

Response: Thank you for your comment. CD4fh has been changed to CD4Tfh in the description and figure. Please refer to the revised manuscript.

24. The authors mix their terminology for genes and proteins throughout the manuscript. Human genes are written using HGNC-approved symbols in italics and uppercase (for example PDCD1, CXCR5), while the corresponding proteins use the same symbols or established antigen names in non-italic uppercase (for example PD-1/CD279).

Response: Thank you for your comment. We have revised the format of gene and protein names

uniformly. Please refer to the revised manuscript. Thank you.

25. In Figure 5 J the data points and error bar of the mild group seem to disappear behind the bar. Please adjust the graph. This should be revised again thought the whole manuscript.

Response: Thank you for your comment. We have revised this, please refer to the revised image, thank you.

Referee #2 (Comments on Novelty/Model System for Author):

Yang et al.'s study provided the single-cell transcriptome and immune repertoire data of OSA with different severity levels. They found that children with severe OSA exhibit significant immune and metabolic disorders, especially the increased infection imprints and impaired antibody maturation provided by BCR and TCR data, which seem to explain their immune decline. The study also proposed potential mechanisms for cellular communication dysfunction and HIF1A-HLA inhibition axis. These data results seem to have a good match and assistance in explaining the clinical symptoms of OSA in children, and could be considered for publication. However, there are some areas in the study that need to be revised or further explained.

Response: Thank you very much for your comments and recognition of our manuscript. We have made revisions based on your suggestions, which have greatly improved the quality of the manuscript. We look forward to these revisions meeting your requirements. Thank you again.

(1)The author describes the HE results of eosinophils and neutrophils, but why are these two types of cells not included in the single-cell sequencing results? This requires a reasonable explanation.

Response: Thank you for your comment. The main reasons for the failure to detect granulocytes in the single-cell sequencing results are as follows: First, as a lymphoid tissue, adenoids typically exhibit minimal granulocyte infiltration. The small number of granulocytes observed in HE staining may not have been adequately sampled in the single-cell preparation, which we consider the primary reason for their absence in the results. Second, granulocytes are prone to apoptosis or rupture during the isolation process, making it difficult for conventional single-cell sequencing to capture intact granulocytes, especially when they are present in low numbers. Third, granulocytes generally exhibit very low gene counts and UMI counts, which likely led to their being filtered out during data analysis or being too scarce to form distinct clusters. In conclusion, targeted analysis of granulocytes in adenoid tissue may require specialized enrichment strategies and customized analytical filtering methods to achieve effective capture. In addition, we have added the limitation of granulocytes in discussion section. Thank you.

(2)As mentioned above, there is a lack of necessary discussion on the impact of changes in granulocytes on the development of OSA, which at least needs to be strengthened in the

discussion section.

Response: Thank you for your comment. We have conducted supplementary discussions on the role of eosinophils and neutrophils in the occurrence of adenoid hypertrophy and OSA during the discussion session. Please refer to the revised manuscript, thank you.

(3)Why was the mfuzz trend analysis in Figure 2 not included in the control group for analysis.

Response: Thank you for your comment. In the Mfuzz clustering analysis of Figure 2, our main goal is to identify gene clusters and functional modules associated with an increase in OSA severity. Therefore, we only apply mild, moderate, and severe OSA for analysis to ensure the purity of the results. Considering that our control data comes from normal snoring, the differences between it and early mild OSA may not represent the molecular events associated with OSA exacerbation, so we did not include the control. However, the differences and changes between the normal snoring group and the early mild OSA group can still be found in other outcome stages. Thanks again.

(4)What is the reference for the changes in cell proportion involved in the study, and why is it not compared with the control?

Response: Thank you for your comment. We apologize for the unclear caption description of changes in cell abundance. When analyzing changes in cell abundance, the ratio of the expected cell number to the actual cell number, and then calculates the degree of cell enrichment (PMID: 30479382). For example, for a certain cell type in a certain group, the number of this cell type in the remaining three groups is used as a control for calculation. In a word, it calculates the degree of enrichment or reduction of a single group relative to the other three groups. We have revised the detailed description in the figure legend.

(5)The decrease in immune cell communication in severe OSA shown in Figure 5 is interesting, but there seems to be a lack of specific changes in ligand receptor molecular results.

Response: Thank you for your comment. We have supplemented the differential ligands and receptor molecules. Interestingly, the mild OSA group upregulated many ligand receptor signaling molecules compared to the control group, such as HLA-E−CD94:NKG2A ,

ICAM1−(ITGAL+ITGB2), TGFB1−(TGFBR1+TGFBR2), VEGFA−VEGFR1, ANXA1−FPR1, CD86−CD28, etc. These reflect the immune activation, increased inflammation, and proliferation signals that accompany the early onset of OSA. However, in the comparison between severe OSA and mild OSA, multiple groups of ligand receptor signals were found to be downregulated, such as COL9A3−CD44, HLA-DOB−CD4, LGALS9−CD45, and TGFB1−(ACVR1B+TGFBR2), etc. indicating that severe OSA did undergo significant changes in cellular interactions. We have added these results, please refer to the revised manuscript for specific content. Thank you.

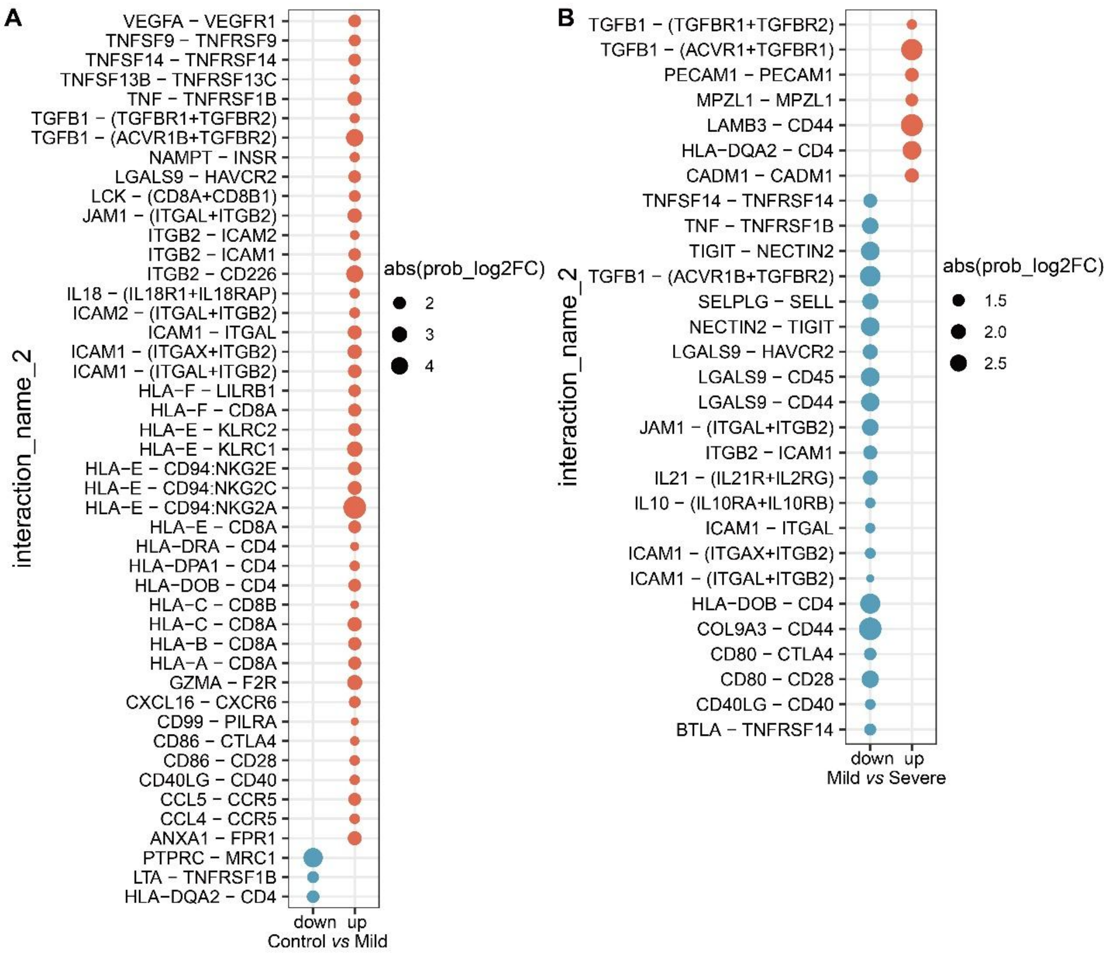

(A) Differences in ligand and receptor pairs between the control group and the mild OSA group. (B) Differences in ligand and receptor pairs between mild OSA and severe OSA groups.

(6)The TCR imprint analysis results in Figure 6 seem to lack significant statistical significance, and the same issue also appears in the SHM analysis results.

Response: Thank you for your comment. We have added statistical tests in TCR annotation analysis and SHM analysis. Overall, there was a significant increase in TCR imprints in the severe OSA group. In addition, compared to the control group, the mild and severe OSA groups did

indeed show a significant increase in SHM, representing the potential impact of infection experience on antibody maturation. Interestingly, the SHM in the severe OSA group did not show significant changes compared to the control group, but showed a significant decrease compared to mild and moderate OSA, indicating that children in the severe OSA group may have impaired antibody maturation. Please refer to the revised manuscript and images, thank you.

(7)The author mentioned that HIF1A inhibits HLA or interferon signaling, but this is mainly based on correlation and some literature results. At least more discussion is needed here to support this result.

Response: Thank you for your comment. We have supplemented the protein level experiment of HIF1A (immunofluorescence) and indeed found that HIF1A is significantly upregulated in the severe OSA group compared to the control and mild OSA groups. Besides, we have added a detailed discussion on the immune suppression mechanism mediated by HIF1A, as this part of the results has been confirmed in previous published studies that hypoxia induced upregulation of HIF1A at the cellular level can lead to downregulation of HLA molecules and immune signals (PMID: 34456926, PMID: 38920249, PMID: 18694997). These studies overall indicate that hypoxia induced HIF1A inhibits immune activation and maturation of dendritic cells, monocytes, and T cells at least at the cell culture level (PMID: 34456926, PMID: 38920249, PMID: 18694997). Our adenoid sequencing data indeed showed similar mRNA levels, and HIF1A showed a significant negative correlation with immune signaling. Therefore, we propose that similar HIF1A-mediated immune suppression may exist in children with real adenoid hypertrophy. However, considering that our focus is mainly on analyzing the correlation between single-cell omics data of different OSA severity levels and clinical outcomes, we can only provide a reasonable and sufficient discussion on this mechanism, and look forward to further mechanism research along these clues by future researchers. Please refer to the revised manuscript for details, thanks again.

(8)Although important and novel enough, the results of this study are mainly descriptive analysis, so excessive interpretation needs to be improved.

Response: Thank you for your comment. We have revised many of the result descriptions in the

manuscript, reducing excessive interpretation and limiting many of the result descriptions to the mRNA level. Please refer to the revised manuscript, thank you.

(9)The terms "adenoidal hypertrophy" and "adenoid hypertrophy" are used interchangeably. Please use abbreviation or choose one and use it consistently throughout the manuscript. Similarly, "scRNA-seq", "single-cell RNA-seq" and "single cell RNA-seq" are all used. "scRNA-seq", "scTCR/BCR-seq" and "scTCR/scBCR sequencing" and "Single cell V(D)J-seq" are all used. "scRNA-seq".

Response: Thank you for your comment. We have made unified revisions to these words and abbreviations, such as using “adenoid hypertrophy”, “single cell RNA-seq”, and “scTCR/BCR-seq” uniformly. Please refer to the revised manuscript, thank you.

(10)Check the usage specifications of acronyms (e.g., polysomnography, HSP and PBMCs) and providing a acronyms list is better.

Response: Thank you for your comment. We have revised all acronyms and provided supplementary tables. Please refer to the revised manuscript. Thank you.

(11)In the discussion section of the article, the results of differentially expressed genes and other articles have been explored and studied in existing literature. Currently, the main focus is on gene functions, but the association value with diseases such as OSA is not clear.

Response: Thank you for your comment. We have added the association between differentially expressed genes or key molecules and the progression of OSA in the discussion section. Please refer to the revised manuscript. Thank you.

(12) Considering that the article has collected and studied samples of different disease degrees, it is suggested that the discussion should strengthen the exploration of the occurrence and progression of OSA to highlight the value of this research in the fine classification of disease degrees.

Response: Thank you for your comment. We have further discussed the progression related outcomes of OSA, particularly the genes, pathways, regulatory networks, and cellular

communication related to the differences between control and mild OSA, as well as between mild and severe OSA. These contents may provide important clues for the occurrence of OSA in disease aggravation. Please refer to the revised manuscript for details. Thank you.

16th Feb 2026

Dear Dr. Qin,

Thank you for the submission of your revised manuscript to EMBO Molecular Medicine. I am pleased to inform you that we will be able to accept your manuscript pending the following final amendments:

1) Authors:
- In our submission system but not on the manuscript title page Feiqiu Wen is listed as a contributing author. Please correct.
- Please provide institutional email address for the co-corresponding author Hongguang Pan in our submission system and the title page of the manuscript.
2) Author Checklist: Please complete all relevant fields in the Author Checklist.
3) Figures: Please make sure that the highlight boxes in Figure 2C represent the corresponding areas in the image. Currently, the first two panels overlap; however, this is not how the highlight boxes have been drawn on the image.
4) Source data: Please provide source data for Figure 1J, 2B and 6D.
5) In the main manuscript file, please do the following:
- Please address all comments suggested by our data editors listed below:
o Figure legends:
1. Please note that the legend for figure 1M is missing in the manuscript. This needs to be rectified.
2. Please define the annotated p values ****/***/**/* as well as provide the exact p-values for the same in the legend of figure 1H as appropriate.
3. Please note that the exact p values are not provided in the legends of figures 1C, J, K, L; 2B, E-H; 3I-L; 4K-N; 5D-F, H-K; 6D, F, G; EV1 D, EV3 E.
4. Please indicate the statistical test used for data analysis in the legends of figures 1H, 4F, 6F.
5. Please note that the box plots need to be defined in terms of minima, maxima, centre, bounds of box and whiskers, and percentile in the legends of figures 5D-F; 6F.
6. Please note that information related to n is missing in the legends of figures 1H, K, L; 5D-F; EV1 D.
7. Please note that the error bars are not defined in the legends of figures 1H, K, L; EV1 D.
8. Please note that the red arrows are not defined in the legend of figure 1B, 6D. This needs to be rectified.
- Please correct the order of the sections in the manuscript text to: Abstract / The Paper Explained / Introduction / Results / Discussion / Methods / Data Availability / Acknowledgements / Disclosure and Competing Interests Statement / References / Main Figure Legends / Tables / Expanded View Figure Legends.
- Please add callouts for Table EV3 and correct the callout of Fig. S5D to Appendix Figure S5D.
- In Methods, please include statement that the experiments involving human participants conformed to the principles set out in the WMA Declaration of Helsinki and the Department of Health and Human Services Belmont Report.
- Author contributions: Please remove it from the manuscript and specify author contributions in our submission system. CRediT has replaced the traditional author contributions section because it offers a systematic machine-readable author contributions format that allows for more effective research assessment. You are encouraged to use the free text boxes beneath each contributing author's name to add specific details on the author's contribution. More information is available in our guide to authors:
https://www.embopress.org/page/journal/17574684/authorguide#authorshipguidelines
- Please remove the sentence "Other unique resources generated in this study may be available from the lead contact under reasonable requirements" form data availability statement.
6) Funding: Please make sure that all information about funding is complete in our submission system and in the manuscript. Currently, Guangdong Basic and Applied Basic Research Foundation (2023A1515110489), Guangdong High-level Hospital Construction Fund Clinical Research Project of Shenzhen Children's Hospital (LCYJ2022097) are missing in our submission system. Please correct.
7) Datasets: The EV datasets should be uploaded as one file per dataset and the corresponding legend should be added to each file. The appendix tables should be renamed Table EV1 - Table EV3 and uploaded as separate files, with the corresponding legend added to the top of each table. The abbreviations list should be removed, and the abbreviations should be incorporated into the manuscript text.
8) Appendix: The appendix figures should be compiled in one PDF file labelled "Appendix", the legends should be removed from the manuscript text and added to the appendix file, underneath the corresponding figures. A table of contents should be added, including page numbers.
9) Synopsis:
- Synopsis image: Please simplify the image and upload it as a high-resolution .jpeg/.png file 550 px-wide x 300-600 pixels high.
- Please check your synopsis text and image before submission with your revised manuscript. Please be aware that in the proof stage minor corrections only are allowed (e.g., typos).
10) As part of the EMBO Publications transparent editorial process (see our Editorial at http://embomolmed.embopress.org/content/2/9/329), EMBO Molecular Medicine will publish online a Review Process File (RPF) to accompany accepted manuscripts. This file will be published in conjunction with your paper and will include the anonymous referee reports, your point-by-point response and all pertinent correspondence relating to the manuscript. Let us know if you

want to remove or not any figures from it prior to publication. Please note that the Authors checklist will be published at the end of the RPF.
11) Please provide a point-by-point letter INCLUDING my comments as well as the reviewer's reports and your detailed responses (as Word file).

I look forward to reading a new revised version of your manuscript as soon as possible.

Yours sincerely,

Zeljko Durdevic

Zeljko Durdevic
Senior Editor
EMBO Molecular Medicine

*** Instructions to submit your revised manuscript ***

*** PLEASE NOTE *** As part of the EMBO Publications transparent editorial process initiative (see our Editorial at https://www.embopress.org/doi/pdf/10.1002/emmm.201000094), EMBO Molecular Medicine will publish online a Review Process File to accompany accepted manuscripts.

In the event of acceptance, this file will be published in conjunction with your paper and will include the anonymous referee reports, your point-by-point response and all pertinent correspondence relating to the manuscript. If you do NOT want this file to be published, please inform the editorial office at contact@embomolmed.org.

*****

When preparing your revised manuscript, please refer to our guidelines: https://link.springer.com/journal/44321/submission-guidelines#cms-Revised-submissions. We perform an initial quality control of all revised manuscripts before re-review; failure to include requested items will delay the evaluation of your revision.

We require:

1) A .docx formatted version of the manuscript text (including legends for main figures, EV figures and tables). Please make sure that the changes are highlighted to be clearly visible.

2) Individual production quality figure files as .eps, .tif, .jpg (one file per figure). For guidance, download the 'Figure Guide PDF': https://media.springernature.com/original/springer-cms/rest/v1/content/27825798/data/v1.

3) A .docx formatted letter INCLUDING the reviewers' reports and your detailed point-by-point responses to their comments. As part of the EMBO Press transparent editorial process, the point-by-point response is part of the Review Process File (RPF), which will be published alongside your paper.

4) A complete author checklist, which you can download from our author guidelines. Please insert information in the checklist that is also reflected in the manuscript. The completed author checklist will also be part of the RPF.

5) Please note that all corresponding authors are required to supply an ORCID ID for their name upon submission of a revised manuscript.

6) It is mandatory to include a 'Data Availability' section after the Materials and Methods. Before submitting your revision, primary datasets produced in this study need to be deposited in an appropriate public database, and the accession numbers and database listed under 'Data Availability'. Please remember to provide a reviewer password if the datasets are not yet public.

In case you have no data that requires deposition in a public database, please state so in this section. Note that the Data

Availability Section is restricted to new primary data that are part of this study.

7) For data quantification: please specify the name of the statistical test used to generate error bars and P values, the number (n) of independent experiments (specify technical or biological replicates) underlying each data point and the test used to calculate p-values in each figure legend. The figure legends should contain a basic description of n, P and the test applied. Graphs must include a description of the bars and the error bars (s.d., s.e.m.).

8) At EMBO Press we ask authors to provide source data for the main manuscript figures. You will receive a separate email with instructions for providing source data with your revised manuscript, including how to upload and organize the files.

9) Our journal encourages inclusion of *data citations in the reference list* to directly cite datasets that were re-used and obtained from public databases. Data citations in the article text are distinct from normal bibliographical citations and should directly link to the database records from which the data can be accessed. In the main text, data citations are formatted as follows: "Data ref: Smith et al, 2001" or "Data ref: NCBI Sequence Read Archive PRJNA342805, 2017". In the Reference list, data citations must be labeled with "[DATASET]". A data reference must provide the database name, accession number/identifiers and a resolvable link to the landing page from which the data can be accessed at the end of the reference.

10) We replaced Supplementary Information with Expanded View (EV) Figures and Tables that are collapsible/expandable online. A maximum of 5 EV Figures can be typeset. EV Figures should be cited as 'Figure EV1, Figure EV2" etc... in the text and their respective legends should be included in the main text after the legends of regular figures.

- For the figures that you do NOT wish to display as Expanded View figures, they should be bundled together with their legends in a single PDF file called *Appendix*, which should start with a short Table of Content. Appendix figures should be referred to in the main text as: "Appendix Figure S1, Appendix Figure S2" etc.

- Additional Tables/Datasets should be labeled and referred to as Table EV1, Dataset EV1, etc. Legends have to be provided in a separate tab in case of .xls files. Alternatively, the legend can be supplied as a separate text file (README) and zipped together with the Table/Dataset file.

11) The paper explained: EMBO Molecular Medicine articles are accompanied by a summary of the articles to emphasize the major findings in the paper and their medical implications for the non-specialist reader. Please provide a draft summary of your article highlighting

- the medical issue you are addressing,

- the results obtained and

- their clinical impact.

This may be edited to ensure that readers understand the significance and context of the research. Please refer to any of our published articles for an example.

12) Author contributions: You will be asked to provide CRediT (Contributor Role Taxonomy) terms in the submission system. These replace a narrative author contribution section in the manuscript.

13) A Conflict of Interest statement should be provided in the main text.

14) Every published paper includes a 'Synopsis' to further enhance discoverability. Synopses are displayed on the journal webpage and are freely accessible to all readers. They include a short stand first (maximum of 300 characters, including space) as well as 2-5 one-sentences bullet points that summarizes the paper. Please write the bullet points to summarize the key NEW findings. They should be designed to be complementary to the abstract - i.e. not repeat the same text. We encourage inclusion of key acronyms and quantitative information (maximum of 30 words / bullet point). Please use the passive voice. Please attach these in a separate file or send them by email, we will incorporate them accordingly.

Please also suggest a striking image or visual abstract to illustrate your article as a PNG file 550 px wide x 300-600 px high.

15) Include a Reagents and Tools Table as part of the Methods section, which can be downloaded from our author guidelines.

EMBO Molecular Medicine has a "scooping protection" policy, whereby similar findings that are published by others during review or revision are not a criterion for rejection. Should you decide to submit a revised version, I do ask that you get in touch after three months if you have not completed it, to update us on the status.

*Additional important information regarding Figures

Each figure should be given in a separate file and should have the following resolution:
Graphs 800-1,200 DPI
Photos 400-800 DPI
Colour (only CMYK) 300-400 DPI"

Figures are not edited by the production team. All lettering should be the same size and style; figure panels should be indicated by capital letters (A, B, C etc). Gridlines are not allowed except for log plots. Figures should be numbered in the order of their appearance in the text with Arabic numerals. Each Figure must have a separate legend and a caption is needed for each panel.

*Additional important information regarding figures and illustrations can be found at
https://media.springernature.com/original/springer-cms/rest/v1/content/27825798/data/v1

***** Reviewer's comments *****

Referee #1 (Remarks for Author):

The authors have sufficiently addressed the previous comments.

Referee #2 (Comments on Novelty/Model System for Author):

This article provides novel immunological data for the study of OSA, which offers valuable references for both the scientific research and clinical treatment.

The authors addressed the remaining editorial issues.

18th Mar 2026

Dear Dr. Qin,

We are pleased to inform you that your manuscript is accepted for publication and is now being sent to our publisher to be included in the next available issue of EMBO Molecular Medicine.

Your manuscript will be processed for publication by EMBO Press. It will be copy edited and you will receive page proofs prior to publication. Please note that you will be contacted by Springer Nature Author Services to complete licensing and payment information.

You may qualify for financial assistance for your publication charges - either via a Springer Nature fully open access agreement or an EMBO initiative. Check your eligibility: https://link.springer.com/journal/44321/how-to-publish-with-us

Should you be planning a Press Release on your article, please get in contact with embo_production@springernature.com as early as possible in order to coordinate publication and release dates.

If you have any questions, please do not hesitate to contact the Editorial Office. Thank you for your contribution to EMBO Molecular Medicine.

Yours sincerely,
Zeljko Durdevic

Zeljko Durdevic
Senior Editor
EMBO Molecular Medicine

>>> Please note that it is EMBO Molecular Medicine policy for the transcript of the editorial process (containing referee reports and your response letter) to be published as an online supplement to each paper. If you do NOT want this, you will need to inform the Editorial Office via email immediately. More information is available here: https://link.springer.com/partners/embo-press/editorial-policies#Peer%20review

EMBO Press Author Checklist

Corresponding Author Name: Shijie Qin	
Journal Submitted to: EMBO Molecular Medicine	
Manuscript Number: EMM-2025-22660	

USEFUL LINKS FOR COMPLETING THIS FORM
The EMBO Journal - Author Guidelines
EMBO Reports - Author Guidelines
Molecular Systems Biology - Author Guidelines
EMBO Molecular Medicine - Author Guidelines

Reporting Checklist for Life Science Articles (updated January
This checklist is adapted from Materials Design Analysis Reporting (MDAR) Checklist for Authors. MDAR establishes a minimum set of requirements in transparent reporting in the life sciences (see Statement of Task: 10.31222/osf.io/9sm4x). Please follow the journal's guidelines in preparing your
Please note that a copy of this checklist will be published alongside your article.

Abridged guidelines for figures
1. Data
The data shown in figures should satisfy the following conditions:
- the data were obtained and processed according to the field's best practice and are presented to reflect the results of the experiments in an accurate and unbiased manner.
- ideally, figure panels should include only measurements that are directly comparable to each other and obtained with the same assay.
- plots include clearly labeled error bars for independent experiments and sample sizes. Unless justified, error bars should not be shown for technical
- if n<5, the individual data points from each experiment should be plotted. Any statistical test employed should be justified.
- Source Data should be included to report the data underlying figures according to the guidelines set out in the authorship guidelines on Data

2. Captions
Each figure caption should contain the following information, for each panel where they are relevant:
- a specification of the experimental system investigated (eg cell line, species name).
- the assay(s) and method(s) used to carry out the reported observations and measurements.
- an explicit mention of the biological and chemical entity(ies) that are being measured.
- an explicit mention of the biological and chemical entity(ies) that are altered/varied/perturbed in a controlled manner.
- the exact sample size (n) for each experimental group/condition, given as a number, not a range;
- a description of the sample collection allowing the reader to understand whether the samples represent technical or biological replicates (including how many animals, litters, cultures, etc.).
- a statement of how many times the experiment shown was independently replicated in the laboratory.
- definitions of statistical methods and measures:
  - common tests, such as t-test (please specify whether paired vs. unpaired), simple $\chi2$ tests, Wilcoxon and Mann-Whitney tests, can be unambiguously identified by name only, but more complex techniques should be described in the methods section;
  - are tests one-sided or two-sided?
  - are there adjustments for multiple comparisons?
  - exact statistical test results, e.g., P values = x but not P values < x;
  - definition of 'center values' as median or average;
  - definition of error bars as s.d. or s.e.m.

Please complete ALL of the questions below.
Select "Not Applicable" only when the requested information is not relevant for your study.

Materials

Newly Created Materials	Information included in the manuscript?	In which section is the information available? (Reagents and Tools Table, Materials and Methods, Figures, Data Availability Section)
New materials and reagents need to be available; do any restrictions apply?	Yes	Reagents and Tools Table

Antibodies	Information included in the manuscript?	In which section is the information available? (Reagents and Tools Table, Materials and Methods, Figures, Data Availability Section)
For **antibodies** provide the following information:<br>- Commercial antibodies: RRID (if possible) or supplier name, catalogue number and or/clone number<br>- Non-commercial: RRID or citation	Yes	Reagents and Tools Table

DNA and RNA sequences	Information included in the manuscript?	In which section is the information available? (Reagents and Tools Table, Materials and Methods, Figures, Data Availability Section)
**Short novel DNA or RNA including primers, probes**: provide the sequences.	Yes	Reagents and Tools Table

Cell materials	Information included in the manuscript?	In which section is the information available? (Reagents and Tools Table, Materials and Methods, Figures, Data Availability Section)
**Cell lines:** Provide species information, strain. Provide accession number in repository **OR** supplier name, catalog number, clone number, and/**OR** RRID.	Not Applicable	
**Primary cultures:** Provide species, strain, sex of origin, genetic modification status.	Not Applicable	
Report if the cell lines were recently **authenticated** (e.g., by STR profiling) and tested for mycoplasma contamination.	Not Applicable	

Experimental animals	Information included in the manuscript?	In which section is the information available? (Reagents and Tools Table, Materials and Methods, Figures, Data Availability Section)
**Laboratory animals or Model organisms:** Provide species, strain, sex, age, genetic modification status. Provide accession number in repository **OR** supplier name, catalog number, clone number, **OR** RRID.	Not Applicable	
**Animal observed in or captured from the field:** Provide species, sex, and age where possible.	Not Applicable	
Please detail **housing and husbandry conditions**.	Not Applicable	

Plants and microbes	Information included in the manuscript?	In which section is the information available? (Reagents and Tools Table, Materials and Methods, Figures, Data Availability Section)
**Plants:** provide species and strain, ecotype and cultivar where relevant, unique accession number if available, and source (including location for collected wild specimens).	Not Applicable	
**Microbes:** provide species and strain, unique accession number if available, and source.	Not Applicable	

Human research participants	Information included in the manuscript?	In which section is the information available? (Reagents and Tools Table, Materials and Methods, Figures, Data Availability Section)
If collected and within the bounds of privacy constraints report on age, sex and gender or ethnicity for all study participants.	Yes	Materials and Methods

Core facilities	Information included in the manuscript?	In which section is the information available? (Reagents and Tools Table, Materials and Methods, Figures, Data Availability Section)
If your work benefited from core facilities, was their service mentioned in the acknowledgments section?	Yes	Materials and Methods

Design

Study protocol	Information included in the manuscript?	In which section is the information available? (Reagents and Tools Table, Materials and Methods, Figures, Data Availability Section)
If study protocol has been **pre-registered, provide DOI in the manuscript**. For clinical trials, provide the trial registration number **OR** cite DOI.	Yes	Materials and Methods
Report the **clinical trial registration number** (at ClinicalTrials.gov or equivalent), where applicable.	Not Applicable	

Laboratory protocol	Information included in the manuscript?	In which section is the information available? (Reagents and Tools Table, Materials and Methods, Figures, Data Availability Section)
Provide DOI OR other citation details if **external detailed step-by-step protocols** are available.	Yes	Materials and Methods

Experimental study design and statistics	Information included in the manuscript?	In which section is the information available? (Reagents and Tools Table, Materials and Methods, Figures, Data Availability Section)
Include a statement about **sample size** estimate even if no statistical methods were used.	Yes	Materials and Methods, Figures
Were any steps taken to minimize the effects of subjective bias when allocating animals/samples to treatment (e.g. **randomization procedure**)? If yes, have they been described?	Yes	Materials and Methods
Include a statement about **blinding** even if no blinding was done.	Not Applicable	
Describe **inclusion/exclusion criteria** if samples or animals were excluded from the analysis. Were the criteria pre-established?<br><br>If sample or data points were omitted from analysis, report if this was due to attrition or intentional exclusion and provide justification.	Yes	Materials and Methods
For every figure, are **statistical tests** justified as appropriate? Do the data meet the assumptions of the tests (e.g., normal distribution)? Describe any methods used to assess it. Is there an estimate of variation within each group of data? Is the variance similar between the groups that are being statistically compared?	Yes	Figures

Sample definition and in-laboratory replication	Information included in the manuscript?	In which section is the information available? (Reagents and Tools Table, Materials and Methods, Figures, Data Availability Section)
In the figure legends: state number of times the experiment was **replicated** in laboratory.	Yes	Figures
In the figure legends: define whether data describe **technical or biological replicates**.	Yes	Figures

Ethics

Ethics	Information included in the manuscript?	In which section is the information available? (Reagents and Tools Table, Materials and Methods, Figures, Data Availability Section)
Studies involving **human participants**: State details of **authority granting ethics approval** (IRB or equivalent committee(s), provide reference number for approval.	Yes	Materials and Methods
Studies involving **human participants**: Include a statement confirming that **informed consent** was obtained from all subjects and that the experiments conformed to the principles set out in the WMA Declaration of Helsinki and the Department of Health and Human Services Belmont Report.	Yes	Materials and Methods
Studies involving **human participants: For publication of patient photos**, include a statement confirming that consent to publish was obtained.	Not Applicable	
Studies involving experimental **animals**: State details of **authority granting ethics approval** (IRB or equivalent committee(s), provide reference number for approval. Include a statement of compliance with ethical regulations.	Not Applicable	
Studies involving **specimen and field samples:** State if relevant **permits** obtained, provide details of authority approving study; if none were required, explain why.	Yes	Materials and Methods

Dual Use Research of Concern (DURC)	Information included in the manuscript?	In which section is the information available? (Reagents and Tools Table, Materials and Methods, Figures, Data Availability Section)
Could your study fall under dual use research restrictions? Please check biosecurity documents and list of **select agents and toxins** (CDC): https://www.selectagents.gov/sat/list.htm	Not Applicable	
If you used a select agent, is the security level of the lab appropriate and reported in the manuscript?	Not Applicable	
If a study is subject to dual use research of concern regulations, is the name of the **authority granting approval and reference number** for the regulatory approval provided in the manuscript?	Not Applicable	

Reporting

The MDAR framework recommends adoption of discipline-specific guidelines, established and endorsed through community initiatives. Journals have their own policy about requiring specific guidelines and recommendations to complement MDAR.

Adherence to community standards	Information included in the manuscript?	In which section is the information available? (Reagents and Tools Table, Materials and Methods, Figures, Data Availability Section)
State if relevant guidelines or checklists (e.g., **ICMJE, MIBBI, ARRIVE, PRISMA**) have been followed or provided.	Not Applicable	
For **tumor marker prognostic studies,** we recommend that you follow the **REMARK** reporting guidelines (see link list at top right). See author guidelines, under 'Reporting Guidelines'. Please confirm you have followed these guidelines.	Not Applicable	
For **phase II and III randomized controlled trials**, please refer to the **CONSORT** flow diagram (see link list at top right) and submit the CONSORT checklist (see link list at top right) with your submission. See author guidelines, under 'Reporting Guidelines'. Please confirm you have submitted this list.	Not Applicable	

Data Availability

Data availability	Information included in the manuscript?	In which section is the information available? (Reagents and Tools Table, Materials and Methods, Figures, Data Availability Section)
Have **primary datasets** been deposited according to the journal's guidelines (see 'Data Deposition' section) and the respective accession numbers provided in the Data Availability Section?	Yes	Data Availability
Were **human clinical and genomic datasets** deposited in a public access-controlled repository in accordance to ethical obligations to the patients and to the applicable consent agreement?	Not Applicable	
Are **computational models** that are central and integral to a study available without restrictions in a machine-readable form? Were the relevant accession numbers or links provided?	Not Applicable	
If publicly available data were reused, provide the respective **data citations in the reference list**.	Not Applicable	
